# Modeling and forecasting the spread of COVID-19 with stochastic and deterministic approaches: Africa and Europe

**DOI:** 10.1186/s13662-021-03213-2

**Published:** 2021-01-20

**Authors:** Abdon Atangana, Seda İğret Araz

**Affiliations:** 1 Institute for Groundwater Studies, Faculty of Natural and Agricultural Sciences, University of the Free State, Bloemfontein, South Africa; 2 Department of Medical Research, China Medical University Hospital, China Medical University, Taichung, Taiwan; 3 Department of Mathematic Education, Faculty of Education, Siirt University, Siirt, 56100 Turkey

**Keywords:** Statistical analysis, Extended blancmange function, Stochastic model, COVID-19 spread with waves, Modified numerical scheme

## Abstract

Using the existing collected data from European and African countries, we present a statistical analysis of forecast of the future number of daily deaths and infections up to 10 September 2020. We presented numerous statistical analyses of collected data from both continents using numerous existing statistical theories. Our predictions show the possibility of the second wave of spread in Europe in the worse scenario and an exponential growth in the number of infections in Africa. The projection of statistical analysis leads us to introducing an extended version of the well-blancmange function to further capture the spread with fractal properties. A mathematical model depicting the spread with nine sub-classes is considered, first converted to a stochastic system, where the existence and uniqueness are presented. Then the model is extended to the concept of nonlocal operators; due to nonlinearity, a modified numerical scheme is suggested and used to present numerical simulations. The suggested mathematical model is able to predict two to three waves of the spread in the near future.

## Introduction

Interdisciplinary research is the way forward for mankind to be in control of its environment. Of course they will not be able to have total control since the nature within which they live is full of uncertainties, many complex phenomena that have not been yet understood with the current collections of knowledge and technology. For example, we cannot explicitly and confidently explain what is happening at the Bermuda Triangle, although many studies have been done around this place, some believe it is a devil’s triangle. There are many other natural occurrences that could not be explained so far with our knowledge. But it has been proven that putting together several concepts from different academic fields could provide better results. COVID-19 is an invisible enemy that left humans with no choice than to put all their efforts from all backgrounds with the aim to protect the survival of their kind. Many souls have been taken, many humans have been infected and some recovered, but still the spread has not yet reached its peak in many countries. While in some countries the curve of daily new infected has nearly reached zero, in others the spread is increasing exponentially. For some statistical analysis, we investigated daily cases of infections and deaths due to the COVID-19 spread that occurred in 54 countries in the European continent and 47 countries in the African continent from the beginning of the outbreak to 15 June 2020. To do this, we used the available data on the website of the World Health Organization (WHO) [1, 2]. Although mathematicians cannot provide vaccine or cure the disease in an infected person, they can use their mathematical tools to foresee what could possibly happen in the near future with some limitations [3–14]. With the new trend of spread, it is possible that the world will face a second wave of COVID-19 spread, this will be the aim of our work.

The paper is organized as follows. In Sect. 2, we present the definitions of differential and integral operators where singular and nonsingular kernels are used. In Sect. 3, the parameter estimations are presented for the infected and deaths in Africa and Europe using the Box–Jenkins model. In Sect. 4, the simulations for smoothing method for the infected and deaths in Africa and Europe are presented. In Sect. 5, the predictions about the cases of infections and deaths in Africa and Europe are provided. In Sect. 6, we give an analysis of COVID-19 spread based on fractal interpolation and fractal dimension. In Sect. 7, existence and uniqueness for a mathematical model with stochastic component are investigated. Also the numerical simulations for such a model are depicted. In Sect. 8, we present a modified scheme based on the Newton polynomial. In Sect. 9, we provide numerical solutions for the suggested COVID-19 model with different differential operators.

## Differential and integral operators

In this section, we present some definitions of differential and integral operators with singular and nonsingular kernels. The fractional derivatives with power-law, exponential decay, and Mittag-Leffler kernel are given as follows:

### Definition 1

1 $$\begin{aligned}& {}_{0}^{C}D_{t}^{\alpha }f ( t ) = \frac{1}{\Gamma ( 1-\alpha ) } \int _{0}^{t}\frac{d}{d\tau }f ( \tau ) ( t-\tau ) ^{-\alpha }\,d\tau , \\& {}_{0}^{CF}D_{t}^{\alpha }f ( t ) = \frac{M ( \alpha ) }{1-\alpha } \int _{0}^{t}\frac{d}{d\tau }f ( \tau ) \exp \biggl[ -\frac{\alpha }{1-\alpha } ( t-\tau ) \biggr]\,d\tau , \\& {}_{0}^{ABC}D_{t}^{\alpha }f ( t ) = \frac{AB ( \alpha ) }{1-\alpha } \int _{0}^{t}\frac{d}{d\tau }f ( \tau ) E_{ \alpha } \biggl[ -\frac{\alpha }{1-\alpha } ( t-\tau ) ^{ \alpha } \biggr] \,d \tau . \end{aligned}$$

The fractional integrals with power-law, exponential decay, and Mittag-Leffler kernel are given as follows:

2 $$\begin{aligned}& {}_{0}^{C}J_{t}^{\alpha }f ( t ) = \frac{1}{\Gamma ( \alpha ) } \int _{0}^{t} ( t-\tau ) ^{\alpha -1}f ( \tau ) \,d \tau , \\& {}_{0}^{CF}J_{t}^{\alpha ,\beta }f ( t ) = \frac{1-\alpha }{M ( \alpha ) }f ( t ) + \frac{\alpha }{M ( \alpha ) }\int _{0}^{t}f ( \tau )\,d\tau , \\& {}_{0}^{AB}J_{t}^{\alpha ,\beta }f ( t ) = \frac{1-\alpha }{AB ( \alpha ) }f ( t ) + \frac{\alpha }{AB ( \alpha ) \Gamma ( \alpha ) } \int _{0}^{t} ( t-\tau ) ^{ \alpha -1}f ( \tau ) \,d \tau . \end{aligned}$$

The fractal-fractional derivatives with power-law kernel, exponential decay, and Mittag-Leffler kernel are given as follows:

3 $$\begin{aligned}& {}_{0}^{FFP}D_{t}^{\alpha ,\beta }f ( t ) = \frac{1}{\Gamma ( 1-\alpha ) }\frac{d}{dt^{\beta }} \int _{0}^{t}f ( \tau ) ( t-\tau ) ^{-\alpha }\,d \tau , \\& {}_{0}^{FFE}D_{t}^{\alpha ,\beta }f ( t ) = \frac{M ( \alpha ) }{1-\alpha } \frac{d}{dt^{\beta }} \int _{0}^{t}f ( \tau ) \exp \biggl[ - \frac{\alpha }{1-\alpha } ( t-\tau ) \biggr]\,d\tau , \\& {}_{0}^{FFM}D_{t}^{\alpha ,\beta }f ( t ) = \frac{AB ( \alpha ) }{1-\alpha } \frac{d}{dt^{\beta }} \int _{0}^{t}f ( \tau ) E_{\alpha } \biggl[ - \frac{\alpha }{1-\alpha } ( t-\tau ) ^{\alpha } \biggr]\,d\tau , \end{aligned}$$

where

4 $$ \frac{df ( t ) }{dt^{\beta }}=\lim_{t\rightarrow t_{1}} \frac{f ( t ) -f ( t_{1} ) }{t^{2-\beta }-t_{1}^{2-\beta }} ( 2-\beta ) . $$

The fractal-fractional integrals with power-law, exponential decay, and Mittag-Leffler kernel are as follows:

5 $$\begin{aligned}& {}_{0}^{FFP}J_{t}^{\alpha ,\beta }f ( t ) = \frac{1}{\Gamma ( \alpha ) } \int _{0}^{t} ( t-\tau ) ^{\alpha -1} \tau ^{1-\beta }f ( \tau )\,d\tau , \\& {}_{0}^{FFE}J_{t}^{\alpha ,\beta }f ( t ) = \frac{1-\alpha }{M ( \alpha ) }t^{1-\beta }f ( t ) + \frac{\alpha }{M ( \alpha ) } \int _{0}^{t}\tau ^{1-\beta }f ( \tau )\,d\tau , \\& {}_{0}^{FFM}J_{t}^{\alpha ,\beta }f ( t ) = \frac{1-\alpha }{AB ( \alpha ) }t^{1-\beta }f ( t ) + \frac{\alpha }{AB ( \alpha ) \Gamma ( \alpha ) } \int _{0}^{t} ( t-\tau ) ^{\alpha -1}\tau ^{1-\beta }f ( \tau )\,d\tau . \end{aligned}$$

## Box–Jenkin’s model development

Autoregressive integrated moving average (ARIMA) approach suggested by Box and Jenkins is one of the most powerful techniques used in time series analysis. The ARIMA model is composed of three parts. First, the autoregressive part is a linear regression which has a relation between past values and future values of data series; second, the integrated part expresses how many times the data series has to be differenced to obtain a stationary series; and the last one is the moving average part which has a relation between past forecast errors and future values of data series [14]. These processes can be presented by the models $\operatorname{AR} ( p ) $, $\operatorname{MA} ( q ) $, $\operatorname{ARMA} ( p,q ) $, and $\operatorname{ARIMA} ( p,d,q ) $. We should decide which model we will choose for our data series. To do this, partial autocorrelation (PACF) and the autocorrelation (ACF) are helpful to obtain parameters for the AR model and the MA model, respectively.

**Figure 1:**
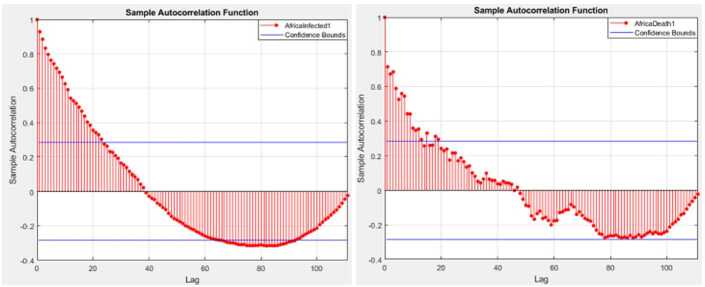
Autocorrelation function for the infected and deaths in Africa

**Figure 2:**
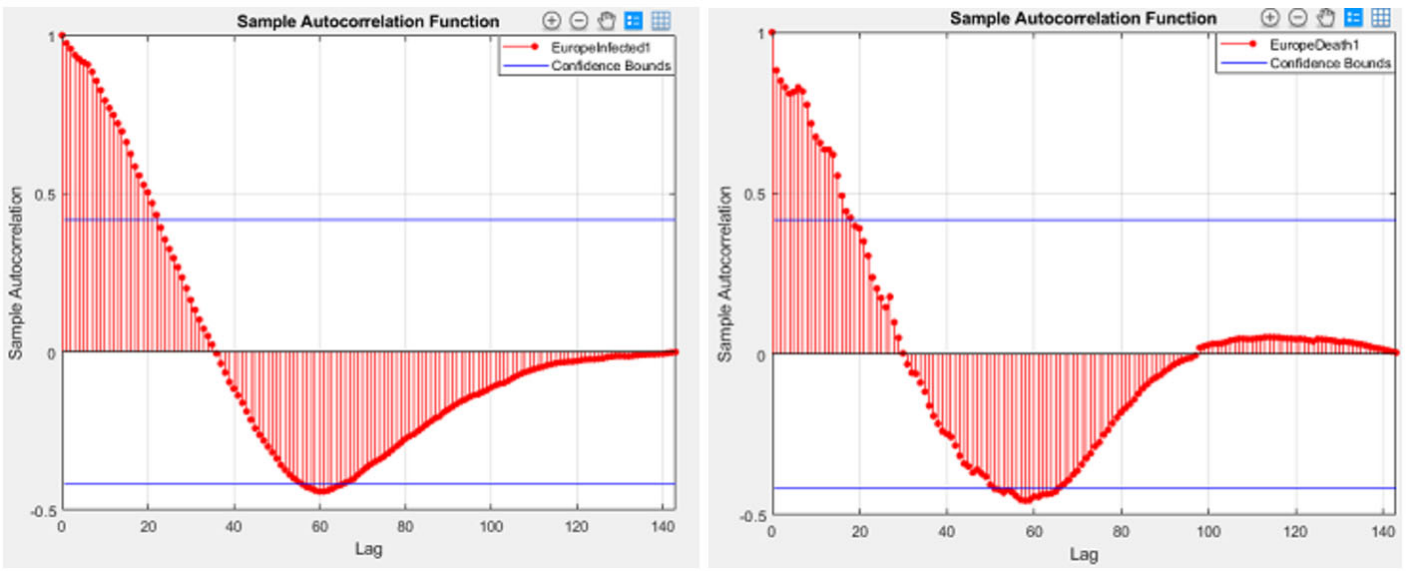
Autocorrelation function for the infected and deaths in Europe

Figures 1 and 2 depict graphs of autocorrelation functions for the infected and deaths in Africa and Europe.

Now we introduce these models. Let $Y_{t}$ be the value of the time series at time *t*. Time series as a *p*-order autoregressive process is as follows:

6 $$ Y_{t}=\delta +\varphi _{1}Y_{t-1}+\varphi _{2}Y_{t-2}+\cdots+\varphi _{p}Y_{t-p}+ \varepsilon _{t}, $$

which is shown as $\operatorname{AR} ( p ) $. Here, *δ* and $\varepsilon _{t} $ describe constant and error terms, respectively. Time series as a *q*th degree of moving average process is given by

7 $$ Y_{t}=\mu +\varepsilon _{t}+\theta _{1} \varepsilon _{t-1}+\theta _{2} \varepsilon _{t-2}+ \cdots+\theta _{q}\varepsilon _{t-q}, $$

which is shown as $\operatorname{MA} ( q ) $. The $\operatorname{ARMA} ( p,q ) $ expression is obtained as a combination of $\operatorname{AR} ( p ) $ and $\operatorname{MA} ( q ) $ equations:

8 $$ Y_{t}=\delta +\varphi _{1}Y_{t-1}+\varphi _{2}Y_{t-2}+\cdots+\varphi _{p}Y_{t-p}+ \varepsilon _{t}+\theta _{1}\varepsilon _{t-1}+ \theta _{2} \varepsilon _{t-2}+\cdots+\theta _{q} \varepsilon _{t-q}. $$

When the time series is not stationary, we take the difference *d* times to make it stationary. The $\operatorname{ARIMA} ( p,q ) $ model is given by

9 $$ \bigl( 1-\varphi _{1}l-\varphi _{2}l^{2}- \cdots-\varphi _{p}l^{p} \bigr) \Delta ^{d}Y_{t}= \delta +\varepsilon _{t}+\theta _{1} \varepsilon _{t-1}+\theta _{2}\varepsilon _{t-2}+\cdots+\theta _{q} \varepsilon _{t-q}. $$

**Figure 3:**
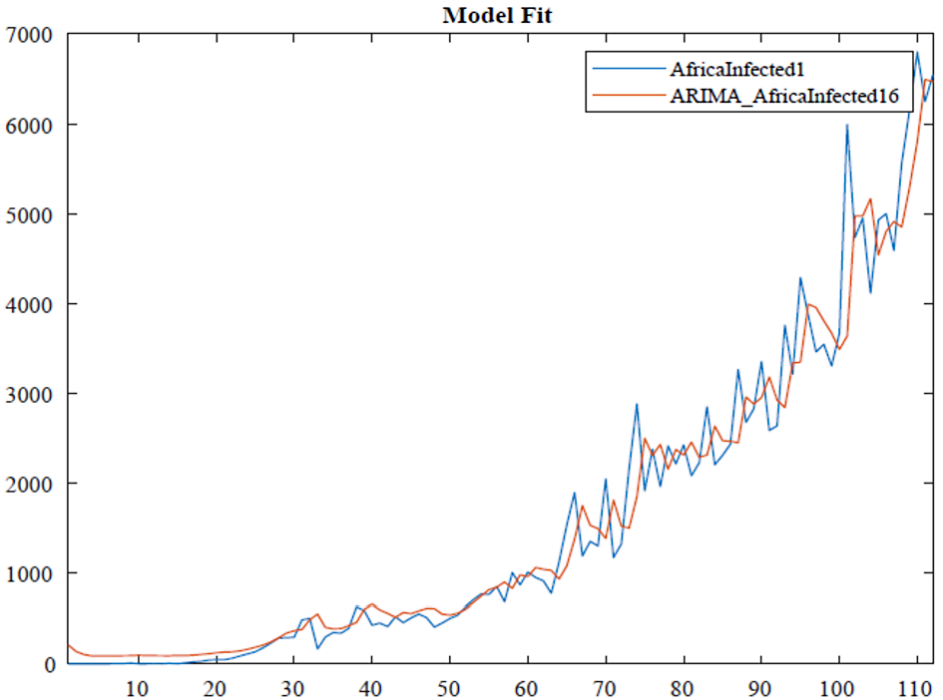
ARIMA model for the infected in Africa

**Figure 4:**
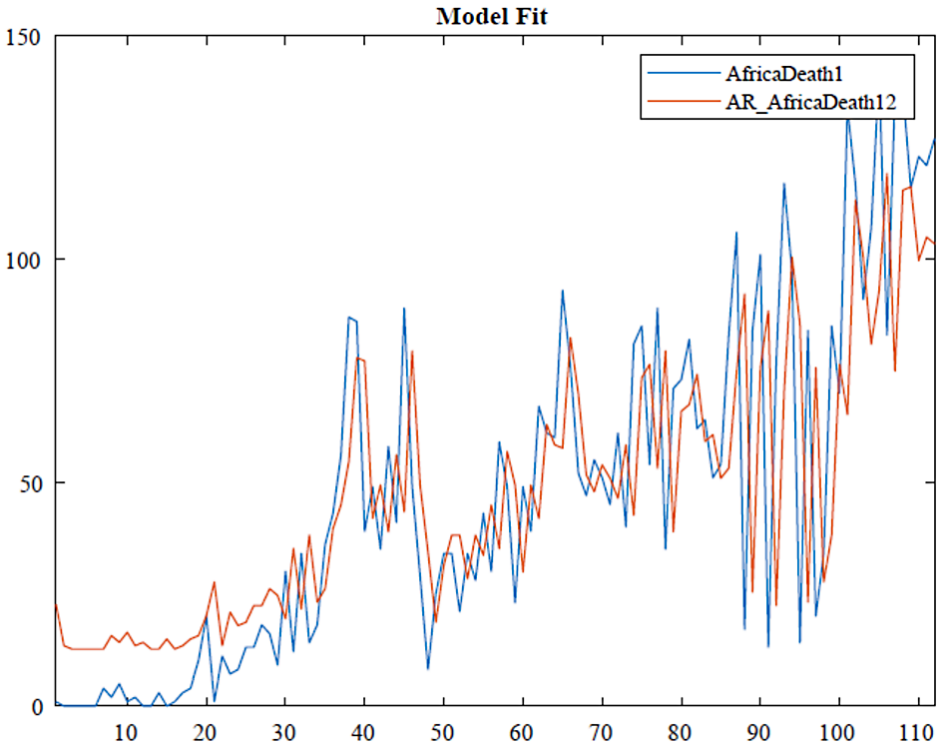
AR model for deaths in Africa

**Figure 5:**
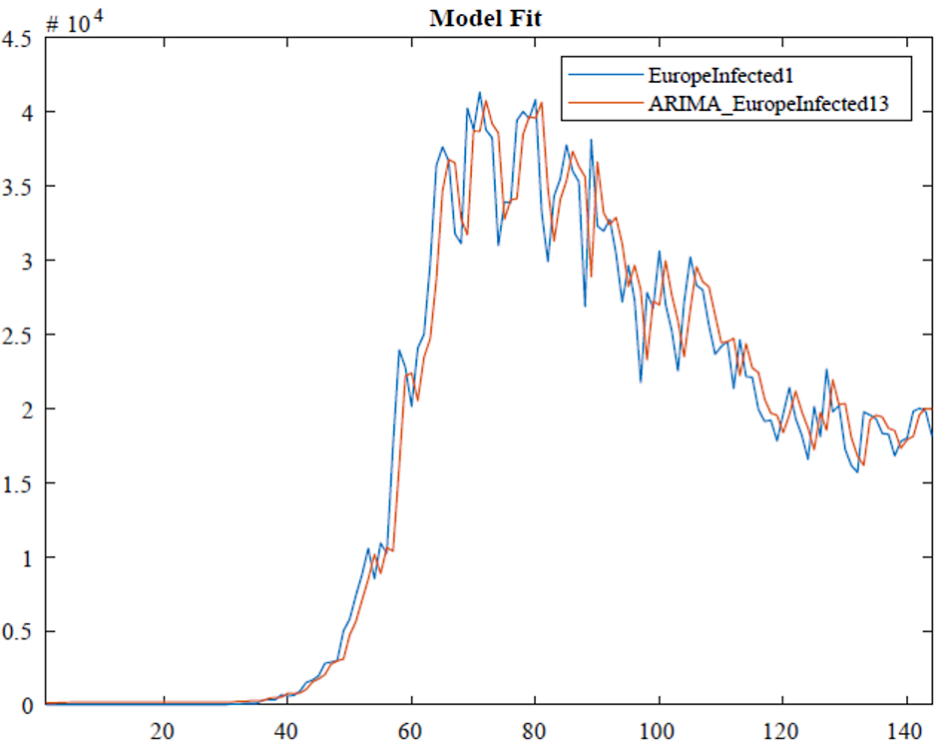
ARIMA model for the infected in Europe

**Figure 6:**
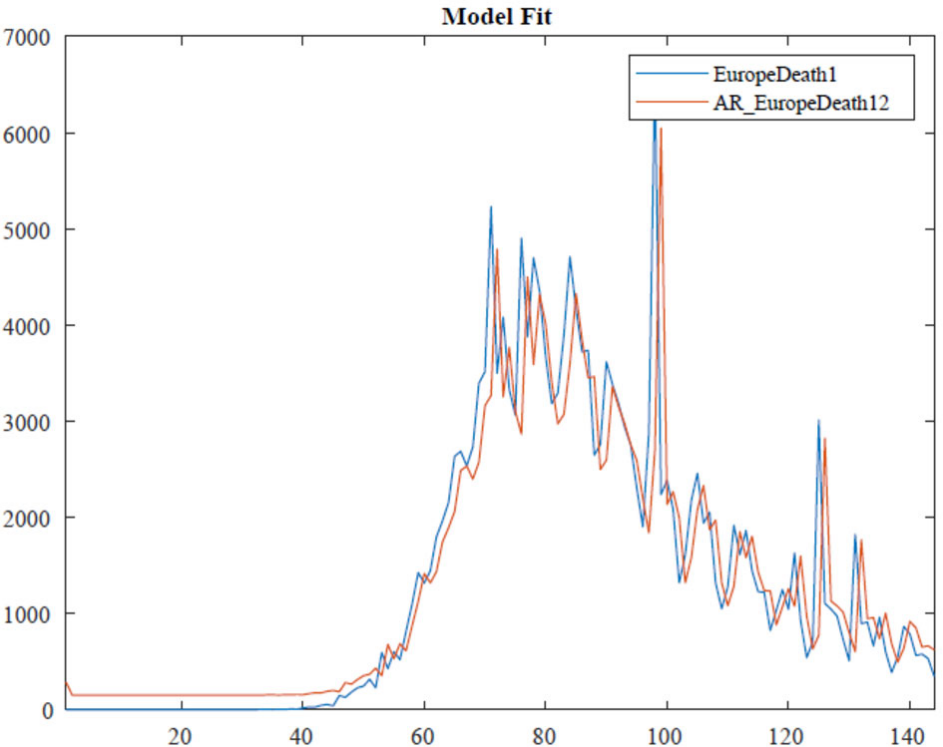
AR model for deaths in Europe

In the ARIMA technique, the model performance can be measured by using some criteria, for instance, Akaike information criteria(AIC), Bayesian information criteria(BIC). Here, we benefit from the Akaike information criteria given as follows:

10 $$\begin{aligned}& AIC =-2\log ( l ) +2k, \\& BIC =-2\log ( l ) +k\ln n, \end{aligned}$$

where *l* states likelihood of the data, *n* is the number of data points, and *k* also defines the intercept of the ARIMA model. The numerical simulation are depicted in Figs. 3, 4, 5 and 6.

According to data series for the infected in Africa, we use the $\operatorname{ARIMA} ( 2,1,0 ) $ model which is given by

11 $$ \bigl( 1-\varphi _{1}l-\varphi _{2}l^{2} \bigr) ( 1-l ) Y_{t}=c+ \varepsilon _{t}. $$

Here,

12 $$\begin{aligned}& AIC =1670.1734, \\& BIC =1680.9388. \end{aligned}$$

In Table 1, we give parameter estimation for infections in Africa.

**Table 1:** Model estimation for infections in Africa

Parameter	Value	Standard error	TStatistic
Constant	89.2032	56.6511	1.5746
AR{1}	−0.44796	0.099221	−4.5147
AR{2}	−0.17789	0.068294	−2.6047
Variance	168,446.2911	12,738.3089	13.2236

According to data series for deaths in Africa, we use the $\operatorname{AR} ( 1 ) $ model which is given by

13 $$ ( 1-\varphi _{1}l ) Y_{t}=c+\varepsilon _{t}. $$

Here,

14 $$\begin{aligned}& AIC =1056.6482, \\& BIC =1064.7768. \end{aligned}$$

In Table 2, we give parameter estimation for deaths in Africa.

**Table 2:** Model estimation for deaths in Africa

Parameter	Value	Standard error	TStatistic
Constant	12.581	6.0023	2.096
AR{1}	0.75094	0.082701	9.0802
Variance	694.3043	92.168	7.533

According to data series for the infected in Europe, we use the $\operatorname{ARIMA} ( 2,1,1 ) $ model which is given by

15 $$ \bigl( 1-\varphi _{1}l-\varphi _{2}l^{2} \bigr) ( 1-l ) Y_{t}=c+ ( 1+\theta _{1}l ) \varepsilon _{t}. $$

Here,

16 $$\begin{aligned}& AIC =2690.5358, \\& BIC =2705.2796. \end{aligned}$$

In Table 3, we give parameter estimation for the infected in Europe.

**Table 3:** Model estimation for the infected in Europe

Parameter	Value	Standard error	TStatistic
Constant	83.7826	118.7108	0.70577
AR{1}	0.3216	0.59303	0.5423
AR{2}	0.035772	0.16277	0.21977
MA{1}	−0.53222	0.58359	−0.91197
Variance	7,214,609.9182	569,786.6944	12.6619

According to data series for deaths in Europe, we use the $\operatorname{AR} ( 1 ) $ model which is given by

17 $$ ( 1-\varphi _{1}l ) Y_{t}=c+\varepsilon _{t}. $$

Here,

18 $$\begin{aligned}& AIC =1670.1734, \\& BIC =1680.9388. \end{aligned}$$

In Table 4, we give parameter estimation for deaths in Europe.

**Table 4:** Model estimation for deaths in Europe

Parameter	Value	Standard error	TStatistic
Constant	151.4852	163.967	0.92388
AR{1}	0.8865	0.041096	21.5714
Variance	460,062.22	27,485.093	16.7386

## Brown’s exponential smoothing method

Brown’s linear exponential smoothing is one type of double exponential smoothing based on two different smoothed series. The formula is composed of an extrapolation of a line through the two centers. The Brown exponential smoothing method is helpful to model the time series having trend but no seasonality.

For non-adaptive Brown exponential smoothing, the procedure can be described as follows.

Firstly, we start with the following initialization:

1. $S_{0}=u_{0}$,
2. $T_{0}=u_{0}$,
3. $a_{0}=2S_{0}-T_{0}$,
4. $F_{1}=a_{0}+b_{0}$.

Then we have the following calculations:

1. $S_{t}=\alpha u_{t}+ ( 1-\alpha ) S_{t-1}$,
2. $T_{t}=\alpha S_{t}+ ( 1-\alpha ) T_{t-1}$,
3. $a_{t}=2S_{t}+T_{t}63$,
4. $\alpha ( S_{t}-T_{t} ) = ( 1-\alpha ) b_{t}$,
5. $F_{t+1}=a_{t}+b_{t}$,

where $0<\alpha <1$ is the smoothing factor. $S_{t}$ and $T_{t}$ are the simply smoothed value and doubly smoothed value for the $( t+1 ) $th time period, respectively. Also $a_{t}$ and $b_{t}$ describe the intercept and the slope, respectively.

**Figure 7:**
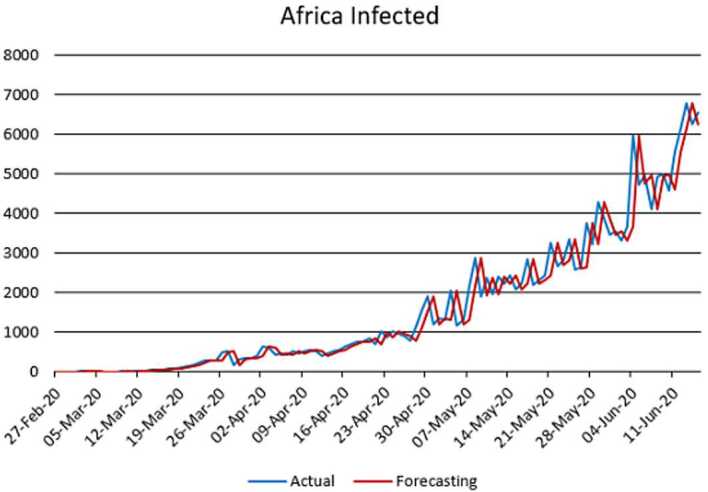
Exponential smoothing for the infected in Africa

**Figure 8:**
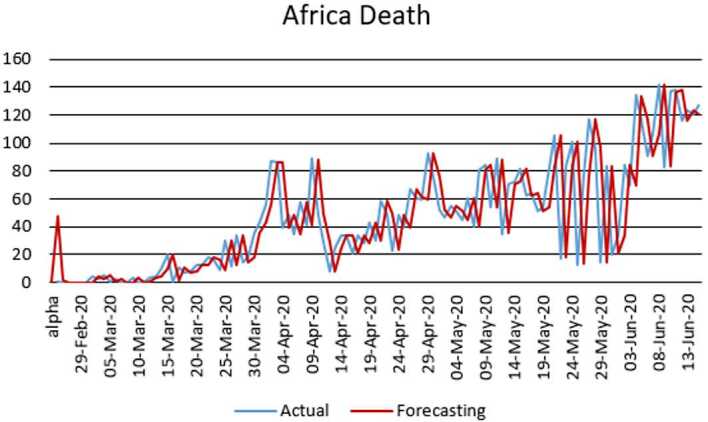
Exponential smoothing for deaths in Africa

**Figure 9:**
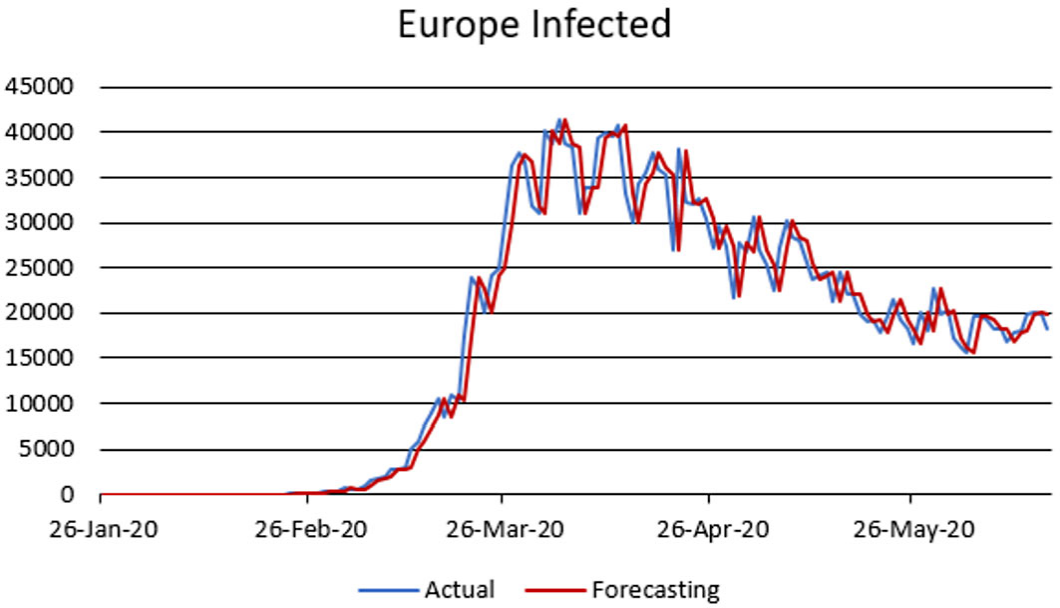
Exponential smoothing for the infected in Europe

**Figure 10:**
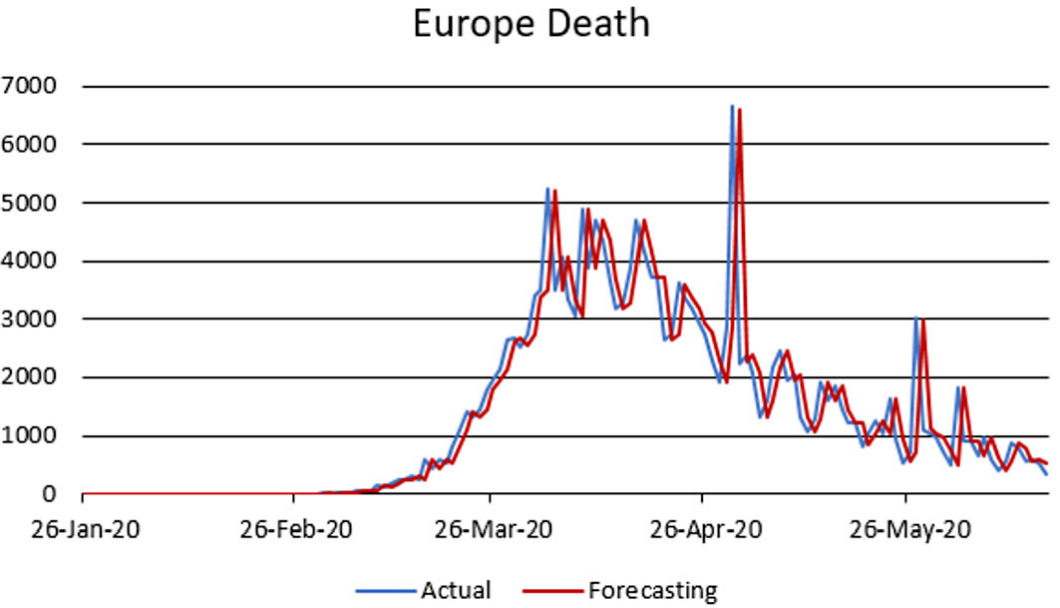
Exponential smoothing for deaths in Europe

In Figs. 7, 8, 9, and 10, we present the simulation for smoothing method for the infected and deaths in Africa and Europe where the smoothing factor was chosen as $\alpha =0.99$.

## Future prediction of daily new numbers of the infected and deaths: Africa and Europe

**Figure 11:**
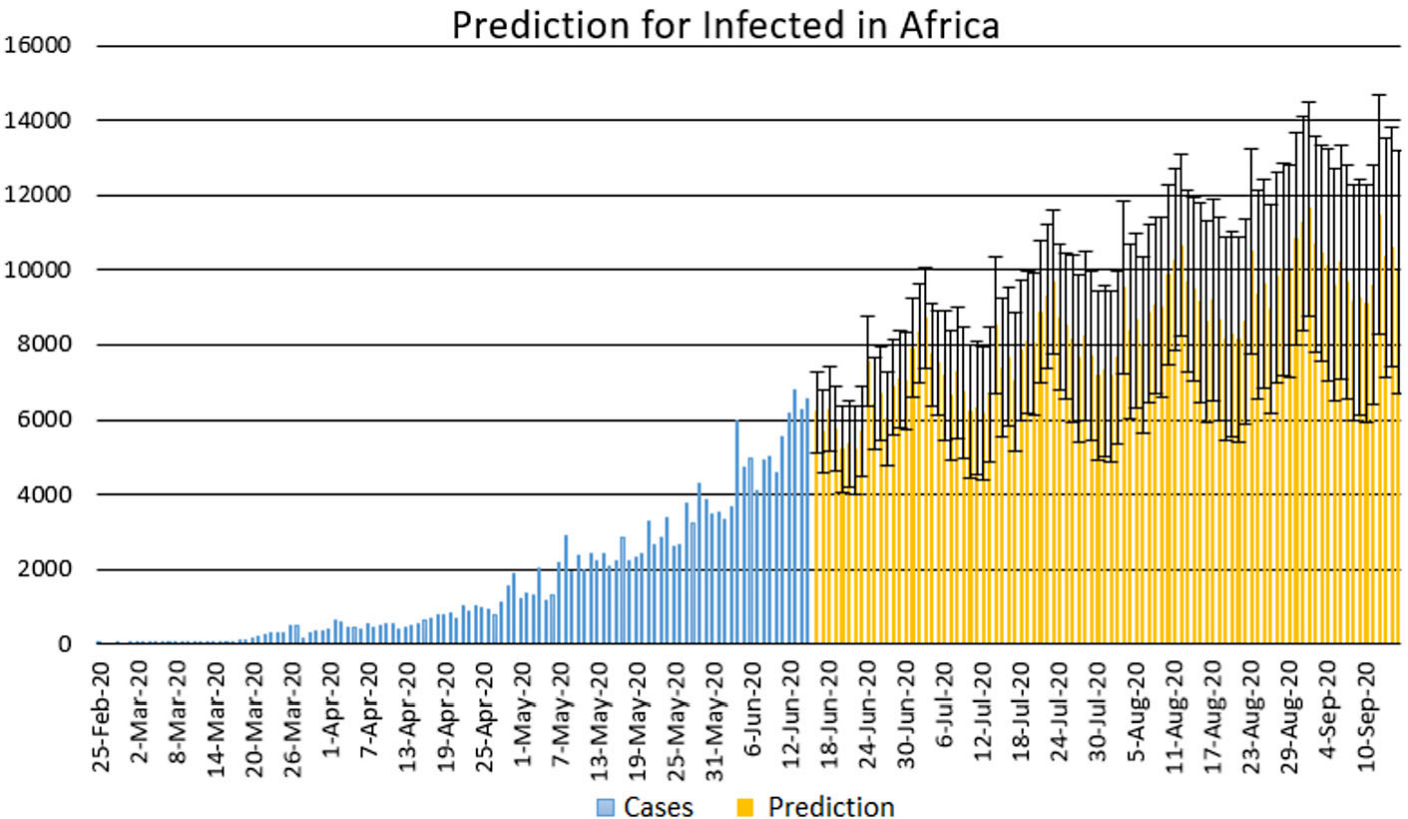
Prediction for the infected in Africa using Forecast Sheet

**Figure 12:**
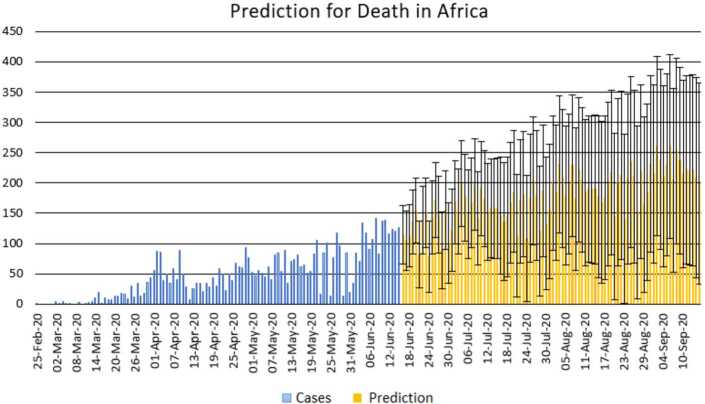
Prediction for deaths in Africa using Forecast Sheet

**Figure 13:**
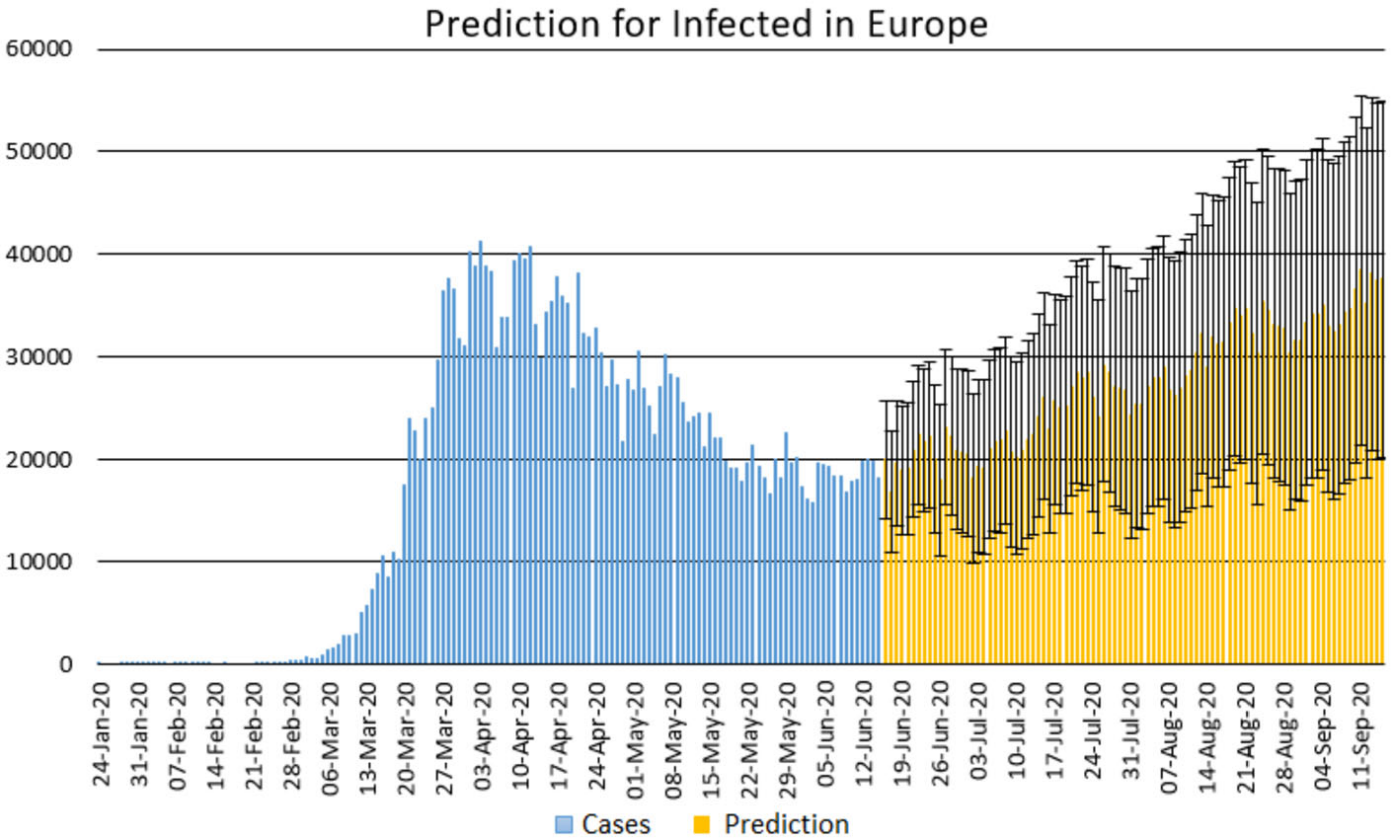
Prediction for the infected in Europe using Forecast Sheet

**Figure 14:**
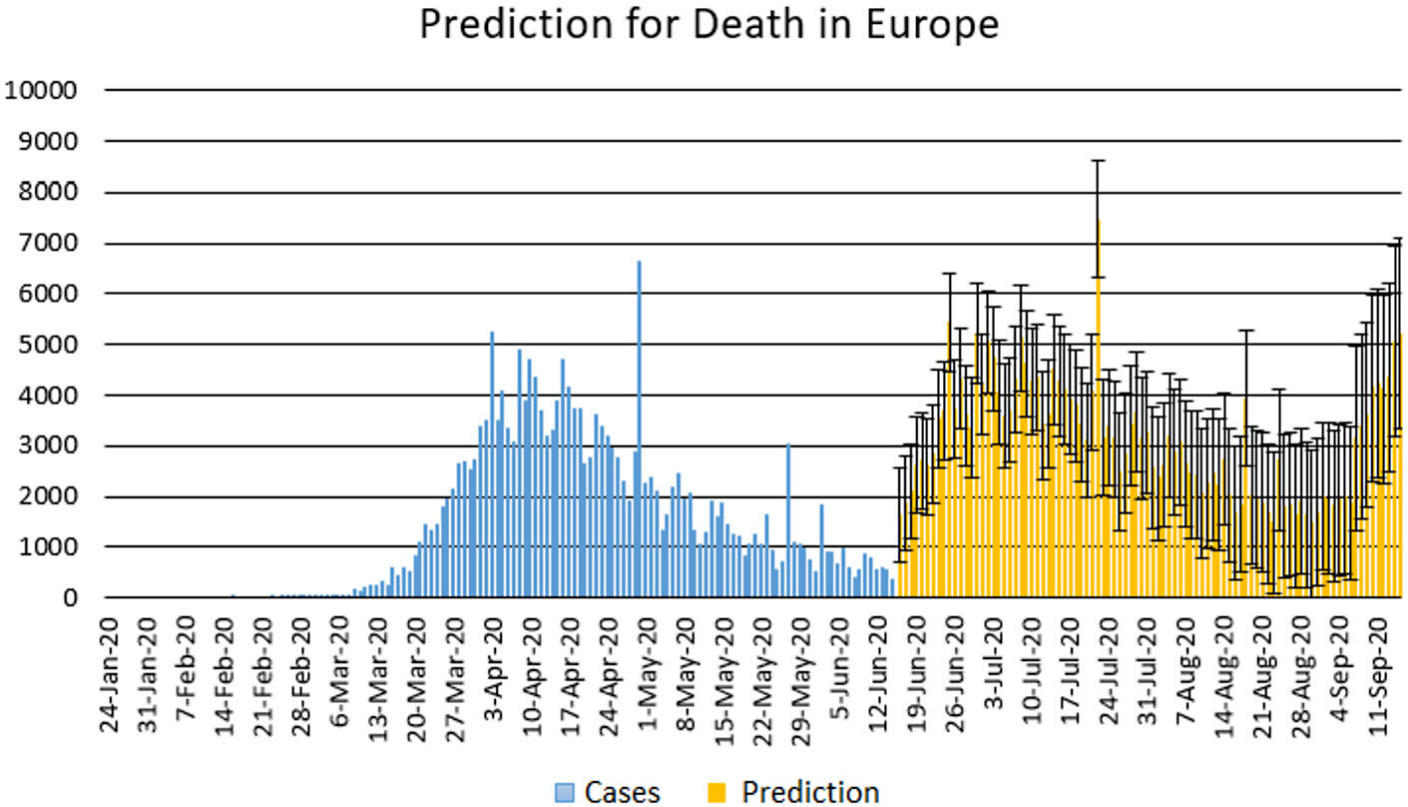
Prediction for deaths in Europe using Forecast Sheet

With the collected data using some statistical formula, it is possible to predict what will possibly happen in the near future. Having in mind what could possibly happen, several measures could be taken to avoid the worst case scenario. In this section, with the data collected for 101 countries from Africa (47) and Europe (54), we aim at presenting possible scenarios or events that could be observed in the near future, the daily numbers of deaths and infections. Numerical simulation are presented in Figs. 11, 12, 13 and 14.

**Figure 15:**
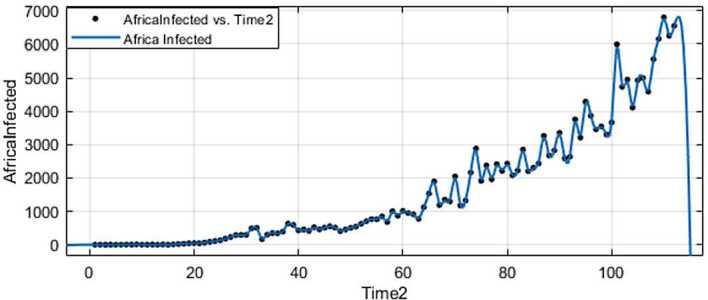
Fitting for the infected in Africa

**Figure 16:**
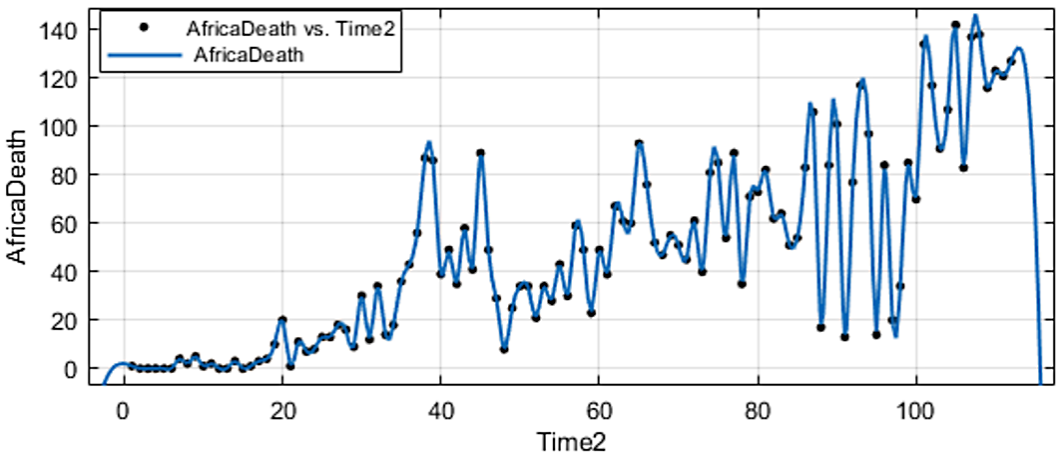
Fitting for deaths in Africa

**Figure 17:**
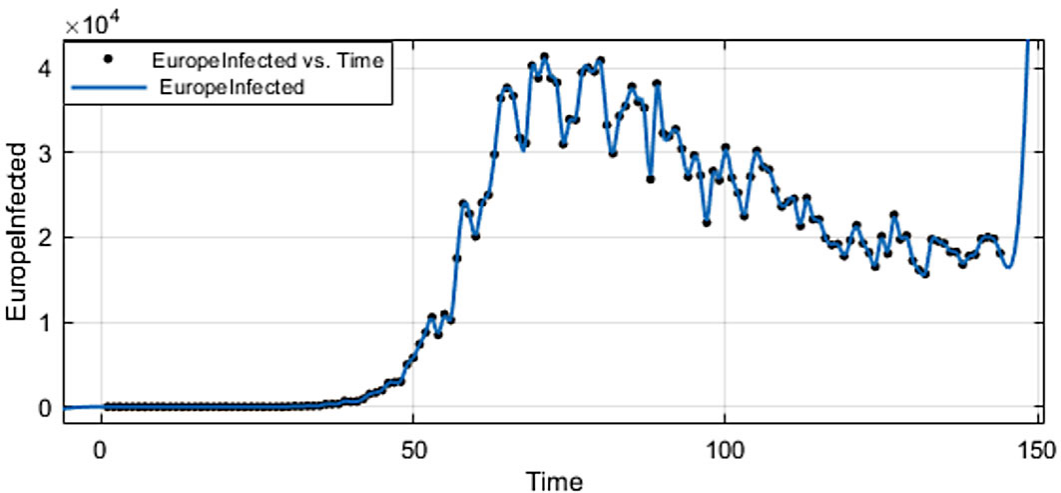
Fitting for the infected in Europe

**Figure 18:**
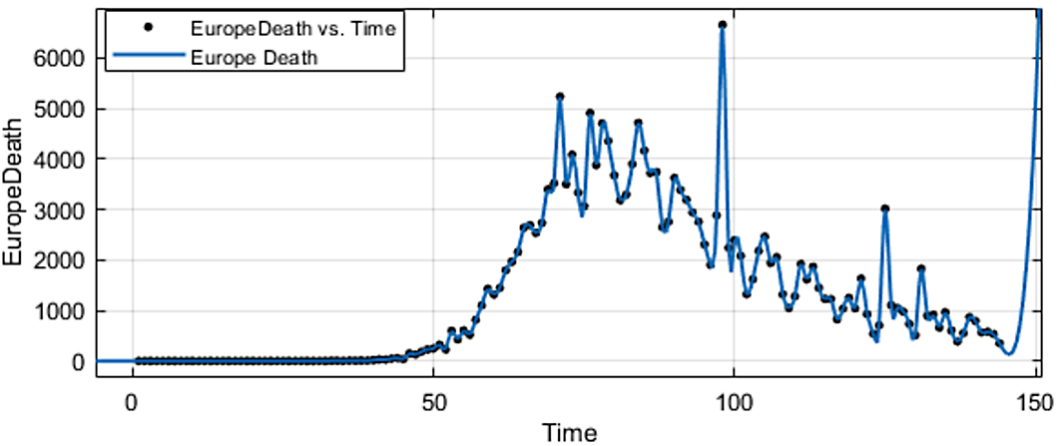
Fitting for deaths in Europe

In Figs. 15, 16, 17, and 18, we present fitting with smoothing spline for the infected and deaths in Africa and Europe.

## An analysis of COVID-19 spread based on fractal interpolation and fractal dimension

In this section, we present some information about fractal dimension, interpolation, and blancmange curve.

### Fractal dimension

Fractal dimensions enable us to compare fractals. Fractal dimensions are important because they can be defined in connection with real-world data, and they can be measured approximately by means of experiments. These numbers allow us to compare sets in the real world with the laboratory fractals.

#### Theorem

(The box counting theorem)

*Let* $N_{n} ( A ) $ *be the number of boxes of side length* $( 1/2^{n} ) $. *Then the fractal dimension* *D* *of* *A* *is given as* [15]

19 $$ D=\lim_{n\rightarrow \infty } \biggl\{ \frac{\ln [ N_{n} ( A ) ] }{\ln ( 2^{n} ) } \biggr\} . $$

### Fractal interpolation

Euclidean geometry and calculus enable us to model using some lines and curves, the shapes that we encounter in the nature [15, 16]. In this section, we present an interpolation function which interpolates the data.

#### Definition 2

An interpolation function $f: [ x_{0},x_{N} ] \rightarrow \mathbb{R} $ corresponding to the set of data $\{ ( x_{i},F_{i} ) \in \mathbb{R} ^{2}:i=0,1,2,\ldots,N \} $ [15]

20 $$ f ( x_{i} ) =F_{i}\quad \text{for }i=1,2,\ldots,N, $$

where $x_{0}< x_{1}< x_{2}\cdots< x_{N}$.

Let $f: [ x_{0},x_{N} ] \rightarrow \mathbb{R} $ denote the unique continuous function which is called a piecewise linear interpolation function. Also this function is linear on each of the subintervals $[ x_{i-1},x_{i} ] $, and it is represented by

21 $$ f ( x ) =F_{i-1}+ \frac{ ( x-x_{i-1} ) }{ ( x_{i}-x_{i-1} ) } ( F_{i}-F_{i-1} )\quad \text{for }x\in [ x_{i-1},x_{i} ] ,i=1,2,\ldots,N. $$

We have the following transformation, which is iterated:

22 $$f_{n}\left(\begin{array}{c} x\\ y \end{array}\right)=\left(\begin{array}{cc} t_{n} & 0\\ u_{n} & y_{n} \end{array}\right)\left(\begin{array}{c} x\\ y \end{array}\right)+\left(\begin{array}{c} v_{n}\\ w_{n} \end{array}\right).$$

When solving this system for $t_{n}$, $u_{n}$, $v_{n}$, and $w_{n}$ in terms of the data and $y_{n}$, we obtain the following:

23 $$\begin{aligned}& t_{n} =\frac{x_{n}-x_{n-1}}{x_{N}-x_{0}}, \\& u_{n} =\frac{F_{n}-F_{n-1}}{x_{N}-x_{0}}-y_{n} \frac{F_{n}-F_{0}}{x_{N}-x_{0}}, \\& v_{n} =\frac{x_{N}x_{n-1}-x_{0}x_{n}}{x_{N}-x_{0}}, \\& w_{n} =\frac{x_{N}F_{n-1}-x_{0}F_{n}}{x_{N}-x_{0}}-y_{n} \frac{x_{N}F_{0}-x_{0}F_{n}}{x_{N}-x_{0}}, \end{aligned}$$

where $0\leq y_{n}<1$ is called the scaling factor [15].

### Blancmange curve

The blancmange function can be given as an example of fractal interpolation function, and this function is defined by

24 $$ \sum_{n=0}^{\infty }\frac{S ( 2^{n}x ) }{2^{n}},\quad x \in [ 0,1 ], $$

where $S ( x ) =\min_{m\in \mathbb{Z} } \vert x-m \vert $, $x\in \mathbb{R} $.

However, many problems cannot be depicted when $c=2$ [16]. Then we discuss the limitations of this blancmange; for example, *t* can only go from 0 to 1, the periodic parameter is 2. Therefore, we change 2 to *c*, where *c* is a real number from 1 to *a*. Therefore, in this section, we extend the blancmange function to a large interval also with any given periodic parameter. So, we have the following formula:

25 $$ \sum_{n=0}^{\infty }\frac{S ( c^{n}x ) }{c^{n}},\quad x \in [ 0,a ], $$

where *c* is the real number. We now present the extended blancmange function for different periodic parameters and different *w*.

**Figure 19:**
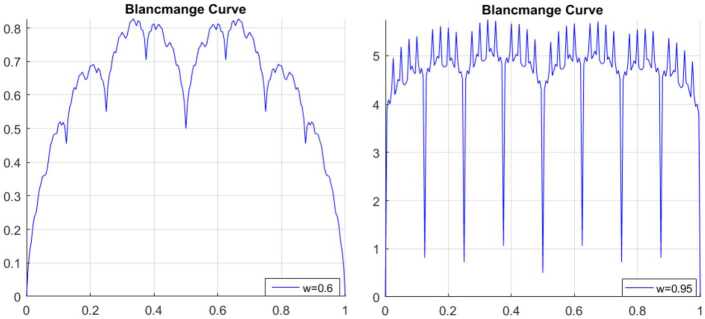
Blancmange function $c = 2$

**Figure 20:**
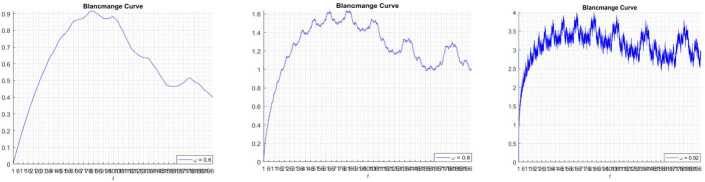
Blancmange function $c = 3.7$

**Figure 21:**
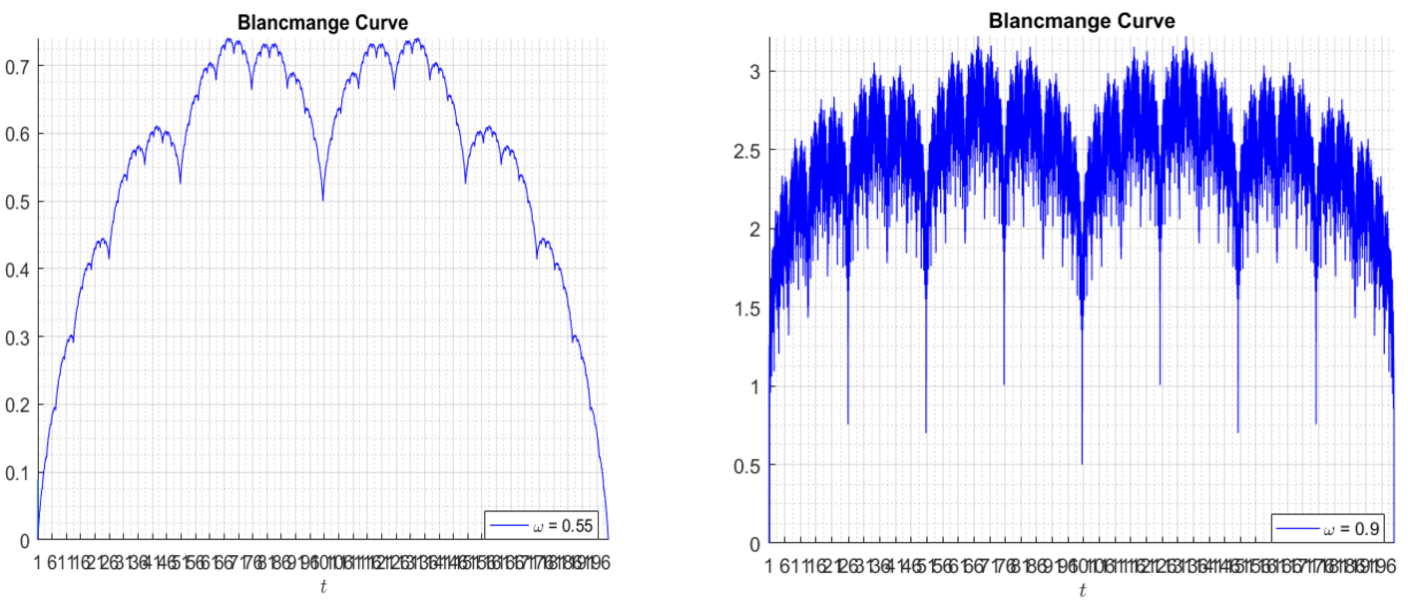
Blancmange function $c = 1.3$

The simulation are presented in Figs. 19, 20, 21, and 18.

## Mathematical model for COVID-19 outbreak

We consider the following mathematical model of COVID-19 spread:

26 $$\begin{aligned}& \overset{\cdot }{S } =\Lambda - \bigl\{ \delta ( t ) \bigl( \alpha I+w ( \beta I_{D}+\gamma I_{A}+\delta _{1}I_{R}+ \delta _{2}I_{T} ) +\gamma _{1}+\mu _{1} \bigr) \bigr\} S, \\& \overset{\cdot }{I} =\delta ( t ) \bigl( \alpha I+w ( \beta I_{D}+ \gamma I_{A}+\delta _{1}I_{R}+\delta _{2}I_{T} ) \bigr) S- ( \varepsilon +\xi +\lambda +\mu _{1} ) I, \\& \overset{\cdot }{I_{A}} =\xi I- ( \theta +\mu +\chi + \mu _{1} ) I_{A}, \\& \overset{\cdot }{I_{D} } =\varepsilon I- ( \eta + \varphi +\mu _{1} ) I_{D}, \\& \overset{\cdot }{I_{R} } =\eta I_{D}+\theta I_{A}- ( v+ \xi +\mu _{1} ) I_{R}, \\& \overset{\cdot }{I_{T}} =\mu I_{A}+vI_{R}- ( \sigma + \tau +\mu _{1} ) I_{T}, \\& \overset{\cdot }{R} = \lambda I+\varphi I_{D}+\chi I_{A}+ \xi I_{R}+\sigma I_{T}- ( \Phi +\mu _{1} ) R, \\& \overset{\cdot }{D} =\tau I_{T}, \\& \overset{\cdot }{V} =\gamma _{1}S+\Phi R-\mu _{1}V. \end{aligned}$$

The above model was suggested by Atangana and Seda, the model has a deterministic character. In this section, we convert the model to a stochastic one by introducing the effect of environmental white noise. To achieve this, we reformulate the model by adding the nonlinear perturbation into each equation of the system. The perturbation may depend on square of the classes *S*, *I*, $I_{A}$, $I_{D}$, $I_{R}$, $I_{T}$, *R*, *D*, and *V* respectively. Here, we perturb only the rate of each class. However, for the vaccine class, it will be perturbed by a natural death rate.

$$\begin{aligned}& \text{For the class }S ( t ) : \quad -\gamma _{1} \rightarrow -\gamma _{1}+ ( \Pi _{11}S+\Pi _{12} ) \overset{\cdot }{B}_{1} ( t ) , \\& \text{For the class }I ( t ) : \quad -\lambda \rightarrow -\lambda + ( \Pi _{21}I+\Pi _{22} )\overset{\cdot }{B}_{2} ( t ) , \\& \text{For the class }I_{A} ( t ) :\quad -\theta \rightarrow -\theta + ( \Pi _{31}I_{A}+\Pi _{32} ) \overset{\cdot }{B}_{3} ( t ) , \\& \text{For the class }I_{D} ( t ) : \quad -\eta \rightarrow -\eta + ( \Pi _{41}I_{D}+\Pi _{42} )\overset{\cdot }{B}_{4} ( t ), \\& \text{For the class }I_{R} ( t ) : \quad -v\rightarrow -v+ ( \Pi _{51}I_{R}+\Pi _{52} )\overset{\cdot }{B}_{5} ( t ), \\& \text{For the class }I_{T} ( t ) :\quad -\sigma \rightarrow -\sigma + ( \Pi _{61}I_{T}+\Pi _{62} )\overset{\cdot }{B}_{6} ( t ), \\& \text{For the class }R ( t ) : \quad -\Phi \rightarrow - \Phi + ( \Pi _{71}R+\Pi _{72} )\overset{\cdot }{B}_{7} ( t ), \\& \text{For the class }D ( t ) : \quad \tau \rightarrow \tau \text{ no change}, \\& \text{For the class }V ( t ) : \quad -\mu _{1} \rightarrow -\mu _{1}+ ( \Pi _{81}V+\Pi _{82} )\overset{\cdot }{B}_{8} ( t ) . \end{aligned}$$

Therefore, the associated stochastic model is given as follows:

27 $$\begin{aligned}& \begin{aligned} dS = {}& \bigl[ \Lambda - \bigl\{ \delta ( t ) \bigl( \alpha I+w ( \beta I_{D}+\gamma I_{A}+\delta _{1}I_{R}+ \delta _{2}I_{T} ) +\gamma _{1}+\mu _{1} \bigr) \bigr\} S \bigr]\,dt \\ &{}+ ( \Pi _{11}S+\Pi _{12} ) S\,dB_{1} ( t ), \end{aligned} \\& \begin{aligned} dI = {}& \bigl[ \delta ( t ) \bigl( \alpha I+w ( \beta I_{D}+\gamma I_{A}+\delta _{1}I_{R}+ \delta _{2}I_{T} ) \bigr) S- ( \varepsilon +\xi +\lambda + \mu _{1} ) I \bigr]\,dt \\ &{}+ ( \Pi _{21}I+\Pi _{22} ) I\,dB_{2} ( t ), \end{aligned} \\& dI_{A} = \bigl[ \xi I- ( \theta +\mu +\chi +\mu _{1} ) I_{A} \bigr]\,dt+ ( \Pi _{31}I_{A}+\Pi _{32} ) I_{A}\,dB_{3} ( t ), \\& dI_{D} = \bigl[ \varepsilon I- ( \eta +\varphi +\mu _{1} ) I_{D} \bigr]\,dt+ ( \Pi _{41}I_{D}+ \Pi _{42} ) I_{D}\,dB_{4} ( t ), \\& dI_{R} = \bigl[ \eta I_{D}+\theta I_{A}- ( v+\xi +\mu _{1} ) I_{R} \bigr]\,dt+ ( \Pi _{51}I_{R}+\Pi _{52} ) I_{D} \,dB_{5} ( t ), \\& dI_{T} = \bigl[ \mu I_{A}+vI_{R}- ( \sigma + \tau +\mu _{1} ) I_{T} \bigr]\,dt+ ( \Pi _{61}I_{T}+\Pi _{62} ) I_{T} \,dB_{6} ( t ), \\& dR = \bigl[ \lambda I+\varphi I_{D}+\chi I_{A}+\xi I_{R}+\sigma I_{T}- ( \Phi +\mu _{1} ) R \bigr]\,dt+ ( \Pi _{71}R+\Pi _{72} ) R\,dB_{7} ( t ), \\& dV = [ \gamma _{1}S+\Phi R-\mu _{1}V ]\,dt++ ( \Pi _{71}V+\Pi _{72} ) V\,dB_{8} ( t ) . \end{aligned}$$

In this conversion, the function $B_{i} ( t ) $ represents the standard Brownian motions valid within the set of probability $( \Omega ,A, \{ A_{t} \} _{t\geq 0},P ) $, where $\{ A_{t} \} _{t\geq 0}$ is filtration valid under the condition described in [17]. Here, $\Pi _{i,j\in [ 1,2,3,4,5,6,7,8 ] }$ are positive and are the intensities of the environmental random disturbance.

### Existence and uniqueness

In this subsection, we present the existence and uniqueness of the system solutions of the stochastic model. To achieve the existence and uniqueness, we convert the system into Volterra type. But first we do the following for simplicity:

28 $$\begin{aligned}& dS = F_{1} ( t,S,I,I_{A},I_{D},I_{R},I_{T},R,V )\,dt+G_{1} ( t,S )\,dB_{1} ( t ), \\& dI = F_{2} ( t,S,I,I_{A},I_{D},I_{R},I_{T},R,V )\,dt+G_{2} ( t,I )\,dB_{2} ( t ), \\& dI_{A} = F_{3} ( t,I,I_{A} ) \,dt+G_{3} ( t,I_{A} )\,dB_{3} ( t ), \\& dI_{D} = F_{4} ( t,I,I_{D}, ) \,dt+G_{4} ( t,I_{D} )\,dB_{4} ( t ), \\& dI_{R} = F_{5} ( t,I_{A},I_{D},I_{R} )\,dt+G_{5} ( t,I_{R} )\,dB_{5} ( t ), \\& dI_{T} = F_{6} ( t,I_{A},I_{R},I_{T} )\,dt+G_{6} ( t,I_{T} )\,dB_{6} ( t ), \\& dR = F_{7} ( t,I,I_{A},I_{D},I_{R},I_{T},R )\,dt+G_{7} ( t,R )\,dB_{7} ( t ), \\& dV = F_{8} ( t,S,R,V )\,dt+G_{8} ( t,V ) \,dB_{8} ( t ) . \end{aligned}$$

Therefore, converting to Volterra, we get

29 $$\begin{aligned}& S ( t ) = S ( 0 ) + \int _{0}^{t}F_{1} ( \tau ,S,I,I_{A},I_{D},I_{R},I_{T},R,V )\,d\tau + \int _{0}^{t}G_{1} ( \tau ,S ) \,dB_{1} ( \tau ), \\& I ( t ) = I ( 0 ) + \int _{0}^{t}F_{2} ( \tau ,S,I,I_{A},I_{D},I_{R},I_{T},R,V )\,d\tau + \int _{0}^{t}G_{2} ( \tau ,I ) \,dB_{2} ( \tau ), \\& I_{A} ( t ) = I_{A} ( 0 ) + \int _{0}^{t}F_{3} ( \tau ,I,I_{A} )\,d\tau + \int _{0}^{t}G_{3} ( \tau ,I_{A} )\,dB_{3} ( \tau ), \\& I_{D} ( t ) = I_{D} ( 0 ) + \int _{0}^{t}F_{4} ( \tau ,I,I_{D} )\,d\tau + \int _{0}^{t}G_{4} ( \tau ,I_{D} )\,dB_{4} ( \tau ), \\& I_{R} ( t ) = I_{R} ( 0 ) + \int _{0}^{t}F_{5} ( \tau ,I_{A},I_{D},I_{R} )\,d\tau + \int _{0}^{t}G_{5} ( \tau ,I_{R} )\,dB_{5} ( \tau ), \\& I_{T} ( t ) = I_{T} ( 0 ) + \int _{0}^{t}F_{6} ( \tau ,I_{A},I_{R},I_{T} )\,d\tau + \int _{0}^{t}G_{6} ( \tau ,I_{T} )\,dB_{6} ( \tau ), \\& R ( t ) = R ( 0 ) + \int _{0}^{t}F_{7} ( \tau ,I,I_{A},I_{D},I_{R},I_{T},R ) \,d \tau + \int _{0}^{t}G_{7} ( \tau ,R ) \,dB_{7} ( \tau ), \\& V ( t ) = V ( 0 ) + \int _{0}^{t}F_{8} ( \tau ,S,R,V )\,d\tau + \int _{0}^{t}G_{8} ( \tau ,S ) \,dB_{8} ( \tau ) . \end{aligned}$$

We present the existence and uniqueness of the stochastic system of COVID-19 model. This will be achieved via the following theorem.

#### Theorem

*Assume that there exist positive constants* $K_{i}$, $\overline{K}_{i}$ *such that*

(i) 30 $$\begin{aligned}& \bigl\vert F_{i} ( x,t ) -F_{i} ( x_{i},t ) \bigr\vert ^{2} < K_{i} \vert x-x_{i} \vert ^{2}, \\& \bigl\vert G_{i} ( x,t ) -G_{i} ( x_{i},t ) \bigr\vert ^{2} < \overline{K}_{i} \vert x-x_{i} \vert ^{2} \end{aligned}$$
(ii) $\forall ( x,t ) \in R^{8}\times [ 0,T ] $
  31 $$ \bigl\vert F_{i} ( x,t ) \bigr\vert ^{2}, \bigl\vert G_{i} ( x,t ) \bigr\vert ^{2}< K \bigl( 1+ \vert x \vert ^{2} \bigr) . $$

*Then there exists a unique solution* $X ( t ) \in R^{8}$ *for our model and it belongs to* $M^{2} ( [ 0,T ] , R^{8} ) $.

The proof can be found in [17], but we have to verify (i) and (ii) for our system. Without loss of generality, we start our investigation with functions $F_{1} ( t,S,I,I_{A},I_{D},I_{R},I_{T},R,V ) $ and $G_{1} ( t,S ) $. For the function *F*, the proof will be performed for $( t,S ) $. Thus

32 $$ \bigl\vert F_{1} ( t,S ) -F_{1} ( t,S_{1} ) \bigr\vert ^{2}= \bigl\vert \delta ( t ) \bigl( \alpha I+w ( \beta I_{D}+\gamma I_{A}+\delta _{1}I_{R}+ \delta _{2}I_{T} ) +\gamma _{1}+\mu _{1} \bigr) ( S-S_{1} ) \bigr\vert ^{2}. $$

We define the following norm:

33 $$ \Vert \varphi \Vert _{\infty }=\sup_{t\in [ 0,T ] } \vert \varphi \vert ^{2}, $$

then

34 $$\begin{aligned} \bigl\vert F_{1} ( S,t ) -F_{1} ( S_{1},t ) \bigr\vert ^{2} \leq &\sup_{t\in [ 0,T ] } \bigl\vert \delta ( t ) \bigl( \alpha I+w ( \beta I_{D}+ \gamma I_{A}+\delta _{1}I_{R}+\delta _{2}I_{T} ) \bigr) ( S-S_{1} ) \bigr\vert ^{2} \\ \leq & \bigl\Vert \delta ( t ) \bigl( \alpha I+w ( \beta I_{D}+ \gamma I_{A}+\delta _{1}I_{R}+\delta _{2}I_{T} ) \bigr) \bigr\Vert _{\infty }^{2} \vert S-S_{1} \vert ^{2} \\ \leq &K_{1} \vert S-S_{1} \vert ^{2} \end{aligned}$$

and

35 $$\begin{array}{rcl} |G_{1}(S,t)-G_{1}(S_{1},t){|}^{2} & = & |(\Pi_{11}S+\Pi_{12})S-(\Pi_{11}S_{1}+\Pi_{12})S_{1}{|}^{2}\\ & = & |\Pi_{11}\left(S^{2}-S_{1}^{2}\right)-\Pi_{12}(S-S_{1}){|}^{2}\\ & = & {\left(\Pi_{11}(S+S_{1})+\Pi_{12}\right)}^{2}|S-S_{1}{|}^{2}\\ & = & \left(\Pi_{11}^{2}{\left(S+S_{1}\right)}^{2}+2\Pi_{11}\Pi_{12}(S+S_{1})+\Pi_{12}^{2}\right)|S-S_{1}{|}^{2}\\ & = & \left(\Pi_{11}^{2}\left(S^{2}+2SS_{1}+S_{1}^{2}\right)+2\Pi_{11}\Pi_{12}(S+S_{1})+\Pi_{12}^{2}\right)|S-S_{1}{|}^{2}\\ & \leq & \left\{\begin{array}{c} \Pi_{11}^{2}\left(\begin{array}{c} \sup_{t\in [0,T]}|S^{2}(t)|+2\sup_{t\in [0,T]}|S(t)|\sup_{t\in [0,T]}|S_{1}(t)|\\ +\sup_{t\in [0,T]}|S_{1}^{2}(t)| \end{array}\right)\\ +2\Pi_{11}\Pi_{12}\left(\sup_{t\in [0,T]}|S(t)|+\sup_{t\in [0,T]}|S_{1}(t)|\right)+\Pi_{12}^{2} \end{array}\right\}\\ & & \times |S-S_{1}{|}^{2}\\ & \leq & \left\{\begin{array}{c} \Pi_{11}^{2}({∥S^{2}∥}_{\infty }+2{∥S∥}_{\infty }{∥S_{1}∥}_{\infty }+{∥S_{1}^{2}∥}_{\infty })\\ +2\Pi_{11}\Pi_{12}{∥S∥}_{\infty }{∥S_{1}∥}_{\infty }+\Pi_{12}^{2} \end{array}\right\}|S-S_{1}{|}^{2}\\ & \leq & {\bar{K}}_{1}|S-S_{1}{|}^{2}, \end{array}$$

where

36 $$\begin{aligned} \overline{K}_{1} =&\Pi _{11}^{2} \bigl( \bigl\Vert S^{2} \bigr\Vert _{\infty }+2 \Vert S \Vert _{\infty } \Vert S_{1} \Vert _{\infty }+ \bigl\Vert S_{1}^{2} \bigr\Vert _{\infty } \bigr) +2\Pi _{11}\Pi _{12} \Vert S \Vert _{\infty } \Vert S_{1} \Vert _{\infty }+\Pi _{12}^{2} \\ =&\Pi _{11}^{2} \bigl( \Vert S \Vert _{\infty }+ \Vert S_{1} \Vert _{\infty } \bigr) ^{2}+2\Pi _{11}\Pi _{12} \Vert S \Vert _{\infty } \Vert S_{1} \Vert _{ \infty }+\Pi _{12}^{2}. \end{aligned}$$

Similarly,

37 $$\begin{aligned}& \overline{K}_{2} = \Pi _{21}^{2} \bigl( \Vert I \Vert _{ \infty }+ \Vert I_{1} \Vert _{\infty } \bigr) ^{2}+2\Pi _{21} \Pi _{22} \Vert I \Vert _{\infty } \Vert I_{1} \Vert _{\infty }+\Pi _{22}^{2}, \\ & \overline{K}_{3} = \Pi _{31}^{2} \bigl( \Vert I_{A} \Vert _{\infty }+ \Vert I_{A1} \Vert _{\infty } \bigr) ^{2}+2 \Pi _{31}\Pi _{32} \Vert I_{A} \Vert _{\infty } \Vert I_{A1} \Vert _{\infty }+\Pi _{32}^{2}, \\ & \overline{K}_{4} = \Pi _{41}^{2} \bigl( \Vert I_{D} \Vert _{\infty }+ \Vert I_{D1} \Vert _{\infty } \bigr) ^{2}+2 \Pi _{41}\Pi _{42} \Vert I_{D} \Vert _{\infty } \Vert I_{D1} \Vert _{\infty }+\Pi _{42}^{2}, \\ & \overline{K}_{5} = \Pi _{51}^{2} \bigl( \Vert I_{R} \Vert _{\infty }+ \Vert I_{R1} \Vert _{\infty } \bigr) ^{2}+2 \Pi _{51}\Pi _{52} \Vert I_{R} \Vert _{\infty } \Vert I_{R1} \Vert _{\infty }+\Pi _{52}^{2}, \\ & \overline{K}_{6} = \Pi _{61}^{2} \bigl( \Vert I_{T} \Vert _{\infty }+ \Vert I_{T1} \Vert _{\infty } \bigr) ^{2}+2 \Pi _{61}\Pi _{62} \Vert I_{T} \Vert _{\infty } \Vert I_{T1} \Vert _{\infty }+\Pi _{62}^{2}, \\ & \overline{K}_{7} = \Pi _{71}^{2} \bigl( \Vert R \Vert _{ \infty }+ \Vert R_{1} \Vert _{\infty } \bigr) ^{2}+2\Pi _{71} \Pi _{72} \Vert R \Vert _{\infty } \Vert R_{1} \Vert _{\infty }+\Pi _{72}^{2}, \\ & \overline{K}_{8} = \Pi _{81}^{2} \bigl( \Vert V \Vert _{ \infty }+ \Vert V_{1} \Vert _{\infty } \bigr) ^{2}+2\Pi _{81} \Pi _{82} \Vert V \Vert _{\infty } \Vert V_{1} \Vert _{\infty }+\Pi _{82}^{2}. \end{aligned}$$

Also

38 $$\begin{aligned} \bigl\vert F_{2} ( I,t ) -F_{2} ( I_{1},t ) \bigr\vert ^{2} =& \bigl\vert \delta ( t ) \alpha ( I-I_{1} ) - ( \varepsilon +\xi +\lambda +\mu _{1} ) ( I-I_{1} ) \bigr\vert ^{2} \\ =& \bigl\vert \bigl( \delta ( t ) \alpha - ( \varepsilon +\xi +\lambda +\mu _{1} ) \bigr) ( I-I_{1} ) \bigr\vert ^{2} \\ \leq &\sup_{t\in [ 0,T ] } \bigl\vert \bigl( \delta ( t ) \alpha - ( \varepsilon +\xi +\lambda +\mu _{1} ) \bigr) \bigr\vert ^{2} \vert I-I_{1} \vert ^{2} \\ \leq & \bigl\Vert \delta ( t ) \bigr\Vert _{ \infty } \big\vert \alpha - ( \varepsilon +\xi +\lambda +\mu _{1} ) \bigr\vert ^{2} \vert I-I_{1} \vert ^{2} \\ \leq &K_{2} \vert I-I_{1} \vert ^{2}, \end{aligned}$$

where

39 $$ K_{2}= \bigl\Vert \delta ( t ) \bigr\Vert _{ \infty } \big\vert \alpha - ( \varepsilon +\xi +\lambda +\mu _{1} ) \bigr\vert ^{2}. $$

Also

40 $$\begin{aligned} \bigl\vert F_{3} ( I_{A},t ) -F_{3} ( I_{A1},t ) \bigr\vert ^{2} =& \bigl\vert - ( \theta + \mu +\chi + \mu _{1} ) ( I_{A}-I_{A1} ) \bigr\vert ^{2} \\ \leq &2 \bigl\vert ( \theta +\mu +\chi +\mu _{1} ) \bigr\vert ^{2} \vert I_{A}-I_{A1} \vert ^{2} \\ \leq &K_{3} \vert I_{A}-I_{A1} \vert ^{2}, \end{aligned}$$

where

41 $$ K_{3}=2 \bigl\vert ( \theta +\mu +\chi +\mu _{1} ) \bigr\vert ^{2}. $$

Similarly, we evaluate

42 $$\begin{aligned}& \begin{aligned}[t] \bigl\vert F_{4} ( I_{D},t ) -F_{4} ( I_{D1},t ) \bigr\vert ^{2} ={}& \vert \eta +\varphi +\mu _{1} \vert ^{2} \vert I_{D}-I_{D1} \vert ^{2} \\ \leq {}&K_{4} \vert I_{D}-I_{D1} \vert ^{2}, \end{aligned} \\& \begin{aligned} \bigl\vert F_{5} ( I_{R},t ) -F_{5} ( I_{R1},t ) \bigr\vert ^{2} &= \vert v+\xi +\mu _{1} \vert ^{2} \vert I_{R}-I_{R1} \vert ^{2} \\ &\leq K_{5} \vert I_{R}-I_{R1} \vert ^{2}, \end{aligned} \\& \begin{aligned} \bigl\vert F_{6} ( I_{T},t ) -F_{6} ( I_{T1},t ) \bigr\vert ^{2} &= \vert \sigma +\tau +\mu _{1} \vert ^{2} \vert I_{T}-I_{T1} \vert ^{2} \\ &\leq K_{6} \vert I_{T}-I_{T1} \vert , \end{aligned} \\& \begin{aligned} \bigl\vert F_{7} ( R,t ) -F_{7} ( R_{1},t ) \bigr\vert ^{2} &= \vert \Phi +\mu _{1} \vert ^{2} \vert R-R_{1} \vert ^{2} \\ &\leq K_{7} \vert R-R_{1} \vert , \end{aligned} \\& \begin{aligned} \bigl\vert F_{8} ( V,t ) -F_{8} ( V_{1},t ) \bigr\vert ^{2} &= \vert \mu _{1} \vert ^{2} \vert V-V_{1} \vert ^{2} \\ &\leq K_{8} \vert V-V_{1} \vert ^{2}. \end{aligned} \end{aligned}$$

For both classes $G_{i}$ and $F_{i}$, we have verified condition (i). Now we verify the second condition.

43 $$\begin{aligned} \bigl\vert F_{1} ( S,t ) \bigr\vert ^{2} =& \bigl\vert \Lambda -\delta ( t ) \bigl( \alpha I+w ( \beta I_{D}+\gamma I_{A}+\delta _{1}I_{R}+\delta _{2}I_{T} ) + \gamma _{1}+\mu _{1} \bigr) S \bigr\vert ^{2} \\ \leq & \bigl\vert \Lambda S-\delta ( t ) \bigl( \alpha I+w ( \beta I_{D}+\gamma I_{A}+\delta _{1}I_{R}+ \delta _{2}I_{T} ) +\gamma _{1}+\mu _{1} \bigr) S \bigr\vert ^{2} \\ \leq & \vert S \vert ^{2} \bigl\vert \Lambda -\delta ( t ) \bigl( \alpha I+w ( \beta I_{D}+\gamma I_{A}+ \delta _{1}I_{R}+\delta _{2}I_{T} ) + \gamma _{1}+\mu _{1} \bigr) \bigr\vert ^{2} \\ < & \bigl( \vert S \vert ^{2}+1 \bigr) \bigl\vert \Lambda - \delta ( t ) \bigl( \alpha I+w ( \beta I_{D}+ \gamma I_{A}+\delta _{1}I_{R}+\delta _{2}I_{T} ) +\gamma _{1}+ \mu _{1} \bigr) \bigr\vert ^{2} \\ < & \bigl( \vert S \vert ^{2}+1 \bigr) \bigl\vert \Lambda - \delta ( t ) \bigl( \alpha I+w ( \beta I_{D}+ \gamma I_{A}+\delta _{1}I_{R}+\delta _{2}I_{T} ) +\gamma _{1}+ \mu _{1} \bigr) \bigr\vert ^{2} \\ < & \bigl( \vert S \vert ^{2}+1 \bigr) \sup_{t\in [ 0,T ] } \bigl\vert \Lambda -\delta ( t ) \bigl( \alpha I+w ( \beta I_{D}+ \gamma I_{A}+\delta _{1}I_{R}+ \delta _{2}I_{T} ) +\gamma _{1}+\mu _{1} \bigr) \bigr\vert ^{2} \\ < &K^{1} \bigl( \vert S \vert ^{2}+1 \bigr), \end{aligned}$$

where

44 $$ K^{1}=\sup_{t\in [ 0,T ] } \bigl\vert \Lambda -\delta ( t ) \bigl( \alpha I+w ( \beta I_{D}+\gamma I_{A}+ \delta _{1}I_{R}+\delta _{2}I_{T} ) + \gamma _{1}+\mu _{1} \bigr) \bigr\vert ^{2}. $$

Then

45 $$\begin{aligned} \bigl\vert G_{1} ( S,t ) -G_{1} ( S_{1},t ) \bigr\vert ^{2} =& \bigl\vert ( \Pi _{11}S+\Pi _{12} ) S \bigr\vert ^{2} \\ \leq & \bigl\vert \Pi _{11}S^{2}+\Pi _{12}S^{2} \bigr\vert ^{2} \\ \leq & ( \Pi _{11}+\Pi _{12} ) ^{2} \bigl\vert S^{2} \bigr\vert ^{2} \\ \leq & ( \Pi _{11}+\Pi _{12} ) ^{2}\sup _{t\in [ 0,T ] } \bigl\vert S^{2} \bigr\vert \vert S \vert ^{2} \\ \leq & ( \Pi _{11}+\Pi _{12} ) ^{2} \bigl\Vert S^{2} \bigr\Vert _{\infty } \bigl( \vert S \vert ^{2}+1 \bigr) \\ \leq &\overline{K}^{1} \bigl( \vert S \vert ^{2}+1 \bigr), \end{aligned}$$

where

46 $$ \overline{K}^{1}= ( \Pi _{11}+\Pi _{12} ) ^{2} \bigl\Vert S^{2} \bigr\Vert _{\infty }. $$

Similarly,

47 $$\begin{aligned}& \overline{K}^{2} = ( \Pi _{21}+\Pi _{22} ) ^{2} \bigl\Vert I^{2} \bigr\Vert _{\infty }, \\& \overline{K}^{3} = ( \Pi _{31}+\Pi _{32} ) ^{2} \bigl\Vert I_{A}^{2} \bigr\Vert _{\infty }, \\& \overline{K}^{4} = ( \Pi _{41}+\Pi _{42} ) ^{2} \bigl\Vert I_{D}^{2} \bigr\Vert _{\infty }, \\& \overline{K}^{5} = ( \Pi _{51}+\Pi _{52} ) ^{2} \bigl\Vert I_{R}^{2} \bigr\Vert _{\infty }, \\& \overline{K}^{6} = ( \Pi _{61}+\Pi _{62} ) ^{2} \bigl\Vert I_{T}^{2} \bigr\Vert _{\infty }, \\& \overline{K}^{7} = ( \Pi _{71}+\Pi _{72} ) ^{2} \bigl\Vert R^{2} \bigr\Vert _{\infty }, \\& \overline{K}^{8} = ( \Pi _{81}+\Pi _{82} ) ^{2} \bigl\Vert V^{2} \bigr\Vert _{\infty }. \end{aligned}$$

Also, we have

48 $$\begin{aligned}& \begin{aligned} \bigl\vert F_{2} ( I,t ) \bigr\vert ^{2} &= \bigl\vert \delta ( t ) \bigl( w ( \beta I_{D}+ \gamma I_{A}+ \delta _{1}I_{R}+\delta _{2}I_{T} ) \bigr) S+\delta ( t ) \alpha IS- ( \varepsilon +\xi +\lambda +\mu _{1} ) I \bigr\vert ^{2} \\ &\leq \bigl\vert \delta ( t ) \bigl( w ( \beta I_{D}+ \gamma I_{A}+\delta _{1}I_{R}+\delta _{2}I_{T} ) \bigr) S+ \delta ( t ) \alpha S- ( \varepsilon +\xi +\lambda + \mu _{1} ) \bigr\vert \vert I \vert ^{2} \\ &< \bigl( \vert S \vert ^{2}+1 \bigr) \sup_{t\in [ 0,T ] } \bigl\vert \delta ( t ) \bigl( w ( \beta I_{D}+\gamma I_{A}+\delta _{1}I_{R}+\delta _{2}I_{T} ) \bigr) S+\delta ( t ) \alpha S \\ &\quad{} - ( \varepsilon +\xi +\lambda +\mu _{1} ) \bigr\vert ^{2} \\ &< K^{2} \bigl( \vert I \vert ^{2}+1 \bigr), \end{aligned} \\& \begin{aligned} \bigl\vert F_{3} ( I_{A},t ) \bigr\vert ^{2} &= \bigl\vert \xi I- ( \theta +\mu +\chi +\mu _{1} ) I_{A} \bigr\vert ^{2} \\ &\leq \bigl\vert \xi I- ( \theta +\mu +\chi +\mu _{1} ) \bigr\vert ^{2} \vert I_{A} \vert ^{2} \\ &\leq \bigl( \vert I_{A} \vert ^{2}+1 \bigr) \sup _{t \in [ 0,T ] } \bigl\vert \xi I- ( \theta +\mu +\chi +\mu _{1} ) \bigr\vert ^{2} \\ &\leq K^{3} \bigl( \vert I_{A} \vert ^{2}+1 \bigr), \end{aligned} \\& \begin{aligned} \bigl\vert F_{4} ( I_{A},t ) \bigr\vert ^{2} &= \bigl\vert \varepsilon I- ( \eta +\varphi +\mu _{1} ) I_{D} \bigr\vert ^{2} \\ &\leq \bigl( \vert I_{D} \vert ^{2}+1 \bigr) \sup _{t \in [ 0,T ] } \bigl\vert \varepsilon I- ( \eta +\varphi +\mu _{1} ) \bigr\vert ^{2} \\ &\leq K^{4} \bigl( \vert I_{D} \vert ^{2}+1 \bigr), \end{aligned} \\& \begin{aligned} \bigl\vert F_{5} ( I_{R},t ) \bigr\vert ^{2} &\leq \bigl( \vert I_{R} \vert ^{2}+1 \bigr) \sup_{t\in [ 0,T ] } \bigl\vert \eta I_{D}+\theta I_{A}- ( v+ \xi +\mu _{1} ) \bigr\vert ^{2} \\ &\leq K^{5} \bigl( \vert I_{R} \vert ^{2}+1 \bigr), \end{aligned} \\& \begin{aligned} \bigl\vert F_{6} ( I_{T},t ) \bigr\vert ^{2} &\leq \bigl( \vert I_{T} \vert ^{2}+1 \bigr) \sup_{t\in [ 0,T ] } \bigl\vert \mu I_{A}+vI_{R}- ( \sigma + \tau +\mu _{1} ) \bigr\vert ^{2} \\ &\leq K^{6} \bigl( \vert I_{T} \vert ^{2}+1 \bigr), \end{aligned} \\& \begin{aligned} \bigl\vert F_{6} ( I_{T},t ) \bigr\vert ^{2} &\leq \bigl( \vert I_{T} \vert ^{2}+1 \bigr) \sup_{t\in [ 0,T ] } \bigl\vert \mu I_{A}+vI_{R}- ( \sigma + \tau +\mu _{1} ) \bigr\vert ^{2} \\ &\leq K^{6} \bigl( \vert I_{T} \vert ^{2}+1 \bigr), \end{aligned} \\& \begin{aligned} \bigl\vert F_{7} ( R,t ) \bigr\vert ^{2} &\leq \bigl( \vert R \vert ^{2}+1 \bigr) \sup _{t\in [ 0,T ] } \bigl\vert \lambda I+\varphi I_{D}+\chi I_{A}+\xi I_{R}+ \sigma I_{T}- ( \Phi +\mu _{1} ) \bigr\vert ^{2} \\ &\leq K^{7} \bigl( \vert R \vert ^{2}+1 \bigr) . \end{aligned} \end{aligned}$$

Finally, we have

$$\begin{aligned} \bigl\vert F_{8} ( V,t ) \bigr\vert ^{2} \leq & \bigl( \vert V \vert ^{2}+1 \bigr) \sup_{t\in [ 0,T ] } \vert \gamma _{1}S+\Phi R-\mu _{1} \vert ^{2} \\ \leq &K^{8} \bigl( \vert V \vert ^{2}+1 \bigr) . \end{aligned}$$

Both $G_{i}$ and $F_{i}$ verify the second condition. Therefore, according to the above theorem, the system has a unique system solution.

### Numerical simulation for the stochastic model

**Figure 22:**
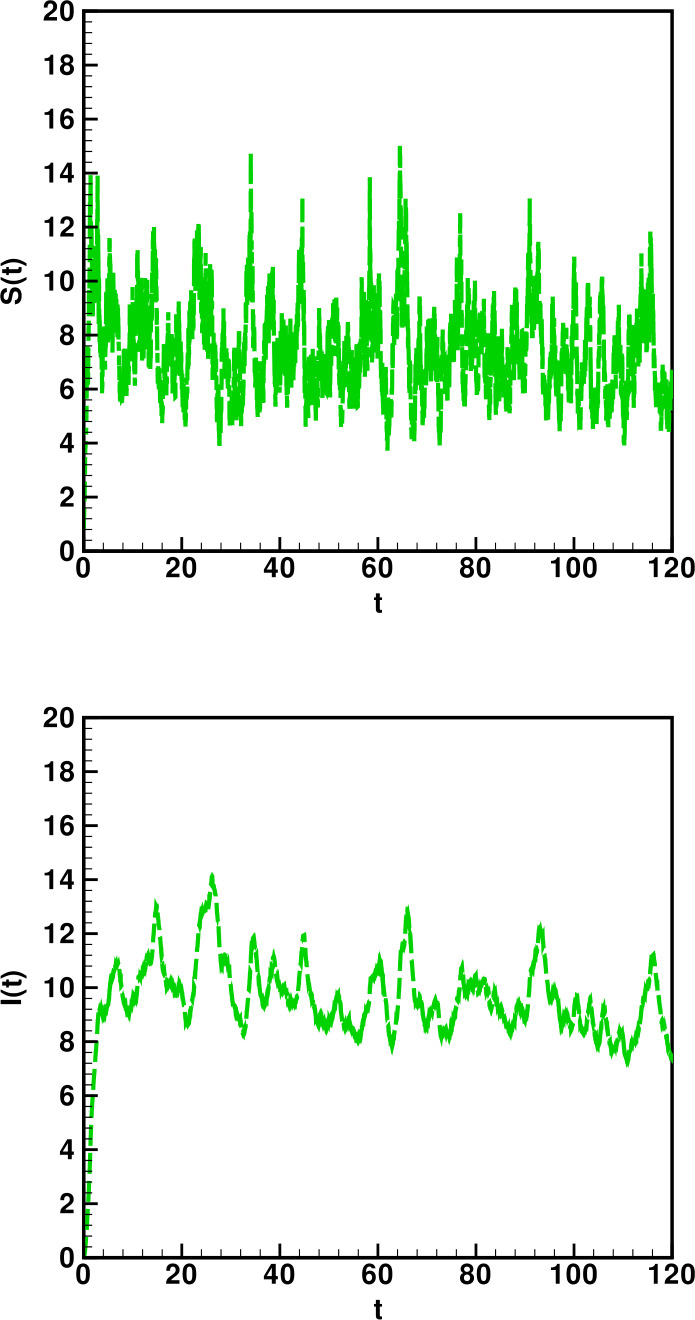
Stochastic behavior of ${S} ( t )$ and ${I} ( t )$ classes

**Figure 23:**
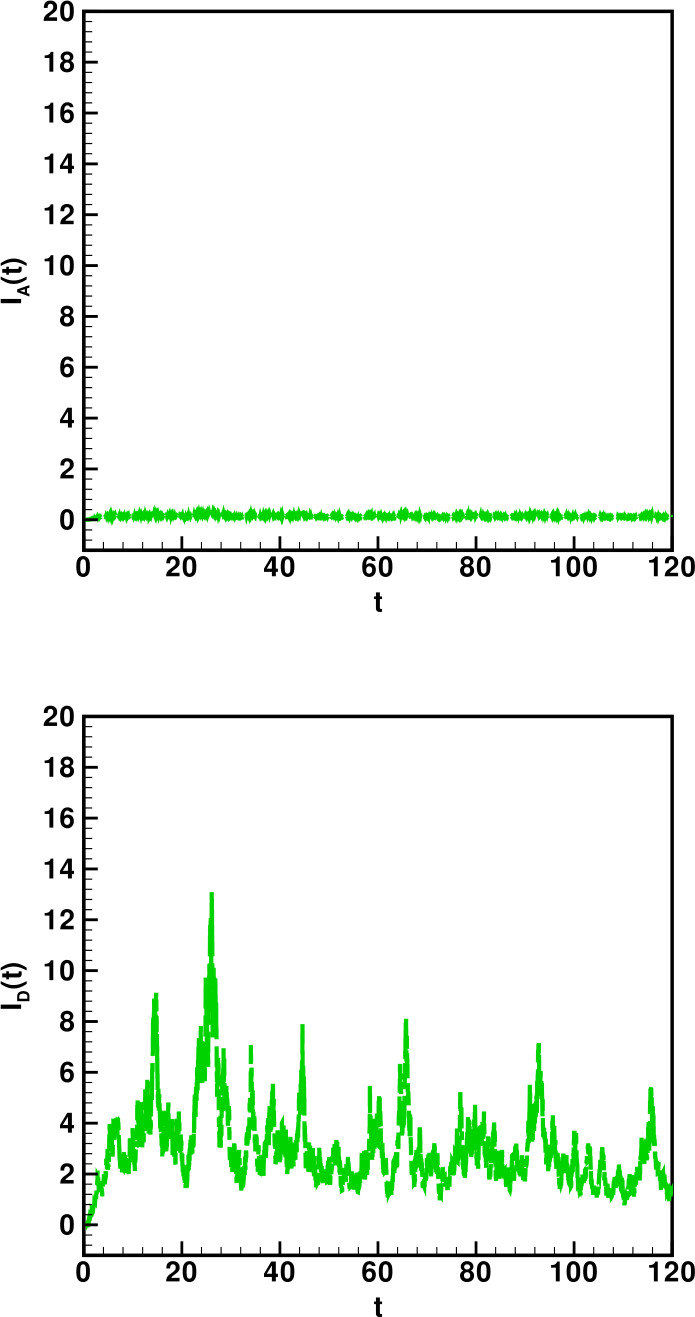
Stochastic behavior of ${I}_{A} ( t )$ and ${I}_{D} ( t )$ classes

**Figure 24:**
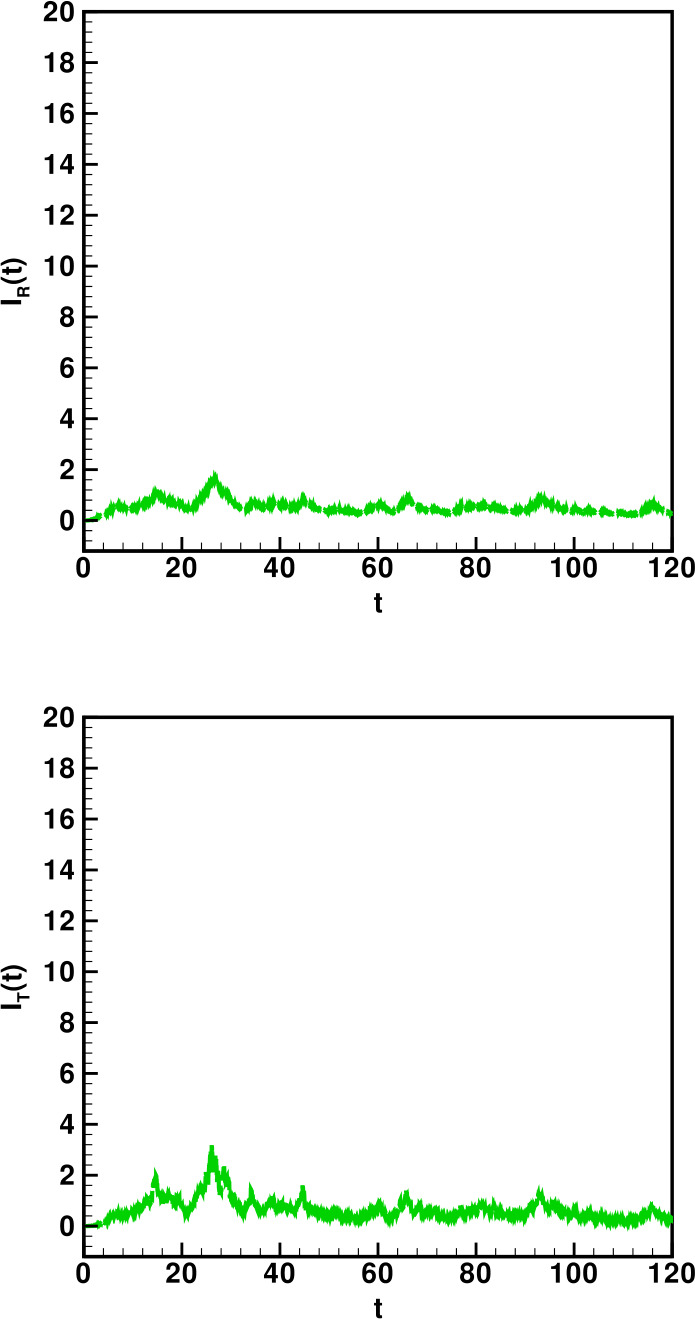
Stochastic behavior of ${I}_{R} ( t )$ and $I_{T} ( t ) $ classes

**Figure 25:**
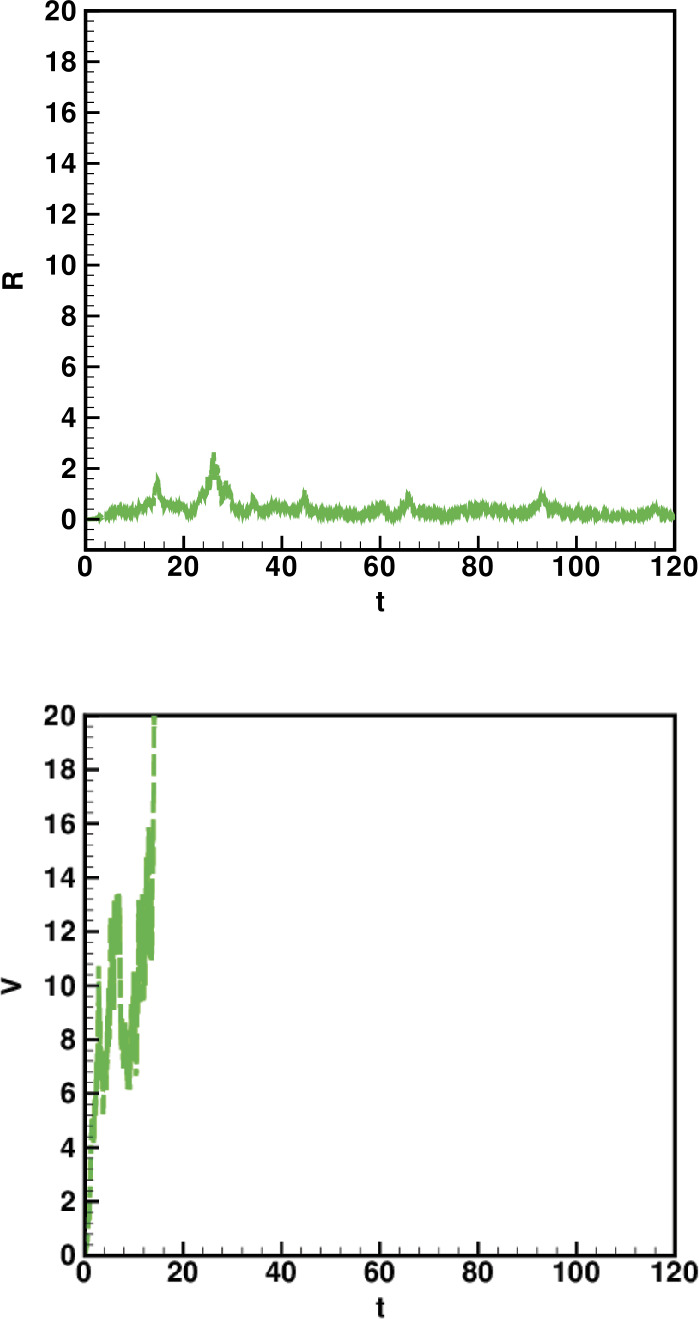
Stochastic behavior of ${R} ( t )$ and ${V} ( t )$ classes

Numerical solutions of the suggested stochastic model are presented in Figs. 22–25. The numerical solution depicts the future stochastic behavior of the susceptible class, five sub-classes of the infected population, the recovered class, the death class, and the vaccination class. These are depicted in figures below.

## Atangana–Seda modified scheme

The mathematical model considered in this work has the ability to depict two to three waves of COVID-19 spread. The model is subjected to a system of initial conditions. Additionally, the model is nonlinear, thus it is impossible to obtain exact solutions to the system, thus numerical schemes are needed. We present a numerical scheme based on the Newton polynomial [18]. However, one needs the initial condition and two additional components for the scheme to be implemented. In this section, we present a modified version that will not need the two additional components, and then the scheme will be used later to provide numerical solutions for the suggested COVID-19 model with different differential operators. We start with the classical case, the following is considered:

49 $$ \frac{dy ( t ) }{dt}=f \bigl( t,y ( t ) \bigr) . $$

Then

50 $$ y^{n+1}=y^{n}+ \biggl\{ \frac{5}{12}f \bigl( t_{n-2},y^{n-2} \bigr) - \frac{4}{3}f \bigl( t_{n-1},y^{n-1} \bigr) +\frac{5}{12}f \bigl( t_{n},y^{n} \bigr) \biggr\} \Delta t. $$

To reduce these requirements, we proceed as follows:

51 $$ \frac{y^{n}-y^{n-1}}{\Delta t}=f \bigl( t_{n},y^{n} \bigr) \quad \Rightarrow \quad y^{n-1}=y^{n}-f \bigl( t_{n},y^{n} \bigr) \Delta t. $$

On the other hand,

52 $$ \frac{y^{n-1}-y^{n-2}}{\Delta t}=f \bigl( t_{n-1},y^{n-1} \bigr) $$

or

53 $$\begin{aligned} y^{n-2} =&y^{n-1}-f \bigl( t_{n-1},y^{n-1} \bigr) \Delta t \\ =&y^{n}-\Delta tf \bigl( t_{n},y^{n} \bigr) - \Delta tf \bigl( t_{n-1},y^{n}-f \bigl( t_{n},y^{n} \bigr) \Delta t \bigr) . \end{aligned}$$

Replacing $y^{n-2}$ and $y^{n-1}$ with their values, we obtain

54 $$\begin{aligned} y^{n+1} =&y^{n}+\frac{5}{12}\Delta tf \bigl( t_{n-2},y^{n}-\Delta tf \bigl( t_{n},y^{n} \bigr) -\Delta tf \bigl( t_{n-1},y^{n}-f \bigl( t_{n},y^{n} \bigr) \Delta t \bigr) \bigr) \\ &{}-\frac{4}{3}f \bigl( t_{n-1},y^{n}-f \bigl( t_{n},y^{n} \bigr) \Delta t \bigr) +\frac{23}{12}f \bigl( t_{n},y^{n} \bigr) \Delta t. \end{aligned}$$

The above does not need $y^{1}$ and $y^{2}$, only the initial condition. With the Caputo–Fabrizio derivative, we consider the following:

55 $$ {}_{0}^{CF}D_{t}^{\alpha }y ( t ) =f \bigl( t,y ( t ) \bigr) . $$

From the definition of the Caputo–Fabrizio integral, we can reformulate the above equation as follows:

56 $$ y ( t ) -y ( 0 ) = \frac{1-\alpha }{M ( \alpha ) }f \bigl( t,y ( t ) \bigr) + \frac{\alpha }{M ( \alpha ) }\int _{0}^{t}f \bigl( \tau ,y ( \tau ) \bigr)\,d\tau . $$

We have, at the point $t_{n+1}= ( n+1 ) \Delta t$,

57 $$ y ( t_{n+1} ) -y ( 0 ) = \frac{1-\alpha }{M ( \alpha ) }f \bigl( t_{n+1},y ( t_{n+1} ) \bigr) +\frac{\alpha }{M ( \alpha ) } \int _{0}^{t_{n+1}}f \bigl( \tau ,y ( \tau ) \bigr)\,d \tau , $$

and at the point $t_{n}=n\Delta t$,

58 $$ y ( t_{n} ) -y ( 0 ) = \frac{1-\alpha }{M ( \alpha ) }f \bigl( t_{n},y ( t_{n} ) \bigr) +\frac{\alpha }{M ( \alpha ) } \int _{0}^{t_{n}}f \bigl( \tau ,y ( \tau ) \bigr)\,d \tau . $$

Taking the difference of these equations, we can write the following:

59 $$\begin{array}{rcl} y(t_{n+1})-y(t_{n}) & = & \frac{1-\alpha }{M(\alpha )}\left[f\left(t_{n+1},y(t_{n+1})\right)-f\left(t_{n},y(t_{n})\right)\right]\\ & & +\frac{\alpha }{M(\alpha )}\int_{t_{n}}^{t_{n+1}}f\left(\tau ,y(\tau )\right)\;d\tau \\ & = & \frac{1-\alpha }{M(\alpha )}\left[\begin{array}{c} f(t_{n+1},y(t_{n})-\Delta tf(t_{n},y(t_{n})))\\ -f(t_{n},y(t_{n})) \end{array}\right]\\ & & +\frac{\alpha }{M(\alpha )}\left\{\begin{array}{c} \frac{5}{12}f(t_{n-2},y^{n}-\Delta tf(t_{n},y^{n})-\Delta tf(t_{n-1},y^{n}-f(t_{n},y^{n})\Delta t))\Delta t\\ -\frac{4}{3}f(t_{n-1},y^{n}-f(t_{n},y^{n})\Delta t)\Delta t\\ +\frac{23}{12}f(t_{n},y^{n})\Delta t \end{array}\right\}. \end{array}$$

With the Caputo derivative, we write

60 $$ \textstyle\begin{cases} {}_{0}^{C}D_{t}^{\alpha }y ( t ) =f ( t,y ( t ) ), \\ y ( 0 ) =y_{0}.\end{cases}$$

We convert the above into

61 $$ y ( t ) -y ( 0 ) = \frac{1}{\Gamma ( \alpha ) }\int _{0}^{t}f \bigl( \tau ,y ( \tau ) \bigr) ( t- \tau ) ^{\alpha -1}\,d\tau . $$

At the point $t_{n+1}= ( n+1 ) \Delta t$, we have the following:

$$ y ( t_{n+1} ) -y ( 0 ) = \frac{1}{\Gamma ( \alpha ) } \int _{0}^{t_{n+1}}f \bigl( \tau ,y ( \tau ) \bigr) ( t_{n+1}-\tau ) ^{ \alpha -1}\,d\tau , $$

and we write

$$ y ( t_{n+1} ) =y ( 0 ) + \frac{1}{\Gamma ( \alpha ) }\sum _{j=2}^{n} \int _{t_{j}}^{t_{j+1}}f \bigl( \tau ,y ( \tau ) \bigr) ( t_{n+1}-\tau ) ^{\alpha -1}\,d\tau . $$

After putting the Newton polynomial into the above equation, the above equation can be written as follows:

62 $$\begin{array}{rl} y^{n+1}= & y^{0}+\frac{{\left(\Delta t\right)}^{\alpha }}{\Gamma (\alpha +1)}\sum_{j=2}^{n}f\left(t_{j-2},y^{j-2}\right)\left[{\left(n-j+1\right)}^{\alpha }-{\left(n-j\right)}^{\alpha }\right]\\ & +\frac{{\left(\Delta t\right)}^{\alpha }}{\Gamma (\alpha +2)}\sum_{j=2}^{n}\left[f\left(t_{j-1},y^{j-1}\right)-f\left(t_{j-2},y^{j-2}\right)\right]\\ & \times \left[\begin{array}{c} {\left(n-j+1\right)}^{\alpha }(n-j+3+2\alpha )\\ -{\left(n-j\right)}^{\alpha }(n-j+3+3\alpha ) \end{array}\right]\\ & +\frac{\alpha {\left(\Delta t\right)}^{\alpha }}{2\Gamma (\alpha +3)}\sum_{j=2}^{n}\left[\begin{array}{c} f(t_{j},y^{j})-2f(t_{j-1},y^{j-1})\\ +f(t_{j-2},y^{j-2}) \end{array}\right]\\ & \times \left[\begin{array}{c} {\left(n-j+1\right)}^{\alpha }\left[\begin{array}{c} 2{\left(n-j\right)}^{2}+(3\alpha +10)(n-j)\\ +2\alpha^{2}+9\alpha +12 \end{array}\right]\\ -{\left(n-j\right)}^{\alpha }\left[\begin{array}{c} 2{\left(n-j\right)}^{2}+(5\alpha +10)(n-j)\\ +6\alpha^{2}+18\alpha +12 \end{array}\right] \end{array}\right], \end{array}$$

where

63 $$\begin{aligned}& f \bigl( t_{j-1},y^{j-1} \bigr) =f \bigl( t_{j-1},y^{j}-f \bigl( t_{j},y^{j} \bigr) \Delta t \bigr), \\& f \bigl( t_{j-2},y^{j-2} \bigr) =f \bigl( t_{j-2},y^{j}- \Delta tf \bigl( t_{j},y^{j} \bigr) -\Delta tf \bigl( t_{j-1},y^{j}-f \bigl( t_{j},y^{j} \bigr) \Delta t \bigr) \bigr) . \end{aligned}$$

With Atangana–Baleanu, we have

64 $$ \textstyle\begin{cases} {}_{0}^{ABC}D_{t}^{\alpha }y ( t ) =f ( t,y ( t ) ), \\ y ( 0 ) =y_{0}.\end{cases}$$

We transform the above equation into

65 $$ y ( t ) -y ( 0 ) = \frac{1-\alpha }{AB ( \alpha ) }f \bigl( t,y ( t ) \bigr) + \frac{\alpha }{AB ( \alpha ) \Gamma ( \alpha ) } \int _{0}^{t}f \bigl( \tau ,y ( \tau ) \bigr) ( t- \tau ) ^{\alpha -1}\,d\tau . $$

At the point $t_{n+1}= ( n+1 ) \Delta t$, we have the following:

66 $$\begin{aligned} y ( t_{n+1} ) -y ( 0 ) =&\frac{1-\alpha }{AB ( \alpha ) }f \bigl( t,y ( t ) \bigr) \\ &{}+ \frac{\alpha }{AB ( \alpha ) \Gamma ( \alpha ) }\int _{0}^{t_{n+1}}f \bigl( \tau ,y ( \tau ) \bigr) ( t_{n+1}-\tau ) ^{\alpha -1}\,d\tau , \end{aligned}$$

and we write

67 $$\begin{aligned} y ( t_{n+1} ) =&y ( 0 ) + \frac{1-\alpha }{AB ( \alpha ) }f \bigl( t_{n+1},y^{n+1} \bigr) \\ &{}+ \frac{\alpha }{AB ( \alpha ) \Gamma ( \alpha ) }\sum_{j=2}^{n} \int _{t_{j}}^{t_{j+1}}f \bigl( \tau ,y ( \tau ) \bigr) ( t_{n+1}-\tau ) ^{\alpha -1}\,d\tau . \end{aligned}$$

After putting the Newton polynomial into the above equation, the above equation can be written as follows:

68 $$\begin{array}{rcl} y^{n+1} & = & y^{0}+\frac{1-\alpha }{AB(\alpha )}f\left(t_{n+1},y(t_{n})-\Delta tf\left(t_{n},y(t_{n})\right)\right)\\ & & +\frac{\alpha {\left(\Delta t\right)}^{\alpha }}{AB(\alpha )\Gamma (\alpha +1)}\sum_{j=2}^{n}f\left(\begin{array}{c} t_{j-2},y^{j}-\Delta tf(t_{j},y^{j})\\ -\Delta tf(t_{j-1},y^{j}-f(t_{j},y^{j})\Delta t) \end{array}\right)\\ & & \times \left[{\left(n-j+1\right)}^{\alpha }-{\left(n-j\right)}^{\alpha }\right]\\ & & +\frac{\alpha {\left(\Delta t\right)}^{\alpha }}{AB(\alpha )\Gamma (\alpha +2)}\sum_{j=2}^{n}\left[\begin{array}{c} f(t_{j-1},y^{j}-f(t_{j},y^{j})\Delta t)\\ -f(t_{j-2},y^{j}-\Delta tf(t_{j},y^{j})-\Delta tf(t_{j-1},y^{j}-f(t_{j},y^{j})\Delta t)) \end{array}\right]\\ & & \times \left[\begin{array}{c} {\left(n-j+1\right)}^{\alpha }(n-j+3+2\alpha )\\ -{\left(n-j\right)}^{\alpha }(n-j+3+3\alpha ) \end{array}\right]\\ & & +\frac{\alpha {\left(\Delta t\right)}^{\alpha }}{2AB(\alpha )\Gamma (\alpha +3)}\sum_{j=2}^{n}\left[\begin{array}{c} f(t_{j},y^{j})-2f(t_{j-1},y^{j-1})\\ +f\left(\begin{array}{c} t_{j-2},y^{j}-\Delta tf(t_{j},y^{j})\\ -\Delta tf(t_{j-1},y^{j}-f(t_{j},y^{j})\Delta t) \end{array}\right) \end{array}\right]\\ & & \times \left[\begin{array}{c} {\left(n-j+1\right)}^{\alpha }\left[\begin{array}{c} 2{\left(n-j\right)}^{2}+(3\alpha +10)(n-j)\\ +2\alpha^{2}+9\alpha +12 \end{array}\right]\\ -{\left(n-j\right)}^{\alpha }\left[\begin{array}{c} 2{\left(n-j\right)}^{2}+(5\alpha +10)(n-j)\\ +6\alpha^{2}+18\alpha +12 \end{array}\right] \end{array}\right]. \end{array}$$

With the Caputo–Fabrizio fractal-fractional derivative, we consider

69 $$\begin{aligned}& \begin{aligned}[t] & {}_{0}^{FFE}D_{t}^{\alpha ,\beta }y ( t ) =f \bigl( t,y ( t ) \bigr), \\ &y ( 0 ) =y_{0}. \end{aligned} \end{aligned}$$

Applying the associated integral operator with exponential kernel, we can reformulate equation (69) as follows:

70 $$ y ( t ) =\frac{1-\alpha }{M ( \alpha ) }t^{1- \beta }f \bigl( t,y ( t ) \bigr) + \frac{\alpha }{M ( \alpha ) }\int _{0}^{t}f \bigl( \tau ,y ( \tau ) \bigr) \tau ^{1- \beta }\,d\tau . $$

At the point $t_{n+1}= ( n+1 ) \Delta t$,

71 $$ y ( t_{n+1} ) = \frac{1-\alpha }{M ( \alpha ) }t_{n+1}^{1-\beta }f \bigl( t_{n+1},y ( t_{n+1} ) \bigr) + \frac{\alpha }{M ( \alpha ) } \int _{0}^{t_{n+1}}f \bigl( \tau ,y ( \tau ) \bigr) \tau ^{1-\beta }\,d\tau , $$

and at the point $t_{n}=n\Delta t$, we have

72 $$ y ( t_{n} ) =\frac{1-\alpha }{M ( \alpha ) }t_{n}^{1-\beta }f \bigl( t_{n},y ( t_{n} ) \bigr) + \frac{\alpha }{M ( \alpha ) } \int _{0}^{t_{n}}f \bigl( \tau ,y ( \tau ) \bigr) \tau ^{1-\beta }\,d\tau . $$

If we take the difference of these equations, we obtain the following equation:

73 $$\begin{array}{rcl} y(t_{n+1})-y(t_{n}) & = & \frac{1-\alpha }{M(\alpha )}\left[\begin{array}{c} t_{n+1}^{1-\beta }f(t_{n+1},y(t_{n+1}))\\ -t_{n}^{1-\beta }f(t_{n},y(t_{n})) \end{array}\right]\\ & & +\frac{\alpha }{M(\alpha )}\int_{t_{n}}^{t_{n+1}}f\left(\tau ,y(\tau )\right)\tau^{1-\beta }\;d\tau . \end{array}$$

For brevity, we consider

74 $$\begin{aligned} y ( t_{n+1} ) -y ( t_{n} ) =&\frac{1-\alpha }{M ( \alpha ) } \bigl[ F \bigl( t_{n+1},y ( t_{n+1} ) \bigr) -F \bigl( t_{n},y ( t_{n} ) \bigr) \bigr] \\ &{}+\frac{\alpha }{M ( \alpha ) } \int _{t_{n}}^{t_{n+1}}F \bigl( \tau ,y ( \tau ) \bigr)\,d \tau , \end{aligned}$$

where

75 $$ F \bigl( t,y ( t ) \bigr) =f \bigl( t,y ( t ) \bigr) t^{1-\beta }. $$

We can rearrange the above scheme as follows:

76 $$\begin{array}{rcl} y^{n+1}-y^{n} & = & \frac{1-\alpha }{M(\alpha )}\left[\begin{array}{c} F(t_{n+1},y(t_{n})-\Delta tf(t_{n},y(t_{n})))\\ -F(t_{n},y(t_{n})) \end{array}\right]\\ & & +\frac{\alpha }{M(\alpha )}\left\{\begin{array}{c} \frac{5}{12}F(t_{n-2},y^{n}-\Delta tf(t_{n},y^{n})-\Delta tF(t_{n-1},y^{n}-f(t_{n},y^{n})\Delta t))\Delta t\\ -\frac{4}{3}F(t_{n-1},y^{n}-f(t_{n},y^{n})\Delta t)\Delta t\\ +\frac{23}{12}F(t_{n},y^{n})\Delta t \end{array}\right\}. \end{array}$$

If we replace $F ( t,y ( t ) ) $ with its value, we can solve our equation numerically with the following scheme:

77 $$\begin{array}{rcl} y^{n+1}-y^{n} & = & \frac{1-\alpha }{M(\alpha )}\left[\begin{array}{c} t_{n+1}^{1-\beta }f(t_{n+1},y(t_{n})-\Delta tf(t_{n},y(t_{n})))\\ -t_{n}^{1-\beta }f(t_{n},y(t_{n})) \end{array}\right]\\ & & +\frac{\alpha }{M(\alpha )}\left\{\begin{array}{c} t_{n-2}^{1-\beta }\frac{5}{12}F(t_{n-2},y^{n}-\Delta tf(t_{n},y^{n})-\Delta tf(t_{n-1},y^{n}-f(t_{n},y^{n})\Delta t))\Delta t\\ -\frac{4}{3}t_{n-1}^{1-\beta }f(t_{n-1},y^{n}-f(t_{n},y^{n})\Delta t)\Delta t\\ +\frac{23}{12}t_{n}^{1-\beta }f(t_{n},y^{n})\Delta t \end{array}\right\}. \end{array}$$

With the Atangana–Baleanu fractal-fractional derivative, we write

78 $$\begin{aligned}& {}_{0}^{FFM}D_{t}^{\alpha ,\beta }y ( t ) =f \bigl( t,y ( t ) \bigr), \\& y ( 0 ) =y_{0}. \end{aligned}$$

Applying the new fractional integral with Mittag-Leffler kernel, we transform the above equation into

79 $$\begin{aligned} y ( t ) =&y ( 0 ) + \frac{1-\alpha }{AB ( \alpha ) }t^{1-\beta }f \bigl( t,y ( t ) \bigr) \\ &{}+ \frac{\alpha }{AB ( \alpha ) \Gamma ( \alpha ) }\int _{0}^{t}f \bigl( \tau ,y ( \tau ) \bigr) ( t- \tau ) ^{\alpha -1}\tau ^{1-\beta }\,d\tau . \end{aligned}$$

At the point $t_{n+1}= ( n+1 ) \Delta t$, we obtain the following:

80 $$\begin{aligned} y ( t_{n+1} ) =&y ( 0 ) + \frac{1-\alpha }{AB ( \alpha ) }t_{n+1}^{1-\beta }f \bigl( t_{n+1},y ( t_{n+1} ) \bigr) \\ &{}+ \frac{\alpha }{AB ( \alpha ) \Gamma ( \alpha ) }\int _{0}^{t_{n+1}}f \bigl( \tau ,y ( \tau ) \bigr) ( t_{n+1}-\tau ) ^{\alpha -1}\tau ^{1-\beta }\,d\tau . \end{aligned}$$

For simplicity, we shall take

81 $$ F \bigl( t,y ( t ) \bigr) =f \bigl( t,y ( t ) \bigr) t^{1-\beta }. $$

We also have

82 $$\begin{aligned} y ( t_{n+1} ) =&y ( 0 ) + \frac{1-\alpha }{AB ( \alpha ) }F \bigl( t_{n+1},y ( t_{n+1} ) \bigr) \\ &{}+ \frac{\alpha }{AB ( \alpha ) \Gamma ( \alpha ) }\sum_{j=2}^{n} \int _{t_{j}}^{t_{j+1}}F \bigl( \tau ,y ( \tau ) \bigr) ( t_{n+1}-\tau ) ^{\alpha -1}\,d\tau . \end{aligned}$$

Replacing them into the above equation and substituting $F ( t,y ( t ) ) =f ( t,y ( t ) ) t^{1-\beta }$, we can get the following numerical scheme:

83 $$\begin{array}{rcl} y^{n+1} & = & \frac{1-\alpha }{AB(\alpha )}t_{n+1}^{1-\beta }f\left(t_{n+1},y(t_{n+1})\right)\\ & & +\frac{\alpha {\left(\Delta t\right)}^{\alpha }}{AB(\alpha )\Gamma (\alpha +1)}\sum_{j=2}^{n}t_{j-2}^{1-\beta }f\left(\begin{array}{c} t_{j-2},y^{j}-\Delta tf(t_{j},y^{j})\\ -\Delta tf(t_{j-1},y^{j}-f(t_{j},y^{j})\Delta t) \end{array}\right)\\ & & \times \left[{\left(n-j+1\right)}^{\alpha }-{\left(n-j\right)}^{\alpha }\right]\\ & & +\frac{\alpha {\left(\Delta t\right)}^{\alpha }}{AB(\alpha )\Gamma (\alpha +2)}\sum_{j=2}^{n}\left[\begin{array}{c} t_{j-1}^{1-\beta }f(t_{j-1},y^{j}-f(t_{j},y^{j})\Delta t)\\ -t_{j-2}^{1-\beta }f\left(\begin{array}{c} t_{j-2},y^{j}-\Delta tf(t_{j},y^{j})\\ -\Delta tf(t_{j-1},y^{j}-f(t_{j},y^{j})\Delta t) \end{array}\right) \end{array}\right]\\ & & \times \left[\begin{array}{c} {\left(n-j+1\right)}^{\alpha }(n-j+3+2\alpha )\\ -{\left(n-j\right)}^{\alpha }(n-j+3+3\alpha ) \end{array}\right]\\ & & +\frac{\alpha {\left(\Delta t\right)}^{\alpha }}{2AB(\alpha )\Gamma (\alpha +3)}\sum_{j=2}^{n}\left[\begin{array}{c} t_{j}^{1-\beta }g(t_{j},y^{j})\\ -2t_{j-1}^{1-\beta }f(t_{j-1},y^{j}-f(t_{j},y^{j})\Delta t)\\ +t_{j-2}^{1-\beta }f\left(\begin{array}{c} t_{j-2},y^{j}-\Delta tf(t_{j},y^{j})\\ -\Delta tf(t_{j-1},y^{j}-f(t_{j},y^{j})\Delta t) \end{array}\right) \end{array}\right]\\ & & \times \left[\begin{array}{c} {\left(n-j+1\right)}^{\alpha }\left[\begin{array}{c} 2{\left(n-j\right)}^{2}+(3\alpha +10)(n-j)\\ +2\alpha^{2}+9\alpha +12 \end{array}\right]\\ -{\left(n-j\right)}^{\alpha }\left[\begin{array}{c} 2{\left(n-j\right)}^{2}+(5\alpha +10)(n-j)\\ +6\alpha^{2}+18\alpha +12 \end{array}\right] \end{array}\right]. \end{array}$$

With the Caputo fractal-fractional derivative, we consider the following:

84 $$\begin{aligned}& {}_{0}^{FFP}D_{t}^{\alpha ,\beta }y ( t ) =f \bigl( t,y ( t ) \bigr), \\& y ( 0 ) =y_{0}. \end{aligned}$$

Applying the new fractional integral with power-law kernel, we transform the above equation into

85 $$ y ( t ) =y ( 0 ) + \frac{1}{\Gamma ( \alpha ) }\int _{0}^{t}f \bigl( \tau ,y ( \tau ) \bigr) ( t- \tau ) ^{\alpha -1}\tau ^{1-\beta }\,d\tau . $$

At the point $t_{n+1}= ( n+1 ) \Delta t$, we obtain the following:

86 $$ y ( t_{n+1} ) =y ( 0 ) + \frac{1}{\Gamma ( \alpha ) } \int _{0}^{t_{n+1}}f \bigl( \tau ,y ( \tau ) \bigr) ( t_{n+1}-\tau ) ^{ \alpha -1}\tau ^{1-\beta }\,d\tau . $$

For simplicity, we shall take

87 $$ F \bigl( t,y ( t ) \bigr) =f \bigl( t,y ( t ) \bigr) t^{1-\beta }. $$

We also have

88 $$ y ( t_{n+1} ) =y ( 0 ) + \frac{1}{\Gamma ( \alpha ) }\sum _{j=2}^{n} \int _{t_{j}}^{t_{j+1}}F \bigl( \tau ,y ( \tau ) \bigr) ( t_{n+1}-\tau ) ^{\alpha -1}\,d\tau . $$

Replacing them into the above equation and substituting $F ( t,y ( t ) ) =f ( t,y ( t ) ) t^{1-\beta }$, we can get the following numerical scheme:

89 $$\begin{array}{rcl} y^{n+1} & = & \frac{{\left(\Delta t\right)}^{\alpha }}{\Gamma (\alpha +1)}\sum_{j=2}^{n}t_{j-2}^{1-\beta }f\left(\begin{array}{c} t_{j-2},y^{j}-\Delta tf(t_{j},y^{j})\\ -\Delta tf(t_{j-1},y^{j}-f(t_{j},y^{j})\Delta t) \end{array}\right)\\ & & \times \left[{\left(n-j+1\right)}^{\alpha }-{\left(n-j\right)}^{\alpha }\right]\\ & & +\frac{{\left(\Delta t\right)}^{\alpha }}{\Gamma (\alpha +2)}\sum_{j=2}^{n}\left[\begin{array}{c} t_{j-1}^{1-\beta }f(t_{j-1},y^{j}-f(t_{j},y^{j})\Delta t)\\ -t_{j-2}^{1-\beta }f\left(\begin{array}{c} t_{j-2},y^{j}-\Delta tf(t_{j},y^{j})\\ -\Delta tf(t_{j-1},y^{j}-f(t_{j},y^{j})\Delta t) \end{array}\right) \end{array}\right]\\ & & \times \left[\begin{array}{c} {\left(n-j+1\right)}^{\alpha }(n-j+3+2\alpha )\\ -{\left(n-j\right)}^{\alpha }(n-j+3+3\alpha ) \end{array}\right]\\ & & +\frac{{\left(\Delta t\right)}^{\alpha }}{2\Gamma (\alpha +3)}\sum_{j=2}^{n}\left[\begin{array}{c} t_{j}^{1-\beta }g(t_{j},y^{j})\\ -2t_{j-1}^{1-\beta }f(t_{j-1},y^{j}-f(t_{j},y^{j})\Delta t)\\ +t_{j-2}^{1-\beta }f\left(\begin{array}{c} t_{j-2},y^{j}-\Delta tf(t_{j},y^{j})\\ -\Delta tf(t_{j-1},y^{j}-f(t_{j},y^{j})\Delta t) \end{array}\right) \end{array}\right]\\ & & \times \left[\begin{array}{c} {\left(n-j+1\right)}^{\alpha }\left[\begin{array}{c} 2{\left(n-j\right)}^{2}+(3\alpha +10)(n-j)\\ +2\alpha^{2}+9\alpha +12 \end{array}\right]\\ -{\left(n-j\right)}^{\alpha }\left[\begin{array}{c} 2{\left(n-j\right)}^{2}+(5\alpha +10)(n-j)\\ +6\alpha^{2}+18\alpha +12 \end{array}\right] \end{array}\right]. \end{array}$$

Finally, we present the numerical scheme with fractal-fractional derivative with variable order. We start with the Caputo–Fabrizio case:

90 $$\begin{aligned}& {}_{0}^{FFE}D_{t}^{\alpha ,\beta ( t ) }y ( t ) =f \bigl( t,y ( t ) \bigr), \\& y ( 0 ) =y_{0}. \end{aligned}$$

The above equation can be reformulated as follows:

91 $$\begin{aligned} y ( t ) =&\frac{1-\alpha }{M ( \alpha ) }t^{2- \beta ( t ) } \biggl[ -\beta \prime ( t ) \ln ( t ) +\frac{2-\beta ( t ) }{t} \biggr] f \bigl( t,y ( t ) \bigr) \\ &{}+\frac{\alpha }{M ( \alpha ) } \int _{0}^{t}f \bigl( \tau ,y ( \tau ) \bigr) \biggl[ \beta \prime ( \tau ) \ln ( \tau ) + \frac{2-\beta ( \tau ) }{\tau } \biggr] \tau ^{2-\beta ( \tau ) }\,d\tau . \end{aligned}$$

We write the above equation as follows:

92 $$\begin{array}{rcl} y(t_{n+1})-y(t_{n}) & = & \frac{1-\alpha }{M(\alpha )}\left[\begin{array}{c} t_{n+1}^{2-\beta (t_{n+1})}(-\frac{\beta (t_{n+2})-\beta (t_{n+1})}{\Delta t}\ln t_{n+1}+\frac{2-\beta (t_{n+1})}{t_{n+1}})f(t_{n+1},y(t_{n+1}))\\ -t_{n}^{2-\beta (t_{n})}(-\frac{\beta (t_{n+1})-\beta (t_{n})}{\Delta t}\ln t_{n}+\frac{2-\beta (t_{n})}{t_{n}})f(t_{n},y(t_{n})) \end{array}\right]\\ & & +\frac{\alpha }{M(\alpha )}\int_{t_{n}}^{t_{n+1}}f\left(\tau ,y(\tau )\right)\left[\beta \prime (\tau )\ln(\tau )+\frac{2-\beta (\tau )}{\tau }\right]\tau^{2-\beta (\tau )}\;d\tau . \end{array}$$

For simplicity, we take

93 $$ F \bigl( t,y ( t ) \bigr) =f \bigl( t,y ( t ) \bigr) \biggl[ -\beta \prime ( t ) \ln ( t ) +\frac{2-\beta ( t ) }{t} \biggr] t^{2-\beta ( t ) }, $$

and we have

94 $$\begin{aligned} y ( t_{n+1} ) -y ( t_{n} ) =&\frac{1-\alpha }{M ( \alpha ) } \bigl[ F \bigl( t_{n+1},y ( t_{n+1} ) \bigr) -F \bigl( t_{n},y ( t_{n} ) \bigr) \bigr] \\ &{}+\frac{\alpha }{M ( \alpha ) } \int _{t_{n}}^{t_{n+1}}F \bigl( \tau ,y ( \tau ) \bigr)\,d \tau . \end{aligned}$$

If we do the same routine and replace $F ( t,y ( t ) ) $ with its value, we have the following numerical approximation:

95 $$\begin{array}{rcl} y^{n+1} & = & y^{n}+\frac{1-\alpha }{M(\alpha )}\left[\begin{array}{c} t_{n+1}^{2-\beta (t_{n+1})}(-\frac{\beta (t_{n+2})-\beta (t_{n+1})}{\Delta t}\ln t_{n+1}+\frac{2-\beta (t_{n+1})}{t_{n+1}})\\ \times f(t_{n+1},y(t_{n})-\Delta tf(t_{n},y(t_{n})))\\ -t_{n}^{2-\beta (t_{n})}(-\frac{\beta (t_{n+1})-\beta (t_{n})}{\Delta t}\ln t_{n}+\frac{2-\beta (t_{n})}{t_{n}})\\ \times f(t_{n},y(t_{n})) \end{array}\right]\\ & & +\frac{\alpha }{M(\alpha )}\left\{\begin{array}{c} \frac{23}{12}t_{n}^{2-\beta (t_{n})}(-\frac{\beta (t_{n+1})-\beta (t_{n})}{\Delta t}\ln t_{n}+\frac{2-\beta (t_{n})}{t_{n}})\\ \times \frac{23}{12}f(t_{n},y^{n})\Delta t\\ -\frac{4}{3}t_{n-1}^{2-\beta (t_{n-1})}(-\frac{\beta (t_{n})-\beta (t_{n-1})}{\Delta t}\ln t_{n-1}+\frac{2-\beta (t_{n-1})}{t_{n-1}})\\ \times f(t_{n-1},y^{n}-f(t_{n},y^{n})\Delta t)\Delta t\\ +\frac{5}{12}t_{n-2}^{2-\beta (t_{n-2})}(-\frac{\beta (t_{n-1})-\beta (t_{n-2})}{\Delta t}\ln t_{n-2}+\frac{2-\beta (t_{n-2})}{t_{n-2}})\\ \times \frac{5}{12}f\left(\begin{array}{c} t_{n-2},y^{n}-\Delta tf(t_{n},y^{n})\\ -\Delta tf(t_{n-1},y^{n}-f(t_{n},y^{n})\Delta t) \end{array}\right)\Delta t \end{array}\right\}. \end{array}$$

We deal with our problem involving the new constant fractional order and variable fractal dimension

96 $$\begin{aligned}& {}_{0}^{FFM}D_{t}^{\alpha ,\beta ( t ) }y ( t ) =f \bigl( t,y ( t ) \bigr), \\& y ( 0 ) =y_{0}, \end{aligned}$$

where the kernel is the Mittag-Leffler kernel. If we integrate the above equation with the new integral operator including the Mittag-Leffler kernel, the above equation can be converted to

97 $$\begin{aligned} y ( t ) =&\frac{1-\alpha }{AB ( \alpha ) }t^{2- \beta ( t ) } \biggl[ -\beta \prime ( t ) \ln ( t ) +\frac{2-\beta ( t ) }{t} \biggr] f \bigl( t,y ( t ) \bigr) \\ &{}+ \frac{\alpha }{AB ( \alpha ) \Gamma ( \alpha ) }\int _{0}^{t}f \bigl( \tau ,y ( \tau ) \bigr) ( t- \tau ) ^{\alpha -1} \\ &{}\times \biggl[ -\beta \prime ( \tau ) \ln ( \tau ) +\frac{2-\beta ( \tau ) }{\tau } \biggr] \tau ^{2-\beta ( \tau ) }\,d\tau . \end{aligned}$$

At the point $t_{n+1}= ( n+1 ) \Delta t$, we have the following:

98 $$\begin{aligned} y ( t_{n+1} ) =&\frac{1-\alpha }{AB ( \alpha ) }t_{n+1}^{2-\beta ( t_{n+1} ) } \biggl( - \frac{\beta ( t_{n+2} ) -\beta ( t_{n+1} ) }{\Delta t}\ln t_{n+1}+ \frac{2-\beta ( t_{n+1} ) }{t_{n+1}} \biggr) \\ &{}\times f \bigl( t_{n+1},y ( t_{n+1} ) \bigr) \\ &{}+ \frac{\alpha }{AB ( \alpha ) \Gamma ( \alpha ) }\int _{0}^{t_{n+1}}f \bigl( \tau ,y ( \tau ) \bigr) ( t_{n+1}-s ) ^{\alpha -1} \\ &{}\times \biggl[ -\beta \prime ( \tau ) \ln ( \tau ) +\frac{2-\beta ( \tau ) }{\tau } \biggr] \tau ^{2-\beta ( \tau ) }\,d\tau . \end{aligned}$$

For brevity, we consider

99 $$ F \bigl( \tau ,y ( \tau ) \bigr) =f \bigl( \tau ,y ( \tau ) \bigr) \biggl[ - \beta \prime ( \tau ) \ln ( \tau ) + \frac{2-\beta ( \tau ) }{\tau } \biggr] \tau ^{2-\beta ( \tau ) }, $$

and we can write the following:

100 $$\begin{aligned} y ( t_{n+1} ) =&\frac{1-\alpha }{AB ( \alpha ) }t_{n+1}^{2-\beta ( t_{n+1} ) } \biggl( - \frac{\beta ( t_{n+2} ) -\beta ( t_{n+1} ) }{\Delta t}\ln t_{n+1}+ \frac{2-\beta ( t_{n+1} ) }{t_{n+1}} \biggr) \\ &{}\times f \bigl( t_{n+1},y ( t_{n+1} ) \bigr) \\ &{}+ \frac{\alpha }{AB ( \alpha ) \Gamma ( \alpha ) }\sum_{j=2}^{n} \int _{t_{j}}^{t_{j+1}}F \bigl( \tau ,y ( \tau ) \bigr) ( t_{n+1}-\tau ) ^{\alpha -1}\,d\tau . \end{aligned}$$

One can replace the Newton polynomial in the above equation as follows. Thus, we have the following scheme:

101 $$\begin{array}{rcl} y^{n+1} & = & \frac{1-\alpha }{AB(\alpha )}t_{n+1}^{2-\beta (t_{n+1})}\left(-\frac{\beta (t_{n+2})-\beta (t_{n+1})}{\Delta t}\ln t_{n+1}+\frac{2-\beta (t_{n+1})}{t_{n+1}}\right)\\ & & \times f\left(t_{n+1},y(t_{n+1})\right)\\ & & +\frac{\alpha {\left(\Delta t\right)}^{\alpha }}{AB(\alpha )\Gamma (\alpha +1)}\sum_{j=2}^{n}F\left(t_{j-2},y^{j-2}\right)\left[{\left(n-j+1\right)}^{\alpha }-{\left(n-j\right)}^{\alpha }\right]\\ & & +\frac{\alpha {\left(\Delta t\right)}^{\alpha }}{AB(\alpha )\Gamma (\alpha +2)}\sum_{j=2}^{n}\left[F\left(t_{j-1},y^{j-1}\right)-F\left(t_{j-2},y^{j-2}\right)\right]\\ & & \times \left[\begin{array}{c} {\left(n-j+1\right)}^{\alpha }(n-j+3+2\alpha )\\ -{\left(n-j\right)}^{\alpha }(n-j+3+3\alpha ) \end{array}\right]\\ & & +\frac{\alpha {\left(\Delta t\right)}^{\alpha }}{2AB(\alpha )\Gamma (\alpha +3)}\sum_{j=2}^{n}\left[F\left(t_{j},y^{j}\right)-2F\left(t_{j-1},y^{j-1}\right)+F\left(t_{j-2},y^{j-2}\right)\right]\\ & & \times \left[\begin{array}{c} {\left(n-j+1\right)}^{\alpha }\left[\begin{array}{c} 2{\left(n-j\right)}^{2}+(3\alpha +10)(n-j)\\ +2\alpha^{2}+9\alpha +12 \end{array}\right]\\ -{\left(n-j\right)}^{\alpha }\left[\begin{array}{c} 2{\left(n-j\right)}^{2}+(5\alpha +10)(n-j)\\ +6\alpha^{2}+18\alpha +12 \end{array}\right] \end{array}\right]. \end{array}$$

Replacing the function $G ( t,y ( t ) ) $ with its value, we can present the following scheme for numerical solution of our equation:

102 $$\begin{array}{rcl} y^{n+1} & = & \frac{1-\alpha }{AB(\alpha )}t_{n+1}^{2-\beta (t_{n+1})}\left(-\frac{\beta (t_{n+2})-\beta (t_{n+1})}{\Delta t}\ln t_{n+1}+\frac{2-\beta (t_{n+1})}{t_{n+1}}\right)\\ & & \times f\left(t_{n+1},y^{n}+f\left(t_{n},y^{n}\right)\Delta t\right)\\ & & +\frac{\alpha {\left(\Delta t\right)}^{\alpha }}{AB(\alpha )\Gamma (\alpha +1)}\sum_{j=2}^{n}t_{j-2}^{2-\beta (t_{j-2})}\left[-\frac{\beta (t_{j-1})-\beta (t_{j-2})}{\Delta t}\ln t_{j-2}+\frac{2-\beta (t_{j-2})}{t_{j-2}}\right]\\ & & \times f\left(\begin{array}{c} t_{j-2},y^{j}-\Delta tf(t_{j},y^{j})\\ -\Delta tf(t_{j-1},y^{j}-f(t_{j},y^{j})\Delta t) \end{array}\right)\left[{\left(n-j+1\right)}^{\alpha }-{\left(n-j\right)}^{\alpha }\right]\\ & & +\frac{\alpha {\left(\Delta t\right)}^{\alpha }}{AB(\alpha )\Gamma (\alpha +2)}\sum_{j=2}^{n}\left[\begin{array}{c} t_{j-1}^{2-\beta (t_{j-1})}[-\frac{\beta (t_{j})-\beta (t_{j-1})}{\Delta t}\ln t_{j-1}+\frac{2-\beta (t_{j-1})}{t_{j-1}}]\\ \times f(t_{j-1},y^{j}-f(t_{j},y^{j})\Delta t)\\ -t_{j-2}^{2-\beta (t_{j-2})}[-\frac{\beta (t_{j-1})-\beta (t_{j-2})}{\Delta t}\ln t_{j-2}+\frac{2-\beta (t_{j-2})}{t_{j-2}}]\\ \times f\left(\begin{array}{c} t_{j-2},y^{j}-\Delta tf(t_{j},y^{j})\\ -\Delta tf(t_{j-1},y^{j}-f(t_{j},y^{j})\Delta t) \end{array}\right) \end{array}\right]\\ & & \times \left[\begin{array}{c} {\left(n-j+1\right)}^{\alpha }(n-j+3+2\alpha )\\ -{\left(n-j\right)}^{\alpha }(n-j+3+3\alpha ) \end{array}\right]\\ & & +\frac{\alpha {\left(\Delta t\right)}^{\alpha }}{2AB(\alpha )\Gamma (\alpha +3)}\sum_{j=2}^{n}\left[\begin{array}{c} t_{j}^{2-\beta (t_{j})}[-\frac{\beta (t_{j+1})-\beta (t_{j})}{\Delta t}\ln t_{j}+\frac{2-\beta (t_{j})}{t_{j}}]\\ \times f(t_{j},y^{j})\Delta t\\ -2t_{j-1}^{2-\beta (t_{j-1})}[-\frac{\beta (t_{j})-\beta (t_{j-1})}{\Delta t}\ln t_{j-1}+\frac{2-\beta (t_{j-1})}{t_{j-1}}]\\ \times f(t_{j-1},y^{j}-f(t_{j},y^{j})\Delta t)\\ +t_{j-2}^{2-\beta (t_{j-2})}[-\frac{\beta (t_{j-1})-\beta (t_{j-2})}{\Delta t}\ln t_{j-2}+\frac{2-\beta (t_{j-2})}{t_{j-2}}]\\ \times f\left(\begin{array}{c} t_{j-2},y^{j}-\Delta tf(t_{j},y^{j})\\ -\Delta tf(t_{j-1},y^{j}-f(t_{j},y^{j})\Delta t) \end{array}\right) \end{array}\right]\\ & & \times \left[\begin{array}{c} {\left(n-j+1\right)}^{\alpha }\left[\begin{array}{c} 2{\left(n-j\right)}^{2}+(3\alpha +10)(n-j)\\ +2\alpha^{2}+9\alpha +12 \end{array}\right]\\ -{\left(n-j\right)}^{\alpha }\left[\begin{array}{c} 2{\left(n-j\right)}^{2}+(5\alpha +10)(n-j)\\ +6\alpha^{2}+18\alpha +12 \end{array}\right] \end{array}\right]. \end{array}$$

We deal with our problem involving the new constant fractional order and variable fractal dimension

103 $$\begin{aligned} \begin{aligned}[t] & {}_{0}^{FFP}D_{t}^{\alpha ,\beta ( t ) }y ( t ) =f \bigl( t,y ( t ) \bigr), \\ &y ( 0 ) =y_{0}, \end{aligned} \end{aligned}$$

where the kernel is the power-law kernel. If we integrate equation (103) with the new integral operator including the power-law kernel, the above equation can be converted to

104 $$ y ( t ) =\frac{1}{\Gamma ( \alpha ) } \int _{0}^{t}f \bigl( \tau ,y ( \tau ) \bigr) ( t- \tau ) ^{ \alpha -1} \biggl[ -\beta \prime ( \tau ) \ln ( \tau ) + \frac{2-\beta ( \tau ) }{\tau } \biggr] \tau ^{2-\beta ( \tau ) }\,d\tau . $$

At the point $t_{n+1}= ( n+1 ) \Delta t$, we have the following:

105 $$\begin{aligned} y ( t_{n+1} ) =&\frac{1}{\Gamma ( \alpha ) }\int _{0}^{t_{n+1}}f \bigl( \tau ,y ( \tau ) \bigr) ( t_{n+1}-s ) ^{\alpha -1} \\ &{}\times \biggl[ -\beta \prime ( \tau ) \ln ( \tau ) +\frac{2-\beta ( \tau ) }{\tau } \biggr] \tau ^{2-\beta ( \tau ) }\,d\tau . \end{aligned}$$

For brevity, we consider

106 $$ F \bigl( \tau ,y ( \tau ) \bigr) =f \bigl( \tau ,y ( \tau ) \bigr) \biggl[ - \beta \prime ( \tau ) \ln ( \tau ) + \frac{2-\beta ( \tau ) }{\tau } \biggr] \tau ^{2-\beta ( \tau ) }, $$

and we can write the following:

107 $$ y ( t_{n+1} ) =\frac{1}{\Gamma ( \alpha ) }\sum _{j=2}^{n} \int _{t_{j}}^{t_{j+1}}F \bigl( \tau ,y ( \tau ) \bigr) ( t_{n+1}-\tau ) ^{\alpha -1}\,d\tau . $$

Thus, we have the following scheme:

108 $$\begin{array}{rcl} y^{n+1} & = & \frac{{\left(\Delta t\right)}^{\alpha }}{\Gamma (\alpha +1)}\sum_{j=2}^{n}F\left(t_{j-2},y^{j-2}\right)\left[{\left(n-j+1\right)}^{\alpha }-{\left(n-j\right)}^{\alpha }\right]\\ & & +\frac{{\left(\Delta t\right)}^{\alpha }}{\Gamma (\alpha +2)}\sum_{j=2}^{n}\left[F\left(t_{j-1},y^{j-1}\right)-F\left(t_{j-2},y^{j-2}\right)\right]\\ & & \times \left[\begin{array}{c} {\left(n-j+1\right)}^{\alpha }(n-j+3+2\alpha )\\ -{\left(n-j\right)}^{\alpha }(n-j+3+3\alpha ) \end{array}\right]\\ & & +\frac{{\left(\Delta t\right)}^{\alpha }}{2\Gamma (\alpha +3)}\sum_{j=2}^{n}\left[F\left(t_{j},y^{j}\right)-2F\left(t_{j-1},y^{j-1}\right)+F\left(t_{j-2},y^{j-2}\right)\right]\\ & & \times \left[\begin{array}{c} {\left(n-j+1\right)}^{\alpha }\left[\begin{array}{c} 2{\left(n-j\right)}^{2}+(3\alpha +10)(n-j)\\ +2\alpha^{2}+9\alpha +12 \end{array}\right]\\ -{\left(n-j\right)}^{\alpha }\left[\begin{array}{c} 2{\left(n-j\right)}^{2}+(5\alpha +10)(n-j)\\ +6\alpha^{2}+18\alpha +12 \end{array}\right] \end{array}\right]. \end{array}$$

Replacing the function $G ( t,y ( t ) ) $ with its value, we can present the following scheme for numerical solution of our equation:

109 $$\begin{array}{rl} y^{n+1} & =\frac{{\left(\Delta t\right)}^{\alpha }}{\Gamma (\alpha +1)}\sum_{j=2}^{n}t_{j-2}^{2-\beta (t_{j-2})}\left[-\frac{\beta (t_{j-1})-\beta (t_{j-2})}{\Delta t}\ln t_{j-2}+\frac{2-\beta (t_{j-2})}{t_{j-2}}\right]\\ & \;\times f\left(\begin{array}{c} t_{j-2},y^{j}-\Delta tf(t_{j},y^{j})\\ -\Delta tf(t_{j-1},y^{j}-f(t_{j},y^{j})\Delta t) \end{array}\right)\left[{\left(n-j+1\right)}^{\alpha }-{\left(n-j\right)}^{\alpha }\right]\\ & \;+\frac{{\left(\Delta t\right)}^{\alpha }}{\Gamma (\alpha +2)}\sum_{j=2}^{n}\left[\begin{array}{c} t_{j-1}^{2-\beta (t_{j-1})}[-\frac{\beta (t_{j})-\beta (t_{j-1})}{\Delta t}\ln t_{j-1}+\frac{2-\beta (t_{j-1})}{t_{j-1}}]\\ \times f(t_{j-1},y^{j}-f(t_{j},y^{j})\Delta t)\\ -t_{j-2}^{2-\beta (t_{j-2})}[-\frac{\beta (t_{j-1})-\beta (t_{j-2})}{\Delta t}\ln t_{j-2}+\frac{2-\beta (t_{j-2})}{t_{j-2}}]\\ \times f\left(\begin{array}{c} t_{j-2},y^{j}-\Delta tf(t_{j},y^{j})\\ -\Delta tf(t_{j-1},y^{j}-f(t_{j},y^{j})\Delta t) \end{array}\right) \end{array}\right]\\ & \;\times \left[\begin{array}{c} {\left(n-j+1\right)}^{\alpha }(n-j+3+2\alpha )\\ -{\left(n-j\right)}^{\alpha }(n-j+3+3\alpha ) \end{array}\right]\\ & \;+\frac{{\left(\Delta t\right)}^{\alpha }}{2\Gamma (\alpha +3)}\sum_{j=2}^{n}\left[\begin{array}{c} t_{j}^{2-\beta (t_{j})}[-\frac{\beta (t_{j+1})-\beta (t_{j})}{\Delta t}\ln t_{j}+\frac{2-\beta (t_{j})}{t_{j}}]\\ \times f(t_{j},y^{j})\Delta t\\ -2t_{j-1}^{2-\beta (t_{j-1})}[-\frac{\beta (t_{j})-\beta (t_{j-1})}{\Delta t}\ln t_{j-1}+\frac{2-\beta (t_{j-1})}{t_{j-1}}]\\ \times f(t_{j-1},y^{j}-f(t_{j},y^{j})\Delta t)\\ +t_{j-2}^{2-\beta (t_{j-2})}[-\frac{\beta (t_{j-1})-\beta (t_{j-2})}{\Delta t}\ln t_{j-2}+\frac{2-\beta (t_{j-2})}{t_{j-2}}]\\ \times f\left(\begin{array}{c} t_{j-2},y^{j}-\Delta tf(t_{j},y^{j})\\ -\Delta tf(t_{j-1},y^{j}-f(t_{j},y^{j})\Delta t) \end{array}\right) \end{array}\right]\\ & \;\times \left[\begin{array}{c} {\left(n-j+1\right)}^{\alpha }\left[\begin{array}{c} 2{\left(n-j\right)}^{2}+(3\alpha +10)(n-j)\\ +2\alpha^{2}+9\alpha +12 \end{array}\right]\\ -{\left(n-j\right)}^{\alpha }\left[\begin{array}{c} 2{\left(n-j\right)}^{2}+(5\alpha +10)(n-j)\\ +6\alpha^{2}+18\alpha +12 \end{array}\right] \end{array}\right]. \end{array}$$

## Application to COVID-19 model

In this section, using the suggested numerical scheme, we present its application to solve the mathematical model of COVID-19 with possibility of waves. The numerical scheme will be applied for all cases where the differential operators are with classical differential operators, modern fractional differential operators, and variable orders, although only few examples will be used for numerical simulations. Firstly, we shall use the Caputo–Fabrizio fractional derivative

110 $$\begin{aligned}& {}_{0}^{CF}D_{t}^{\alpha }S = \Lambda - \bigl( \delta ( t ) \bigl( \alpha I^{\ast }+w\beta I_{D}^{\ast }+ \gamma wI_{A}^{\ast }+w \delta _{1}I_{R}^{\ast }+w \delta _{2}I_{T}^{\ast } \bigr) +\gamma _{1}+ \mu _{1} \bigr) S, \\& {}_{0}^{CF}D_{t}^{\alpha }I = \bigl( \delta ( t ) \bigl( \alpha I^{\ast }+w\beta I_{D}^{\ast }+\gamma wI_{A}^{\ast }+w \delta _{1}I_{R}^{\ast }+w \delta _{2}I_{T}^{\ast } \bigr) \bigr) S- ( \varepsilon +\xi +\lambda +\mu _{1} ) I, \\& {}_{0}^{CF}D_{t}^{\alpha }I_{A} = \xi I- ( \theta +\mu + \chi +\mu _{1} ) I_{A}, \\& {}_{0}^{CF}D_{t}^{\alpha }I_{D} = \varepsilon I- ( \eta + \varphi +\mu _{1} ) I_{D}, \\& {}_{0}^{CF}D_{t}^{\alpha }I_{R} = \eta I_{D}+\theta I_{A}- ( v+\xi +\mu _{1} ) I_{R}, \\& {}_{0}^{CF}D_{t}^{\alpha }I_{T} = \mu I_{A}+vI_{R}- ( \sigma +\tau +\mu _{1} ) I_{T}, \\& {}_{0}^{CF}D_{t}^{\alpha }R = \lambda I+ \varphi I_{D}+ \chi I_{A}+\xi I_{R}+\sigma I_{T}- ( \Phi +\mu _{1} ) R, \\& {}_{0}^{CF}D_{t}^{\alpha }D = \tau I_{T}, \\& {}_{0}^{CF}D_{t}^{\alpha }V = \gamma _{1}S+\Phi R-\mu _{1}V. \end{aligned}$$

For simplicity, we rearrange the above equation as follows:

111 $$\begin{aligned}& {}_{0}^{CF}D_{t}^{\alpha }S = S^{\ast } ( t,S,I,I_{A},I_{D},I_{R},I_{T},R,D,V ), \\& {}_{0}^{CF}D_{t}^{\alpha }I = I^{\ast } ( t,S,I,I_{A},I_{D},I_{R},I_{T},R,D,V ), \\& {}_{0}^{CF}D_{t}^{\alpha }I_{A} = I_{A}^{\ast } ( t,S,I,I_{A},I_{D},I_{R},I_{T},R,D,V ), \\& {}_{0}^{CF}D_{t}^{\alpha }I_{D} = I_{D}^{\ast } ( t,S,I,I_{A},I_{D},I_{R},I_{T},R,D,V ), \\& {}_{0}^{CF}D_{t}^{\alpha }I_{R} = I_{R}^{\ast } ( t,S,I,I_{A},I_{D},I_{R},I_{T},R,D,V ), \\& {}_{0}^{CF}D_{t}^{\alpha }I_{T} = I_{T}^{\ast } ( t,S,I,I_{A},I_{D},I_{R},I_{T},R,D,V ), \\& {}_{0}^{CF}D_{t}^{\alpha }R = R^{\ast } ( t,S,I,I_{A},I_{D},I_{R},I_{T},R,D,V ), \\& {}_{0}^{CF}D_{t}^{\alpha }D = D^{\ast } ( t,S,I,I_{A},I_{D},I_{R},I_{T},R,D,V ), \\& {}_{0}^{CF}D_{t}^{\alpha }V = V^{\ast } ( t,S,I,I_{A},I_{D},I_{R},I_{T},R,D,V ) . \end{aligned}$$

Thus, we can have the following scheme for our model:

112 $$S^{n+1}=S^{n}+\frac{1-\alpha }{M(\alpha )}\left[\begin{array}{c} S^{*}\left(\begin{array}{c} t_{n+1},S^{n}+\Delta tS^{n*},I^{n}+\Delta tI^{n*},I_{A}^{n}+\Delta tI_{A}^{n*},\\ I_{D}^{n}+\Delta tI_{D}^{n*},I_{R}^{n}+\Delta tI_{R}^{n*},I_{T}^{n}+\Delta tI_{T}^{n*},\\ R^{n}+\Delta tR^{n*},D^{n}+\Delta tD^{n*},V^{n}+\Delta tV^{n*} \end{array}\right)\\ -S^{*}\left(t_{n},S^{n},I^{n},I_{A}^{n},I_{D}^{n},I_{R}^{n},I_{T}^{n},R^{n},D^{n},V^{n}\right) \end{array}\right]$$

113 Sn+1=+αM(α){2312S∗(tn,Sn,In,IAn,IDn,IRn,ITn,Rn,Dn,Vn)Δt−43S∗(tn,Sn−ΔtSn∗,In−ΔtIn∗,IAn−ΔtIAn∗,IDn−ΔtIDn∗,IRn−ΔtIRn∗,ITn−ΔtITn∗,Rn−ΔtRn∗,Dn−ΔtDn∗,Vn−ΔtVn∗)Δt+512S∗(tn−2,Sn−ΔtSn∗−ΔtS(n−1)∗,In−ΔtIn∗−ΔtI(n−1)∗,IAn−ΔtIAn∗−ΔtIA(n−1)∗,IDn−ΔtIDn∗−ΔtID(n−1)∗,IRn−ΔtIRn∗−ΔtIR(n−1)∗,ITn−ΔtITn∗−ΔtIT(n−1)∗,Rn−ΔtRn∗−ΔtR(n−1)∗,Dn−ΔtDn∗−ΔtD(n−1)∗,Vn−ΔtVn∗−ΔtV(n−1)∗)Δt},In+1=Sn+1−αM(α)[I∗(tn+1,Sn+ΔtSn∗,In+ΔtIn∗,IAn+ΔtIAn∗,IDn+ΔtIDn∗,IRn+ΔtIRn∗,ITn+ΔtITn∗,Rn+ΔtRn∗,Dn+ΔtDn∗,Vn+ΔtVn∗)−I∗(tn,Sn,In,IAn,IDn,IRn,ITn,Rn,Dn,Vn)]

114 In+1=+αM(α){2312I∗(tn,Sn,In,IAn,IDn,IRn,ITn,Rn,Dn,Vn)Δt−43I∗(tn,Sn−ΔtSn∗,In−ΔtIn∗,IAn−ΔtIAn∗,IDn−ΔtIDn∗,IRn−ΔtIRn∗,ITn−ΔtITn∗,Rn−ΔtRn∗,Dn−ΔtDn∗,Vn−ΔtVn∗)Δt+512I∗(tn−2,Sn−ΔtSn∗−ΔtS(n−1)∗,In−ΔtIn∗−ΔtI(n−1)∗,IAn−ΔtIAn∗−ΔtIA(n−1)∗,IDn−ΔtIDn∗−ΔtID(n−1)∗,IRn−ΔtIRn∗−ΔtIR(n−1)∗,ITn−ΔtITn∗−ΔtIT(n−1)∗,Rn−ΔtRn∗−ΔtR(n−1)∗,Dn−ΔtDn∗−ΔtD(n−1)∗,Vn−ΔtVn∗−ΔtV(n−1)∗)Δt},IAn+1=IAn+1−αM(α)[IA∗(tn+1,Sn+ΔtSn∗,In+ΔtIn∗,IAn+ΔtIAn∗,IDn+ΔtIDn∗,IRn+ΔtIRn∗,ITn+ΔtITn∗,Rn+ΔtRn∗,Dn+ΔtDn∗,Vn+ΔtVn∗)−IA∗(tn,Sn,In,IAn,IDn,IRn,ITn,Rn,Dn,Vn)]

115 IAn+1=+αM(α){2312IA∗(tn,Sn,In,IAn,IDn,IRn,ITn,Rn,Dn,Vn)Δt−43IA∗(tn,Sn−ΔtSn∗,In−ΔtIn∗,IAn−ΔtIAn∗,IDn−ΔtIDn∗,IRn−ΔtIRn∗,ITn−ΔtITn∗,Rn−ΔtRn∗,Dn−ΔtDn∗,Vn−ΔtVn∗)Δt+512IA∗(tn−2,Sn−ΔtSn∗−ΔtS(n−1)∗,In−ΔtIn∗−ΔtI(n−1)∗,IAn−ΔtIAn∗−ΔtIA(n−1)∗,IDn−ΔtIDn∗−ΔtID(n−1)∗,IRn−ΔtIRn∗−ΔtIR(n−1)∗,ITn−ΔtITn∗−ΔtIT(n−1)∗,Rn−ΔtRn∗−ΔtR(n−1)∗,Dn−ΔtDn∗−ΔtD(n−1)∗,Vn−ΔtVn∗−ΔtV(n−1)∗)Δt},IDn+1=IDn+1−αM(α)[ID∗(tn+1,Sn+ΔtSn∗,In+ΔtIn∗,IAn+ΔtIAn∗,IDn+ΔtIDn∗,IRn+ΔtIRn∗,ITn+ΔtITn∗,Rn+ΔtRn∗,Dn+ΔtDn∗,Vn+ΔtVn∗)−ID∗(tn,Sn,In,IAn,IDn,IRn,ITn,Rn,Dn,Vn)]

116 IDn+1=+αM(α){2312ID∗(tn,Sn,In,IAn,IDn,IRn,ITn,Rn,Dn,Vn)Δt−43ID∗(tn,Sn−ΔtSn∗,In−ΔtIn∗,IAn−ΔtIAn∗,IDn−ΔtIDn∗,IRn−ΔtIRn∗,ITn−ΔtITn∗,Rn−ΔtRn∗,Dn−ΔtDn∗,Vn−ΔtVn∗)Δt+512ID∗(tn−2,Sn−ΔtSn∗−ΔtS(n−1)∗,In−ΔtIn∗−ΔtI(n−1)∗,IAn−ΔtIAn∗−ΔtIA(n−1)∗,IDn−ΔtIDn∗−ΔtID(n−1)∗,IRn−ΔtIRn∗−ΔtIR(n−1)∗,ITn−ΔtITn∗−ΔtIT(n−1)∗,Rn−ΔtRn∗−ΔtR(n−1)∗,Dn−ΔtDn∗−ΔtD(n−1)∗,Vn−ΔtVn∗−ΔtV(n−1)∗)Δt},IRn+1=IRn+1−αM(α)[IR∗(tn+1,Sn+ΔtSn∗,In+ΔtIn∗,IAn+ΔtIAn∗,IDn+ΔtIDn∗,IRn+ΔtIRn∗,ITn+ΔtITn∗,Rn+ΔtRn∗,Dn+ΔtDn∗,Vn+ΔtVn∗)−IR∗(tn,Sn,In,IAn,IDn,IRn,ITn,Rn,Dn,Vn)]

117 IRn+1=+αM(α){2312IR∗(tn,Sn,In,IAn,IDn,IRn,ITn,Rn,Dn,Vn)Δt−43IR∗(tn,Sn−ΔtSn∗,In−ΔtIn∗,IAn−ΔtIAn∗,IDn−ΔtIDn∗,IRn−ΔtIRn∗,ITn−ΔtITn∗,Rn−ΔtRn∗,Dn−ΔtDn∗,Vn−ΔtVn∗)Δt+512IR∗(tn−2,Sn−ΔtSn∗−ΔtS(n−1)∗,In−ΔtIn∗−ΔtI(n−1)∗,IAn−ΔtIAn∗−ΔtIA(n−1)∗,IDn−ΔtIDn∗−ΔtID(n−1)∗,IRn−ΔtIRn∗−ΔtIR(n−1)∗,ITn−ΔtITn∗−ΔtIT(n−1)∗,Rn−ΔtRn∗−ΔtR(n−1)∗,Dn−ΔtDn∗−ΔtD(n−1)∗,Vn−ΔtVn∗−ΔtV(n−1)∗)Δt},ITn+1=ITn+1−αM(α)[IT∗(tn+1,Sn+ΔtSn∗,In+ΔtIn∗,IAn+ΔtIAn∗,IDn+ΔtIDn∗,IRn+ΔtIRn∗,ITn+ΔtITn∗,Rn+ΔtRn∗,Dn+ΔtDn∗,Vn+ΔtVn∗)−IT∗(tn,Sn,In,IAn,IDn,IRn,ITn,Rn,Dn,Vn)]

118 ITn+1=+αM(α){2312IT∗(tn,Sn,In,IAn,IDn,IRn,ITn,Rn,Dn,Vn)Δt−43IT∗(tn,Sn−ΔtSn∗,In−ΔtIn∗,IAn−ΔtIAn∗,IDn−ΔtIDn∗,IRn−ΔtIRn∗,ITn−ΔtITn∗,Rn−ΔtRn∗,Dn−ΔtDn∗,Vn−ΔtVn∗)Δt+512IT∗(tn−2,Sn−ΔtSn∗−ΔtS(n−1)∗,In−ΔtIn∗−ΔtI(n−1)∗,IAn−ΔtIAn∗−ΔtIA(n−1)∗,IDn−ΔtIDn∗−ΔtID(n−1)∗,IRn−ΔtIRn∗−ΔtIR(n−1)∗,ITn−ΔtITn∗−ΔtIT(n−1)∗,Rn−ΔtRn∗−ΔtR(n−1)∗,Dn−ΔtDn∗−ΔtD(n−1)∗,Vn−ΔtVn∗−ΔtV(n−1)∗)Δt},Rn+1=Rn+1−αM(α)[R∗(tn+1,Sn+ΔtSn∗,In+ΔtIn∗,IAn+ΔtIAn∗,IDn+ΔtIDn∗,IRn+ΔtIRn∗,ITn+ΔtITn∗,Rn+ΔtRn∗,Dn+ΔtDn∗,Vn+ΔtVn∗)−R∗(tn,Sn,In,IAn,IDn,IRn,ITn,Rn,Dn,Vn)]

119 Rn+1=+αM(α){2312R∗(tn,Sn,In,IAn,IDn,IRn,ITn,Rn,Dn,Vn)Δt−43R∗(tn,Sn−ΔtSn∗,In−ΔtIn∗,IAn−ΔtIAn∗,IDn−ΔtIDn∗,IRn−ΔtIRn∗,ITn−ΔtITn∗,Rn−ΔtRn∗,Dn−ΔtDn∗,Vn−ΔtVn∗)Δt+512R∗(tn−2,Sn−ΔtSn∗−ΔtS(n−1)∗,In−ΔtIn∗−ΔtI(n−1)∗,IAn−ΔtIAn∗−ΔtIA(n−1)∗,IDn−ΔtIDn∗−ΔtID(n−1)∗,IRn−ΔtIRn∗−ΔtIR(n−1)∗,ITn−ΔtITn∗−ΔtIT(n−1)∗,Rn−ΔtRn∗−ΔtR(n−1)∗,Dn−ΔtDn∗−ΔtD(n−1)∗,Vn−ΔtVn∗−ΔtV(n−1)∗)Δt},Dn+1=Dn+1−αM(α)[D∗(tn+1,Sn+ΔtSn∗,In+ΔtIn∗,IAn+ΔtIAn∗,IDn+ΔtIDn∗,IRn+ΔtIRn∗,ITn+ΔtITn∗,Rn+ΔtRn∗,Dn+ΔtDn∗,Vn+ΔtVn∗)−D∗(tn,Sn,In,IAn,IDn,IRn,ITn,Rn,Dn,Vn)]

120 Dn+1=+αM(α){2312D∗(tn,Sn,In,IAn,IDn,IRn,ITn,Rn,Dn,Vn)Δt−43D∗(tn,Sn−ΔtSn∗,In−ΔtIn∗,IAn−ΔtIAn∗,IDn−ΔtIDn∗,IRn−ΔtIRn∗,ITn−ΔtITn∗,Rn−ΔtRn∗,Dn−ΔtDn∗,Vn−ΔtVn∗)Δt+512D∗(tn−2,Sn−ΔtSn∗−ΔtS(n−1)∗,In−ΔtIn∗−ΔtI(n−1)∗,IAn−ΔtIAn∗−ΔtIA(n−1)∗,IDn−ΔtIDn∗−ΔtID(n−1)∗,IRn−ΔtIRn∗−ΔtIR(n−1)∗,ITn−ΔtITn∗−ΔtIT(n−1)∗,Rn−ΔtRn∗−ΔtR(n−1)∗,Dn−ΔtDn∗−ΔtD(n−1)∗,Vn−ΔtVn∗−ΔtV(n−1)∗)Δt},Vn+1=Vn+1−αM(α)[V∗(tn+1,Sn+ΔtSn∗,In+ΔtIn∗,IAn+ΔtIAn∗,IDn+ΔtIDn∗,IRn+ΔtIRn∗,ITn+ΔtITn∗,Rn+ΔtRn∗,Dn+ΔtDn∗,Vn+ΔtVn∗)−V∗(tn,Sn,In,IAn,IDn,IRn,ITn,Rn,Dn,Vn)]Vn+1=+αM(α){2312V∗(tn,Sn,In,IAn,IDn,IRn,ITn,Rn,Dn,Vn)Δt−43V∗(tn,Sn−ΔtSn∗,In−ΔtIn∗,IAn−ΔtIAn∗,IDn−ΔtIDn∗,IRn−ΔtIRn∗,ITn−ΔtITn∗,Rn−ΔtRn∗,Dn−ΔtDn∗,Vn−ΔtVn∗)Δt+512V∗(tn−2,Sn−ΔtSn∗−ΔtS(n−1)∗,In−ΔtIn∗−ΔtI(n−1)∗,IAn−ΔtIAn∗−ΔtIA(n−1)∗,IDn−ΔtIDn∗−ΔtID(n−1)∗,IRn−ΔtIRn∗−ΔtIR(n−1)∗,ITn−ΔtITn∗−ΔtIT(n−1)∗,Rn−ΔtRn∗−ΔtR(n−1)∗,Dn−ΔtDn∗−ΔtD(n−1)∗,Vn−ΔtVn∗−ΔtV(n−1)∗)Δt}.

With the Atangana–Baleanu fractional derivative, we can solve numerically our model as follows:

121 Sn+1=1−αAB(α)S∗(tn+1,Sn+ΔtSn∗,In+ΔtIn∗,IAn+ΔtIAn∗,IDn+ΔtIDn∗,IRn+ΔtIRn∗,ITn+ΔtITn∗,Rn+ΔtRn∗,Dn+ΔtDn∗,Vn+ΔtVn∗)+α(Δt)αAB(α)Γ(α+1)×∑j=2nS∗(tj−2,Sj−ΔtSj∗−ΔtS(j−1)∗,Ij−ΔtIj∗−ΔtI(j−1)∗,IAj−ΔtIAj∗−ΔtIA(j−1)∗,IDj−ΔtIDj∗−ΔtID(j−1)∗,IRj−ΔtIRj∗−ΔtIR(j−1)∗,ITj−ΔtITj∗−ΔtIT(j−1)∗,Rj−ΔtRj∗−ΔtR(j−1)∗,Dj−ΔtDj∗−ΔtD(j−1)∗,Vj−ΔtVj∗−ΔtV(j−1)∗)×Π+α(Δt)αAB(α)Γ(α+2)×∑j=2n[S∗(tj−1,Sj−ΔtSj∗,Ij−ΔtIj∗,IAj−ΔtIAj∗,IDj−ΔtIDj∗,IRj−ΔtIRj∗,ITj−ΔtITj∗,Rj−ΔtRj∗,Dj−ΔtDj∗,Vj−ΔtVj∗)−S∗(tj−2,Sj−ΔtSj∗−ΔtS(j−1)∗,Ij−ΔtIj∗−ΔtI(j−1)∗,IAj−ΔtIAj∗−ΔtIA(j−1)∗,IDj−ΔtIDj∗−ΔtID(j−1)∗,IRj−ΔtIRj∗−ΔtIR(j−1)∗,ITj−ΔtITj∗−ΔtIT(j−1)∗,Rj−ΔtRj∗−ΔtR(j−1)∗,Dj−ΔtDj∗−ΔtD(j−1)∗,Vj−ΔtVj∗−ΔtV(j−1)∗)]×Σ+α(Δt)α2AB(α)Γ(α+3)×∑j=2n[S∗(tj,Sj,Ij,IAj,IDj,IRj,ITj,Rj,Dj,Vj)−2S∗(tj−1,Sj−ΔtSj∗,Ij−ΔtIj∗,IAj−ΔtIAj∗,IDj−ΔtIDj∗,IRj−ΔtIRj∗,ITj−ΔtITj∗,Rj−ΔtRj∗,Dj−ΔtDj∗,Vj−ΔtVj∗)+S∗(tj−2,Sj−ΔtSj∗−ΔtS(j−1)∗,Ij−ΔtIj∗−ΔtI(j−1)∗,IAj−ΔtIAj∗−ΔtIA(j−1)∗,IDj−ΔtIDj∗−ΔtID(j−1)∗,IRj−ΔtIRj∗−ΔtIR(j−1)∗,ITj−ΔtITj∗−ΔtIT(j−1)∗,Rj−ΔtRj∗−ΔtR(j−1)∗,Dj−ΔtDj∗−ΔtD(j−1)∗,Vj−ΔtVj∗−ΔtV(j−1)∗)]×Δ,In+1=1−αAB(α)I∗(tn+1,Sn+ΔtSn∗,In+ΔtIn∗,IAn+ΔtIAn∗,IDn+ΔtIDn∗,IRn+ΔtIRn∗,ITn+ΔtITn∗,Rn+ΔtRn∗,Dn+ΔtDn∗,Vn+ΔtVn∗)+α(Δt)αAB(α)Γ(α+1)×∑j=2nI∗(tj−2,Sj−ΔtSj∗−ΔtS(j−1)∗,Ij−ΔtIj∗−ΔtI(j−1)∗,IAj−ΔtIAj∗−ΔtIA(j−1)∗,IDj−ΔtIDj∗−ΔtID(j−1)∗,IRj−ΔtIRj∗−ΔtIR(j−1)∗,ITj−ΔtITj∗−ΔtIT(j−1)∗,Rj−ΔtRj∗−ΔtR(j−1)∗,Dj−ΔtDj∗−ΔtD(j−1)∗,Vj−ΔtVj∗−ΔtV(j−1)∗)×Π+α(Δt)αAB(α)Γ(α+2)×∑j=2n[I∗(tj−1,Sj−ΔtSj∗,Ij−ΔtIj∗,IAj−ΔtIAj∗,IDj−ΔtIDj∗,IRj−ΔtIRj∗,ITj−ΔtITj∗,Rj−ΔtRj∗,Dj−ΔtDj∗,Vj−ΔtVj∗)−I∗(tj−2,Sj−ΔtSj∗−ΔtS(j−1)∗,Ij−ΔtIj∗−ΔtI(j−1)∗,IAj−ΔtIAj∗−ΔtIA(j−1)∗,IDj−ΔtIDj∗−ΔtID(j−1)∗,IRj−ΔtIRj∗−ΔtIR(j−1)∗,ITj−ΔtITj∗−ΔtIT(j−1)∗,Rj−ΔtRj∗−ΔtR(j−1)∗,Dj−ΔtDj∗−ΔtD(j−1)∗,Vj−ΔtVj∗−ΔtV(j−1)∗)]×Σ+α(Δt)α2AB(α)Γ(α+3)×∑j=2n[I∗(tj,Sj,Ij,IAj,IDj,IRj,ITj,Rj,Dj,Vj)−2I∗(tj−1,Sj−ΔtSj∗,Ij−ΔtIj∗,IAj−ΔtIAj∗,IDj−ΔtIDj∗,IRj−ΔtIRj∗,ITj−ΔtITj∗,Rj−ΔtRj∗,Dj−ΔtDj∗,Vj−ΔtVj∗)+I∗(tj−2,Sj−ΔtSj∗−ΔtS(j−1)∗,Ij−ΔtIj∗−ΔtI(j−1)∗,IAj−ΔtIAj∗−ΔtIA(j−1)∗,IDj−ΔtIDj∗−ΔtID(j−1)∗,IRj−ΔtIRj∗−ΔtIR(j−1)∗,ITj−ΔtITj∗−ΔtIT(j−1)∗,Rj−ΔtRj∗−ΔtR(j−1)∗,Dj−ΔtDj∗−ΔtD(j−1)∗,Vj−ΔtVj∗−ΔtV(j−1)∗)]×Δ,IAn+1=1−αAB(α)IA∗(tn+1,Sn+ΔtSn∗,In+ΔtIn∗,IAn+ΔtIAn∗,IDn+ΔtIDn∗,IRn+ΔtIRn∗,ITn+ΔtITn∗,Rn+ΔtRn∗,Dn+ΔtDn∗,Vn+ΔtVn∗)IAn+1=+α(Δt)αAB(α)Γ(α+1)IAn+1=×∑j=2nIA∗(tj−2,Sj−ΔtSj∗−ΔtS(j−1)∗,Ij−ΔtIj∗−ΔtI(j−1)∗,IAj−ΔtIAj∗−ΔtIA(j−1)∗,IDj−ΔtIDj∗−ΔtID(j−1)∗,IRj−ΔtIRj∗−ΔtIR(j−1)∗,ITj−ΔtITj∗−ΔtIT(j−1)∗,Rj−ΔtRj∗−ΔtR(j−1)∗,Dj−ΔtDj∗−ΔtD(j−1)∗,Vj−ΔtVj∗−ΔtV(j−1)∗)×ΠIAn+1=+α(Δt)αAB(α)Γ(α+2)IAn+1=×∑j=2n[IA∗(tj−1,Sj−ΔtSj∗,Ij−ΔtIj∗,IAj−ΔtIAj∗,IDj−ΔtIDj∗,IRj−ΔtIRj∗,ITj−ΔtITj∗,Rj−ΔtRj∗,Dj−ΔtDj∗,Vj−ΔtVj∗)−IA∗(tj−2,Sj−ΔtSj∗−ΔtS(j−1)∗,Ij−ΔtIj∗−ΔtI(j−1)∗,IAj−ΔtIAj∗−ΔtIA(j−1)∗,IDj−ΔtIDj∗−ΔtID(j−1)∗,IRj−ΔtIRj∗−ΔtIR(j−1)∗,ITj−ΔtITj∗−ΔtIT(j−1)∗,Rj−ΔtRj∗−ΔtR(j−1)∗,Dj−ΔtDj∗−ΔtD(j−1)∗,Vj−ΔtVj∗−ΔtV(j−1)∗)]×ΣIAn+1=+α(Δt)α2AB(α)Γ(α+3)IAn+1=×∑j=2n[IA∗(tj,Sj,Ij,IAj,IDj,IRj,ITj,Rj,Dj,Vj)−2IA∗(tj−1,Sj−ΔtSj∗,Ij−ΔtIj∗,IAj−ΔtIAj∗,IDj−ΔtIDj∗,IRj−ΔtIRj∗,ITj−ΔtITj∗,Rj−ΔtRj∗,Dj−ΔtDj∗,Vj−ΔtVj∗)+IA∗(tj−2,Sj−ΔtSj∗−ΔtS(j−1)∗,Ij−ΔtIj∗−ΔtI(j−1)∗,IAj−ΔtIAj∗−ΔtIA(j−1)∗,IDj−ΔtIDj∗−ΔtID(j−1)∗,IRj−ΔtIRj∗−ΔtIR(j−1)∗,ITj−ΔtITj∗−ΔtIT(j−1)∗,Rj−ΔtRj∗−ΔtR(j−1)∗,Dj−ΔtDj∗−ΔtD(j−1)∗,Vj−ΔtVj∗−ΔtV(j−1)∗)]×Δ,IDn+1=1−αAB(α)ID∗(tn+1,Sn+ΔtSn∗,In+ΔtIn∗,IAn+ΔtIAn∗,IDn+ΔtIDn∗,IRn+ΔtIRn∗,ITn+ΔtITn∗,Rn+ΔtRn∗,Dn+ΔtDn∗,Vn+ΔtVn∗)IDn+1=+α(Δt)αAB(α)Γ(α+1)IDn+1=×∑j=2nID∗(tj−2,Sj−ΔtSj∗−ΔtS(j−1)∗,Ij−ΔtIj∗−ΔtI(j−1)∗,IAj−ΔtIAj∗−ΔtIA(j−1)∗,IDj−ΔtIDj∗−ΔtID(j−1)∗,IRj−ΔtIRj∗−ΔtIR(j−1)∗,ITj−ΔtITj∗−ΔtIT(j−1)∗,Rj−ΔtRj∗−ΔtR(j−1)∗,Dj−ΔtDj∗−ΔtD(j−1)∗,Vj−ΔtVj∗−ΔtV(j−1)∗)×ΠIDn+1=+α(Δt)αAB(α)Γ(α+2)IDn+1=×∑j=2n[ID∗(tj−1,Sj−ΔtSj∗,Ij−ΔtIj∗,IAj−ΔtIAj∗,IDj−ΔtIDj∗,IRj−ΔtIRj∗,ITj−ΔtITj∗,Rj−ΔtRj∗,Dj−ΔtDj∗,Vj−ΔtVj∗)−ID∗(tj−2,Sj−ΔtSj∗−ΔtS(j−1)∗,Ij−ΔtIj∗−ΔtI(j−1)∗,IAj−ΔtIAj∗−ΔtIA(j−1)∗,IDj−ΔtIDj∗−ΔtID(j−1)∗,IRj−ΔtIRj∗−ΔtIR(j−1)∗,ITj−ΔtITj∗−ΔtIT(j−1)∗,Rj−ΔtRj∗−ΔtR(j−1)∗,Dj−ΔtDj∗−ΔtD(j−1)∗,Vj−ΔtVj∗−ΔtV(j−1)∗)]×ΣIDn+1=+α(Δt)α2AB(α)Γ(α+3)IDn+1=×∑j=2n[ID∗(tj,Sj,Ij,IAj,IDj,IRj,ITj,Rj,Dj,Vj)−2ID∗(tj−1,Sj−ΔtSj∗,Ij−ΔtIj∗,IAj−ΔtIAj∗,IDj−ΔtIDj∗,IRj−ΔtIRj∗,ITj−ΔtITj∗,Rj−ΔtRj∗,Dj−ΔtDj∗,Vj−ΔtVj∗)+ID∗(tj−2,Sj−ΔtSj∗−ΔtS(j−1)∗,Ij−ΔtIj∗−ΔtI(j−1)∗,IAj−ΔtIAj∗−ΔtIA(j−1)∗,IDj−ΔtIDj∗−ΔtID(j−1)∗,IRj−ΔtIRj∗−ΔtIR(j−1)∗,ITj−ΔtITj∗−ΔtIT(j−1)∗,Rj−ΔtRj∗−ΔtR(j−1)∗,Dj−ΔtDj∗−ΔtD(j−1)∗,Vj−ΔtVj∗−ΔtV(j−1)∗)]×Δ,IRn+1=1−αAB(α)IR∗(tn+1,Sn+ΔtSn∗,In+ΔtIn∗,IAn+ΔtIAn∗,IDn+ΔtIDn∗,IRn+ΔtIRn∗,ITn+ΔtITn∗,Rn+ΔtRn∗,Dn+ΔtDn∗,Vn+ΔtVn∗)IRn+1=+α(Δt)αAB(α)Γ(α+1)IRn+1=×∑j=2nIR∗(tj−2,Sj−ΔtSj∗−ΔtS(j−1)∗,Ij−ΔtIj∗−ΔtI(j−1)∗,IAj−ΔtIAj∗−ΔtIA(j−1)∗,IDj−ΔtIDj∗−ΔtID(j−1)∗,IRj−ΔtIRj∗−ΔtIR(j−1)∗,ITj−ΔtITj∗−ΔtIT(j−1)∗,Rj−ΔtRj∗−ΔtR(j−1)∗,Dj−ΔtDj∗−ΔtD(j−1)∗,Vj−ΔtVj∗−ΔtV(j−1)∗)×ΠIRn+1=+α(Δt)αAB(α)Γ(α+2)IRn+1=×∑j=2n[IR∗(tj−1,Sj−ΔtSj∗,Ij−ΔtIj∗,IAj−ΔtIAj∗,IDj−ΔtIDj∗,IRj−ΔtIRj∗,ITj−ΔtITj∗,Rj−ΔtRj∗,Dj−ΔtDj∗,Vj−ΔtVj∗)−IR∗(tj−2,Sj−ΔtSj∗−ΔtS(j−1)∗,Ij−ΔtIj∗−ΔtI(j−1)∗,IAj−ΔtIAj∗−ΔtIA(j−1)∗,IDj−ΔtIDj∗−ΔtID(j−1)∗,IRj−ΔtIRj∗−ΔtIR(j−1)∗,ITj−ΔtITj∗−ΔtIT(j−1)∗,Rj−ΔtRj∗−ΔtR(j−1)∗,Dj−ΔtDj∗−ΔtD(j−1)∗,Vj−ΔtVj∗−ΔtV(j−1)∗)]×ΣIRn+1=+α(Δt)α2AB(α)Γ(α+3)IRn+1=×∑j=2n[IR∗(tj,Sj,Ij,IAj,IDj,IRj,ITj,Rj,Dj,Vj)−2IR∗(tj−1,Sj−ΔtSj∗,Ij−ΔtIj∗,IAj−ΔtIAj∗,IDj−ΔtIDj∗,IRj−ΔtIRj∗,ITj−ΔtITj∗,Rj−ΔtRj∗,Dj−ΔtDj∗,Vj−ΔtVj∗)+IR∗(tj−2,Sj−ΔtSj∗−ΔtS(j−1)∗,Ij−ΔtIj∗−ΔtI(j−1)∗,IAj−ΔtIAj∗−ΔtIA(j−1)∗,IDj−ΔtIDj∗−ΔtID(j−1)∗,IRj−ΔtIRj∗−ΔtIR(j−1)∗,ITj−ΔtITj∗−ΔtIT(j−1)∗,Rj−ΔtRj∗−ΔtR(j−1)∗,Dj−ΔtDj∗−ΔtD(j−1)∗,Vj−ΔtVj∗−ΔtV(j−1)∗)]×Δ,ITn+1=1−αAB(α)IT∗(tn+1,Sn+ΔtSn∗,In+ΔtIn∗,IAn+ΔtIAn∗,IDn+ΔtIDn∗,IRn+ΔtIRn∗,ITn+ΔtITn∗,Rn+ΔtRn∗,Dn+ΔtDn∗,Vn+ΔtVn∗)ITn+1=+α(Δt)αAB(α)Γ(α+1)ITn+1=×∑j=2nIT∗(tj−2,Sj−ΔtSj∗−ΔtS(j−1)∗,Ij−ΔtIj∗−ΔtI(j−1)∗,IAj−ΔtIAj∗−ΔtIA(j−1)∗,IDj−ΔtIDj∗−ΔtID(j−1)∗,IRj−ΔtIRj∗−ΔtIR(j−1)∗,ITj−ΔtITj∗−ΔtIT(j−1)∗,Rj−ΔtRj∗−ΔtR(j−1)∗,Dj−ΔtDj∗−ΔtD(j−1)∗,Vj−ΔtVj∗−ΔtV(j−1)∗)×ΠITn+1=+α(Δt)αAB(α)Γ(α+2)ITn+1=×∑j=2n[IT∗(tj−1,Sj−ΔtSj∗,Ij−ΔtIj∗,IAj−ΔtIAj∗,IDj−ΔtIDj∗,IRj−ΔtIRj∗,ITj−ΔtITj∗,Rj−ΔtRj∗,Dj−ΔtDj∗,Vj−ΔtVj∗)−IT∗(tj−2,Sj−ΔtSj∗−ΔtS(j−1)∗,Ij−ΔtIj∗−ΔtI(j−1)∗,IAj−ΔtIAj∗−ΔtIA(j−1)∗,IDj−ΔtIDj∗−ΔtID(j−1)∗,IRj−ΔtIRj∗−ΔtIR(j−1)∗,ITj−ΔtITj∗−ΔtIT(j−1)∗,Rj−ΔtRj∗−ΔtR(j−1)∗,Dj−ΔtDj∗−ΔtD(j−1)∗,Vj−ΔtVj∗−ΔtV(j−1)∗)]×ΣITn+1=+α(Δt)α2AB(α)Γ(α+3)ITn+1=×∑j=2n[IT∗(tj,Sj,Ij,IAj,IDj,IRj,ITj,Rj,Dj,Vj)−2IT∗(tj−1,Sj−ΔtSj∗,Ij−ΔtIj∗,IAj−ΔtIAj∗,IDj−ΔtIDj∗,IRj−ΔtIRj∗,ITj−ΔtITj∗,Rj−ΔtRj∗,Dj−ΔtDj∗,Vj−ΔtVj∗)+IT∗(tj−2,Sj−ΔtSj∗−ΔtS(j−1)∗,Ij−ΔtIj∗−ΔtI(j−1)∗,IAj−ΔtIAj∗−ΔtIA(j−1)∗,IDj−ΔtIDj∗−ΔtID(j−1)∗,IRj−ΔtIRj∗−ΔtIR(j−1)∗,ITj−ΔtITj∗−ΔtIT(j−1)∗,Rj−ΔtRj∗−ΔtR(j−1)∗,Dj−ΔtDj∗−ΔtD(j−1)∗,Vj−ΔtVj∗−ΔtV(j−1)∗)]×Δ,Rn+1=1−αAB(α)R∗(tn+1,Sn+ΔtSn∗,In+ΔtIn∗,IAn+ΔtIAn∗,IDn+ΔtIDn∗,IRn+ΔtIRn∗,ITn+ΔtITn∗,Rn+ΔtRn∗,Dn+ΔtDn∗,Vn+ΔtVn∗)Rn+1=+α(Δt)αAB(α)Γ(α+1)Rn+1=×∑j=2nR∗(tj−2,Sj−ΔtSj∗−ΔtS(j−1)∗,Ij−ΔtIj∗−ΔtI(j−1)∗,IAj−ΔtIAj∗−ΔtIA(j−1)∗,IDj−ΔtIDj∗−ΔtID(j−1)∗,IRj−ΔtIRj∗−ΔtIR(j−1)∗,ITj−ΔtITj∗−ΔtIT(j−1)∗,Rj−ΔtRj∗−ΔtR(j−1)∗,Dj−ΔtDj∗−ΔtD(j−1)∗,Vj−ΔtVj∗−ΔtV(j−1)∗)×ΠRn+1=+α(Δt)αAB(α)Γ(α+2)Rn+1=×∑j=2n[R∗(tj−1,Sj−ΔtSj∗,Ij−ΔtIj∗,IAj−ΔtIAj∗,IDj−ΔtIDj∗,IRj−ΔtIRj∗,ITj−ΔtITj∗,Rj−ΔtRj∗,Dj−ΔtDj∗,Vj−ΔtVj∗)−R∗(tj−2,Sj−ΔtSj∗−ΔtS(j−1)∗,Ij−ΔtIj∗−ΔtI(j−1)∗,IAj−ΔtIAj∗−ΔtIA(j−1)∗,IDj−ΔtIDj∗−ΔtID(j−1)∗,IRj−ΔtIRj∗−ΔtIR(j−1)∗,ITj−ΔtITj∗−ΔtIT(j−1)∗,Rj−ΔtRj∗−ΔtR(j−1)∗,Dj−ΔtDj∗−ΔtD(j−1)∗,Vj−ΔtVj∗−ΔtV(j−1)∗)]×ΣRn+1=+α(Δt)α2AB(α)Γ(α+3)Rn+1=×∑j=2n[R∗(tj,Sj,Ij,IAj,IDj,IRj,ITj,Rj,Dj,Vj)−2R∗(tj−1,Sj−ΔtSj∗,Ij−ΔtIj∗,IAj−ΔtIAj∗,IDj−ΔtIDj∗,IRj−ΔtIRj∗,ITj−ΔtITj∗,Rj−ΔtRj∗,Dj−ΔtDj∗,Vj−ΔtVj∗)+R∗(tj−2,Sj−ΔtSj∗−ΔtS(j−1)∗,Ij−ΔtIj∗−ΔtI(j−1)∗,IAj−ΔtIAj∗−ΔtIA(j−1)∗,IDj−ΔtIDj∗−ΔtID(j−1)∗,IRj−ΔtIRj∗−ΔtIR(j−1)∗,ITj−ΔtITj∗−ΔtIT(j−1)∗,Rj−ΔtRj∗−ΔtR(j−1)∗,Dj−ΔtDj∗−ΔtD(j−1)∗,Vj−ΔtVj∗−ΔtV(j−1)∗)]×Δ,Dn+1=1−αAB(α)D∗(tn+1,Sn+ΔtSn∗,In+ΔtIn∗,IAn+ΔtIAn∗,IDn+ΔtIDn∗,IRn+ΔtIRn∗,ITn+ΔtITn∗,Rn+ΔtRn∗,Dn+ΔtDn∗,Vn+ΔtVn∗)Dn+1=+α(Δt)αAB(α)Γ(α+1)Dn+1=×∑j=2nD∗(tj−2,Sj−ΔtSj∗−ΔtS(j−1)∗,Ij−ΔtIj∗−ΔtI(j−1)∗,IAj−ΔtIAj∗−ΔtIA(j−1)∗,IDj−ΔtIDj∗−ΔtID(j−1)∗,IRj−ΔtIRj∗−ΔtIR(j−1)∗,ITj−ΔtITj∗−ΔtIT(j−1)∗,Rj−ΔtRj∗−ΔtR(j−1)∗,Dj−ΔtDj∗−ΔtD(j−1)∗,Vj−ΔtVj∗−ΔtV(j−1)∗)×ΠDn+1=+α(Δt)αAB(α)Γ(α+2)Dn+1=×∑j=2n[D∗(tj−1,Sj−ΔtSj∗,Ij−ΔtIj∗,IAj−ΔtIAj∗,IDj−ΔtIDj∗,IRj−ΔtIRj∗,ITj−ΔtITj∗,Rj−ΔtRj∗,Dj−ΔtDj∗,Vj−ΔtVj∗)−D∗(tj−2,Sj−ΔtSj∗−ΔtS(j−1)∗,Ij−ΔtIj∗−ΔtI(j−1)∗,IAj−ΔtIAj∗−ΔtIA(j−1)∗,IDj−ΔtIDj∗−ΔtID(j−1)∗,IRj−ΔtIRj∗−ΔtIR(j−1)∗,ITj−ΔtITj∗−ΔtIT(j−1)∗,Rj−ΔtRj∗−ΔtR(j−1)∗,Dj−ΔtDj∗−ΔtD(j−1)∗,Vj−ΔtVj∗−ΔtV(j−1)∗)]×ΣDn+1=+α(Δt)α2AB(α)Γ(α+3)Dn+1=×∑j=2n[D∗(tj,Sj,Ij,IAj,IDj,IRj,ITj,Rj,Dj,Vj)−2D∗(tj−1,Sj−ΔtSj∗,Ij−ΔtIj∗,IAj−ΔtIAj∗,IDj−ΔtIDj∗,IRj−ΔtIRj∗,ITj−ΔtITj∗,Rj−ΔtRj∗,Dj−ΔtDj∗,Vj−ΔtVj∗)+D∗(tj−2,Sj−ΔtSj∗−ΔtS(j−1)∗,Ij−ΔtIj∗−ΔtI(j−1)∗,IAj−ΔtIAj∗−ΔtIA(j−1)∗,IDj−ΔtIDj∗−ΔtID(j−1)∗,IRj−ΔtIRj∗−ΔtIR(j−1)∗,ITj−ΔtITj∗−ΔtIT(j−1)∗,Rj−ΔtRj∗−ΔtR(j−1)∗,Dj−ΔtDj∗−ΔtD(j−1)∗,Vj−ΔtVj∗−ΔtV(j−1)∗)]×Δ,Vn+1=1−αAB(α)V∗(tn+1,Sn+ΔtSn∗,In+ΔtIn∗,IAn+ΔtIAn∗,IDn+ΔtIDn∗,IRn+ΔtIRn∗,ITn+ΔtITn∗,Rn+ΔtRn∗,Dn+ΔtDn∗,Vn+ΔtVn∗)Vn+1=+α(Δt)αAB(α)Γ(α+1)Vn+1=×∑j=2nV∗(tj−2,Sj−ΔtSj∗−ΔtS(j−1)∗,Ij−ΔtIj∗−ΔtI(j−1)∗,IAj−ΔtIAj∗−ΔtIA(j−1)∗,IDj−ΔtIDj∗−ΔtID(j−1)∗,IRj−ΔtIRj∗−ΔtIR(j−1)∗,ITj−ΔtITj∗−ΔtIT(j−1)∗,Rj−ΔtRj∗−ΔtR(j−1)∗,Dj−ΔtDj∗−ΔtD(j−1)∗,Vj−ΔtVj∗−ΔtV(j−1)∗)×ΠVn+1=+α(Δt)αAB(α)Γ(α+2)Vn+1=×∑j=2n[V∗(tj−1,Sj−ΔtSj∗,Ij−ΔtIj∗,IAj−ΔtIAj∗,IDj−ΔtIDj∗,IRj−ΔtIRj∗,ITj−ΔtITj∗,Rj−ΔtRj∗,Dj−ΔtDj∗,Vj−ΔtVj∗)−V∗(tj−2,Sj−ΔtSj∗−ΔtS(j−1)∗,Ij−ΔtIj∗−ΔtI(j−1)∗,IAj−ΔtIAj∗−ΔtIA(j−1)∗,IDj−ΔtIDj∗−ΔtID(j−1)∗,IRj−ΔtIRj∗−ΔtIR(j−1)∗,ITj−ΔtITj∗−ΔtIT(j−1)∗,Rj−ΔtRj∗−ΔtR(j−1)∗,Dj−ΔtDj∗−ΔtD(j−1)∗,Vj−ΔtVj∗−ΔtV(j−1)∗)]×ΣVn+1=+α(Δt)α2AB(α)Γ(α+3)Vn+1=×∑j=2n[V∗(tj,Sj,Ij,IAj,IDj,IRj,ITj,Rj,Dj,Vj)−2V∗(tj−1,Sj−ΔtSj∗,Ij−ΔtIj∗,IAj−ΔtIAj∗,IDj−ΔtIDj∗,IRj−ΔtIRj∗,ITj−ΔtITj∗,Rj−ΔtRj∗,Dj−ΔtDj∗,Vj−ΔtVj∗)+V∗(tj−2,Sj−ΔtSj∗−ΔtS(j−1)∗,Ij−ΔtIj∗−ΔtI(j−1)∗,IAj−ΔtIAj∗−ΔtIA(j−1)∗,IDj−ΔtIDj∗−ΔtID(j−1)∗,IRj−ΔtIRj∗−ΔtIR(j−1)∗,ITj−ΔtITj∗−ΔtIT(j−1)∗,Rj−ΔtRj∗−ΔtR(j−1)∗,Dj−ΔtDj∗−ΔtD(j−1)∗,Vj−ΔtVj∗−ΔtV(j−1)∗)]×Δ,

where

122 $$\begin{array}{c} \Delta =\left[\begin{array}{c} {\left(n-j+1\right)}^{\alpha }\left[\begin{array}{c} 2{\left(n-j\right)}^{2}+(3\alpha +10)(n-j)\\ +2\alpha^{2}+9\alpha +12 \end{array}\right]\\ -{\left(n-j\right)}^{\alpha }\left[\begin{array}{c} 2{\left(n-j\right)}^{2}+(5\alpha +10)(n-j)\\ +6\alpha^{2}+18\alpha +12 \end{array}\right] \end{array}\right],\\ \Sigma =\left[\begin{array}{c} {\left(n-j+1\right)}^{\alpha }(n-j+3+2\alpha )\\ -{\left(n-j\right)}^{\alpha }(n-j+3+3\alpha ) \end{array}\right],\;\;\Pi =\left[{\left(n-j+1\right)}^{\alpha }-{\left(n-j\right)}^{\alpha }\right]. \end{array}$$

With the Caputo fractional derivative, we can obtain the following:

123 Sn+1=(Δt)αΓ(α+1)∑j=2nS∗(tj−2,Sj−ΔtSj∗−ΔtS(j−1)∗,Ij−ΔtIj∗−ΔtI(j−1)∗,IAj−ΔtIAj∗−ΔtIA(j−1)∗,IDj−ΔtIDj∗−ΔtID(j−1)∗,IRj−ΔtIRj∗−ΔtIR(j−1)∗,ITj−ΔtITj∗−ΔtIT(j−1)∗,Rj−ΔtRj∗−ΔtR(j−1)∗,Dj−ΔtDj∗−ΔtD(j−1)∗,Vj−ΔtVj∗−ΔtV(j−1)∗)×Π+(Δt)αΓ(α+2)∑j=2n[S∗(tj−1,Sj−ΔtSj∗,Ij−ΔtIj∗,IAj−ΔtIAj∗,IDj−ΔtIDj∗,IRj−ΔtIRj∗,ITj−ΔtITj∗,Rj−ΔtRj∗,Dj−ΔtDj∗,Vj−ΔtVj∗)−S∗(tj−2,Sj−ΔtSj∗−ΔtS(j−1)∗,Ij−ΔtIj∗−ΔtI(j−1)∗,IAj−ΔtIAj∗−ΔtIA(j−1)∗,IDj−ΔtIDj∗−ΔtID(j−1)∗,IRj−ΔtIRj∗−ΔtIR(j−1)∗,ITj−ΔtITj∗−ΔtIT(j−1)∗,Rj−ΔtRj∗−ΔtR(j−1)∗,Dj−ΔtDj∗−ΔtD(j−1)∗,Vj−ΔtVj∗−ΔtV(j−1)∗)]×Σ+(Δt)α2Γ(α+3)∑j=2n[S∗(tj,Sj,Ij,IAj,IDj,IRj,ITj,Rj,Dj,Vj)−2S∗(tj−1,Sj−ΔtSj∗,Ij−ΔtIj∗,IAj−ΔtIAj∗,IDj−ΔtIDj∗,IRj−ΔtIRj∗,ITj−ΔtITj∗,Rj−ΔtRj∗,Dj−ΔtDj∗,Vj−ΔtVj∗)+S∗(tj−2,Sj−ΔtSj∗−ΔtS(j−1)∗,Ij−ΔtIj∗−ΔtI(j−1)∗,IAj−ΔtIAj∗−ΔtIA(j−1)∗,IDj−ΔtIDj∗−ΔtID(j−1)∗,IRj−ΔtIRj∗−ΔtIR(j−1)∗,ITj−ΔtITj∗−ΔtIT(j−1)∗,Rj−ΔtRj∗−ΔtR(j−1)∗,Dj−ΔtDj∗−ΔtD(j−1)∗,Vj−ΔtVj∗−ΔtV(j−1)∗)]×Δ,In+1=(Δt)αΓ(α+1)∑j=2nI∗(tj−2,Sj−ΔtSj∗−ΔtS(j−1)∗,Ij−ΔtIj∗−ΔtI(j−1)∗,IAj−ΔtIAj∗−ΔtIA(j−1)∗,IDj−ΔtIDj∗−ΔtID(j−1)∗,IRj−ΔtIRj∗−ΔtIR(j−1)∗,ITj−ΔtITj∗−ΔtIT(j−1)∗,Rj−ΔtRj∗−ΔtR(j−1)∗,Dj−ΔtDj∗−ΔtD(j−1)∗,Vj−ΔtVj∗−ΔtV(j−1)∗)×ΠIn+1=+(Δt)αΓ(α+2)∑j=2n[I∗(tj−1,Sj−ΔtSj∗,Ij−ΔtIj∗,IAj−ΔtIAj∗,IDj−ΔtIDj∗,IRj−ΔtIRj∗,ITj−ΔtITj∗,Rj−ΔtRj∗,Dj−ΔtDj∗,Vj−ΔtVj∗)−I∗(tj−2,Sj−ΔtSj∗−ΔtS(j−1)∗,Ij−ΔtIj∗−ΔtI(j−1)∗,IAj−ΔtIAj∗−ΔtIA(j−1)∗,IDj−ΔtIDj∗−ΔtID(j−1)∗,IRj−ΔtIRj∗−ΔtIR(j−1)∗,ITj−ΔtITj∗−ΔtIT(j−1)∗,Rj−ΔtRj∗−ΔtR(j−1)∗,Dj−ΔtDj∗−ΔtD(j−1)∗,Vj−ΔtVj∗−ΔtV(j−1)∗)]×ΣIn+1=+(Δt)α2Γ(α+3)∑j=2n[I∗(tj,Sj,Ij,IAj,IDj,IRj,ITj,Rj,Dj,Vj)−2I∗(tj−1,Sj−ΔtSj∗,Ij−ΔtIj∗,IAj−ΔtIAj∗,IDj−ΔtIDj∗,IRj−ΔtIRj∗,ITj−ΔtITj∗,Rj−ΔtRj∗,Dj−ΔtDj∗,Vj−ΔtVj∗)+I∗(tj−2,Sj−ΔtSj∗−ΔtS(j−1)∗,Ij−ΔtIj∗−ΔtI(j−1)∗,IAj−ΔtIAj∗−ΔtIA(j−1)∗,IDj−ΔtIDj∗−ΔtID(j−1)∗,IRj−ΔtIRj∗−ΔtIR(j−1)∗,ITj−ΔtITj∗−ΔtIT(j−1)∗,Rj−ΔtRj∗−ΔtR(j−1)∗,Dj−ΔtDj∗−ΔtD(j−1)∗,Vj−ΔtVj∗−ΔtV(j−1)∗)]In+1=×Δ,IAn+1=(Δt)αΓ(α+1)∑j=2nIA∗(tj−2,Sj−ΔtSj∗−ΔtS(j−1)∗,Ij−ΔtIj∗−ΔtI(j−1)∗,IAj−ΔtIAj∗−ΔtIA(j−1)∗,IDj−ΔtIDj∗−ΔtID(j−1)∗,IRj−ΔtIRj∗−ΔtIR(j−1)∗,ITj−ΔtITj∗−ΔtIT(j−1)∗,Rj−ΔtRj∗−ΔtR(j−1)∗,Dj−ΔtDj∗−ΔtD(j−1)∗,Vj−ΔtVj∗−ΔtV(j−1)∗)×Π+(Δt)αΓ(α+2)∑j=2n[IA∗(tj−1,Sj−ΔtSj∗,Ij−ΔtIj∗,IAj−ΔtIAj∗,IDj−ΔtIDj∗,IRj−ΔtIRj∗,ITj−ΔtITj∗,Rj−ΔtRj∗,Dj−ΔtDj∗,Vj−ΔtVj∗)−IA∗(tj−2,Sj−ΔtSj∗−ΔtS(j−1)∗,Ij−ΔtIj∗−ΔtI(j−1)∗,IAj−ΔtIAj∗−ΔtIA(j−1)∗,IDj−ΔtIDj∗−ΔtID(j−1)∗,IRj−ΔtIRj∗−ΔtIR(j−1)∗,ITj−ΔtITj∗−ΔtIT(j−1)∗,Rj−ΔtRj∗−ΔtR(j−1)∗,Dj−ΔtDj∗−ΔtD(j−1)∗,Vj−ΔtVj∗−ΔtV(j−1)∗)]×Σ+(Δt)α2Γ(α+3)∑j=2n[IA∗(tj,Sj,Ij,IAj,IDj,IRj,ITj,Rj,Dj,Vj)−2IA∗(tj−1,Sj−ΔtSj∗,Ij−ΔtIj∗,IAj−ΔtIAj∗,IDj−ΔtIDj∗,IRj−ΔtIRj∗,ITj−ΔtITj∗,Rj−ΔtRj∗,Dj−ΔtDj∗,Vj−ΔtVj∗)+IA∗(tj−2,Sj−ΔtSj∗−ΔtS(j−1)∗,Ij−ΔtIj∗−ΔtI(j−1)∗,IAj−ΔtIAj∗−ΔtIA(j−1)∗,IDj−ΔtIDj∗−ΔtID(j−1)∗,IRj−ΔtIRj∗−ΔtIR(j−1)∗,ITj−ΔtITj∗−ΔtIT(j−1)∗,Rj−ΔtRj∗−ΔtR(j−1)∗,Dj−ΔtDj∗−ΔtD(j−1)∗,Vj−ΔtVj∗−ΔtV(j−1)∗)]×Δ,IDn+1=(Δt)αΓ(α+1)∑j=2nID∗(tj−2,Sj−ΔtSj∗−ΔtS(j−1)∗,Ij−ΔtIj∗−ΔtI(j−1)∗,IAj−ΔtIAj∗−ΔtIA(j−1)∗,IDj−ΔtIDj∗−ΔtID(j−1)∗,IRj−ΔtIRj∗−ΔtIR(j−1)∗,ITj−ΔtITj∗−ΔtIT(j−1)∗,Rj−ΔtRj∗−ΔtR(j−1)∗,Dj−ΔtDj∗−ΔtD(j−1)∗,Vj−ΔtVj∗−ΔtV(j−1)∗)×ΠIDn+1=+(Δt)αΓ(α+2)IDn+1=×∑j=2n[ID∗(tj−1,Sj−ΔtSj∗,Ij−ΔtIj∗,IAj−ΔtIAj∗,IDj−ΔtIDj∗,IRj−ΔtIRj∗,ITj−ΔtITj∗,Rj−ΔtRj∗,Dj−ΔtDj∗,Vj−ΔtVj∗)−ID∗(tj−2,Sj−ΔtSj∗−ΔtS(j−1)∗,Ij−ΔtIj∗−ΔtI(j−1)∗,IAj−ΔtIAj∗−ΔtIA(j−1)∗,IDj−ΔtIDj∗−ΔtID(j−1)∗,IRj−ΔtIRj∗−ΔtIR(j−1)∗,ITj−ΔtITj∗−ΔtIT(j−1)∗,Rj−ΔtRj∗−ΔtR(j−1)∗,Dj−ΔtDj∗−ΔtD(j−1)∗,Vj−ΔtVj∗−ΔtV(j−1)∗)]×ΣIDn+1=+(Δt)α2Γ(α+3)IDn+1=×∑j=2n[ID∗(tj,Sj,Ij,IAj,IDj,IRj,ITj,Rj,Dj,Vj)−2ID∗(tj−1,Sj−ΔtSj∗,Ij−ΔtIj∗,IAj−ΔtIAj∗,IDj−ΔtIDj∗,IRj−ΔtIRj∗,ITj−ΔtITj∗,Rj−ΔtRj∗,Dj−ΔtDj∗,Vj−ΔtVj∗)+ID∗(tj−2,Sj−ΔtSj∗−ΔtS(j−1)∗,Ij−ΔtIj∗−ΔtI(j−1)∗,IAj−ΔtIAj∗−ΔtIA(j−1)∗,IDj−ΔtIDj∗−ΔtID(j−1)∗,IRj−ΔtIRj∗−ΔtIR(j−1)∗,ITj−ΔtITj∗−ΔtIT(j−1)∗,Rj−ΔtRj∗−ΔtR(j−1)∗,Dj−ΔtDj∗−ΔtD(j−1)∗,Vj−ΔtVj∗−ΔtV(j−1)∗)]IDn+1=×Δ,IRn+1=(Δt)αΓ(α+1)∑j=2nIR∗(tj−2,Sj−ΔtSj∗−ΔtS(j−1)∗,Ij−ΔtIj∗−ΔtI(j−1)∗,IAj−ΔtIAj∗−ΔtIA(j−1)∗,IDj−ΔtIDj∗−ΔtID(j−1)∗,IRj−ΔtIRj∗−ΔtIR(j−1)∗,ITj−ΔtITj∗−ΔtIT(j−1)∗,Rj−ΔtRj∗−ΔtR(j−1)∗,Dj−ΔtDj∗−ΔtD(j−1)∗,Vj−ΔtVj∗−ΔtV(j−1)∗)×ΠIRn+1=+(Δt)αΓ(α+2)∑j=2n[IR∗(tj−1,Sj−ΔtSj∗,Ij−ΔtIj∗,IAj−ΔtIAj∗,IDj−ΔtIDj∗,IRj−ΔtIRj∗,ITj−ΔtITj∗,Rj−ΔtRj∗,Dj−ΔtDj∗,Vj−ΔtVj∗)−IR∗(tj−2,Sj−ΔtSj∗−ΔtS(j−1)∗,Ij−ΔtIj∗−ΔtI(j−1)∗,IAj−ΔtIAj∗−ΔtIA(j−1)∗,IDj−ΔtIDj∗−ΔtID(j−1)∗,IRj−ΔtIRj∗−ΔtIR(j−1)∗,ITj−ΔtITj∗−ΔtIT(j−1)∗,Rj−ΔtRj∗−ΔtR(j−1)∗,Dj−ΔtDj∗−ΔtD(j−1)∗,Vj−ΔtVj∗−ΔtV(j−1)∗)]×ΣIRn+1=+(Δt)α2Γ(α+3)∑j=2n[IR∗(tj,Sj,Ij,IAj,IDj,IRj,ITj,Rj,Dj,Vj)−2IR∗(tj−1,Sj−ΔtSj∗,Ij−ΔtIj∗,IAj−ΔtIAj∗,IDj−ΔtIDj∗,IRj−ΔtIRj∗,ITj−ΔtITj∗,Rj−ΔtRj∗,Dj−ΔtDj∗,Vj−ΔtVj∗)+IR∗(tj−2,Sj−ΔtSj∗−ΔtS(j−1)∗,Ij−ΔtIj∗−ΔtI(j−1)∗,IAj−ΔtIAj∗−ΔtIA(j−1)∗,IDj−ΔtIDj∗−ΔtID(j−1)∗,IRj−ΔtIRj∗−ΔtIR(j−1)∗,ITj−ΔtITj∗−ΔtIT(j−1)∗,Rj−ΔtRj∗−ΔtR(j−1)∗,Dj−ΔtDj∗−ΔtD(j−1)∗,Vj−ΔtVj∗−ΔtV(j−1)∗)]IRn+1=×Δ,ITn+1=(Δt)αΓ(α+1)∑j=2nIT∗(tj−2,Sj−ΔtSj∗−ΔtS(j−1)∗,Ij−ΔtIj∗−ΔtI(j−1)∗,IAj−ΔtIAj∗−ΔtIA(j−1)∗,IDj−ΔtIDj∗−ΔtID(j−1)∗,IRj−ΔtIRj∗−ΔtIR(j−1)∗,ITj−ΔtITj∗−ΔtIT(j−1)∗,Rj−ΔtRj∗−ΔtR(j−1)∗,Dj−ΔtDj∗−ΔtD(j−1)∗,Vj−ΔtVj∗−ΔtV(j−1)∗)×ΠITn+1=+α(Δt)αΓ(α+2)∑j=2n[IT∗(tj−1,Sj−ΔtSj∗,Ij−ΔtIj∗,IAj−ΔtIAj∗,IDj−ΔtIDj∗,IRj−ΔtIRj∗,ITj−ΔtITj∗,Rj−ΔtRj∗,Dj−ΔtDj∗,Vj−ΔtVj∗)−IT∗(tj−2,Sj−ΔtSj∗−ΔtS(j−1)∗,Ij−ΔtIj∗−ΔtI(j−1)∗,IAj−ΔtIAj∗−ΔtIA(j−1)∗,IDj−ΔtIDj∗−ΔtID(j−1)∗,IRj−ΔtIRj∗−ΔtIR(j−1)∗,ITj−ΔtITj∗−ΔtIT(j−1)∗,Rj−ΔtRj∗−ΔtR(j−1)∗,Dj−ΔtDj∗−ΔtD(j−1)∗,Vj−ΔtVj∗−ΔtV(j−1)∗)]×ΣITn+1=+(Δt)α2Γ(α+3)∑j=2n[IT∗(tj,Sj,Ij,IAj,IDj,IRj,ITj,Rj,Dj,Vj)−2IT∗(tj−1,Sj−ΔtSj∗,Ij−ΔtIj∗,IAj−ΔtIAj∗,IDj−ΔtIDj∗,IRj−ΔtIRj∗,ITj−ΔtITj∗,Rj−ΔtRj∗,Dj−ΔtDj∗,Vj−ΔtVj∗)+IT∗(tj−2,Sj−ΔtSj∗−ΔtS(j−1)∗,Ij−ΔtIj∗−ΔtI(j−1)∗,IAj−ΔtIAj∗−ΔtIA(j−1)∗,IDj−ΔtIDj∗−ΔtID(j−1)∗,IRj−ΔtIRj∗−ΔtIR(j−1)∗,ITj−ΔtITj∗−ΔtIT(j−1)∗,Rj−ΔtRj∗−ΔtR(j−1)∗,Dj−ΔtDj∗−ΔtD(j−1)∗,Vj−ΔtVj∗−ΔtV(j−1)∗)]ITn+1=×Δ,Rn+1=(Δt)αΓ(α+1)∑j=2nR∗(tj−2,Sj−ΔtSj∗−ΔtS(j−1)∗,Ij−ΔtIj∗−ΔtI(j−1)∗,IAj−ΔtIAj∗−ΔtIA(j−1)∗,IDj−ΔtIDj∗−ΔtID(j−1)∗,IRj−ΔtIRj∗−ΔtIR(j−1)∗,ITj−ΔtITj∗−ΔtIT(j−1)∗,Rj−ΔtRj∗−ΔtR(j−1)∗,Dj−ΔtDj∗−ΔtD(j−1)∗,Vj−ΔtVj∗−ΔtV(j−1)∗)×ΠRn+1=+(Δt)αΓ(α+2)∑j=2n[R∗(tj−1,Sj−ΔtSj∗,Ij−ΔtIj∗,IAj−ΔtIAj∗,IDj−ΔtIDj∗,IRj−ΔtIRj∗,ITj−ΔtITj∗,Rj−ΔtRj∗,Dj−ΔtDj∗,Vj−ΔtVj∗)−R∗(tj−2,Sj−ΔtSj∗−ΔtS(j−1)∗,Ij−ΔtIj∗−ΔtI(j−1)∗,IAj−ΔtIAj∗−ΔtIA(j−1)∗,IDj−ΔtIDj∗−ΔtID(j−1)∗,IRj−ΔtIRj∗−ΔtIR(j−1)∗,ITj−ΔtITj∗−ΔtIT(j−1)∗,Rj−ΔtRj∗−ΔtR(j−1)∗,Dj−ΔtDj∗−ΔtD(j−1)∗,Vj−ΔtVj∗−ΔtV(j−1)∗)]Rn+1=×ΣRn+1=+(Δt)α2Γ(α+3)∑j=2n[R∗(tj,Sj,Ij,IAj,IDj,IRj,ITj,Rj,Dj,Vj)−2R∗(tj−1,Sj−ΔtSj∗,Ij−ΔtIj∗,IAj−ΔtIAj∗,IDj−ΔtIDj∗,IRj−ΔtIRj∗,ITj−ΔtITj∗,Rj−ΔtRj∗,Dj−ΔtDj∗,Vj−ΔtVj∗)+R∗(tj−2,Sj−ΔtSj∗−ΔtS(j−1)∗,Ij−ΔtIj∗−ΔtI(j−1)∗,IAj−ΔtIAj∗−ΔtIA(j−1)∗,IDj−ΔtIDj∗−ΔtID(j−1)∗,IRj−ΔtIRj∗−ΔtIR(j−1)∗,ITj−ΔtITj∗−ΔtIT(j−1)∗,Rj−ΔtRj∗−ΔtR(j−1)∗,Dj−ΔtDj∗−ΔtD(j−1)∗,Vj−ΔtVj∗−ΔtV(j−1)∗)]Rn+1=×Δ,Dn+1=(Δt)αΓ(α+1)∑j=2nD∗(tj−2,Sj−ΔtSj∗−ΔtS(j−1)∗,Ij−ΔtIj∗−ΔtI(j−1)∗,IAj−ΔtIAj∗−ΔtIA(j−1)∗,IDj−ΔtIDj∗−ΔtID(j−1)∗,IRj−ΔtIRj∗−ΔtIR(j−1)∗,ITj−ΔtITj∗−ΔtIT(j−1)∗,Rj−ΔtRj∗−ΔtR(j−1)∗,Dj−ΔtDj∗−ΔtD(j−1)∗,Vj−ΔtVj∗−ΔtV(j−1)∗)×ΠDn+1=+(Δt)αΓ(α+2)∑j=2n[D∗(tj−1,Sj−ΔtSj∗,Ij−ΔtIj∗,IAj−ΔtIAj∗,IDj−ΔtIDj∗,IRj−ΔtIRj∗,ITj−ΔtITj∗,Rj−ΔtRj∗,Dj−ΔtDj∗,Vj−ΔtVj∗)−D∗(tj−2,Sj−ΔtSj∗−ΔtS(j−1)∗,Ij−ΔtIj∗−ΔtI(j−1)∗,IAj−ΔtIAj∗−ΔtIA(j−1)∗,IDj−ΔtIDj∗−ΔtID(j−1)∗,IRj−ΔtIRj∗−ΔtIR(j−1)∗,ITj−ΔtITj∗−ΔtIT(j−1)∗,Rj−ΔtRj∗−ΔtR(j−1)∗,Dj−ΔtDj∗−ΔtD(j−1)∗,Vj−ΔtVj∗−ΔtV(j−1)∗)]Dn+1=×ΣDn+1=+(Δt)α2Γ(α+3)∑j=2n[D∗(tj,Sj,Ij,IAj,IDj,IRj,ITj,Rj,Dj,Vj)−2D∗(tj−1,Sj−ΔtSj∗,Ij−ΔtIj∗,IAj−ΔtIAj∗,IDj−ΔtIDj∗,IRj−ΔtIRj∗,ITj−ΔtITj∗,Rj−ΔtRj∗,Dj−ΔtDj∗,Vj−ΔtVj∗)+D∗(tj−2,Sj−ΔtSj∗−ΔtS(j−1)∗,Ij−ΔtIj∗−ΔtI(j−1)∗,IAj−ΔtIAj∗−ΔtIA(j−1)∗,IDj−ΔtIDj∗−ΔtID(j−1)∗,IRj−ΔtIRj∗−ΔtIR(j−1)∗,ITj−ΔtITj∗−ΔtIT(j−1)∗,Rj−ΔtRj∗−ΔtR(j−1)∗,Dj−ΔtDj∗−ΔtD(j−1)∗,Vj−ΔtVj∗−ΔtV(j−1)∗)]Dn+1=×Δ,Vn+1=(Δt)αΓ(α+1)∑j=2nV∗(tj−2,Sj−ΔtSj∗−ΔtS(j−1)∗,Ij−ΔtIj∗−ΔtI(j−1)∗,IAj−ΔtIAj∗−ΔtIA(j−1)∗,IDj−ΔtIDj∗−ΔtID(j−1)∗,IRj−ΔtIRj∗−ΔtIR(j−1)∗,ITj−ΔtITj∗−ΔtIT(j−1)∗,Rj−ΔtRj∗−ΔtR(j−1)∗,Dj−ΔtDj∗−ΔtD(j−1)∗,Vj−ΔtVj∗−ΔtV(j−1)∗)×Π+(Δt)αΓ(α+2)∑j=2n[V∗(tj−1,Sj−ΔtSj∗,Ij−ΔtIj∗,IAj−ΔtIAj∗,IDj−ΔtIDj∗,IRj−ΔtIRj∗,ITj−ΔtITj∗,Rj−ΔtRj∗,Dj−ΔtDj∗,Vj−ΔtVj∗)−V∗(tj−2,Sj−ΔtSj∗−ΔtS(j−1)∗,Ij−ΔtIj∗−ΔtI(j−1)∗,IAj−ΔtIAj∗−ΔtIA(j−1)∗,IDj−ΔtIDj∗−ΔtID(j−1)∗,IRj−ΔtIRj∗−ΔtIR(j−1)∗,ITj−ΔtITj∗−ΔtIT(j−1)∗,Rj−ΔtRj∗−ΔtR(j−1)∗,Dj−ΔtDj∗−ΔtD(j−1)∗,Vj−ΔtVj∗−ΔtV(j−1)∗)]×Σ+(Δt)α2Γ(α+3)∑j=2n[V∗(tj,Sj,Ij,IAj,IDj,IRj,ITj,Rj,Dj,Vj)−2V∗(tj−1,Sj−ΔtSj∗,Ij−ΔtIj∗,IAj−ΔtIAj∗,IDj−ΔtIDj∗,IRj−ΔtIRj∗,ITj−ΔtITj∗,Rj−ΔtRj∗,Dj−ΔtDj∗,Vj−ΔtVj∗)+V∗(tj−2,Sj−ΔtSj∗−ΔtS(j−1)∗,Ij−ΔtIj∗−ΔtI(j−1)∗,IAj−ΔtIAj∗−ΔtIA(j−1)∗,IDj−ΔtIDj∗−ΔtID(j−1)∗,IRj−ΔtIRj∗−ΔtIR(j−1)∗,ITj−ΔtITj∗−ΔtIT(j−1)∗,Rj−ΔtRj∗−ΔtR(j−1)∗,Dj−ΔtDj∗−ΔtD(j−1)∗,Vj−ΔtVj∗−ΔtV(j−1)∗)]×Δ.

We now do the same routine for fractal-fractional derivatives. We start with the Caputo–Fabrizio fractal-fractional derivative

124 $$\begin{aligned}& {}_{0}^{FFE}D_{t}^{\alpha }S = S^{\ast } ( t,S,I,I_{A},I_{D},I_{R},I_{T},R,D,V ), \\& {}_{0}^{FFE}D_{t}^{\alpha }I = I^{\ast } ( t,S,I,I_{A},I_{D},I_{R},I_{T},R,D,V ), \\& {}_{0}^{FFE}D_{t}^{\alpha }I_{A} = I_{A}^{\ast } ( t,S,I,I_{A},I_{D},I_{R},I_{T},R,D,V ), \\& {}_{0}^{FFE}D_{t}^{\alpha }I_{D} = I_{D}^{\ast } ( t,S,I,I_{A},I_{D},I_{R},I_{T},R,D,V ), \\& {}_{0}^{FFE}D_{t}^{\alpha }I_{R} = I_{R}^{\ast } ( t,S,I,I_{A},I_{D},I_{R},I_{T},R,D,V ), \\& {}_{0}^{FFE}D_{t}^{\alpha }I_{T} = I_{T}^{\ast } ( t,S,I,I_{A},I_{D},I_{R},I_{T},R,D,V ), \\& {}_{0}^{FFE}D_{t}^{\alpha }R = R^{\ast } ( t,S,I,I_{A},I_{D},I_{R},I_{T},R,D,V ), \\& {}_{0}^{FFE}D_{t}^{\alpha }D = D^{\ast } ( t,S,I,I_{A},I_{D},I_{R},I_{T},R,D,V ), \\& {}_{0}^{FFE}D_{t}^{\alpha }V = V^{\ast } ( t,S,I,I_{A},I_{D},I_{R},I_{T},R,D,V ) . \end{aligned}$$

After applying the fractional integral with exponential kernel and putting the Newton polynomial into these equations, we can solve our model as follows:

125 $$\begin{array}{rl} S^{n+1}= & S^{n}+\frac{1-\alpha }{M(\alpha )}\left[\begin{array}{c} t_{n+1}^{1-\beta }S^{*}\left(\begin{array}{c} t_{n+1},S^{n}+\Delta tS^{n*},I^{n}+\Delta tI^{n*},I_{A}^{n}+\Delta tI_{A}^{n*},\\ I_{D}^{n}+\Delta tI_{D}^{n*},I_{R}^{n}+\Delta tI_{R}^{n*},I_{T}^{n}+\Delta tI_{T}^{n*},\\ R^{n}+\Delta tR^{n*},D^{n}+\Delta tD^{n*},V^{n}+\Delta tV^{n*} \end{array}\right)\\ -t_{n}^{1-\beta }S^{*}\left(t_{n},S^{n},I^{n},I_{A}^{n},I_{D}^{n},I_{R}^{n},I_{T}^{n},R^{n},D^{n},V^{n}\right) \end{array}\right]\\ & +\frac{\alpha }{M(\alpha )}\\ & \times \left\{\begin{array}{c} \frac{23}{12}t_{n}^{1-\beta }S^{*}(t_{n},S^{n},I^{n},I_{A}^{n},I_{D}^{n},I_{R}^{n},I_{T}^{n},R^{n},D^{n},V^{n})\Delta t\\ -\frac{4}{3}t_{n-1}^{1-\beta }S^{*}\left(\begin{array}{c} t_{n-1},S^{n}-\Delta tS^{n*},I^{n}-\Delta tI^{n*},I_{A}^{n}-\Delta tI_{A}^{n*},\\ I_{D}^{n}-\Delta tI_{D}^{n*},I_{R}^{n}-\Delta tI_{R}^{n*},I_{T}^{n}-\Delta tI_{T}^{n*},\\ R^{n}-\Delta tR^{n*},D^{n}-\Delta tD^{n*},V^{n}-\Delta tV^{n*} \end{array}\right)\Delta t\\ +\frac{5}{12}t_{n-2}^{1-\beta }S^{*}\left(\begin{array}{c} t_{n-2},S^{n}-\Delta tS^{n*}-\Delta tS^{(n-1)*},I^{n}-\Delta tI^{n*}-\Delta tI^{(n-1)*},\\ I_{A}^{n}-\Delta tI_{A}^{n*}-\Delta tI_{A}^{(n-1)*},I_{D}^{n}-\Delta tI_{D}^{n*}-\Delta tI_{D}^{(n-1)*},\\ I_{R}^{n}-\Delta tI_{R}^{n*}-\Delta tI_{R}^{(n-1)*},I_{T}^{n}-\Delta tI_{T}^{n*}-\Delta tI_{T}^{(n-1)*},\\ R^{n}-\Delta tR^{n*}-\Delta tR^{(n-1)*},D^{n}-\Delta tD^{n*}-\Delta tD^{(n-1)*},\\ V^{n}-\Delta tV^{n*}-\Delta tV^{(n-1)*} \end{array}\right)\Delta t \end{array}\right\}, \end{array}$$

126 $$\begin{aligned}& I^{n+1} =S^{n}+\frac{1-\alpha }{M ( \alpha ) } \end{aligned}$$

127 In+1=[tn+11−βI∗(tn+1,Sn+ΔtSn∗,In+ΔtIn∗,IAn+ΔtIAn∗,IDn+ΔtIDn∗,IRn+ΔtIRn∗,ITn+ΔtITn∗,Rn+ΔtRn∗,Dn+ΔtDn∗,Vn+ΔtVn∗)−tn1−βI∗(tn,Sn,In,IAn,IDn,IRn,ITn,Rn,Dn,Vn)]In+1=+αM(α)In+1=×{2312tn1−βI∗(tn,Sn,In,IAn,IDn,IRn,ITn,Rn,Dn,Vn)Δt−43tn−11−βI∗(tn−1,Sn−ΔtSn∗,In−ΔtIn∗,IAn−ΔtIAn∗,IDn−ΔtIDn∗,IRn−ΔtIRn∗,ITn−ΔtITn∗,Rn−ΔtRn∗,Dn−ΔtDn∗,Vn−ΔtVn∗)Δt+512tn−21−βI∗(tn−2,Sn−ΔtSn∗−ΔtS(n−1)∗,In−ΔtIn∗−ΔtI(n−1)∗,IAn−ΔtIAn∗−ΔtIA(n−1)∗,IDn−ΔtIDn∗−ΔtID(n−1)∗,IRn−ΔtIRn∗−ΔtIR(n−1)∗,ITn−ΔtITn∗−ΔtIT(n−1)∗,Rn−ΔtRn∗−ΔtR(n−1)∗,Dn−ΔtDn∗−ΔtD(n−1)∗,Vn−ΔtVn∗−ΔtV(n−1)∗)Δt},IAn+1=IAn+1−αM(α)[tn+11−βIA∗(tn+1,Sn+ΔtSn∗,In+ΔtIn∗,IAn+ΔtIAn∗,IDn+ΔtIDn∗,IRn+ΔtIRn∗,ITn+ΔtITn∗,Rn+ΔtRn∗,Dn+ΔtDn∗,Vn+ΔtVn∗)−tn1−βIA∗(tn,Sn,In,IAn,IDn,IRn,ITn,Rn,Dn,Vn)]+αM(α)×{2312tn1−βIA∗(tn,Sn,In,IAn,IDn,IRn,ITn,Rn,Dn,Vn)Δt−43tn−11−βIA∗(tn−1,Sn−ΔtSn∗,In−ΔtIn∗,IAn−ΔtIAn∗,IDn−ΔtIDn∗,IRn−ΔtIRn∗,ITn−ΔtITn∗,Rn−ΔtRn∗,Dn−ΔtDn∗,Vn−ΔtVn∗)Δt+512tn−21−βIA∗(tn−2,Sn−ΔtSn∗−ΔtS(n−1)∗,In−ΔtIn∗−ΔtI(n−1)∗,IAn−ΔtIAn∗−ΔtIA(n−1)∗,IDn−ΔtIDn∗−ΔtID(n−1)∗,IRn−ΔtIRn∗−ΔtIR(n−1)∗,ITn−ΔtITn∗−ΔtIT(n−1)∗,Rn−ΔtRn∗−ΔtR(n−1)∗,Dn−ΔtDn∗−ΔtD(n−1)∗,Vn−ΔtVn∗−ΔtV(n−1)∗)Δt},

128 $$\begin{array}{rl} I_{D}^{n+1}= & I_{D}^{n}+\frac{1-\alpha }{M(\alpha )}\left[\begin{array}{c} t_{n+1}^{1-\beta }I_{D}^{*}\left(\begin{array}{c} t_{n+1},S^{n}+\Delta tS^{n*},I^{n}+\Delta tI^{n*},I_{A}^{n}+\Delta tI_{A}^{n*},\\ I_{D}^{n}+\Delta tI_{D}^{n*},I_{R}^{n}+\Delta tI_{R}^{n*},I_{T}^{n}+\Delta tI_{T}^{n*},\\ R^{n}+\Delta tR^{n*},D^{n}+\Delta tD^{n*},V^{n}+\Delta tV^{n*} \end{array}\right)\\ -t_{n}^{1-\beta }I_{D}^{*}\left(t_{n},S^{n},I^{n},I_{A}^{n},I_{D}^{n},I_{R}^{n},I_{T}^{n},R^{n},D^{n},V^{n}\right) \end{array}\right]\\ & +\frac{\alpha }{M(\alpha )}\\ & \times \left\{\begin{array}{c} \frac{23}{12}t_{n}^{1-\beta }I_{D}^{*}(t_{n},S^{n},I^{n},I_{A}^{n},I_{D}^{n},I_{R}^{n},I_{T}^{n},R^{n},D^{n},V^{n})\Delta t\\ -\frac{4}{3}t_{n-1}^{1-\beta }I_{D}^{*}\left(\begin{array}{c} t_{n-1},S^{n}-\Delta tS^{n*},I^{n}-\Delta tI^{n*},I_{A}^{n}-\Delta tI_{A}^{n*},\\ I_{D}^{n}-\Delta tI_{D}^{n*},I_{R}^{n}-\Delta tI_{R}^{n*},I_{T}^{n}-\Delta tI_{T}^{n*},\\ R^{n}-\Delta tR^{n*},D^{n}-\Delta tD^{n*},V^{n}-\Delta tV^{n*} \end{array}\right)\Delta t\\ +\frac{5}{12}t_{n-2}^{1-\beta }I_{D}^{*}\left(\begin{array}{c} t_{n-2},S^{n}-\Delta tS^{n*}-\Delta tS^{(n-1)*},I^{n}-\Delta tI^{n*}-\Delta tI^{(n-1)*},\\ I_{A}^{n}-\Delta tI_{A}^{n*}-\Delta tI_{A}^{(n-1)*},I_{D}^{n}-\Delta tI_{D}^{n*}-\Delta tI_{D}^{(n-1)*},\\ I_{R}^{n}-\Delta tI_{R}^{n*}-\Delta tI_{R}^{(n-1)*},I_{T}^{n}-\Delta tI_{T}^{n*}-\Delta tI_{T}^{(n-1)*},\\ R^{n}-\Delta tR^{n*}-\Delta tR^{(n-1)*},D^{n}-\Delta tD^{n*}-\Delta tD^{(n-1)*},\\ V^{n}-\Delta tV^{n*}-\Delta tV^{(n-1)*} \end{array}\right)\Delta t \end{array}\right\}, \end{array}$$

129 $$I_{R}^{n+1}=I_{R}^{n}+\frac{1-\alpha }{M(\alpha )}\left[\begin{array}{c} t_{n+1}^{1-\beta }I_{R}^{*}\left(\begin{array}{c} t_{n+1},S^{n}+\Delta tS^{n*},I^{n}+\Delta tI^{n*},I_{A}^{n}+\Delta tI_{A}^{n*},\\ I_{D}^{n}+\Delta tI_{D}^{n*},I_{R}^{n}+\Delta tI_{R}^{n*},I_{T}^{n}+\Delta tI_{T}^{n*},\\ R^{n}+\Delta tR^{n*},D^{n}+\Delta tD^{n*},V^{n}+\Delta tV^{n*} \end{array}\right)\\ -t_{n}^{1-\beta }I_{R}^{*}\left(t_{n},S^{n},I^{n},I_{A}^{n},I_{D}^{n},I_{R}^{n},I_{T}^{n},R^{n},D^{n},V^{n}\right) \end{array}\right]$$

130 IRn+1=+αM(α)IRn+1=×{2312tn1−βIR∗(tn,Sn,In,IAn,IDn,IRn,ITn,Rn,Dn,Vn)Δt−43tn−11−βIR∗(tn−1,Sn−ΔtSn∗,In−ΔtIn∗,IAn−ΔtIAn∗,IDn−ΔtIDn∗,IRn−ΔtIRn∗,ITn−ΔtITn∗,Rn−ΔtRn∗,Dn−ΔtDn∗,Vn−ΔtVn∗)Δt+512tn−21−βIR∗(tn−2,Sn−ΔtSn∗−ΔtS(n−1)∗,In−ΔtIn∗−ΔtI(n−1)∗,IAn−ΔtIAn∗−ΔtIA(n−1)∗,IDn−ΔtIDn∗−ΔtID(n−1)∗,IRn−ΔtIRn∗−ΔtIR(n−1)∗,ITn−ΔtITn∗−ΔtIT(n−1)∗,Rn−ΔtRn∗−ΔtR(n−1)∗,Dn−ΔtDn∗−ΔtD(n−1)∗,Vn−ΔtVn∗−ΔtV(n−1)∗)Δt},ITn+1=ITn+1−αM(α)[tn+11−βIT∗(tn+1,Sn+ΔtSn∗,In+ΔtIn∗,IAn+ΔtIAn∗,IDn+ΔtIDn∗,IRn+ΔtIRn∗,ITn+ΔtITn∗,Rn+ΔtRn∗,Dn+ΔtDn∗,Vn+ΔtVn∗)−tn1−βIT∗(tn,Sn,In,IAn,IDn,IRn,ITn,Rn,Dn,Vn)]+αM(α)×{2312tn1−βIT∗(tn,Sn,In,IAn,IDn,IRn,ITn,Rn,Dn,Vn)Δt−43tn−11−βIT∗(tn−1,Sn−ΔtSn∗,In−ΔtIn∗,IAn−ΔtIAn∗,IDn−ΔtIDn∗,IRn−ΔtIRn∗,ITn−ΔtITn∗,Rn−ΔtRn∗,Dn−ΔtDn∗,Vn−ΔtVn∗)Δt+512tn−21−βIT∗(tn−2,Sn−ΔtSn∗−ΔtS(n−1)∗,In−ΔtIn∗−ΔtI(n−1)∗,IAn−ΔtIAn∗−ΔtIA(n−1)∗,IDn−ΔtIDn∗−ΔtID(n−1)∗,IRn−ΔtIRn∗−ΔtIR(n−1)∗,ITn−ΔtITn∗−ΔtIT(n−1)∗,Rn−ΔtRn∗−ΔtR(n−1)∗,Dn−ΔtDn∗−ΔtD(n−1)∗,Vn−ΔtVn∗−ΔtV(n−1)∗)Δt},

131 $$\begin{array}{rl} R^{n+1}= & R^{n}+\frac{1-\alpha }{M(\alpha )}\left[\begin{array}{c} t_{n+1}^{1-\beta }R^{*}\left(\begin{array}{c} t_{n+1},S^{n}+\Delta tS^{n*},I^{n}+\Delta tI^{n*},I_{A}^{n}+\Delta tI_{A}^{n*},\\ I_{D}^{n}+\Delta tI_{D}^{n*},I_{R}^{n}+\Delta tI_{R}^{n*},I_{T}^{n}+\Delta tI_{T}^{n*},\\ R^{n}+\Delta tR^{n*},D^{n}+\Delta tD^{n*},V^{n}+\Delta tV^{n*} \end{array}\right)\\ -t_{n}^{1-\beta }R^{*}\left(t_{n},S^{n},I^{n},I_{A}^{n},I_{D}^{n},I_{R}^{n},I_{T}^{n},R^{n},D^{n},V^{n}\right) \end{array}\right]\\ & +\frac{\alpha }{M(\alpha )}\\ & \times \left\{\begin{array}{c} \frac{23}{12}t_{n}^{1-\beta }R^{*}(t_{n},S^{n},I^{n},I_{A}^{n},I_{D}^{n},I_{R}^{n},I_{T}^{n},R^{n},D^{n},V^{n})\Delta t\\ -\frac{4}{3}t_{n-1}^{1-\beta }R^{*}\left(\begin{array}{c} t_{n-1},S^{n}-\Delta tS^{n*},I^{n}-\Delta tI^{n*},I_{A}^{n}-\Delta tI_{A}^{n*},\\ I_{D}^{n}-\Delta tI_{D}^{n*},I_{R}^{n}-\Delta tI_{R}^{n*},I_{T}^{n}-\Delta tI_{T}^{n*},\\ R^{n}-\Delta tR^{n*},D^{n}-\Delta tD^{n*},V^{n}-\Delta tV^{n*} \end{array}\right)\Delta t\\ +\frac{5}{12}t_{n-2}^{1-\beta }R^{*}\left(\begin{array}{c} t_{n-2},S^{n}-\Delta tS^{n*}-\Delta tS^{(n-1)*},I^{n}-\Delta tI^{n*}-\Delta tI^{(n-1)*},\\ I_{A}^{n}-\Delta tI_{A}^{n*}-\Delta tI_{A}^{(n-1)*},I_{D}^{n}-\Delta tI_{D}^{n*}-\Delta tI_{D}^{(n-1)*},\\ I_{R}^{n}-\Delta tI_{R}^{n*}-\Delta tI_{R}^{(n-1)*},I_{T}^{n}-\Delta tI_{T}^{n*}-\Delta tI_{T}^{(n-1)*},\\ R^{n}-\Delta tR^{n*}-\Delta tR^{(n-1)*},D^{n}-\Delta tD^{n*}-\Delta tD^{(n-1)*},\\ V^{n}-\Delta tV^{n*}-\Delta tV^{(n-1)*} \end{array}\right)\Delta t \end{array}\right\}, \end{array}$$

132 $$\begin{array}{rl} D^{n+1}= & D^{n}+\frac{1-\alpha }{M(\alpha )}\left[\begin{array}{c} t_{n+1}^{1-\beta }D^{*}\left(\begin{array}{c} t_{n+1},S^{n}+\Delta tS^{n*},I^{n}+\Delta tI^{n*},I_{A}^{n}+\Delta tI_{A}^{n*},\\ I_{D}^{n}+\Delta tI_{D}^{n*},I_{R}^{n}+\Delta tI_{R}^{n*},I_{T}^{n}+\Delta tI_{T}^{n*},\\ R^{n}+\Delta tR^{n*},D^{n}+\Delta tD^{n*},V^{n}+\Delta tV^{n*} \end{array}\right)\\ -t_{n}^{1-\beta }D^{*}\left(t_{n},S^{n},I^{n},I_{A}^{n},I_{D}^{n},I_{R}^{n},I_{T}^{n},R^{n},D^{n},V^{n}\right) \end{array}\right]\\ & +\frac{\alpha }{M(\alpha )}\\ & \times \left\{\begin{array}{c} \frac{23}{12}t_{n}^{1-\beta }D^{*}(t_{n},S^{n},I^{n},I_{A}^{n},I_{D}^{n},I_{R}^{n},I_{T}^{n},R^{n},D^{n},V^{n})\Delta t\\ -\frac{4}{3}t_{n-1}^{1-\beta }D^{*}\left(\begin{array}{c} t_{n-1},S^{n}-\Delta tS^{n*},I^{n}-\Delta tI^{n*},I_{A}^{n}-\Delta tI_{A}^{n*},\\ I_{D}^{n}-\Delta tI_{D}^{n*},I_{R}^{n}-\Delta tI_{R}^{n*},I_{T}^{n}-\Delta tI_{T}^{n*},\\ R^{n}-\Delta tR^{n*},D^{n}-\Delta tD^{n*},V^{n}-\Delta tV^{n*} \end{array}\right)\Delta t\\ +\frac{5}{12}t_{n-2}^{1-\beta }D^{*}\left(\begin{array}{c} t_{n-2},S^{n}-\Delta tS^{n*}-\Delta tS^{(n-1)*},I^{n}-\Delta tI^{n*}-\Delta tI^{(n-1)*},\\ I_{A}^{n}-\Delta tI_{A}^{n*}-\Delta tI_{A}^{(n-1)*},I_{D}^{n}-\Delta tI_{D}^{n*}-\Delta tI_{D}^{(n-1)*},\\ I_{R}^{n}-\Delta tI_{R}^{n*}-\Delta tI_{R}^{(n-1)*},I_{T}^{n}-\Delta tI_{T}^{n*}-\Delta tI_{T}^{(n-1)*},\\ R^{n}-\Delta tR^{n*}-\Delta tR^{(n-1)*},D^{n}-\Delta tD^{n*}-\Delta tD^{(n-1)*},\\ V^{n}-\Delta tV^{n*}-\Delta tV^{(n-1)*} \end{array}\right)\Delta t \end{array}\right\}, \end{array}$$

133 $$\begin{array}{rl} V^{n+1}= & V^{n}+\frac{1-\alpha }{M(\alpha )}\left[\begin{array}{c} t_{n+1}^{1-\beta }V^{*}\left(\begin{array}{c} t_{n+1},S^{n}+\Delta tS^{n*},I^{n}+\Delta tI^{n*},I_{A}^{n}+\Delta tI_{A}^{n*},\\ I_{D}^{n}+\Delta tI_{D}^{n*},I_{R}^{n}+\Delta tI_{R}^{n*},I_{T}^{n}+\Delta tI_{T}^{n*},\\ R^{n}+\Delta tR^{n*},D^{n}+\Delta tD^{n*},V^{n}+\Delta tV^{n*} \end{array}\right)\\ -t_{n}^{1-\beta }V^{*}\left(t_{n},S^{n},I^{n},I_{A}^{n},I_{D}^{n},I_{R}^{n},I_{T}^{n},R^{n},D^{n},V^{n}\right) \end{array}\right]\\ & +\frac{\alpha }{M(\alpha )}\\ & \times \left\{\begin{array}{c} \frac{23}{12}t_{n}^{1-\beta }V^{*}(t_{n},S^{n},I^{n},I_{A}^{n},I_{D}^{n},I_{R}^{n},I_{T}^{n},R^{n},D^{n},V^{n})\Delta t\\ -\frac{4}{3}t_{n-1}^{1-\beta }V^{*}\left(\begin{array}{c} t_{n-1},S^{n}-\Delta tS^{n*},I^{n}-\Delta tI^{n*},I_{A}^{n}-\Delta tI_{A}^{n*},\\ I_{D}^{n}-\Delta tI_{D}^{n*},I_{R}^{n}-\Delta tI_{R}^{n*},I_{T}^{n}-\Delta tI_{T}^{n*},\\ R^{n}-\Delta tR^{n*},D^{n}-\Delta tD^{n*},V^{n}-\Delta tV^{n*} \end{array}\right)\Delta t\\ +\frac{5}{12}t_{n-2}^{1-\beta }V^{*}\left(\begin{array}{c} t_{n-2},S^{n}-\Delta tS^{n*}-\Delta tS^{(n-1)*},I^{n}-\Delta tI^{n*}-\Delta tI^{(n-1)*},\\ I_{A}^{n}-\Delta tI_{A}^{n*}-\Delta tI_{A}^{(n-1)*},I_{D}^{n}-\Delta tI_{D}^{n*}-\Delta tI_{D}^{(n-1)*},\\ I_{R}^{n}-\Delta tI_{R}^{n*}-\Delta tI_{R}^{(n-1)*},I_{T}^{n}-\Delta tI_{T}^{n*}-\Delta tI_{T}^{(n-1)*},\\ R^{n}-\Delta tR^{n*}-\Delta tR^{(n-1)*},D^{n}-\Delta tD^{n*}-\Delta tD^{(n-1)*},\\ V^{n}-\Delta tV^{n*}-\Delta tV^{(n-1)*} \end{array}\right)\Delta t \end{array}\right\}. \end{array}$$

For the Atangana–Baleanu fractal-fractional derivative, we can have the following numerical scheme:

134 Sn+1=1−αAB(α)tn+11−βS∗(tn+1,Sn+ΔtSn∗,In+ΔtIn∗,IAn+ΔtIAn∗,IDn+ΔtIDn∗,IRn+ΔtIRn∗,ITn+ΔtITn∗,Rn+ΔtRn∗,Dn+ΔtDn∗,Vn+ΔtVn∗)Sn+1=+α(Δt)αAB(α)Γ(α+1)Sn+1=×∑j=2ntj−21−βS∗(tj−2,Sj−ΔtSj∗−ΔtS(j−1)∗,Ij−ΔtIj∗−ΔtI(j−1)∗,IAj−ΔtIAj∗−ΔtIA(j−1)∗,IDj−ΔtIDj∗−ΔtID(j−1)∗,IRj−ΔtIRj∗−ΔtIR(j−1)∗,ITj−ΔtITj∗−ΔtIT(j−1)∗,Rj−ΔtRj∗−ΔtR(j−1)∗,Dj−ΔtDj∗−ΔtD(j−1)∗,Vj−ΔtVj∗−ΔtV(j−1)∗)×ΠSn+1=+α(Δt)αAB(α)Γ(α+2)Sn+1=×∑j=2n[tj−11−βS∗(tj−1,Sj−ΔtSj∗,Ij−ΔtIj∗,IAj−ΔtIAj∗,IDj−ΔtIDj∗,IRj−ΔtIRj∗,ITj−ΔtITj∗,Rj−ΔtRj∗,Dj−ΔtDj∗,Vj−ΔtVj∗)−tj−21−βS∗(tj−2,Sj−ΔtSj∗−ΔtS(j−1)∗,Ij−ΔtIj∗−ΔtI(j−1)∗,IAj−ΔtIAj∗−ΔtIA(j−1)∗,IDj−ΔtIDj∗−ΔtID(j−1)∗,IRj−ΔtIRj∗−ΔtIR(j−1)∗,ITj−ΔtITj∗−ΔtIT(j−1)∗,Rj−ΔtRj∗−ΔtR(j−1)∗,Dj−ΔtDj∗−ΔtD(j−1)∗,Vj−ΔtVj∗−ΔtV(j−1)∗)]×ΣSn+1=+α(Δt)α2AB(α)Γ(α+3)Sn+1=×∑j=2n[tj1−βS∗(tj,Sj,Ij,IAj,IDj,IRj,ITj,Rj,Dj,Vj)−2tj−11−βS∗(tj−1,Sj−ΔtSj∗,Ij−ΔtIj∗,IAj−ΔtIAj∗,IDj−ΔtIDj∗,IRj−ΔtIRj∗,ITj−ΔtITj∗,Rj−ΔtRj∗,Dj−ΔtDj∗,Vj−ΔtVj∗)+tj−21−βS∗(tj−2,Sj−ΔtSj∗−ΔtS(j−1)∗,Ij−ΔtIj∗−ΔtI(j−1)∗,IAj−ΔtIAj∗−ΔtIA(j−1)∗,IDj−ΔtIDj∗−ΔtID(j−1)∗,IRj−ΔtIRj∗−ΔtIR(j−1)∗,ITj−ΔtITj∗−ΔtIT(j−1)∗,Rj−ΔtRj∗−ΔtR(j−1)∗,Dj−ΔtDj∗−ΔtD(j−1)∗,Vj−ΔtVj∗−ΔtV(j−1)∗)]×Δ,In+1=1−αAB(α)tn+11−βI∗(tn+1,Sn+ΔtSn∗,In+ΔtIn∗,IAn+ΔtIAn∗,IDn+ΔtIDn∗,IRn+ΔtIRn∗,ITn+ΔtITn∗,Rn+ΔtRn∗,Dn+ΔtDn∗,Vn+ΔtVn∗)In+1=+α(Δt)αAB(α)Γ(α+1)In+1=×∑j=2ntj−21−βI∗(tj−2,Sj−ΔtSj∗−ΔtS(j−1)∗,Ij−ΔtIj∗−ΔtI(j−1)∗,IAj−ΔtIAj∗−ΔtIA(j−1)∗,IDj−ΔtIDj∗−ΔtID(j−1)∗,IRj−ΔtIRj∗−ΔtIR(j−1)∗,ITj−ΔtITj∗−ΔtIT(j−1)∗,Rj−ΔtRj∗−ΔtR(j−1)∗,Dj−ΔtDj∗−ΔtD(j−1)∗,Vj−ΔtVj∗−ΔtV(j−1)∗)×ΠIn+1=+α(Δt)αAB(α)Γ(α+2)In+1=×∑j=2n[tj−11−βI∗(tj−1,Sj−ΔtSj∗,Ij−ΔtIj∗,IAj−ΔtIAj∗,IDj−ΔtIDj∗,IRj−ΔtIRj∗,ITj−ΔtITj∗,Rj−ΔtRj∗,Dj−ΔtDj∗,Vj−ΔtVj∗)−tj−21−βI∗(tj−2,Sj−ΔtSj∗−ΔtS(j−1)∗,Ij−ΔtIj∗−ΔtI(j−1)∗,IAj−ΔtIAj∗−ΔtIA(j−1)∗,IDj−ΔtIDj∗−ΔtID(j−1)∗,IRj−ΔtIRj∗−ΔtIR(j−1)∗,ITj−ΔtITj∗−ΔtIT(j−1)∗,Rj−ΔtRj∗−ΔtR(j−1)∗,Dj−ΔtDj∗−ΔtD(j−1)∗,Vj−ΔtVj∗−ΔtV(j−1)∗)]×ΣIn+1=+α(Δt)α2AB(α)Γ(α+3)In+1=×∑j=2n[tj1−βI∗(tj,Sj,Ij,IAj,IDj,IRj,ITj,Rj,Dj,Vj)−2tj−11−βI∗(tj−1,Sj−ΔtSj∗,Ij−ΔtIj∗,IAj−ΔtIAj∗,IDj−ΔtIDj∗,IRj−ΔtIRj∗,ITj−ΔtITj∗,Rj−ΔtRj∗,Dj−ΔtDj∗,Vj−ΔtVj∗)+tj−21−βI∗(tj−2,Sj−ΔtSj∗−ΔtS(j−1)∗,Ij−ΔtIj∗−ΔtI(j−1)∗,IAj−ΔtIAj∗−ΔtIA(j−1)∗,IDj−ΔtIDj∗−ΔtID(j−1)∗,IRj−ΔtIRj∗−ΔtIR(j−1)∗,ITj−ΔtITj∗−ΔtIT(j−1)∗,Rj−ΔtRj∗−ΔtR(j−1)∗,Dj−ΔtDj∗−ΔtD(j−1)∗,Vj−ΔtVj∗−ΔtV(j−1)∗)]×Δ,IAn+1=1−αAB(α)tn+11−βIA∗(tn+1,Sn+ΔtSn∗,In+ΔtIn∗,IAn+ΔtIAn∗,IDn+ΔtIDn∗,IRn+ΔtIRn∗,ITn+ΔtITn∗,Rn+ΔtRn∗,Dn+ΔtDn∗,Vn+ΔtVn∗)IAn+1=+α(Δt)αAB(α)Γ(α+1)IAn+1=×∑j=2ntj−21−βIA∗(tj−2,Sj−ΔtSj∗−ΔtS(j−1)∗,Ij−ΔtIj∗−ΔtI(j−1)∗,IAj−ΔtIAj∗−ΔtIA(j−1)∗,IDj−ΔtIDj∗−ΔtID(j−1)∗,IRj−ΔtIRj∗−ΔtIR(j−1)∗,ITj−ΔtITj∗−ΔtIT(j−1)∗,Rj−ΔtRj∗−ΔtR(j−1)∗,Dj−ΔtDj∗−ΔtD(j−1)∗,Vj−ΔtVj∗−ΔtV(j−1)∗)×ΠIAn+1=+α(Δt)αAB(α)Γ(α+2)IAn+1=×∑j=2n[tj−11−βIA∗(tj−1,Sj−ΔtSj∗,Ij−ΔtIj∗,IAj−ΔtIAj∗,IDj−ΔtIDj∗,IRj−ΔtIRj∗,ITj−ΔtITj∗,Rj−ΔtRj∗,Dj−ΔtDj∗,Vj−ΔtVj∗)−tj−21−βIA∗(tj−2,Sj−ΔtSj∗−ΔtS(j−1)∗,Ij−ΔtIj∗−ΔtI(j−1)∗,IAj−ΔtIAj∗−ΔtIA(j−1)∗,IDj−ΔtIDj∗−ΔtID(j−1)∗,IRj−ΔtIRj∗−ΔtIR(j−1)∗,ITj−ΔtITj∗−ΔtIT(j−1)∗,Rj−ΔtRj∗−ΔtR(j−1)∗,Dj−ΔtDj∗−ΔtD(j−1)∗,Vj−ΔtVj∗−ΔtV(j−1)∗)]×ΣIAn+1=+α(Δt)α2AB(α)Γ(α+3)IAn+1=×∑j=2n[tj1−βIA∗(tj,Sj,Ij,IAj,IDj,IRj,ITj,Rj,Dj,Vj)−2tj−11−βIA∗(tj−1,Sj−ΔtSj∗,Ij−ΔtIj∗,IAj−ΔtIAj∗,IDj−ΔtIDj∗,IRj−ΔtIRj∗,ITj−ΔtITj∗,Rj−ΔtRj∗,Dj−ΔtDj∗,Vj−ΔtVj∗)+tj−21−βIA∗(tj−2,Sj−ΔtSj∗−ΔtS(j−1)∗,Ij−ΔtIj∗−ΔtI(j−1)∗,IAj−ΔtIAj∗−ΔtIA(j−1)∗,IDj−ΔtIDj∗−ΔtID(j−1)∗,IRj−ΔtIRj∗−ΔtIR(j−1)∗,ITj−ΔtITj∗−ΔtIT(j−1)∗,Rj−ΔtRj∗−ΔtR(j−1)∗,Dj−ΔtDj∗−ΔtD(j−1)∗,Vj−ΔtVj∗−ΔtV(j−1)∗)]×Δ,IDn+1=1−αAB(α)tn+11−βID∗(tn+1,Sn+ΔtSn∗,In+ΔtIn∗,IAn+ΔtIAn∗,IDn+ΔtIDn∗,IRn+ΔtIRn∗,ITn+ΔtITn∗,Rn+ΔtRn∗,Dn+ΔtDn∗,Vn+ΔtVn∗)IDn+1=+α(Δt)αAB(α)Γ(α+1)IDn+1=×∑j=2ntj−21−βID∗(tj−2,Sj−ΔtSj∗−ΔtS(j−1)∗,Ij−ΔtIj∗−ΔtI(j−1)∗,IAj−ΔtIAj∗−ΔtIA(j−1)∗,IDj−ΔtIDj∗−ΔtID(j−1)∗,IRj−ΔtIRj∗−ΔtIR(j−1)∗,ITj−ΔtITj∗−ΔtIT(j−1)∗,Rj−ΔtRj∗−ΔtR(j−1)∗,Dj−ΔtDj∗−ΔtD(j−1)∗,Vj−ΔtVj∗−ΔtV(j−1)∗)×ΠIDn+1=+α(Δt)αAB(α)Γ(α+2)IDn+1=×∑j=2n[tj−11−βID∗(tj−1,Sj−ΔtSj∗,Ij−ΔtIj∗,IAj−ΔtIAj∗,IDj−ΔtIDj∗,IRj−ΔtIRj∗,ITj−ΔtITj∗,Rj−ΔtRj∗,Dj−ΔtDj∗,Vj−ΔtVj∗)−tj−21−βID∗(tj−2,Sj−ΔtSj∗−ΔtS(j−1)∗,Ij−ΔtIj∗−ΔtI(j−1)∗,IAj−ΔtIAj∗−ΔtIA(j−1)∗,IDj−ΔtIDj∗−ΔtID(j−1)∗,IRj−ΔtIRj∗−ΔtIR(j−1)∗,ITj−ΔtITj∗−ΔtIT(j−1)∗,Rj−ΔtRj∗−ΔtR(j−1)∗,Dj−ΔtDj∗−ΔtD(j−1)∗,Vj−ΔtVj∗−ΔtV(j−1)∗)]×ΣIDn+1=+α(Δt)α2AB(α)Γ(α+3)IDn+1=×∑j=2n[tj1−βID∗(tj,Sj,Ij,IAj,IDj,IRj,ITj,Rj,Dj,Vj)−2tj−11−βID∗(tj−1,Sj−ΔtSj∗,Ij−ΔtIj∗,IAj−ΔtIAj∗,IDj−ΔtIDj∗,IRj−ΔtIRj∗,ITj−ΔtITj∗,Rj−ΔtRj∗,Dj−ΔtDj∗,Vj−ΔtVj∗)+tj−21−βID∗(tj−2,Sj−ΔtSj∗−ΔtS(j−1)∗,Ij−ΔtIj∗−ΔtI(j−1)∗,IAj−ΔtIAj∗−ΔtIA(j−1)∗,IDj−ΔtIDj∗−ΔtID(j−1)∗,IRj−ΔtIRj∗−ΔtIR(j−1)∗,ITj−ΔtITj∗−ΔtIT(j−1)∗,Rj−ΔtRj∗−ΔtR(j−1)∗,Dj−ΔtDj∗−ΔtD(j−1)∗,Vj−ΔtVj∗−ΔtV(j−1)∗)]×Δ,IRn+1=1−αAB(α)tn+11−βIR∗(tn+1,Sn+ΔtSn∗,In+ΔtIn∗,IAn+ΔtIAn∗,IDn+ΔtIDn∗,IRn+ΔtIRn∗,ITn+ΔtITn∗,Rn+ΔtRn∗,Dn+ΔtDn∗,Vn+ΔtVn∗)+α(Δt)αAB(α)Γ(α+1)×∑j=2ntj−21−βIR∗(tj−2,Sj−ΔtSj∗−ΔtS(j−1)∗,Ij−ΔtIj∗−ΔtI(j−1)∗,IAj−ΔtIAj∗−ΔtIA(j−1)∗,IDj−ΔtIDj∗−ΔtID(j−1)∗,IRj−ΔtIRj∗−ΔtIR(j−1)∗,ITj−ΔtITj∗−ΔtIT(j−1)∗,Rj−ΔtRj∗−ΔtR(j−1)∗,Dj−ΔtDj∗−ΔtD(j−1)∗,Vj−ΔtVj∗−ΔtV(j−1)∗)×Π+α(Δt)αAB(α)Γ(α+2)×∑j=2n[tj−11−βIR∗(tj−1,Sj−ΔtSj∗,Ij−ΔtIj∗,IAj−ΔtIAj∗,IDj−ΔtIDj∗,IRj−ΔtIRj∗,ITj−ΔtITj∗,Rj−ΔtRj∗,Dj−ΔtDj∗,Vj−ΔtVj∗)−tj−21−βIR∗(tj−2,Sj−ΔtSj∗−ΔtS(j−1)∗,Ij−ΔtIj∗−ΔtI(j−1)∗,IAj−ΔtIAj∗−ΔtIA(j−1)∗,IDj−ΔtIDj∗−ΔtID(j−1)∗,IRj−ΔtIRj∗−ΔtIR(j−1)∗,ITj−ΔtITj∗−ΔtIT(j−1)∗,Rj−ΔtRj∗−ΔtR(j−1)∗,Dj−ΔtDj∗−ΔtD(j−1)∗,Vj−ΔtVj∗−ΔtV(j−1)∗)]×Σ+α(Δt)α2AB(α)Γ(α+3)×∑j=2n[tj1−βIR∗(tj,Sj,Ij,IAj,IDj,IRj,ITj,Rj,Dj,Vj)−2tj−11−βIR∗(tj−1,Sj−ΔtSj∗,Ij−ΔtIj∗,IAj−ΔtIAj∗,IDj−ΔtIDj∗,IRj−ΔtIRj∗,ITj−ΔtITj∗,Rj−ΔtRj∗,Dj−ΔtDj∗,Vj−ΔtVj∗)+tj−21−βIR∗(tj−2,Sj−ΔtSj∗−ΔtS(j−1)∗,Ij−ΔtIj∗−ΔtI(j−1)∗,IAj−ΔtIAj∗−ΔtIA(j−1)∗,IDj−ΔtIDj∗−ΔtID(j−1)∗,IRj−ΔtIRj∗−ΔtIR(j−1)∗,ITj−ΔtITj∗−ΔtIT(j−1)∗,Rj−ΔtRj∗−ΔtR(j−1)∗,Dj−ΔtDj∗−ΔtD(j−1)∗,Vj−ΔtVj∗−ΔtV(j−1)∗)]×Δ,ITn+1=1−αAB(α)tn+11−βIT∗(tn+1,Sn+ΔtSn∗,In+ΔtIn∗,IAn+ΔtIAn∗IDn+ΔtIDn∗,IRn+ΔtIRn∗,ITn+ΔtITn∗,Rn+ΔtRn∗,Dn+ΔtDn∗,Vn+ΔtVn∗)ITn+1=+α(Δt)αAB(α)Γ(α+1)ITn+1=×∑j=2ntj−21−βIT∗(tj−2,Sj−ΔtSj∗−ΔtS(j−1)∗,Ij−ΔtIj∗−ΔtI(j−1)∗,IAj−ΔtIAj∗−ΔtIA(j−1)∗,IDj−ΔtIDj∗−ΔtID(j−1)∗,IRj−ΔtIRj∗−ΔtIR(j−1)∗,ITj−ΔtITj∗−ΔtIT(j−1)∗,Rj−ΔtRj∗−ΔtR(j−1)∗,Dj−ΔtDj∗−ΔtD(j−1)∗,Vj−ΔtVj∗−ΔtV(j−1)∗)×ΠITn+1=+α(Δt)αAB(α)Γ(α+2)ITn+1=×∑j=2n[tj−11−βIT∗(tj−1,Sj−ΔtSj∗,Ij−ΔtIj∗,IAj−ΔtIAj∗,IDj−ΔtIDj∗,IRj−ΔtIRj∗,ITj−ΔtITj∗,Rj−ΔtRj∗,Dj−ΔtDj∗,Vj−ΔtVj∗)−tj−21−βIT∗(tj−2,Sj−ΔtSj∗−ΔtS(j−1)∗,Ij−ΔtIj∗−ΔtI(j−1)∗,IAj−ΔtIAj∗−ΔtIA(j−1)∗,IDj−ΔtIDj∗−ΔtID(j−1)∗,IRj−ΔtIRj∗−ΔtIR(j−1)∗,ITj−ΔtITj∗−ΔtIT(j−1)∗,Rj−ΔtRj∗−ΔtR(j−1)∗,Dj−ΔtDj∗−ΔtD(j−1)∗,Vj−ΔtVj∗−ΔtV(j−1)∗)]×ΣITn+1=+α(Δt)α2AB(α)Γ(α+3)ITn+1=×∑j=2n[tj1−βIT∗(tj,Sj,Ij,IAj,IDj,IRj,ITj,Rj,Dj,Vj)−2tj−121−βIT∗(tj−1,Sj−ΔtSj∗,Ij−ΔtIj∗,IAj−ΔtIAj∗,IDj−ΔtIDj∗,IRj−ΔtIRj∗,ITj−ΔtITj∗,Rj−ΔtRj∗,Dj−ΔtDj∗,Vj−ΔtVj∗)+tj−21−βIT∗(tj−2,Sj−ΔtSj∗−ΔtS(j−1)∗,Ij−ΔtIj∗−ΔtI(j−1)∗,IAj−ΔtIAj∗−ΔtIA(j−1)∗,IDj−ΔtIDj∗−ΔtID(j−1)∗,IRj−ΔtIRj∗−ΔtIR(j−1)∗,ITj−ΔtITj∗−ΔtIT(j−1)∗,Rj−ΔtRj∗−ΔtR(j−1)∗,Dj−ΔtDj∗−ΔtD(j−1)∗,Vj−ΔtVj∗−ΔtV(j−1)∗)]×Δ,Rn+1=1−αAB(α)tn+11−βR∗(tn+1,Sn+ΔtSn∗,In+ΔtIn∗,IAn+ΔtIAn∗,IDn+ΔtIDn∗,IRn+ΔtIRn∗,ITn+ΔtITn∗,Rn+ΔtRn∗,Dn+ΔtDn∗,Vn+ΔtVn∗)Rn+1=+α(Δt)αAB(α)Γ(α+1)Rn+1=×∑j=2ntj−21−βR∗(tj−2,Sj−ΔtSj∗−ΔtS(j−1)∗,Ij−ΔtIj∗−ΔtI(j−1)∗,IAj−ΔtIAj∗−ΔtIA(j−1)∗,IDj−ΔtIDj∗−ΔtID(j−1)∗,IRj−ΔtIRj∗−ΔtIR(j−1)∗,ITj−ΔtITj∗−ΔtIT(j−1)∗,Rj−ΔtRj∗−ΔtR(j−1)∗,Dj−ΔtDj∗−ΔtD(j−1)∗,Vj−ΔtVj∗−ΔtV(j−1)∗)×ΠRn+1=+α(Δt)αAB(α)Γ(α+2)Rn+1=×∑j=2n[tj−11−βR∗(tj−1,Sj−ΔtSj∗,Ij−ΔtIj∗,IAj−ΔtIAj∗,IDj−ΔtIDj∗,IRj−ΔtIRj∗,ITj−ΔtITj∗,Rj−ΔtRj∗,Dj−ΔtDj∗,Vj−ΔtVj∗)−tj−21−βR∗(tj−2,Sj−ΔtSj∗−ΔtS(j−1)∗,Ij−ΔtIj∗−ΔtI(j−1)∗,IAj−ΔtIAj∗−ΔtIA(j−1)∗,IDj−ΔtIDj∗−ΔtID(j−1)∗,IRj−ΔtIRj∗−ΔtIR(j−1)∗,ITj−ΔtITj∗−ΔtIT(j−1)∗,Rj−ΔtRj∗−ΔtR(j−1)∗,Dj−ΔtDj∗−ΔtD(j−1)∗,Vj−ΔtVj∗−ΔtV(j−1)∗)]×ΣRn+1=+α(Δt)α2AB(α)Γ(α+3)Rn+1=×∑j=2n[tj1−βR∗(tj,Sj,Ij,IAj,IDj,IRj,ITj,Rj,Dj,Vj)−2tj−11−βR∗(tj−1,Sj−ΔtSj∗,Ij−ΔtIj∗,IAj−ΔtIAj∗,IDj−ΔtIDj∗,IRj−ΔtIRj∗,ITj−ΔtITj∗,Rj−ΔtRj∗,Dj−ΔtDj∗,Vj−ΔtVj∗)+tj−21−βR∗(tj−2,Sj−ΔtSj∗−ΔtS(j−1)∗,Ij−ΔtIj∗−ΔtI(j−1)∗,IAj−ΔtIAj∗−ΔtIA(j−1)∗,IDj−ΔtIDj∗−ΔtID(j−1)∗,IRj−ΔtIRj∗−ΔtIR(j−1)∗,ITj−ΔtITj∗−ΔtIT(j−1)∗,Rj−ΔtRj∗−ΔtR(j−1)∗,Dj−ΔtDj∗−ΔtD(j−1)∗,Vj−ΔtVj∗−ΔtV(j−1)∗)]×Δ,Dn+1=1−αAB(α)tn+11−βD∗(tn+1,Sn+ΔtSn∗,In+ΔtIn∗,IAn+ΔtIAn∗,IDn+ΔtIDn∗,IRn+ΔtIRn∗,ITn+ΔtITn∗,Rn+ΔtRn∗,Dn+ΔtDn∗,Vn+ΔtVn∗)Dn+1=+α(Δt)αAB(α)Γ(α+1)Dn+1=×∑j=2ntj−21−βD∗(tj−2,Sj−ΔtSj∗−ΔtS(j−1)∗,Ij−ΔtIj∗−ΔtI(j−1)∗,IAj−ΔtIAj∗−ΔtIA(j−1)∗,IDj−ΔtIDj∗−ΔtID(j−1)∗,IRj−ΔtIRj∗−ΔtIR(j−1)∗,ITj−ΔtITj∗−ΔtIT(j−1)∗,Rj−ΔtRj∗−ΔtR(j−1)∗,Dj−ΔtDj∗−ΔtD(j−1)∗,Vj−ΔtVj∗−ΔtV(j−1)∗)×ΠDn+1=+α(Δt)αAB(α)Γ(α+2)Dn+1=×∑j=2n[tj−11−βD∗(tj−1,Sj−ΔtSj∗,Ij−ΔtIj∗,IAj−ΔtIAj∗,IDj−ΔtIDj∗,IRj−ΔtIRj∗,ITj−ΔtITj∗,Rj−ΔtRj∗,Dj−ΔtDj∗,Vj−ΔtVj∗)−tj−21−βD∗(tj−2,Sj−ΔtSj∗−ΔtS(j−1)∗,Ij−ΔtIj∗−ΔtI(j−1)∗,IAj−ΔtIAj∗−ΔtIA(j−1)∗,IDj−ΔtIDj∗−ΔtID(j−1)∗,IRj−ΔtIRj∗−ΔtIR(j−1)∗,ITj−ΔtITj∗−ΔtIT(j−1)∗,Rj−ΔtRj∗−ΔtR(j−1)∗,Dj−ΔtDj∗−ΔtD(j−1)∗,Vj−ΔtVj∗−ΔtV(j−1)∗)]×ΣDn+1=+α(Δt)α2AB(α)Γ(α+3)Dn+1=×∑j=2n[tj1−βD∗(tj,Sj,Ij,IAj,IDj,IRj,ITj,Rj,Dj,Vj)−2tj−11−βD∗(tj−1,Sj−ΔtSj∗,Ij−ΔtIj∗,IAj−ΔtIAj∗,IDj−ΔtIDj∗,IRj−ΔtIRj∗,ITj−ΔtITj∗,Rj−ΔtRj∗,Dj−ΔtDj∗,Vj−ΔtVj∗)+tj−21−βD∗(tj−2,Sj−ΔtSj∗−ΔtS(j−1)∗,Ij−ΔtIj∗−ΔtI(j−1)∗,IAj−ΔtIAj∗−ΔtIA(j−1)∗,IDj−ΔtIDj∗−ΔtID(j−1)∗,IRj−ΔtIRj∗−ΔtIR(j−1)∗,ITj−ΔtITj∗−ΔtIT(j−1)∗,Rj−ΔtRj∗−ΔtR(j−1)∗,Dj−ΔtDj∗−ΔtD(j−1)∗,Vj−ΔtVj∗−ΔtV(j−1)∗)]×Δ,Vn+1=1−αAB(α)tn+11−βV∗(tn+1,Sn+ΔtSn∗,In+ΔtIn∗,IAn+ΔtIAn∗,IDn+ΔtIDn∗,IRn+ΔtIRn∗,ITn+ΔtITn∗,Rn+ΔtRn∗,Dn+ΔtDn∗,Vn+ΔtVn∗)Vn+1=+α(Δt)αAB(α)Γ(α+1)Vn+1=×∑j=2ntj−21−βV∗(tj−2,Sj−ΔtSj∗−ΔtS(j−1)∗,Ij−ΔtIj∗−ΔtI(j−1)∗,IAj−ΔtIAj∗−ΔtIA(j−1)∗,IDj−ΔtIDj∗−ΔtID(j−1)∗,IRj−ΔtIRj∗−ΔtIR(j−1)∗,ITj−ΔtITj∗−ΔtIT(j−1)∗,Rj−ΔtRj∗−ΔtR(j−1)∗,Dj−ΔtDj∗−ΔtD(j−1)∗,Vj−ΔtVj∗−ΔtV(j−1)∗)×ΠVn+1=+α(Δt)αAB(α)Γ(α+2)Vn+1=×∑j=2n[tj−11−βV∗(tj−1,Sj−ΔtSj∗,Ij−ΔtIj∗,IAj−ΔtIAj∗,IDj−ΔtIDj∗,IRj−ΔtIRj∗,ITj−ΔtITj∗,Rj−ΔtRj∗,Dj−ΔtDj∗,Vj−ΔtVj∗)−tj−21−βV∗(tj−2,Sj−ΔtSj∗−ΔtS(j−1)∗,Ij−ΔtIj∗−ΔtI(j−1)∗,IAj−ΔtIAj∗−ΔtIA(j−1)∗,IDj−ΔtIDj∗−ΔtID(j−1)∗,IRj−ΔtIRj∗−ΔtIR(j−1)∗,ITj−ΔtITj∗−ΔtIT(j−1)∗,Rj−ΔtRj∗−ΔtR(j−1)∗,Dj−ΔtDj∗−ΔtD(j−1)∗,Vj−ΔtVj∗−ΔtV(j−1)∗)]×ΣVn+1=+α(Δt)α2AB(α)Γ(α+3)Vn+1=×∑j=2n[tj1−βV∗(tj,Sj,Ij,IAj,IDj,IRj,ITj,Rj,Dj,Vj)−2tj−11−βV∗(tj−1,Sj−ΔtSj∗,Ij−ΔtIj∗,IAj−ΔtIAj∗,IDj−ΔtIDj∗,IRj−ΔtIRj∗,ITj−ΔtITj∗,Rj−ΔtRj∗,Dj−ΔtDj∗,Vj−ΔtVj∗)+tj−21−βV∗(tj−2,Sj−ΔtSj∗−ΔtS(j−1)∗,Ij−ΔtIj∗−ΔtI(j−1)∗,IAj−ΔtIAj∗−ΔtIA(j−1)∗,IDj−ΔtIDj∗−ΔtID(j−1)∗,IRj−ΔtIRj∗−ΔtIR(j−1)∗,ITj−ΔtITj∗−ΔtIT(j−1)∗,Rj−ΔtRj∗−ΔtR(j−1)∗,Dj−ΔtDj∗−ΔtD(j−1)∗,Vj−ΔtVj∗−ΔtV(j−1)∗)]×Δ.

For the power-law kernel, we can have the following:

135 Sn+1=(Δt)αΓ(α+1)×∑j=2ntj−21−βS∗(tj−2,Sj−ΔtSj∗−ΔtS(j−1)∗,Ij−ΔtIj∗−ΔtI(j−1)∗,IAj−ΔtIAj∗−ΔtIA(j−1)∗,IDj−ΔtIDj∗−ΔtID(j−1)∗,IRj−ΔtIRj∗−ΔtIR(j−1)∗,ITj−ΔtITj∗−ΔtIT(j−1)∗,Rj−ΔtRj∗−ΔtR(j−1)∗,Dj−ΔtDj∗−ΔtD(j−1)∗,Vj−ΔtVj∗−ΔtV(j−1)∗)×Π+(Δt)αΓ(α+2)×∑j=2n[tj−11−βS∗(tj−1,Sj−ΔtSj∗,Ij−ΔtIj∗,IAj−ΔtIAj∗,IDj−ΔtIDj∗,IRj−ΔtIRj∗,ITj−ΔtITj∗,Rj−ΔtRj∗,Dj−ΔtDj∗,Vj−ΔtVj∗)−tj−21−βS∗(tj−2,Sj−ΔtSj∗−ΔtS(j−1)∗,Ij−ΔtIj∗−ΔtI(j−1)∗,IAj−ΔtIAj∗−ΔtIA(j−1)∗,IDj−ΔtIDj∗−ΔtID(j−1)∗,IRj−ΔtIRj∗−ΔtIR(j−1)∗,ITj−ΔtITj∗−ΔtIT(j−1)∗,Rj−ΔtRj∗−ΔtR(j−1)∗,Dj−ΔtDj∗−ΔtD(j−1)∗,Vj−ΔtVj∗−ΔtV(j−1)∗)]×Σ+(Δt)α2Γ(α+3)×∑j=2n[tj1−βS∗(tj,Sj,Ij,IAj,IDj,IRj,ITj,Rj,Dj,Vj)−2tj−11−βS∗(tj−1,Sj−ΔtSj∗,Ij−ΔtIj∗,IAj−ΔtIAj∗,IDj−ΔtIDj∗,IRj−ΔtIRj∗,ITj−ΔtITj∗,Rj−ΔtRj∗,Dj−ΔtDj∗,Vj−ΔtVj∗)+tj−21−βS∗(tj−2,Sj−ΔtSj∗−ΔtS(j−1)∗,Ij−ΔtIj∗−ΔtI(j−1)∗,IAj−ΔtIAj∗−ΔtIA(j−1)∗,IDj−ΔtIDj∗−ΔtID(j−1)∗,IRj−ΔtIRj∗−ΔtIR(j−1)∗,ITj−ΔtITj∗−ΔtIT(j−1)∗,Rj−ΔtRj∗−ΔtR(j−1)∗,Dj−ΔtDj∗−ΔtD(j−1)∗,Vj−ΔtVj∗−ΔtV(j−1)∗)]×Δ,In+1=(Δt)αΓ(α+1)∑j=2ntj−21−βI∗(tj−2,Sj−ΔtSj∗−ΔtS(j−1)∗,Ij−ΔtIj∗−ΔtI(j−1)∗,IAj−ΔtIAj∗−ΔtIA(j−1)∗,IDj−ΔtIDj∗−ΔtID(j−1)∗,IRj−ΔtIRj∗−ΔtIR(j−1)∗,ITj−ΔtITj∗−ΔtIT(j−1)∗,Rj−ΔtRj∗−ΔtR(j−1)∗,Dj−ΔtDj∗−ΔtD(j−1)∗,Vj−ΔtVj∗−ΔtV(j−1)∗)×ΠIn+1=+(Δt)αΓ(α+2)In+1=×∑j=2n[tj−11−βI∗(tj−1,Sj−ΔtSj∗,Ij−ΔtIj∗,IAj−ΔtIAj∗,IDj−ΔtIDj∗,IRj−ΔtIRj∗,ITj−ΔtITj∗,Rj−ΔtRj∗,Dj−ΔtDj∗,Vj−ΔtVj∗)−tj−21−βI∗(tj−2,Sj−ΔtSj∗−ΔtS(j−1)∗,Ij−ΔtIj∗−ΔtI(j−1)∗,IAj−ΔtIAj∗−ΔtIA(j−1)∗,IDj−ΔtIDj∗−ΔtID(j−1)∗,IRj−ΔtIRj∗−ΔtIR(j−1)∗,ITj−ΔtITj∗−ΔtIT(j−1)∗,Rj−ΔtRj∗−ΔtR(j−1)∗,Dj−ΔtDj∗−ΔtD(j−1)∗,Vj−ΔtVj∗−ΔtV(j−1)∗)]×ΣIn+1=+(Δt)α2Γ(α+3)In+1=×∑j=2n[tj1−βI∗(tj,Sj,Ij,IAj,IDj,IRj,ITj,Rj,Dj,Vj)−2tj−11−βI∗(tj−1,Sj−ΔtSj∗,Ij−ΔtIj∗,IAj−ΔtIAj∗,IDj−ΔtIDj∗,IRj−ΔtIRj∗,ITj−ΔtITj∗,Rj−ΔtRj∗,Dj−ΔtDj∗,Vj−ΔtVj∗)+tj−21−βI∗(tj−2,Sj−ΔtSj∗−ΔtS(j−1)∗,Ij−ΔtIj∗−ΔtI(j−1)∗,IAj−ΔtIAj∗−ΔtIA(j−1)∗,IDj−ΔtIDj∗−ΔtID(j−1)∗,IRj−ΔtIRj∗−ΔtIR(j−1)∗,ITj−ΔtITj∗−ΔtIT(j−1)∗,Rj−ΔtRj∗−ΔtR(j−1)∗,Dj−ΔtDj∗−ΔtD(j−1)∗,Vj−ΔtVj∗−ΔtV(j−1)∗)]×Δ,IAn+1=(Δt)αΓ(α+1)∑j=2ntj−21−βIA∗(tj−2,Sj−ΔtSj∗−ΔtS(j−1)∗,Ij−ΔtIj∗−ΔtI(j−1)∗,IAj−ΔtIAj∗−ΔtIA(j−1)∗,IDj−ΔtIDj∗−ΔtID(j−1)∗,IRj−ΔtIRj∗−ΔtIR(j−1)∗,ITj−ΔtITj∗−ΔtIT(j−1)∗,Rj−ΔtRj∗−ΔtR(j−1)∗,Dj−ΔtDj∗−ΔtD(j−1)∗,Vj−ΔtVj∗−ΔtV(j−1)∗)×ΠIAn+1=+(Δt)αΓ(α+2)IAn+1=×∑j=2n[tj−11−βIA∗(tj−1,Sj−ΔtSj∗,Ij−ΔtIj∗,IAj−ΔtIAj∗,IDj−ΔtIDj∗,IRj−ΔtIRj∗,ITj−ΔtITj∗,Rj−ΔtRj∗,Dj−ΔtDj∗,Vj−ΔtVj∗)−tj−21−βIA∗(tj−2,Sj−ΔtSj∗−ΔtS(j−1)∗,Ij−ΔtIj∗−ΔtI(j−1)∗,IAj−ΔtIAj∗−ΔtIA(j−1)∗,IDj−ΔtIDj∗−ΔtID(j−1)∗,IRj−ΔtIRj∗−ΔtIR(j−1)∗,ITj−ΔtITj∗−ΔtIT(j−1)∗,Rj−ΔtRj∗−ΔtR(j−1)∗,Dj−ΔtDj∗−ΔtD(j−1)∗,Vj−ΔtVj∗−ΔtV(j−1)∗)]×ΣIAn+1=+(Δt)α2Γ(α+3)IAn+1=×∑j=2n[tj1−βIA∗(tj,Sj,Ij,IAj,IDj,IRj,ITj,Rj,Dj,Vj)−2tj−11−βIA∗(tj−1,Sj−ΔtSj∗,Ij−ΔtIj∗,IAj−ΔtIAj∗,IDj−ΔtIDj∗,IRj−ΔtIRj∗,ITj−ΔtITj∗,Rj−ΔtRj∗,Dj−ΔtDj∗,Vj−ΔtVj∗)+tj−21−βIA∗(tj−2,Sj−ΔtSj∗−ΔtS(j−1)∗,Ij−ΔtIj∗−ΔtI(j−1)∗,IAj−ΔtIAj∗−ΔtIA(j−1)∗,IDj−ΔtIDj∗−ΔtID(j−1)∗,IRj−ΔtIRj∗−ΔtIR(j−1)∗,ITj−ΔtITj∗−ΔtIT(j−1)∗,Rj−ΔtRj∗−ΔtR(j−1)∗,Dj−ΔtDj∗−ΔtD(j−1)∗,Vj−ΔtVj∗−ΔtV(j−1)∗)]×Δ,IDn+1=(Δt)αΓ(α+1)∑j=2ntj−21−βID∗(tj−2,Sj−ΔtSj∗−ΔtS(j−1)∗,Ij−ΔtIj∗−ΔtI(j−1)∗,IAj−ΔtIAj∗−ΔtIA(j−1)∗,IDj−ΔtIDj∗−ΔtID(j−1)∗,IRj−ΔtIRj∗−ΔtIR(j−1)∗,ITj−ΔtITj∗−ΔtIT(j−1)∗,Rj−ΔtRj∗−ΔtR(j−1)∗,Dj−ΔtDj∗−ΔtD(j−1)∗,Vj−ΔtVj∗−ΔtV(j−1)∗)×ΠIDn+1=+(Δt)αΓ(α+2)IDn+1=×∑j=2n[tj−11−βID∗(tj−1,Sj−ΔtSj∗,Ij−ΔtIj∗,IAj−ΔtIAj∗,IDj−ΔtIDj∗,IRj−ΔtIRj∗,ITj−ΔtITj∗,Rj−ΔtRj∗,Dj−ΔtDj∗,Vj−ΔtVj∗)−tj−21−βID∗(tj−2,Sj−ΔtSj∗−ΔtS(j−1)∗,Ij−ΔtIj∗−ΔtI(j−1)∗,IAj−ΔtIAj∗−ΔtIA(j−1)∗,IDj−ΔtIDj∗−ΔtID(j−1)∗,IRj−ΔtIRj∗−ΔtIR(j−1)∗,ITj−ΔtITj∗−ΔtIT(j−1)∗,Rj−ΔtRj∗−ΔtR(j−1)∗,Dj−ΔtDj∗−ΔtD(j−1)∗,Vj−ΔtVj∗−ΔtV(j−1)∗)]×ΣIDn+1=+(Δt)α2Γ(α+3)IDn+1=×∑j=2n[tj1−βID∗(tj,Sj,Ij,IAj,IDj,IRj,ITj,Rj,Dj,Vj)−2tj−11−βID∗(tj−1,Sj−ΔtSj∗,Ij−ΔtIj∗,IAj−ΔtIAj∗,IDj−ΔtIDj∗,IRj−ΔtIRj∗,ITj−ΔtITj∗,Rj−ΔtRj∗,Dj−ΔtDj∗,Vj−ΔtVj∗)+tj−21−βID∗(tj−2,Sj−ΔtSj∗−ΔtS(j−1)∗,Ij−ΔtIj∗−ΔtI(j−1)∗,IAj−ΔtIAj∗−ΔtIA(j−1)∗,IDj−ΔtIDj∗−ΔtID(j−1)∗,IRj−ΔtIRj∗−ΔtIR(j−1)∗,ITj−ΔtITj∗−ΔtIT(j−1)∗,Rj−ΔtRj∗−ΔtR(j−1)∗,Dj−ΔtDj∗−ΔtD(j−1)∗,Vj−ΔtVj∗−ΔtV(j−1)∗)]×Δ,IRn+1=(Δt)αΓ(α+1)∑j=2ntj−21−βIR∗(tj−2,Sj−ΔtSj∗−ΔtS(j−1)∗,Ij−ΔtIj∗−ΔtI(j−1)∗,IAj−ΔtIAj∗−ΔtIA(j−1)∗,IDj−ΔtIDj∗−ΔtID(j−1)∗,IRj−ΔtIRj∗−ΔtIR(j−1)∗,ITj−ΔtITj∗−ΔtIT(j−1)∗,Rj−ΔtRj∗−ΔtR(j−1)∗,Dj−ΔtDj∗−ΔtD(j−1)∗,Vj−ΔtVj∗−ΔtV(j−1)∗)×Π+(Δt)αΓ(α+2)×∑j=2n[tj−121−βIR∗(tj−1,Sj−ΔtSj∗,Ij−ΔtIj∗,IAj−ΔtIAj∗,IDj−ΔtIDj∗,IRj−ΔtIRj∗,ITj−ΔtITj∗,Rj−ΔtRj∗,Dj−ΔtDj∗,Vj−ΔtVj∗)−tj−21−βIR∗(tj−2,Sj−ΔtSj∗−ΔtS(j−1)∗,Ij−ΔtIj∗−ΔtI(j−1)∗,IAj−ΔtIAj∗−ΔtIA(j−1)∗,IDj−ΔtIDj∗−ΔtID(j−1)∗,IRj−ΔtIRj∗−ΔtIR(j−1)∗,ITj−ΔtITj∗−ΔtIT(j−1)∗,Rj−ΔtRj∗−ΔtR(j−1)∗,Dj−ΔtDj∗−ΔtD(j−1)∗,Vj−ΔtVj∗−ΔtV(j−1)∗)]×Σ+(Δt)α2Γ(α+3)×∑j=2n[tj1−βIR∗(tj,Sj,Ij,IAj,IDj,IRj,ITj,Rj,Dj,Vj)−2tj−11−βIR∗(tj−1,Sj−ΔtSj∗,Ij−ΔtIj∗,IAj−ΔtIAj∗,IDj−ΔtIDj∗,IRj−ΔtIRj∗,ITj−ΔtITj∗,Rj−ΔtRj∗,Dj−ΔtDj∗,Vj−ΔtVj∗)+tj−21−βIR∗(tj−2,Sj−ΔtSj∗−ΔtS(j−1)∗,Ij−ΔtIj∗−ΔtI(j−1)∗,IAj−ΔtIAj∗−ΔtIA(j−1)∗,IDj−ΔtIDj∗−ΔtID(j−1)∗,IRj−ΔtIRj∗−ΔtIR(j−1)∗,ITj−ΔtITj∗−ΔtIT(j−1)∗,Rj−ΔtRj∗−ΔtR(j−1)∗,Dj−ΔtDj∗−ΔtD(j−1)∗,Vj−ΔtVj∗−ΔtV(j−1)∗)]×Δ,ITn+1=(Δt)αΓ(α+1)∑j=2ntj−21−βIT∗(tj−2,Sj−ΔtSj∗−ΔtS(j−1)∗,Ij−ΔtIj∗−ΔtI(j−1)∗,IAj−ΔtIAj∗−ΔtIA(j−1)∗,IDj−ΔtIDj∗−ΔtID(j−1)∗,IRj−ΔtIRj∗−ΔtIR(j−1)∗,ITj−ΔtITj∗−ΔtIT(j−1)∗,Rj−ΔtRj∗−ΔtR(j−1)∗,Dj−ΔtDj∗−ΔtD(j−1)∗,Vj−ΔtVj∗−ΔtV(j−1)∗)×ΠITn+1=+α(Δt)αΓ(α+2)ITn+1=×∑j=2n[tj−11−βIT∗(tj−1,Sj−ΔtSj∗,Ij−ΔtIj∗,IAj−ΔtIAj∗,IDj−ΔtIDj∗,IRj−ΔtIRj∗,ITj−ΔtITj∗,Rj−ΔtRj∗,Dj−ΔtDj∗,Vj−ΔtVj∗)−tj−21−βIT∗(tj−2,Sj−ΔtSj∗−ΔtS(j−1)∗,Ij−ΔtIj∗−ΔtI(j−1)∗,IAj−ΔtIAj∗−ΔtIA(j−1)∗,IDj−ΔtIDj∗−ΔtID(j−1)∗,IRj−ΔtIRj∗−ΔtIR(j−1)∗,ITj−ΔtITj∗−ΔtIT(j−1)∗,Rj−ΔtRj∗−ΔtR(j−1)∗,Dj−ΔtDj∗−ΔtD(j−1)∗,Vj−ΔtVj∗−ΔtV(j−1)∗)]×ΣITn+1=+(Δt)α2Γ(α+3)ITn+1=×∑j=2n[tj1−βIT∗(tj,Sj,Ij,IAj,IDj,IRj,ITj,Rj,Dj,Vj)−2tj−11−βIT∗(tj−1,Sj−ΔtSj∗,Ij−ΔtIj∗,IAj−ΔtIAj∗,IDj−ΔtIDj∗,IRj−ΔtIRj∗,ITj−ΔtITj∗,Rj−ΔtRj∗,Dj−ΔtDj∗,Vj−ΔtVj∗)+tj−21−βIT∗(tj−2,Sj−ΔtSj∗−ΔtS(j−1)∗,Ij−ΔtIj∗−ΔtI(j−1)∗,IAj−ΔtIAj∗−ΔtIA(j−1)∗,IDj−ΔtIDj∗−ΔtID(j−1)∗,IRj−ΔtIRj∗−ΔtIR(j−1)∗,ITj−ΔtITj∗−ΔtIT(j−1)∗,Rj−ΔtRj∗−ΔtR(j−1)∗,Dj−ΔtDj∗−ΔtD(j−1)∗,Vj−ΔtVj∗−ΔtV(j−1)∗)]×Δ,Rn+1=(Δt)αΓ(α+1)∑j=2ntj−21−βR∗(tj−2,Sj−ΔtSj∗−ΔtS(j−1)∗,Ij−ΔtIj∗−ΔtI(j−1)∗,IAj−ΔtIAj∗−ΔtIA(j−1)∗,IDj−ΔtIDj∗−ΔtID(j−1)∗,IRj−ΔtIRj∗−ΔtIR(j−1)∗,ITj−ΔtITj∗−ΔtIT(j−1)∗,Rj−ΔtRj∗−ΔtR(j−1)∗,Dj−ΔtDj∗−ΔtD(j−1)∗,Vj−ΔtVj∗−ΔtV(j−1)∗)×ΠRn+1=+(Δt)αΓ(α+2)Rn+1=×∑j=2n[tj−11−βR∗(tj−1,Sj−ΔtSj∗,Ij−ΔtIj∗,IAj−ΔtIAj∗,IDj−ΔtIDj∗,IRj−ΔtIRj∗,ITj−ΔtITj∗,Rj−ΔtRj∗,Dj−ΔtDj∗,Vj−ΔtVj∗)−tj−21−βR∗(tj−2,Sj−ΔtSj∗−ΔtS(j−1)∗,Ij−ΔtIj∗−ΔtI(j−1)∗,IAj−ΔtIAj∗−ΔtIA(j−1)∗,IDj−ΔtIDj∗−ΔtID(j−1)∗,IRj−ΔtIRj∗−ΔtIR(j−1)∗,ITj−ΔtITj∗−ΔtIT(j−1)∗,Rj−ΔtRj∗−ΔtR(j−1)∗,Dj−ΔtDj∗−ΔtD(j−1)∗,Vj−ΔtVj∗−ΔtV(j−1)∗)]×ΣRn+1=+(Δt)α2Γ(α+3)Rn+1=×∑j=2n[tj1−βR∗(tj,Sj,Ij,IAj,IDj,IRj,ITj,Rj,Dj,Vj)−2tj−11−βR∗(tj−1,Sj−ΔtSj∗,Ij−ΔtIj∗,IAj−ΔtIAj∗,IDj−ΔtIDj∗,IRj−ΔtIRj∗,ITj−ΔtITj∗,Rj−ΔtRj∗,Dj−ΔtDj∗,Vj−ΔtVj∗)+tj−21−βR∗(tj−2,Sj−ΔtSj∗−ΔtS(j−1)∗,Ij−ΔtIj∗−ΔtI(j−1)∗,IAj−ΔtIAj∗−ΔtIA(j−1)∗,IDj−ΔtIDj∗−ΔtID(j−1)∗,IRj−ΔtIRj∗−ΔtIR(j−1)∗,ITj−ΔtITj∗−ΔtIT(j−1)∗,Rj−ΔtRj∗−ΔtR(j−1)∗,Dj−ΔtDj∗−ΔtD(j−1)∗,Vj−ΔtVj∗−ΔtV(j−1)∗)]×Δ,Dn+1=(Δt)αΓ(α+1)∑j=2ntj−21−βD∗(tj−2,Sj−ΔtSj∗−ΔtS(j−1)∗,Ij−ΔtIj∗−ΔtI(j−1)∗,IAj−ΔtIAj∗−ΔtIA(j−1)∗,IDj−ΔtIDj∗−ΔtID(j−1)∗,IRj−ΔtIRj∗−ΔtIR(j−1)∗,ITj−ΔtITj∗−ΔtIT(j−1)∗,Rj−ΔtRj∗−ΔtR(j−1)∗,Dj−ΔtDj∗−ΔtD(j−1)∗,Vj−ΔtVj∗−ΔtV(j−1)∗)×Π+(Δt)αΓ(α+2)×∑j=2n[tj−11−βD∗(tj−1,Sj−ΔtSj∗,Ij−ΔtIj∗,IAj−ΔtIAj∗,IDj−ΔtIDj∗,IRj−ΔtIRj∗,ITj−ΔtITj∗,Rj−ΔtRj∗,Dj−ΔtDj∗,Vj−ΔtVj∗)−tj−21−βD∗(tj−2,Sj−ΔtSj∗−ΔtS(j−1)∗,Ij−ΔtIj∗−ΔtI(j−1)∗,IAj−ΔtIAj∗−ΔtIA(j−1)∗,IDj−ΔtIDj∗−ΔtID(j−1)∗,IRj−ΔtIRj∗−ΔtIR(j−1)∗,ITj−ΔtITj∗−ΔtIT(j−1)∗,Rj−ΔtRj∗−ΔtR(j−1)∗,Dj−ΔtDj∗−ΔtD(j−1)∗,Vj−ΔtVj∗−ΔtV(j−1)∗)]×Σ+(Δt)α2Γ(α+3)×∑j=2n[tj1−βD∗(tj,Sj,Ij,IAj,IDj,IRj,ITj,Rj,Dj,Vj)−2tj−11−βD∗(tj−1,Sj−ΔtSj∗,Ij−ΔtIj∗,IAj−ΔtIAj∗,IDj−ΔtIDj∗,IRj−ΔtIRj∗,ITj−ΔtITj∗,Rj−ΔtRj∗,Dj−ΔtDj∗,Vj−ΔtVj∗)+tj−21−βD∗(tj−2,Sj−ΔtSj∗−ΔtS(j−1)∗,Ij−ΔtIj∗−ΔtI(j−1)∗,IAj−ΔtIAj∗−ΔtIA(j−1)∗,IDj−ΔtIDj∗−ΔtID(j−1)∗,IRj−ΔtIRj∗−ΔtIR(j−1)∗,ITj−ΔtITj∗−ΔtIT(j−1)∗,Rj−ΔtRj∗−ΔtR(j−1)∗,Dj−ΔtDj∗−ΔtD(j−1)∗,Vj−ΔtVj∗−ΔtV(j−1)∗)]×Δ,Vn+1=(Δt)αΓ(α+1)∑j=2ntj−21−βV∗(tj−2,Sj−ΔtSj∗−ΔtS(j−1)∗,Ij−ΔtIj∗−ΔtI(j−1)∗,IAj−ΔtIAj∗−ΔtIA(j−1)∗,IDj−ΔtIDj∗−ΔtID(j−1)∗,IRj−ΔtIRj∗−ΔtIR(j−1)∗,ITj−ΔtITj∗−ΔtIT(j−1)∗,Rj−ΔtRj∗−ΔtR(j−1)∗,Dj−ΔtDj∗−ΔtD(j−1)∗,Vj−ΔtVj∗−ΔtV(j−1)∗)×ΠVn+1=+(Δt)αΓ(α+2)Vn+1=×∑j=2n[tj−11−βV∗(tj−1,Sj−ΔtSj∗,Ij−ΔtIj∗,IAj−ΔtIAj∗,IDj−ΔtIDj∗,IRj−ΔtIRj∗,ITj−ΔtITj∗,Rj−ΔtRj∗,Dj−ΔtDj∗,Vj−ΔtVj∗)−tj−21−βV∗(tj−2,Sj−ΔtSj∗−ΔtS(j−1)∗,Ij−ΔtIj∗−ΔtI(j−1)∗,IAj−ΔtIAj∗−ΔtIA(j−1)∗,IDj−ΔtIDj∗−ΔtID(j−1)∗,IRj−ΔtIRj∗−ΔtIR(j−1)∗,ITj−ΔtITj∗−ΔtIT(j−1)∗,Rj−ΔtRj∗−ΔtR(j−1)∗,Dj−ΔtDj∗−ΔtD(j−1)∗,Vj−ΔtVj∗−ΔtV(j−1)∗)]×ΣVn+1=+(Δt)α2Γ(α+3)Vn+1=×∑j=2n[tj1−βV∗(tj,Sj,Ij,IAj,IDj,IRj,ITj,Rj,Dj,Vj)−2tj−11−βV∗(tj−1,Sj−ΔtSj∗,Ij−ΔtIj∗,IAj−ΔtIAj∗,IDj−ΔtIDj∗,IRj−ΔtIRj∗,ITj−ΔtITj∗,Rj−ΔtRj∗,Dj−ΔtDj∗,Vj−ΔtVj∗)+tj−21−βV∗(tj−2,Sj−ΔtSj∗−ΔtS(j−1)∗,Ij−ΔtIj∗−ΔtI(j−1)∗,IAj−ΔtIAj∗−ΔtIA(j−1)∗,IDj−ΔtIDj∗−ΔtID(j−1)∗,IRj−ΔtIRj∗−ΔtIR(j−1)∗,ITj−ΔtITj∗−ΔtIT(j−1)∗,Rj−ΔtRj∗−ΔtR(j−1)∗,Dj−ΔtDj∗−ΔtD(j−1)∗,Vj−ΔtVj∗−ΔtV(j−1)∗)]×Δ.

Now we apply

136 $$\begin{aligned}& {}_{0}^{FFE}D_{t}^{\alpha ,\beta ( t ) }S = S^{\ast } ( t,S,I,I_{A},I_{D},I_{R},I_{T},R,D,V ), \\ & {}_{0}^{FFE}D_{t}^{\alpha ,\beta ( t ) }I = I^{ \ast } ( t,S,I,I_{A},I_{D},I_{R},I_{T},R,D,V ), \\ & {}_{0}^{FFE}D_{t}^{\alpha ,\beta ( t ) }I_{A} = I_{A}^{ \ast } ( t,S,I,I_{A},I_{D},I_{R},I_{T},R,D,V ), \\ & {}_{0}^{FFE}D_{t}^{\alpha ,\beta ( t ) }I_{D} = I_{D}^{ \ast } ( t,S,I,I_{A},I_{D},I_{R},I_{T},R,D,V ), \\ & {}_{0}^{FFE}D_{t}^{\alpha ,\beta ( t ) }I_{R} = I_{R}^{ \ast } ( t,S,I,I_{A},I_{D},I_{R},I_{T},R,D,V ), \\ & {}_{0}^{FFE}D_{t}^{\alpha ,\beta ( t ) }I_{T} = I_{T}^{ \ast } ( t,S,I,I_{A},I_{D},I_{R},I_{T},R,D,V ), \\ & {}_{0}^{FFE}D_{t}^{\alpha ,\beta ( t ) }R = R^{ \ast } ( t,S,I,I_{A},I_{D},I_{R},I_{T},R,D,V ), \\ & {}_{0}^{FFE}D_{t}^{\alpha ,\beta ( t ) }D = D^{ \ast } ( t,S,I,I_{A},I_{D},I_{R},I_{T},R,D,V ), \\ & {}_{0}^{FFE}D_{t}^{\alpha ,\beta ( t ) }V = V^{ \ast } ( t,S,I,I_{A},I_{D},I_{R},I_{T},R,D,V ) . \end{aligned}$$

After applying the fractional integral with exponential kernel and putting the Newton polynomial into these equations, we can solve our model as follows:

137 $$S^{n+1}=S^{n}+\frac{1-\alpha }{M(\alpha )}\left[\begin{array}{c} t_{n+1}^{2-\beta (t_{n+1})}(-\frac{\beta (t_{n+2})-\beta (t_{n+1})}{\Delta t}\ln t_{n+1}+\frac{2-\beta (t_{n+1})}{t_{n+1}})\\ \times S^{*}\left(\begin{array}{c} t_{n+1},S^{n}+\Delta tS^{n*},I^{n}+\Delta tI^{n*},I_{A}^{n}+\Delta tI_{A}^{n*},\\ I_{D}^{n}+\Delta tI_{D}^{n*},I_{R}^{n}+\Delta tI_{R}^{n*},I_{T}^{n}+\Delta tI_{T}^{n*},\\ R^{n}+\Delta tR^{n*},D^{n}+\Delta tD^{n*},V^{n}+\Delta tV^{n*} \end{array}\right)\\ -t_{n}^{2-\beta (t_{n})}(-\frac{\beta (t_{n+1})-\beta (t_{n})}{\Delta t}\ln t_{n}+\frac{2-\beta (t_{n})}{t_{n}})\\ S^{*}\left(t_{n},S^{n},I^{n},I_{A}^{n},I_{D}^{n},I_{R}^{n},I_{T}^{n},R^{n},D^{n},V^{n}\right) \end{array}\right]$$

138 Sn+1=+αM(α){2312tn2−β(tn)(−β(tn+1)−β(tn)Δtlntn+2−β(tn)tn)×S∗(tn,Sn,In,IAn,IDn,IRn,ITn,Rn,Dn,Vn)Δt−43tn−12−β(tn−1)(−β(tn)−β(tn−1)Δtlntn−1+2−β(tn−1)tn−1)×S∗(tn−1,Sn−ΔtSn∗,In−ΔtIn∗,IAn−ΔtIAn∗,IDn−ΔtIDn∗,IRn−ΔtIRn∗,ITn−ΔtITn∗,Rn−ΔtRn∗,Dn−ΔtDn∗,Vn−ΔtVn∗)Δt+512tn−22−β(tn−2)(−β(tn−1)−β(tn−2)Δtlntn−2+2−β(tn−2)tn−2)×S∗(tn−2,Sn−ΔtSn∗−ΔtS(n−1)∗,In−ΔtIn∗−ΔtI(n−1)∗,IAn−ΔtIAn∗−ΔtIA(n−1)∗,IDn−ΔtIDn∗−ΔtID(n−1)∗,IRn−ΔtIRn∗−ΔtIR(n−1)∗,ITn−ΔtITn∗−ΔtIT(n−1)∗,Rn−ΔtRn∗−ΔtR(n−1)∗,Dn−ΔtDn∗−ΔtD(n−1)∗,Vn−ΔtVn∗−ΔtV(n−1)∗)Δt},In+1=In+1−αM(α)×[tn+12−β(tn+1)(−β(tn+2)−β(tn+1)Δtlntn+1+2−β(tn+1)tn+1)×I∗(tn+1,Sn+ΔtSn∗,In+ΔtIn∗,IAn+ΔtIAn∗,IDn+ΔtIDn∗,IRn+ΔtIRn∗,ITn+ΔtITn∗,Rn+ΔtRn∗,Dn+ΔtDn∗,Vn+ΔtVn∗)−tn2−β(tn)(−β(tn+1)−β(tn)Δtlntn+2−β(tn)tn)I∗(tn,Sn,In,IAn,IDn,IRn,ITn,Rn,Dn,Vn)]+αM(α){2312tn2−β(tn)(−β(tn+1)−β(tn)Δtlntn+2−β(tn)tn)×I∗(tn,Sn,In,IAn,IDn,IRn,ITn,Rn,Dn,Vn)Δt−43tn−12−β(tn−1)(−β(tn)−β(tn−1)Δtlntn−1+2−β(tn−1)tn−1)×I∗(tn−1,Sn−ΔtSn∗,In−ΔtIn∗,IAn−ΔtIAn∗,IDn−ΔtIDn∗,IRn−ΔtIRn∗,ITn−ΔtITn∗,Rn−ΔtRn∗,Dn−ΔtDn∗,Vn−ΔtVn∗)Δt+512tn−22−β(tn−2)(−β(tn−1)−β(tn−2)Δtlntn−2+2−β(tn−2)tn−2)×I∗(tn−2,Sn−ΔtSn∗−ΔtS(n−1)∗,In−ΔtIn∗−ΔtI(n−1)∗,IAn−ΔtIAn∗−ΔtIA(n−1)∗,IDn−ΔtIDn∗−ΔtID(n−1)∗,IRn−ΔtIRn∗−ΔtIR(n−1)∗,ITn−ΔtITn∗−ΔtIT(n−1)∗,Rn−ΔtRn∗−ΔtR(n−1)∗,Dn−ΔtDn∗−ΔtD(n−1)∗,Vn−ΔtVn∗−ΔtV(n−1)∗)Δt},IAn+1=IAn+1−αM(α)[tn+12−β(tn+1)(−β(tn+2)−β(tn+1)Δtlntn+1+2−β(tn+1)tn+1)×IA∗(tn+1,Sn+ΔtSn∗,In+ΔtIn∗,IAn+ΔtIAn∗,IDn+ΔtIDn∗,IRn+ΔtIRn∗,ITn+ΔtITn∗,Rn+ΔtRn∗,Dn+ΔtDn∗,Vn+ΔtVn∗)−tn2−β(tn)(−β(tn+1)−β(tn)Δtlntn+2−β(tn)tn)×IA∗(tn,Sn,In,IAn,IDn,IRn,ITn,Rn,Dn,Vn)]IAn+1=+αM(α){2312tn2−β(tn)(−β(tn+1)−β(tn)Δtlntn+2−β(tn)tn)×IA∗(tn,Sn,In,IAn,IDn,IRn,ITn,Rn,Dn,Vn)Δt−43tn−12−β(tn−1)(−β(tn)−β(tn−1)Δtlntn−1+2−β(tn−1)tn−1)×IA∗(tn−1,Sn−ΔtSn∗,In−ΔtIn∗,IAn−ΔtIAn∗,IDn−ΔtIDn∗,IRn−ΔtIRn∗,ITn−ΔtITn∗,Rn−ΔtRn∗,Dn−ΔtDn∗,Vn−ΔtVn∗)Δt+512tn−22−β(tn−2)(−β(tn−1)−β(tn−2)Δtlntn−2+2−β(tn−2)tn−2)×IA∗(tn−2,Sn−ΔtSn∗−ΔtS(n−1)∗,In−ΔtIn∗−ΔtI(n−1)∗,IAn−ΔtIAn∗−ΔtIA(n−1)∗,IDn−ΔtIDn∗−ΔtID(n−1)∗,IRn−ΔtIRn∗−ΔtIR(n−1)∗,ITn−ΔtITn∗−ΔtIT(n−1)∗,Rn−ΔtRn∗−ΔtR(n−1)∗,Dn−ΔtDn∗−ΔtD(n−1)∗,Vn−ΔtVn∗−ΔtV(n−1)∗)Δt},IDn+1=IDn+1−αM(α)×[tn+12−β(tn+1)(−β(tn+2)−β(tn+1)Δtlntn+1+2−β(tn+1)tn+1)×ID∗(tn+1,Sn+ΔtSn∗,In+ΔtIn∗,IAn+ΔtIAn∗,IDn+ΔtIDn∗,IRn+ΔtIRn∗,ITn+ΔtITn∗,Rn+ΔtRn∗,Dn+ΔtDn∗,Vn+ΔtVn∗)−tn2−β(tn)(−β(tn+1)−β(tn)Δtlntn+2−β(tn)tn)×ID∗(tn,Sn,In,IAn,IDn,IRn,ITn,Rn,Dn,Vn)]+αM(α){2312tn2−β(tn)(−β(tn+1)−β(tn)Δtlntn+2−β(tn)tn)×ID∗(tn,Sn,In,IAn,IDn,IRn,ITn,Rn,Dn,Vn)Δt−43tn−12−β(tn−1)(−β(tn)−β(tn−1)Δtlntn−1+2−β(tn−1)tn−1)×ID∗(tn−1,Sn−ΔtSn∗,In−ΔtIn∗,IAn−ΔtIAn∗,IDn−ΔtIDn∗,IRn−ΔtIRn∗,ITn−ΔtITn∗,Rn−ΔtRn∗,Dn−ΔtDn∗,Vn−ΔtVn∗)Δt+512tn−22−β(tn−2)(−β(tn−1)−β(tn−2)Δtlntn−2+2−β(tn−2)tn−2)×ID∗(tn−2,Sn−ΔtSn∗−ΔtS(n−1)∗,In−ΔtIn∗−ΔtI(n−1)∗,IAn−ΔtIAn∗−ΔtIA(n−1)∗,IDn−ΔtIDn∗−ΔtID(n−1)∗,IRn−ΔtIRn∗−ΔtIR(n−1)∗,ITn−ΔtITn∗−ΔtIT(n−1)∗,Rn−ΔtRn∗−ΔtR(n−1)∗,Dn−ΔtDn∗−ΔtD(n−1)∗,Vn−ΔtVn∗−ΔtV(n−1)∗)Δt},IRn+1=IRn+1−αM(α)[tn+12−β(tn+1)(−β(tn+2)−β(tn+1)Δtlntn+1+2−β(tn+1)tn+1)×IR∗(tn+1,Sn+ΔtSn∗,In+ΔtIn∗,IAn+ΔtIAn∗,IDn+ΔtIDn∗,IRn+ΔtIRn∗,ITn+ΔtITn∗,Rn+ΔtRn∗,Dn+ΔtDn∗,Vn+ΔtVn∗)−tn2−β(tn)(−β(tn+1)−β(tn)Δtlntn+2−β(tn)tn)×IR∗(tn,Sn,In,IAn,IDn,IRn,ITn,Rn,Dn,Vn)]IRn+1=+αM(α){2312tn2−β(tn)(−β(tn+1)−β(tn)Δtlntn+2−β(tn)tn)×ID∗(tn,Sn,In,IAn,IDn,IRn,ITn,Rn,Dn,Vn)Δt−43tn−12−β(tn−1)(−β(tn)−β(tn−1)Δtlntn−1+2−β(tn−1)tn−1)×IR∗(tn−1,Sn−ΔtSn∗,In−ΔtIn∗,IAn−ΔtIAn∗,IDn−ΔtIDn∗,IRn−ΔtIRn∗,ITn−ΔtITn∗,Rn−ΔtRn∗,Dn−ΔtDn∗,Vn−ΔtVn∗)Δt+512tn−22−β(tn−2)(−β(tn−1)−β(tn−2)Δtlntn−2+2−β(tn−2)tn−2)×IR∗(tn−2,Sn−ΔtSn∗−ΔtS(n−1)∗,In−ΔtIn∗−ΔtI(n−1)∗,IAn−ΔtIAn∗−ΔtIA(n−1)∗,IDn−ΔtIDn∗−ΔtID(n−1)∗,IRn−ΔtIRn∗−ΔtIR(n−1)∗,ITn−ΔtITn∗−ΔtIT(n−1)∗,Rn−ΔtRn∗−ΔtR(n−1)∗,Dn−ΔtDn∗−ΔtD(n−1)∗,Vn−ΔtVn∗−ΔtV(n−1)∗)Δt},ITn+1=ITn+1−αM(α)×[tn+12−β(tn+1)(−β(tn+2)−β(tn+1)Δtlntn+1+2−β(tn+1)tn+1)×IT∗(tn+1,Sn+ΔtSn∗,In+ΔtIn∗,IAn+ΔtIAn∗,IDn+ΔtIDn∗,IRn+ΔtIRn∗,ITn+ΔtITn∗,Rn+ΔtRn∗,Dn+ΔtDn∗,Vn+ΔtVn∗)−tn2−β(tn)(−β(tn+1)−β(tn)Δtlntn+2−β(tn)tn)×IT∗(tn,Sn,In,IAn,IDn,IRn,ITn,Rn,Dn,Vn)]+αM(α){2312tn2−β(tn)(−β(tn+1)−β(tn)Δtlntn+2−β(tn)tn)×IT∗(tn,Sn,In,IAn,IDn,IRn,ITn,Rn,Dn,Vn)Δt−43tn−12−β(tn−1)(−β(tn)−β(tn−1)Δtlntn−1+2−β(tn−1)tn−1)×IT∗(tn−1,Sn−ΔtSn∗,In−ΔtIn∗,IAn−ΔtIAn∗,IDn−ΔtIDn∗,IRn−ΔtIRn∗,ITn−ΔtITn∗,Rn−ΔtRn∗,Dn−ΔtDn∗,Vn−ΔtVn∗)Δt+512tn−22−β(tn−2)(−β(tn−1)−β(tn−2)Δtlntn−2+2−β(tn−2)tn−2)×IT∗(tn−2,Sn−ΔtSn∗−ΔtS(n−1)∗,In−ΔtIn∗−ΔtI(n−1)∗,IAn−ΔtIAn∗−ΔtIA(n−1)∗,IDn−ΔtIDn∗−ΔtID(n−1)∗,IRn−ΔtIRn∗−ΔtIR(n−1)∗,ITn−ΔtITn∗−ΔtIT(n−1)∗,Rn−ΔtRn∗−ΔtR(n−1)∗,Dn−ΔtDn∗−ΔtD(n−1)∗,Vn−ΔtVn∗−ΔtV(n−1)∗)Δt},Rn+1=Rn+1−αM(α)Rn+1=×[tn+12−β(tn+1)(−β(tn+2)−β(tn+1)Δtlntn+1+2−β(tn+1)tn+1)×R∗(tn+1,Sn+ΔtSn∗,In+ΔtIn∗,IAn+ΔtIAn∗,IDn+ΔtIDn∗,IRn+ΔtIRn∗,ITn+ΔtITn∗,Rn+ΔtRn∗,Dn+ΔtDn∗,Vn+ΔtVn∗)−tn2−β(tn)(−β(tn+1)−β(tn)Δtlntn+2−β(tn)tn)R∗(tn,Sn,In,IAn,IDn,IRn,ITn,Rn,Dn,Vn)]Rn+1=+αM(α){2312tn2−β(tn)(−β(tn+1)−β(tn)Δtlntn+2−β(tn)tn)×R∗(tn,Sn,In,IAn,IDn,IRn,ITn,Rn,Dn,Vn)Δt−43tn−12−β(tn−1)(−β(tn)−β(tn−1)Δtlntn−1+2−β(tn−1)tn−1)×R∗(tn−1,Sn−ΔtSn∗,In−ΔtIn∗,IAn−ΔtIAn∗,IDn−ΔtIDn∗,IRn−ΔtIRn∗,ITn−ΔtITn∗,Rn−ΔtRn∗,Dn−ΔtDn∗,Vn−ΔtVn∗)Δt+512tn−22−β(tn−2)(−β(tn−1)−β(tn−2)Δtlntn−2+2−β(tn−2)tn−2)×R∗(tn−2,Sn−ΔtSn∗−ΔtS(n−1)∗,In−ΔtIn∗−ΔtI(n−1)∗,IAn−ΔtIAn∗−ΔtIA(n−1)∗,IDn−ΔtIDn∗−ΔtID(n−1)∗,IRn−ΔtIRn∗−ΔtIR(n−1)∗,ITn−ΔtITn∗−ΔtIT(n−1)∗,Rn−ΔtRn∗−ΔtR(n−1)∗,Dn−ΔtDn∗−ΔtD(n−1)∗,Vn−ΔtVn∗−ΔtV(n−1)∗)Δt},Dn+1=Dn+1−αM(α)×[tn+12−β(tn+1)(−β(tn+2)−β(tn+1)Δtlntn+1+2−β(tn+1)tn+1)×D∗(tn+1,Sn+ΔtSn∗,In+ΔtIn∗,IAn+ΔtIAn∗,IDn+ΔtIDn∗,IRn+ΔtIRn∗,ITn+ΔtITn∗,Rn+ΔtRn∗,Dn+ΔtDn∗,Vn+ΔtVn∗)−tn2−β(tn)(−β(tn+1)−β(tn)Δtlntn+2−β(tn)tn)×D∗(tn,Sn,In,IAn,IDn,IRn,ITn,Rn,Dn,Vn)]+αM(α){2312tn2−β(tn)(−β(tn+1)−β(tn)Δtlntn+2−β(tn)tn)×D∗(tn,Sn,In,IAn,IDn,IRn,ITn,Rn,Dn,Vn)Δt−43tn−12−β(tn−1)(−β(tn)−β(tn−1)Δtlntn−1+2−β(tn−1)tn−1)×D∗(tn−1,Sn−ΔtSn∗,In−ΔtIn∗,IAn−ΔtIAn∗,IDn−ΔtIDn∗,IRn−ΔtIRn∗,ITn−ΔtITn∗,Rn−ΔtRn∗,Dn−ΔtDn∗,Vn−ΔtVn∗)Δt+512tn−22−β(tn−2)(−β(tn−1)−β(tn−2)Δtlntn−2+2−β(tn−2)tn−2)×D∗(tn−2,Sn−ΔtSn∗−ΔtS(n−1)∗,In−ΔtIn∗−ΔtI(n−1)∗,IAn−ΔtIAn∗−ΔtIA(n−1)∗,IDn−ΔtIDn∗−ΔtID(n−1)∗,IRn−ΔtIRn∗−ΔtIR(n−1)∗,ITn−ΔtITn∗−ΔtIT(n−1)∗,Rn−ΔtRn∗−ΔtR(n−1)∗,Dn−ΔtDn∗−ΔtD(n−1)∗,Vn−ΔtVn∗−ΔtV(n−1)∗)Δt},Vn+1=Vn+1−αM(α)Vn+1=×[tn+12−β(tn+1)(−β(tn+2)−β(tn+1)Δtlntn+1+2−β(tn+1)tn+1)×V∗(tn+1,Sn+ΔtSn∗,In+ΔtIn∗,IAn+ΔtIAn∗,IDn+ΔtIDn∗,IRn+ΔtIRn∗,ITn+ΔtITn∗,Rn+ΔtRn∗,Dn+ΔtDn∗,Vn+ΔtVn∗)−tn2−β(tn)(−β(tn+1)−β(tn)Δtlntn+2−β(tn)tn)×V∗(tn,Sn,In,IAn,IDn,IRn,ITn,Rn,Dn,Vn)]Vn+1=+αM(α){2312tn2−β(tn)(−β(tn+1)−β(tn)Δtlntn+2−β(tn)tn)×R∗(tn,Sn,In,IAn,IDn,IRn,ITn,Rn,Dn,Vn)Δt−43tn−12−β(tn−1)(−β(tn)−β(tn−1)Δtlntn−1+2−β(tn−1)tn−1)×V∗(tn−1,Sn−ΔtSn∗,In−ΔtIn∗,IAn−ΔtIAn∗,IDn−ΔtIDn∗,IRn−ΔtIRn∗,ITn−ΔtITn∗,Rn−ΔtRn∗,Dn−ΔtDn∗,Vn−ΔtVn∗)Δt+512tn−22−β(tn−2)(−β(tn−1)−β(tn−2)Δtlntn−2+2−β(tn−2)tn−2)×V∗(tn−2,Sn−ΔtSn∗−ΔtS(n−1)∗,In−ΔtIn∗−ΔtI(n−1)∗,IAn−ΔtIAn∗−ΔtIA(n−1)∗,IDn−ΔtIDn∗−ΔtID(n−1)∗,IRn−ΔtIRn∗−ΔtIR(n−1)∗,ITn−ΔtITn∗−ΔtIT(n−1)∗,Rn−ΔtRn∗−ΔtR(n−1)∗,Dn−ΔtDn∗−ΔtD(n−1)∗,Vn−ΔtVn∗−ΔtV(n−1)∗)Δt}.

For the Atangana–Baleanu fractal-fractional derivative, we can have the following numerical scheme:

139 Sn+1=1−αAB(α)tn+12−β(tn+1)(−β(tn+2)−β(tn+1)Δtlntn+1+2−β(tn+1)tn+1)Sn+1=×S∗(tn+1,Sn+ΔtSn∗,In+ΔtIn∗,IAn+ΔtIAn∗,IDn+ΔtIDn∗,IRn+ΔtIRn∗,ITn+ΔtITn∗,Rn+ΔtRn∗,Dn+ΔtDn∗,Vn+ΔtVn∗)Sn+1=+α(Δt)αAB(α)Γ(α+1)∑j=2ntj−22−β(tj−2)(−β(tj−1)−β(tj−2)Δtlntj−2+2−β(tj−2)tj−2)Sn+1=×S∗(tj−2,Sj−ΔtSj∗−ΔtS(j−1)∗,Ij−ΔtIj∗−ΔtI(j−1)∗,IAj−ΔtIAj∗−ΔtIA(j−1)∗,IDj−ΔtIDj∗−ΔtID(j−1)∗,IRj−ΔtIRj∗−ΔtIR(j−1)∗,ITj−ΔtITj∗−ΔtIT(j−1)∗,Rj−ΔtRj∗−ΔtR(j−1)∗,Dj−ΔtDj∗−ΔtD(j−1)∗,Vj−ΔtVj∗−ΔtV(j−1)∗)×ΠSn+1=+α(Δt)αAB(α)Γ(α+2)Sn+1=×∑j=2n[tj−12−β(tj−1)(−β(tj)−β(tj−1)Δtlntj−1+2−β(tj−1)tj−1)×S∗(tj−1,Sj−ΔtSj∗,Ij−ΔtIj∗,IAj−ΔtIAj∗,IDj−ΔtIDj∗,IRj−ΔtIRj∗,ITj−ΔtITj∗,Rj−ΔtRj∗,Dj−ΔtDj∗,Vj−ΔtVj∗)−tj−22−β(tj−2)(−β(tj−1)−β(tj−2)Δtlntj−2+2−β(tj−2)tj−2)×S∗(tj−2,Sj−ΔtSj∗−ΔtS(j−1)∗,Ij−ΔtIj∗−ΔtI(j−1)∗,IAj−ΔtIAj∗−ΔtIA(j−1)∗,IDj−ΔtIDj∗−ΔtID(j−1)∗,IRj−ΔtIRj∗−ΔtIR(j−1)∗,ITj−ΔtITj∗−ΔtIT(j−1)∗,Rj−ΔtRj∗−ΔtR(j−1)∗,Dj−ΔtDj∗−ΔtD(j−1)∗,Vj−ΔtVj∗−ΔtV(j−1)∗)]×ΣSn+1=+α(Δt)α2AB(α)Γ(α+3)Sn+1=×∑j=2n[tj2−β(tj)(−β(tj+1)−β(tj)Δtlntj+2−β(tj)tj)×S∗(tj,Sj,Ij,IAj,IDj,IRj,ITj,Rj,Dj,Vj)−2tj−12−β(tj−1)(−β(tj)−β(tj−1)Δtlntj−1+2−β(tj−1)tj−1)×S∗(tj−1,Sj−ΔtSj∗,Ij−ΔtIj∗,IAj−ΔtIAj∗,IDj−ΔtIDj∗,IRj−ΔtIRj∗,ITj−ΔtITj∗,Rj−ΔtRj∗,Dj−ΔtDj∗,Vj−ΔtVj∗)+tj−22−β(tj−2)(−β(tj−1)−β(tj−2)Δtlntj−2+2−β(tj−2)tj−2)×S∗(tj−2,Sj−ΔtSj∗−ΔtS(j−1)∗,Ij−ΔtIj∗−ΔtI(j−1)∗,IAj−ΔtIAj∗−ΔtIA(j−1)∗,IDj−ΔtIDj∗−ΔtID(j−1)∗,IRj−ΔtIRj∗−ΔtIR(j−1)∗,ITj−ΔtITj∗−ΔtIT(j−1)∗,Rj−ΔtRj∗−ΔtR(j−1)∗,Dj−ΔtDj∗−ΔtD(j−1)∗,Vj−ΔtVj∗−ΔtV(j−1)∗)]×Δ,In+1=1−αAB(α)tn+12−β(tn+1)(−β(tn+2)−β(tn+1)Δtlntn+1+2−β(tn+1)tn+1)In+1=×I∗(tn+1,Sn+ΔtSn∗,In+ΔtIn∗,IAn+ΔtIAn∗,IDn+ΔtIDn∗,IRn+ΔtIRn∗,ITn+ΔtITn∗,Rn+ΔtRn∗,Dn+ΔtDn∗,Vn+ΔtVn∗)In+1=+α(Δt)αAB(α)Γ(α+1)∑j=2ntj−22−β(tj−2)(−β(tj−1)−β(tj−2)Δtlntj−2+2−β(tj−2)tj−2)In+1=×I∗(tj−2,Sj−ΔtSj∗−ΔtS(j−1)∗,Ij−ΔtIj∗−ΔtI(j−1)∗,IAj−ΔtIAj∗−ΔtIA(j−1)∗,IDj−ΔtIDj∗−ΔtID(j−1)∗,IRj−ΔtIRj∗−ΔtIR(j−1)∗,ITj−ΔtITj∗−ΔtIT(j−1)∗,Rj−ΔtRj∗−ΔtR(j−1)∗,Dj−ΔtDj∗−ΔtD(j−1)∗,Vj−ΔtVj∗−ΔtV(j−1)∗)×ΠIn+1=+α(Δt)αAB(α)Γ(α+2)In+1=×∑j=2n[tj−12−β(tj−1)(−β(tj)−β(tj−1)Δtlntj−1+2−β(tj−1)tj−1)×I∗(tj−1,Sj−ΔtSj∗,Ij−ΔtIj∗,IAj−ΔtIAj∗,IDj−ΔtIDj∗,IRj−ΔtIRj∗,ITj−ΔtITj∗,Rj−ΔtRj∗,Dj−ΔtDj∗,Vj−ΔtVj∗)−tj−22−β(tj−2)(−β(tj−1)−β(tj−2)Δtlntj−2+2−β(tj−2)tj−2)×I∗(tj−2,Sj−ΔtSj∗−ΔtS(j−1)∗,Ij−ΔtIj∗−ΔtI(j−1)∗,IAj−ΔtIAj∗−ΔtIA(j−1)∗,IDj−ΔtIDj∗−ΔtID(j−1)∗,IRj−ΔtIRj∗−ΔtIR(j−1)∗,ITj−ΔtITj∗−ΔtIT(j−1)∗,Rj−ΔtRj∗−ΔtR(j−1)∗,Dj−ΔtDj∗−ΔtD(j−1)∗,Vj−ΔtVj∗−ΔtV(j−1)∗)]×ΣIn+1=+α(Δt)α2AB(α)Γ(α+3)In+1=×∑j=2n[tj2−β(tj)(−β(tj+1)−β(tj)Δtlntj+2−β(tj)tj)×I∗(tj,Sj,Ij,IAj,IDj,IRj,ITj,Rj,Dj,Vj)−2tj−12−β(tj−1)(−β(tj)−β(tj−1)Δtlntj−1+2−β(tj−1)tj−1)×I∗(tj−1,Sj−ΔtSj∗,Ij−ΔtIj∗,IAj−ΔtIAj∗,IDj−ΔtIDj∗,IRj−ΔtIRj∗,ITj−ΔtITj∗,Rj−ΔtRj∗,Dj−ΔtDj∗,Vj−ΔtVj∗)+tj−22−β(tj−2)(−β(tj−1)−β(tj−2)Δtlntj−2+2−β(tj−2)tj−2)×I∗(tj−2,Sj−ΔtSj∗−ΔtS(j−1)∗,Ij−ΔtIj∗−ΔtI(j−1)∗,IAj−ΔtIAj∗−ΔtIA(j−1)∗,IDj−ΔtIDj∗−ΔtID(j−1)∗,IRj−ΔtIRj∗−ΔtIR(j−1)∗,ITj−ΔtITj∗−ΔtIT(j−1)∗,Rj−ΔtRj∗−ΔtR(j−1)∗,Dj−ΔtDj∗−ΔtD(j−1)∗,Vj−ΔtVj∗−ΔtV(j−1)∗)]×Δ,IAn+1=1−αAB(α)tn+12−β(tn+1)(−β(tn+2)−β(tn+1)Δtlntn+1+2−β(tn+1)tn+1)IAn+1=×IA∗(tn+1,Sn+ΔtSn∗,In+ΔtIn∗,IAn+ΔtIAn∗,IDn+ΔtIDn∗,IRn+ΔtIRn∗,ITn+ΔtITn∗,Rn+ΔtRn∗,Dn+ΔtDn∗,Vn+ΔtVn∗)IAn+1=+α(Δt)αAB(α)Γ(α+1)∑j=2ntj−22−β(tj−2)(−β(tj−1)−β(tj−2)Δtlntj−2+2−β(tj−2)tj−2)IAn+1=×IA∗(tj−2,Sj−ΔtSj∗−ΔtS(j−1)∗,Ij−ΔtIj∗−ΔtI(j−1)∗,IAj−ΔtIAj∗−ΔtIA(j−1)∗,IDj−ΔtIDj∗−ΔtID(j−1)∗,IRj−ΔtIRj∗−ΔtIR(j−1)∗,ITj−ΔtITj∗−ΔtIT(j−1)∗,Rj−ΔtRj∗−ΔtR(j−1)∗,Dj−ΔtDj∗−ΔtD(j−1)∗,Vj−ΔtVj∗−ΔtV(j−1)∗)×ΠIAn+1=+α(Δt)αAB(α)Γ(α+2)IAn+1=×∑j=2n[tj−12−β(tj−1)(−β(tj)−β(tj−1)Δtlntj−1+2−β(tj−1)tj−1)×IA∗(tj−1,Sj−ΔtSj∗,Ij−ΔtIj∗,IAj−ΔtIAj∗,IDj−ΔtIDj∗,IRj−ΔtIRj∗,ITj−ΔtITj∗,Rj−ΔtRj∗,Dj−ΔtDj∗,Vj−ΔtVj∗)−tj−22−β(tj−2)(−β(tj−1)−β(tj−2)Δtlntj−2+2−β(tj−2)tj−2)×IA∗(tj−2,Sj−ΔtSj∗−ΔtS(j−1)∗,Ij−ΔtIj∗−ΔtI(j−1)∗,IAj−ΔtIAj∗−ΔtIA(j−1)∗,IDj−ΔtIDj∗−ΔtID(j−1)∗,IRj−ΔtIRj∗−ΔtIR(j−1)∗,ITj−ΔtITj∗−ΔtIT(j−1)∗,Rj−ΔtRj∗−ΔtR(j−1)∗,Dj−ΔtDj∗−ΔtD(j−1)∗,Vj−ΔtVj∗−ΔtV(j−1)∗)]×ΣIAn+1=+α(Δt)α2AB(α)Γ(α+3)IAn+1=×∑j=2n[tj2−β(tj)(−β(tj+1)−β(tj)Δtlntj+2−β(tj)tj)×IA∗(tj,Sj,Ij,IAj,IDj,IRj,ITj,Rj,Dj,Vj)−2tj−12−β(tj−1)(−β(tj)−β(tj−1)Δtlntj−1+2−β(tj−1)tj−1)×IA∗(tj−1,Sj−ΔtSj∗,Ij−ΔtIj∗,IAj−ΔtIAj∗,IDj−ΔtIDj∗,IRj−ΔtIRj∗,ITj−ΔtITj∗,Rj−ΔtRj∗,Dj−ΔtDj∗,Vj−ΔtVj∗)+tj−22−β(tj−2)(−β(tj−1)−β(tj−2)Δtlntj−2+2−β(tj−2)tj−2)×IA∗(tj−2,Sj−ΔtSj∗−ΔtS(j−1)∗,Ij−ΔtIj∗−ΔtI(j−1)∗,IAj−ΔtIAj∗−ΔtIA(j−1)∗,IDj−ΔtIDj∗−ΔtID(j−1)∗,IRj−ΔtIRj∗−ΔtIR(j−1)∗,ITj−ΔtITj∗−ΔtIT(j−1)∗,Rj−ΔtRj∗−ΔtR(j−1)∗,Dj−ΔtDj∗−ΔtD(j−1)∗,Vj−ΔtVj∗−ΔtV(j−1)∗)]×Δ,IDn+1=1−αAB(α)tn+12−β(tn+1)(−β(tn+2)−β(tn+1)Δtlntn+1+2−β(tn+1)tn+1)IDn+1=×ID∗(tn+1,Sn+ΔtSn∗,In+ΔtIn∗,IAn+ΔtIAn∗,IDn+ΔtIDn∗,IRn+ΔtIRn∗,ITn+ΔtITn∗,Rn+ΔtRn∗,Dn+ΔtDn∗,Vn+ΔtVn∗)IDn+1=+α(Δt)αAB(α)Γ(α+1)∑j=2ntj−22−β(tj−2)(−β(tj−1)−β(tj−2)Δtlntj−2+2−β(tj−2)tj−2)IDn+1=×ID∗(tj−2,Sj−ΔtSj∗−ΔtS(j−1)∗,Ij−ΔtIj∗−ΔtI(j−1)∗,IAj−ΔtIAj∗−ΔtIA(j−1)∗,IDj−ΔtIDj∗−ΔtID(j−1)∗,IRj−ΔtIRj∗−ΔtIR(j−1)∗,ITj−ΔtITj∗−ΔtIT(j−1)∗,Rj−ΔtRj∗−ΔtR(j−1)∗,Dj−ΔtDj∗−ΔtD(j−1)∗,Vj−ΔtVj∗−ΔtV(j−1)∗)×ΠIDn+1=+α(Δt)αAB(α)Γ(α+2)IDn+1=×∑j=2n[tj−12−β(tj−1)(−β(tj)−β(tj−1)Δtlntj−1+2−β(tj−1)tj−1)×ID∗(tj−1,Sj−ΔtSj∗,Ij−ΔtIj∗,IAj−ΔtIAj∗,IDj−ΔtIDj∗,IRj−ΔtIRj∗,ITj−ΔtITj∗,Rj−ΔtRj∗,Dj−ΔtDj∗,Vj−ΔtVj∗)−tj−22−β(tj−2)(−β(tj−1)−β(tj−2)Δtlntj−2+2−β(tj−2)tj−2)×ID∗(tj−2,Sj−ΔtSj∗−ΔtS(j−1)∗,Ij−ΔtIj∗−ΔtI(j−1)∗,IAj−ΔtIAj∗−ΔtIA(j−1)∗,IDj−ΔtIDj∗−ΔtID(j−1)∗,IRj−ΔtIRj∗−ΔtIR(j−1)∗,ITj−ΔtITj∗−ΔtIT(j−1)∗,Rj−ΔtRj∗−ΔtR(j−1)∗,Dj−ΔtDj∗−ΔtD(j−1)∗,Vj−ΔtVj∗−ΔtV(j−1)∗)]×ΣIDn+1=+α(Δt)α2AB(α)Γ(α+3)IDn+1=×∑j=2n[tj2−β(tj)(−β(tj+1)−β(tj)Δtlntj+2−β(tj)tj)×ID∗(tj,Sj,Ij,IAj,IDj,IRj,ITj,Rj,Dj,Vj)−2tj−12−β(tj−1)(−β(tj)−β(tj−1)Δtlntj−1+2−β(tj−1)tj−1)×ID∗(tj−1,Sj−ΔtSj∗,Ij−ΔtIj∗,IAj−ΔtIAj∗,IDj−ΔtIDj∗,IRj−ΔtIRj∗,ITj−ΔtITj∗,Rj−ΔtRj∗,Dj−ΔtDj∗,Vj−ΔtVj∗)+tj−22−β(tj−2)(−β(tj−1)−β(tj−2)Δtlntj−2+2−β(tj−2)tj−2)×ID∗(tj−2,Sj−ΔtSj∗−ΔtS(j−1)∗,Ij−ΔtIj∗−ΔtI(j−1)∗,IAj−ΔtIAj∗−ΔtIA(j−1)∗,IDj−ΔtIDj∗−ΔtID(j−1)∗,IRj−ΔtIRj∗−ΔtIR(j−1)∗,ITj−ΔtITj∗−ΔtIT(j−1)∗,Rj−ΔtRj∗−ΔtR(j−1)∗,Dj−ΔtDj∗−ΔtD(j−1)∗,Vj−ΔtVj∗−ΔtV(j−1)∗)]×Δ,IRn+1=1−αAB(α)tn+12−β(tn+1)(−β(tn+2)−β(tn+1)Δtlntn+1+2−β(tn+1)tn+1)IRn+1=×IR∗(tn+1,Sn+ΔtSn∗,In+ΔtIn∗,IAn+ΔtIAn∗,IDn+ΔtIDn∗,IRn+ΔtIRn∗,ITn+ΔtITn∗,Rn+ΔtRn∗,Dn+ΔtDn∗,Vn+ΔtVn∗)IRn+1=+α(Δt)αAB(α)Γ(α+1)∑j=2ntj−22−β(tj−2)(−β(tj−1)−β(tj−2)Δtlntj−2+2−β(tj−2)tj−2)IRn+1=×IR∗(tj−2,Sj−ΔtSj∗−ΔtS(j−1)∗,Ij−ΔtIj∗−ΔtI(j−1)∗,IAj−ΔtIAj∗−ΔtIA(j−1)∗,IDj−ΔtIDj∗−ΔtID(j−1)∗,IRj−ΔtIRj∗−ΔtIR(j−1)∗,ITj−ΔtITj∗−ΔtIT(j−1)∗,Rj−ΔtRj∗−ΔtR(j−1)∗,Dj−ΔtDj∗−ΔtD(j−1)∗,Vj−ΔtVj∗−ΔtV(j−1)∗)×ΠIRn+1=+α(Δt)αAB(α)Γ(α+2)IRn+1=×∑j=2n[tj−12−β(tj−1)(−β(tj)−β(tj−1)Δtlntj−1+2−β(tj−1)tj−1)×IR∗(tj−1,Sj−ΔtSj∗,Ij−ΔtIj∗,IAj−ΔtIAj∗,IDj−ΔtIDj∗,IRj−ΔtIRj∗,ITj−ΔtITj∗,Rj−ΔtRj∗,Dj−ΔtDj∗,Vj−ΔtVj∗)−tj−22−β(tj−2)(−β(tj−1)−β(tj−2)Δtlntj−2+2−β(tj−2)tj−2)×IR∗(tj−2,Sj−ΔtSj∗−ΔtS(j−1)∗,Ij−ΔtIj∗−ΔtI(j−1)∗,IAj−ΔtIAj∗−ΔtIA(j−1)∗,IDj−ΔtIDj∗−ΔtID(j−1)∗,IRj−ΔtIRj∗−ΔtIR(j−1)∗,ITj−ΔtITj∗−ΔtIT(j−1)∗,Rj−ΔtRj∗−ΔtR(j−1)∗,Dj−ΔtDj∗−ΔtD(j−1)∗,Vj−ΔtVj∗−ΔtV(j−1)∗)]×ΣIRn+1=+α(Δt)α2AB(α)Γ(α+3)IRn+1=×∑j=2n[tj2−β(tj)(−β(tj+1)−β(tj)Δtlntj+2−β(tj)tj)×IR∗(tj,Sj,Ij,IAj,IDj,IRj,ITj,Rj,Dj,Vj)−2tj−12−β(tj−1)(−β(tj)−β(tj−1)Δtlntj−1+2−β(tj−1)tj−1)×IR∗(tj−1,Sj−ΔtSj∗,Ij−ΔtIj∗,IAj−ΔtIAj∗,IDj−ΔtIDj∗,IRj−ΔtIRj∗,ITj−ΔtITj∗,Rj−ΔtRj∗,Dj−ΔtDj∗,Vj−ΔtVj∗)+tj−22−β(tj−2)(−β(tj−1)−β(tj−2)Δtlntj−2+2−β(tj−2)tj−2)×IR∗(tj−2,Sj−ΔtSj∗−ΔtS(j−1)∗,Ij−ΔtIj∗−ΔtI(j−1)∗,IAj−ΔtIAj∗−ΔtIA(j−1)∗,IDj−ΔtIDj∗−ΔtID(j−1)∗,IRj−ΔtIRj∗−ΔtIR(j−1)∗,ITj−ΔtITj∗−ΔtIT(j−1)∗,Rj−ΔtRj∗−ΔtR(j−1)∗,Dj−ΔtDj∗−ΔtD(j−1)∗,Vj−ΔtVj∗−ΔtV(j−1)∗)]×Δ,ITn+1=1−αAB(α)tn+12−β(tn+1)(−β(tn+2)−β(tn+1)Δtlntn+1+2−β(tn+1)tn+1)ITn+1=×IT∗(tn+1,Sn+ΔtSn∗,In+ΔtIn∗,IAn+ΔtIAn∗,IDn+ΔtIDn∗,IRn+ΔtIRn∗,ITn+ΔtITn∗,Rn+ΔtRn∗,Dn+ΔtDn∗,Vn+ΔtVn∗)ITn+1=+α(Δt)αAB(α)Γ(α+1)∑j=2ntj−22−β(tj−2)(−β(tj−1)−β(tj−2)Δtlntj−2+2−β(tj−2)tj−2)ITn+1=×IT∗(tj−2,Sj−ΔtSj∗−ΔtS(j−1)∗,Ij−ΔtIj∗−ΔtI(j−1)∗,IAj−ΔtIAj∗−ΔtIA(j−1)∗,IDj−ΔtIDj∗−ΔtID(j−1)∗,IRj−ΔtIRj∗−ΔtIR(j−1)∗,ITj−ΔtITj∗−ΔtIT(j−1)∗,Rj−ΔtRj∗−ΔtR(j−1)∗,Dj−ΔtDj∗−ΔtD(j−1)∗,Vj−ΔtVj∗−ΔtV(j−1)∗)×ΠITn+1=+α(Δt)αAB(α)Γ(α+2)ITn+1=×∑j=2n[tj−12−β(tj−1)(−β(tj)−β(tj−1)Δtlntj−1+2−β(tj−1)tj−1)×IT∗(tj−1,Sj−ΔtSj∗,Ij−ΔtIj∗,IAj−ΔtIAj∗,IDj−ΔtIDj∗,IRj−ΔtIRj∗,ITj−ΔtITj∗,Rj−ΔtRj∗,Dj−ΔtDj∗,Vj−ΔtVj∗)−tj−22−β(tj−2)(−β(tj−1)−β(tj−2)Δtlntj−2+2−β(tj−2)tj−2)×IT∗(tj−2,Sj−ΔtSj∗−ΔtS(j−1)∗,Ij−ΔtIj∗−ΔtI(j−1)∗,IAj−ΔtIAj∗−ΔtIA(j−1)∗,IDj−ΔtIDj∗−ΔtID(j−1)∗,IRj−ΔtIRj∗−ΔtIR(j−1)∗,ITj−ΔtITj∗−ΔtIT(j−1)∗,Rj−ΔtRj∗−ΔtR(j−1)∗,Dj−ΔtDj∗−ΔtD(j−1)∗,Vj−ΔtVj∗−ΔtV(j−1)∗)]×ΣITn+1=+α(Δt)α2AB(α)Γ(α+3)ITn+1=×∑j=2n[tj2−β(tj)(−β(tj+1)−β(tj)Δtlntj+2−β(tj)tj)×IT∗(tj,Sj,Ij,IAj,IDj,IRj,ITj,Rj,Dj,Vj)−2tj−12−β(tj−1)(−β(tj)−β(tj−1)Δtlntj−1+2−β(tj−1)tj−1)×IT∗(tj−1,Sj−ΔtSj∗,Ij−ΔtIj∗,IAj−ΔtIAj∗,IDj−ΔtIDj∗,IRj−ΔtIRj∗,ITj−ΔtITj∗,Rj−ΔtRj∗,Dj−ΔtDj∗,Vj−ΔtVj∗)+tj−22−β(tj−2)(−β(tj−1)−β(tj−2)Δtlntj−2+2−β(tj−2)tj−2)×IT∗(tj−2,Sj−ΔtSj∗−ΔtS(j−1)∗,Ij−ΔtIj∗−ΔtI(j−1)∗,IAj−ΔtIAj∗−ΔtIA(j−1)∗,IDj−ΔtIDj∗−ΔtID(j−1)∗,IRj−ΔtIRj∗−ΔtIR(j−1)∗,ITj−ΔtITj∗−ΔtIT(j−1)∗,Rj−ΔtRj∗−ΔtR(j−1)∗,Dj−ΔtDj∗−ΔtD(j−1)∗,Vj−ΔtVj∗−ΔtV(j−1)∗)]×Δ,Rn+1=1−αAB(α)tn+12−β(tn+1)(−β(tn+2)−β(tn+1)Δtlntn+1+2−β(tn+1)tn+1)Rn+1=×R∗(tn+1,Sn+ΔtSn∗,In+ΔtIn∗,IAn+ΔtIAn∗,IDn+ΔtIDn∗,IRn+ΔtIRn∗,ITn+ΔtITn∗,Rn+ΔtRn∗,Dn+ΔtDn∗,Vn+ΔtVn∗)Rn+1=+α(Δt)αAB(α)Γ(α+1)∑j=2ntj−22−β(tj−2)(−β(tj−1)−β(tj−2)Δtlntj−2+2−β(tj−2)tj−2)Rn+1=×R∗(tj−2,Sj−ΔtSj∗−ΔtS(j−1)∗,Ij−ΔtIj∗−ΔtI(j−1)∗,IAj−ΔtIAj∗−ΔtIA(j−1)∗,IDj−ΔtIDj∗−ΔtID(j−1)∗,IRj−ΔtIRj∗−ΔtIR(j−1)∗,ITj−ΔtITj∗−ΔtIT(j−1)∗,Rj−ΔtRj∗−ΔtR(j−1)∗,Dj−ΔtDj∗−ΔtD(j−1)∗,Vj−ΔtVj∗−ΔtV(j−1)∗)×ΠRn+1=+α(Δt)αAB(α)Γ(α+2)Rn+1=×∑j=2n[tj−12−β(tj−1)(−β(tj)−β(tj−1)Δtlntj−1+2−β(tj−1)tj−1)×R∗(tj−1,Sj−ΔtSj∗,Ij−ΔtIj∗,IAj−ΔtIAj∗,IDj−ΔtIDj∗,IRj−ΔtIRj∗,ITj−ΔtITj∗,Rj−ΔtRj∗,Dj−ΔtDj∗,Vj−ΔtVj∗)−tj−22−β(tj−2)(−β(tj−1)−β(tj−2)Δtlntj−2+2−β(tj−2)tj−2)×R∗(tj−2,Sj−ΔtSj∗−ΔtS(j−1)∗,Ij−ΔtIj∗−ΔtI(j−1)∗,IAj−ΔtIAj∗−ΔtIA(j−1)∗,IDj−ΔtIDj∗−ΔtID(j−1)∗,IRj−ΔtIRj∗−ΔtIR(j−1)∗,ITj−ΔtITj∗−ΔtIT(j−1)∗,Rj−ΔtRj∗−ΔtR(j−1)∗,Dj−ΔtDj∗−ΔtD(j−1)∗,Vj−ΔtVj∗−ΔtV(j−1)∗)]×ΣRn+1=+α(Δt)α2AB(α)Γ(α+3)Rn+1=×∑j=2n[tj2−β(tj)(−β(tj+1)−β(tj)Δtlntj+2−β(tj)tj)×R∗(tj,Sj,Ij,IAj,IDj,IRj,ITj,Rj,Dj,Vj)−2tj−12−β(tj−1)(−β(tj)−β(tj−1)Δtlntj−1+2−β(tj−1)tj−1)×R∗(tj−1,Sj−ΔtSj∗,Ij−ΔtIj∗,IAj−ΔtIAj∗,IDj−ΔtIDj∗,IRj−ΔtIRj∗,ITj−ΔtITj∗,Rj−ΔtRj∗,Dj−ΔtDj∗,Vj−ΔtVj∗)+tj−22−β(tj−2)(−β(tj−1)−β(tj−2)Δtlntj−2+2−β(tj−2)tj−2)×R∗(tj−2,Sj−ΔtSj∗−ΔtS(j−1)∗,Ij−ΔtIj∗−ΔtI(j−1)∗,IAj−ΔtIAj∗−ΔtIA(j−1)∗,IDj−ΔtIDj∗−ΔtID(j−1)∗,IRj−ΔtIRj∗−ΔtIR(j−1)∗,ITj−ΔtITj∗−ΔtIT(j−1)∗,Rj−ΔtRj∗−ΔtR(j−1)∗,Dj−ΔtDj∗−ΔtD(j−1)∗,Vj−ΔtVj∗−ΔtV(j−1)∗)]×Δ,Dn+1=1−αAB(α)tn+12−β(tn+1)(−β(tn+2)−β(tn+1)Δtlntn+1+2−β(tn+1)tn+1)Dn+1=×D∗(tn+1,Sn+ΔtSn∗,In+ΔtIn∗,IAn+ΔtIAn∗,IDn+ΔtIDn∗,IRn+ΔtIRn∗,ITn+ΔtITn∗,Rn+ΔtRn∗,Dn+ΔtDn∗,Vn+ΔtVn∗)Dn+1=+α(Δt)αAB(α)Γ(α+1)∑j=2ntj−22−β(tj−2)(−β(tj−1)−β(tj−2)Δtlntj−2+2−β(tj−2)tj−2)Dn+1=×D∗(tj−2,Sj−ΔtSj∗−ΔtS(j−1)∗,Ij−ΔtIj∗−ΔtI(j−1)∗,IAj−ΔtIAj∗−ΔtIA(j−1)∗,IDj−ΔtIDj∗−ΔtID(j−1)∗,IRj−ΔtIRj∗−ΔtIR(j−1)∗,ITj−ΔtITj∗−ΔtIT(j−1)∗,Rj−ΔtRj∗−ΔtR(j−1)∗,Dj−ΔtDj∗−ΔtD(j−1)∗,Vj−ΔtVj∗−ΔtV(j−1)∗)×ΠDn+1=+α(Δt)αAB(α)Γ(α+2)Dn+1=×∑j=2n[tj−12−β(tj−1)(−β(tj)−β(tj−1)Δtlntj−1+2−β(tj−1)tj−1)×D∗(tj−1,Sj−ΔtSj∗,Ij−ΔtIj∗,IAj−ΔtIAj∗,IDj−ΔtIDj∗,IRj−ΔtIRj∗,ITj−ΔtITj∗,Rj−ΔtRj∗,Dj−ΔtDj∗,Vj−ΔtVj∗)−tj−22−β(tj−2)(−β(tj−1)−β(tj−2)Δtlntj−2+2−β(tj−2)tj−2)×D∗(tj−2,Sj−ΔtSj∗−ΔtS(j−1)∗,Ij−ΔtIj∗−ΔtI(j−1)∗,IAj−ΔtIAj∗−ΔtIA(j−1)∗,IDj−ΔtIDj∗−ΔtID(j−1)∗,IRj−ΔtIRj∗−ΔtIR(j−1)∗,ITj−ΔtITj∗−ΔtIT(j−1)∗,Rj−ΔtRj∗−ΔtR(j−1)∗,Dj−ΔtDj∗−ΔtD(j−1)∗,Vj−ΔtVj∗−ΔtV(j−1)∗)]×ΣDn+1=+α(Δt)α2AB(α)Γ(α+3)Dn+1=×∑j=2n[tj2−β(tj)(−β(tj+1)−β(tj)Δtlntj+2−β(tj)tj)×D∗(tj,Sj,Ij,IAj,IDj,IRj,ITj,Rj,Dj,Vj)−2tj−12−β(tj−1)(−β(tj)−β(tj−1)Δtlntj−1+2−β(tj−1)tj−1)×D∗(tj−1,Sj−ΔtSj∗,Ij−ΔtIj∗,IAj−ΔtIAj∗,IDj−ΔtIDj∗,IRj−ΔtIRj∗,ITj−ΔtITj∗,Rj−ΔtRj∗,Dj−ΔtDj∗,Vj−ΔtVj∗)+tj−22−β(tj−2)(−β(tj−1)−β(tj−2)Δtlntj−2+2−β(tj−2)tj−2)×D∗(tj−2,Sj−ΔtSj∗−ΔtS(j−1)∗,Ij−ΔtIj∗−ΔtI(j−1)∗,IAj−ΔtIAj∗−ΔtIA(j−1)∗,IDj−ΔtIDj∗−ΔtID(j−1)∗,IRj−ΔtIRj∗−ΔtIR(j−1)∗,ITj−ΔtITj∗−ΔtIT(j−1)∗,Rj−ΔtRj∗−ΔtR(j−1)∗,Dj−ΔtDj∗−ΔtD(j−1)∗,Vj−ΔtVj∗−ΔtV(j−1)∗)]×Δ,Vn+1=1−αAB(α)tn+12−β(tn+1)(−β(tn+2)−β(tn+1)Δtlntn+1+2−β(tn+1)tn+1)Vn+1=×V∗(tn+1,Sn+ΔtSn∗,In+ΔtIn∗,IAn+ΔtIAn∗,IDn+ΔtIDn∗,IRn+ΔtIRn∗,ITn+ΔtITn∗,Rn+ΔtRn∗,Dn+ΔtDn∗,Vn+ΔtVn∗)Vn+1=+α(Δt)αAB(α)Γ(α+1)∑j=2ntj−22−β(tj−2)(−β(tj−1)−β(tj−2)Δtlntj−2+2−β(tj−2)tj−2)Vn+1=×V∗(tj−2,Sj−ΔtSj∗−ΔtS(j−1)∗,Ij−ΔtIj∗−ΔtI(j−1)∗,IAj−ΔtIAj∗−ΔtIA(j−1)∗,IDj−ΔtIDj∗−ΔtID(j−1)∗,IRj−ΔtIRj∗−ΔtIR(j−1)∗,ITj−ΔtITj∗−ΔtIT(j−1)∗,Rj−ΔtRj∗−ΔtR(j−1)∗,Dj−ΔtDj∗−ΔtD(j−1)∗,Vj−ΔtVj∗−ΔtV(j−1)∗)×ΠVn+1=+α(Δt)αAB(α)Γ(α+2)Vn+1=×∑j=2n[tj−12−β(tj−1)(−β(tj)−β(tj−1)Δtlntj−1+2−β(tj−1)tj−1)×V∗(tj−1,Sj−ΔtSj∗,Ij−ΔtIj∗,IAj−ΔtIAj∗,IDj−ΔtIDj∗,IRj−ΔtIRj∗,ITj−ΔtITj∗,Rj−ΔtRj∗,Dj−ΔtDj∗,Vj−ΔtVj∗)−tj−22−β(tj−2)(−β(tj−1)−β(tj−2)Δtlntj−2+2−β(tj−2)tj−2)×V∗(tj−2,Sj−ΔtSj∗−ΔtS(j−1)∗,Ij−ΔtIj∗−ΔtI(j−1)∗,IAj−ΔtIAj∗−ΔtIA(j−1)∗,IDj−ΔtIDj∗−ΔtID(j−1)∗,IRj−ΔtIRj∗−ΔtIR(j−1)∗,ITj−ΔtITj∗−ΔtIT(j−1)∗,Rj−ΔtRj∗−ΔtR(j−1)∗,Dj−ΔtDj∗−ΔtD(j−1)∗,Vj−ΔtVj∗−ΔtV(j−1)∗)]×ΣVn+1=+α(Δt)α2AB(α)Γ(α+3)Vn+1=×∑j=2n[tj2−β(tj)(−β(tj+1)−β(tj)Δtlntj+2−β(tj)tj)×V∗(tj,Sj,Ij,IAj,IDj,IRj,ITj,Rj,Dj,Vj)−2tj−12−β(tj−1)(−β(tj)−β(tj−1)Δtlntj−1+2−β(tj−1)tj−1)×V∗(tj−1,Sj−ΔtSj∗,Ij−ΔtIj∗,IAj−ΔtIAj∗,IDj−ΔtIDj∗,IRj−ΔtIRj∗,ITj−ΔtITj∗,Rj−ΔtRj∗,Dj−ΔtDj∗,Vj−ΔtVj∗)+tj−22−β(tj−2)(−β(tj−1)−β(tj−2)Δtlntj−2+2−β(tj−2)tj−2)×V∗(tj−2,Sj−ΔtSj∗−ΔtS(j−1)∗,Ij−ΔtIj∗−ΔtI(j−1)∗,IAj−ΔtIAj∗−ΔtIA(j−1)∗,IDj−ΔtIDj∗−ΔtID(j−1)∗,IRj−ΔtIRj∗−ΔtIR(j−1)∗,ITj−ΔtITj∗−ΔtIT(j−1)∗,Rj−ΔtRj∗−ΔtR(j−1)∗,Dj−ΔtDj∗−ΔtD(j−1)∗,Vj−ΔtVj∗−ΔtV(j−1)∗)]×Δ.

For the power-law kernel, we can have the following:

140 Sn+1=(Δt)αΓ(α+1)∑j=2ntj−22−β(tj−2)(−β(tj−1)−β(tj−2)Δtlntj−2+2−β(tj−2)tj−2)Sn+1=×S∗(tj−2,Sj−ΔtSj∗−ΔtS(j−1)∗,Ij−ΔtIj∗−ΔtI(j−1)∗,IAj−ΔtIAj∗−ΔtIA(j−1)∗,IDj−ΔtIDj∗−ΔtID(j−1)∗,IRj−ΔtIRj∗−ΔtIR(j−1)∗,ITj−ΔtITj∗−ΔtIT(j−1)∗,Rj−ΔtRj∗−ΔtR(j−1)∗,Dj−ΔtDj∗−ΔtD(j−1)∗,Vj−ΔtVj∗−ΔtV(j−1)∗)×ΠSn+1=+(Δt)αΓ(α+2)∑j=2n[tj−12−β(tj−1)(−β(tj)−β(tj−1)Δtlntj−1+2−β(tj−1)tj−1)×S∗(tj−1,Sj−ΔtSj∗,Ij−ΔtIj∗,IAj−ΔtIAj∗,IDj−ΔtIDj∗,IRj−ΔtIRj∗,ITj−ΔtITj∗,Rj−ΔtRj∗,Dj−ΔtDj∗,Vj−ΔtVj∗)−tj−22−β(tj−2)(−β(tj−1)−β(tj−2)Δtlntj−2+2−β(tj−2)tj−2)×S∗(tj−2,Sj−ΔtSj∗−ΔtS(j−1)∗,Ij−ΔtIj∗−ΔtI(j−1)∗,IAj−ΔtIAj∗−ΔtIA(j−1)∗,IDj−ΔtIDj∗−ΔtID(j−1)∗,IRj−ΔtIRj∗−ΔtIR(j−1)∗,ITj−ΔtITj∗−ΔtIT(j−1)∗,Rj−ΔtRj∗−ΔtR(j−1)∗,Dj−ΔtDj∗−ΔtD(j−1)∗,Vj−ΔtVj∗−ΔtV(j−1)∗)]Sn+1=×ΣSn+1=+(Δt)α2Γ(α+3)∑j=2n[tj2−β(tj)(−β(tj+1)−β(tj)Δtlntj+2−β(tj)tj)×S∗(tj,Sj,Ij,IAj,IDj,IRj,ITj,Rj,Dj,Vj)−2tj−12−β(tj−1)(−β(tj)−β(tj−1)Δtlntj−1+2−β(tj−1)tj−1)×S∗(tj−1,Sj−ΔtSj∗,Ij−ΔtIj∗,IAj−ΔtIAj∗,IDj−ΔtIDj∗,IRj−ΔtIRj∗,ITj−ΔtITj∗,Rj−ΔtRj∗,Dj−ΔtDj∗,Vj−ΔtVj∗)+tj−22−β(tj−2)(−β(tj−1)−β(tj−2)Δtlntj−2+2−β(tj−2)tj−2)×S∗(tj−2,Sj−ΔtSj∗−ΔtS(j−1)∗,Ij−ΔtIj∗−ΔtI(j−1)∗,IAj−ΔtIAj∗−ΔtIA(j−1)∗,IDj−ΔtIDj∗−ΔtID(j−1)∗,IRj−ΔtIRj∗−ΔtIR(j−1)∗,ITj−ΔtITj∗−ΔtIT(j−1)∗,Rj−ΔtRj∗−ΔtR(j−1)∗,Dj−ΔtDj∗−ΔtD(j−1)∗,Vj−ΔtVj∗−ΔtV(j−1)∗)]Sn+1=×Δ,In+1=(Δt)αΓ(α+1)∑j=2ntj−22−β(tj−2)(−β(tj−1)−β(tj−2)Δtlntj−2+2−β(tj−2)tj−2)In+1=×I∗(tj−2,Sj−ΔtSj∗−ΔtS(j−1)∗,Ij−ΔtIj∗−ΔtI(j−1)∗,IAj−ΔtIAj∗−ΔtIA(j−1)∗,IDj−ΔtIDj∗−ΔtID(j−1)∗,IRj−ΔtIRj∗−ΔtIR(j−1)∗,ITj−ΔtITj∗−ΔtIT(j−1)∗,Rj−ΔtRj∗−ΔtR(j−1)∗,Dj−ΔtDj∗−ΔtD(j−1)∗,Vj−ΔtVj∗−ΔtV(j−1)∗)×ΠIn+1=+(Δt)αΓ(α+2)∑j=2n[tj−12−β(tj−1)(−β(tj)−β(tj−1)Δtlntj−1+2−β(tj−1)tj−1)×I∗(tj−1,Sj−ΔtSj∗,Ij−ΔtIj∗,IAj−ΔtIAj∗,IDj−ΔtIDj∗,IRj−ΔtIRj∗,ITj−ΔtITj∗,Rj−ΔtRj∗,Dj−ΔtDj∗,Vj−ΔtVj∗)−tj−22−β(tj−2)(−β(tj−1)−β(tj−2)Δtlntj−2+2−β(tj−2)tj−2)×I∗(tj−2,Sj−ΔtSj∗−ΔtS(j−1)∗,Ij−ΔtIj∗−ΔtI(j−1)∗,IAj−ΔtIAj∗−ΔtIA(j−1)∗,IDj−ΔtIDj∗−ΔtID(j−1)∗,IRj−ΔtIRj∗−ΔtIR(j−1)∗,ITj−ΔtITj∗−ΔtIT(j−1)∗,Rj−ΔtRj∗−ΔtR(j−1)∗,Dj−ΔtDj∗−ΔtD(j−1)∗,Vj−ΔtVj∗−ΔtV(j−1)∗)]In+1=×ΣIn+1=+α(Δt)α2Γ(α+3)∑j=2n[tj2−β(tj)(−β(tj+1)−β(tj)Δtlntj+2−β(tj)tj)×I∗(tj,Sj,Ij,IAj,IDj,IRj,ITj,Rj,Dj,Vj)−2tj−12−β(tj−1)(−β(tj)−β(tj−1)Δtlntj−1+2−β(tj−1)tj−1)×I∗(tj−1,Sj−ΔtSj∗,Ij−ΔtIj∗,IAj−ΔtIAj∗,IDj−ΔtIDj∗,IRj−ΔtIRj∗,ITj−ΔtITj∗,Rj−ΔtRj∗,Dj−ΔtDj∗,Vj−ΔtVj∗)+tj−22−β(tj−2)(−β(tj−1)−β(tj−2)Δtlntj−2+2−β(tj−2)tj−2)×I∗(tj−2,Sj−ΔtSj∗−ΔtS(j−1)∗,Ij−ΔtIj∗−ΔtI(j−1)∗,IAj−ΔtIAj∗−ΔtIA(j−1)∗,IDj−ΔtIDj∗−ΔtID(j−1)∗,IRj−ΔtIRj∗−ΔtIR(j−1)∗,ITj−ΔtITj∗−ΔtIT(j−1)∗,Rj−ΔtRj∗−ΔtR(j−1)∗,Dj−ΔtDj∗−ΔtD(j−1)∗,Vj−ΔtVj∗−ΔtV(j−1)∗)]In+1=×Δ,IAn+1=(Δt)αΓ(α+1)∑j=2ntj−22−β(tj−2)(−β(tj−1)−β(tj−2)Δtlntj−2+2−β(tj−2)tj−2)IAn+1=×IA∗(tj−2,Sj−ΔtSj∗−ΔtS(j−1)∗,Ij−ΔtIj∗−ΔtI(j−1)∗,IAj−ΔtIAj∗−ΔtIA(j−1)∗,IDj−ΔtIDj∗−ΔtID(j−1)∗,IRj−ΔtIRj∗−ΔtIR(j−1)∗,ITj−ΔtITj∗−ΔtIT(j−1)∗,Rj−ΔtRj∗−ΔtR(j−1)∗,Dj−ΔtDj∗−ΔtD(j−1)∗,Vj−ΔtVj∗−ΔtV(j−1)∗)×ΠIAn+1=+(Δt)αΓ(α+2)∑j=2n[tj−12−β(tj−1)(−β(tj)−β(tj−1)Δtlntj−1+2−β(tj−1)tj−1)×IA∗(tj−1,Sj−ΔtSj∗,Ij−ΔtIj∗,IAj−ΔtIAj∗,IDj−ΔtIDj∗,IRj−ΔtIRj∗,ITj−ΔtITj∗,Rj−ΔtRj∗,Dj−ΔtDj∗,Vj−ΔtVj∗)−tj−22−β(tj−2)(−β(tj−1)−β(tj−2)Δtlntj−2+2−β(tj−2)tj−2)×IA∗(tj−2,Sj−ΔtSj∗−ΔtS(j−1)∗,Ij−ΔtIj∗−ΔtI(j−1)∗,IAj−ΔtIAj∗−ΔtIA(j−1)∗,IDj−ΔtIDj∗−ΔtID(j−1)∗,IRj−ΔtIRj∗−ΔtIR(j−1)∗,ITj−ΔtITj∗−ΔtIT(j−1)∗,Rj−ΔtRj∗−ΔtR(j−1)∗,Dj−ΔtDj∗−ΔtD(j−1)∗,Vj−ΔtVj∗−ΔtV(j−1)∗)]IAn+1=×ΣIAn+1=+(Δt)α2Γ(α+3)∑j=2n[tj2−β(tj)(−β(tj+1)−β(tj)Δtlntj+2−β(tj)tj)×IA∗(tj,Sj,Ij,IAj,IDj,IRj,ITj,Rj,Dj,Vj)−2tj−12−β(tj−1)(−β(tj)−β(tj−1)Δtlntj−1+2−β(tj−1)tj−1)×IA∗(tj−1,Sj−ΔtSj∗,Ij−ΔtIj∗,IAj−ΔtIAj∗,IDj−ΔtIDj∗,IRj−ΔtIRj∗,ITj−ΔtITj∗,Rj−ΔtRj∗,Dj−ΔtDj∗,Vj−ΔtVj∗)+tj−22−β(tj−2)(−β(tj−1)−β(tj−2)Δtlntj−2+2−β(tj−2)tj−2)×IA∗(tj−2,Sj−ΔtSj∗−ΔtS(j−1)∗,Ij−ΔtIj∗−ΔtI(j−1)∗,IAj−ΔtIAj∗−ΔtIA(j−1)∗,IDj−ΔtIDj∗−ΔtID(j−1)∗,IRj−ΔtIRj∗−ΔtIR(j−1)∗,ITj−ΔtITj∗−ΔtIT(j−1)∗,Rj−ΔtRj∗−ΔtR(j−1)∗,Dj−ΔtDj∗−ΔtD(j−1)∗,Vj−ΔtVj∗−ΔtV(j−1)∗)]IAn+1=×Δ,IDn+1=(Δt)αΓ(α+1)∑j=2ntj−22−β(tj−2)(−β(tj−1)−β(tj−2)Δtlntj−2+2−β(tj−2)tj−2)IDn+1=×ID∗(tj−2,Sj−ΔtSj∗−ΔtS(j−1)∗,Ij−ΔtIj∗−ΔtI(j−1)∗,IAj−ΔtIAj∗−ΔtIA(j−1)∗,IDj−ΔtIDj∗−ΔtID(j−1)∗,IRj−ΔtIRj∗−ΔtIR(j−1)∗,ITj−ΔtITj∗−ΔtIT(j−1)∗,Rj−ΔtRj∗−ΔtR(j−1)∗,Dj−ΔtDj∗−ΔtD(j−1)∗,Vj−ΔtVj∗−ΔtV(j−1)∗)×ΠIDn+1=+(Δt)αΓ(α+2)∑j=2n[tj−12−β(tj−1)(−β(tj)−β(tj−1)Δtlntj−1+2−β(tj−1)tj−1)×ID∗(tj−1,Sj−ΔtSj∗,Ij−ΔtIj∗,IAj−ΔtIAj∗,IDj−ΔtIDj∗,IRj−ΔtIRj∗,ITj−ΔtITj∗,Rj−ΔtRj∗,Dj−ΔtDj∗,Vj−ΔtVj∗)−tj−22−β(tj−2)(−β(tj−1)−β(tj−2)Δtlntj−2+2−β(tj−2)tj−2)×ID∗(tj−2,Sj−ΔtSj∗−ΔtS(j−1)∗,Ij−ΔtIj∗−ΔtI(j−1)∗,IAj−ΔtIAj∗−ΔtIA(j−1)∗,IDj−ΔtIDj∗−ΔtID(j−1)∗,IRj−ΔtIRj∗−ΔtIR(j−1)∗,ITj−ΔtITj∗−ΔtIT(j−1)∗,Rj−ΔtRj∗−ΔtR(j−1)∗,Dj−ΔtDj∗−ΔtD(j−1)∗,Vj−ΔtVj∗−ΔtV(j−1)∗)]IDn+1=×ΣIDn+1=+(Δt)α2Γ(α+3)∑j=2n[tj2−β(tj)(−β(tj+1)−β(tj)Δtlntj+2−β(tj)tj)×ID∗(tj,Sj,Ij,IAj,IDj,IRj,ITj,Rj,Dj,Vj)−2tj−12−β(tj−1)(−β(tj)−β(tj−1)Δtlntj−1+2−β(tj−1)tj−1)×ID∗(tj−1,Sj−ΔtSj∗,Ij−ΔtIj∗,IAj−ΔtIAj∗,IDj−ΔtIDj∗,IRj−ΔtIRj∗,ITj−ΔtITj∗,Rj−ΔtRj∗,Dj−ΔtDj∗,Vj−ΔtVj∗)+tj−22−β(tj−2)(−β(tj−1)−β(tj−2)Δtlntj−2+2−β(tj−2)tj−2)×ID∗(tj−2,Sj−ΔtSj∗−ΔtS(j−1)∗,Ij−ΔtIj∗−ΔtI(j−1)∗,IAj−ΔtIAj∗−ΔtIA(j−1)∗,IDj−ΔtIDj∗−ΔtID(j−1)∗,IRj−ΔtIRj∗−ΔtIR(j−1)∗,ITj−ΔtITj∗−ΔtIT(j−1)∗,Rj−ΔtRj∗−ΔtR(j−1)∗,Dj−ΔtDj∗−ΔtD(j−1)∗,Vj−ΔtVj∗−ΔtV(j−1)∗)]IDn+1=×Δ,IRn+1=(Δt)αΓ(α+1)∑j=2ntj−22−β(tj−2)(−β(tj−1)−β(tj−2)Δtlntj−2+2−β(tj−2)tj−2)IRn+1=×IR∗(tj−2,Sj−ΔtSj∗−ΔtS(j−1)∗,Ij−ΔtIj∗−ΔtI(j−1)∗,IAj−ΔtIAj∗−ΔtIA(j−1)∗,IDj−ΔtIDj∗−ΔtID(j−1)∗,IRj−ΔtIRj∗−ΔtIR(j−1)∗,ITj−ΔtITj∗−ΔtIT(j−1)∗,Rj−ΔtRj∗−ΔtR(j−1)∗,Dj−ΔtDj∗−ΔtD(j−1)∗,Vj−ΔtVj∗−ΔtV(j−1)∗)×ΠIRn+1=+(Δt)αΓ(α+2)∑j=2n[tj−12−β(tj−1)(−β(tj)−β(tj−1)Δtlntj−1+2−β(tj−1)tj−1)×IR∗(tj−1,Sj−ΔtSj∗,Ij−ΔtIj∗,IAj−ΔtIAj∗,IDj−ΔtIDj∗,IRj−ΔtIRj∗,ITj−ΔtITj∗,Rj−ΔtRj∗,Dj−ΔtDj∗,Vj−ΔtVj∗)−tj−22−β(tj−2)(−β(tj−1)−β(tj−2)Δtlntj−2+2−β(tj−2)tj−2)×IR∗(tj−2,Sj−ΔtSj∗−ΔtS(j−1)∗,Ij−ΔtIj∗−ΔtI(j−1)∗,IAj−ΔtIAj∗−ΔtIA(j−1)∗,IDj−ΔtIDj∗−ΔtID(j−1)∗,IRj−ΔtIRj∗−ΔtIR(j−1)∗,ITj−ΔtITj∗−ΔtIT(j−1)∗,Rj−ΔtRj∗−ΔtR(j−1)∗,Dj−ΔtDj∗−ΔtD(j−1)∗,Vj−ΔtVj∗−ΔtV(j−1)∗)]IRn+1=×ΣIRn+1=+(Δt)α2Γ(α+3)∑j=2n[tj2−β(tj)(−β(tj+1)−β(tj)Δtlntj+2−β(tj)tj)×IR∗(tj,Sj,Ij,IAj,IDj,IRj,ITj,Rj,Dj,Vj)−2tj−12−β(tj−1)(−β(tj)−β(tj−1)Δtlntj−1+2−β(tj−1)tj−1)×IR∗(tj−1,Sj−ΔtSj∗,Ij−ΔtIj∗,IAj−ΔtIAj∗,IDj−ΔtIDj∗,IRj−ΔtIRj∗,ITj−ΔtITj∗,Rj−ΔtRj∗,Dj−ΔtDj∗,Vj−ΔtVj∗)+tj−22−β(tj−2)(−β(tj−1)−β(tj−2)Δtlntj−2+2−β(tj−2)tj−2)×IR∗(tj−2,Sj−ΔtSj∗−ΔtS(j−1)∗,Ij−ΔtIj∗−ΔtI(j−1)∗,IAj−ΔtIAj∗−ΔtIA(j−1)∗,IDj−ΔtIDj∗−ΔtID(j−1)∗,IRj−ΔtIRj∗−ΔtIR(j−1)∗,ITj−ΔtITj∗−ΔtIT(j−1)∗,Rj−ΔtRj∗−ΔtR(j−1)∗,Dj−ΔtDj∗−ΔtD(j−1)∗,Vj−ΔtVj∗−ΔtV(j−1)∗)]IRn+1=×Δ,ITn+1=(Δt)αΓ(α+1)∑j=2ntj−22−β(tj−2)(−β(tj−1)−β(tj−2)Δtlntj−2+2−β(tj−2)tj−2)ITn+1=×IT∗(tj−2,Sj−ΔtSj∗−ΔtS(j−1)∗,Ij−ΔtIj∗−ΔtI(j−1)∗,IAj−ΔtIAj∗−ΔtIA(j−1)∗,IDj−ΔtIDj∗−ΔtID(j−1)∗,IRj−ΔtIRj∗−ΔtIR(j−1)∗,ITj−ΔtITj∗−ΔtIT(j−1)∗,Rj−ΔtRj∗−ΔtR(j−1)∗,Dj−ΔtDj∗−ΔtD(j−1)∗,Vj−ΔtVj∗−ΔtV(j−1)∗)×ΠITn+1=+(Δt)αΓ(α+2)∑j=2n[tj−12−β(tj−1)(−β(tj)−β(tj−1)Δtlntj−1+2−β(tj−1)tj−1)×IT∗(tj−1,Sj−ΔtSj∗,Ij−ΔtIj∗,IAj−ΔtIAj∗,IDj−ΔtIDj∗,IRj−ΔtIRj∗,ITj−ΔtITj∗,Rj−ΔtRj∗,Dj−ΔtDj∗,Vj−ΔtVj∗)−tj−22−β(tj−2)(−β(tj−1)−β(tj−2)Δtlntj−2+2−β(tj−2)tj−2)×IT∗(tj−2,Sj−ΔtSj∗−ΔtS(j−1)∗,Ij−ΔtIj∗−ΔtI(j−1)∗,IAj−ΔtIAj∗−ΔtIA(j−1)∗,IDj−ΔtIDj∗−ΔtID(j−1)∗,IRj−ΔtIRj∗−ΔtIR(j−1)∗,ITj−ΔtITj∗−ΔtIT(j−1)∗,Rj−ΔtRj∗−ΔtR(j−1)∗,Dj−ΔtDj∗−ΔtD(j−1)∗,Vj−ΔtVj∗−ΔtV(j−1)∗)]ITn+1=×ΣITn+1=+(Δt)α2Γ(α+3)∑j=2n[tj2−β(tj)(−β(tj+1)−β(tj)Δtlntj+2−β(tj)tj)×IT∗(tj,Sj,Ij,IAj,IDj,IRj,ITj,Rj,Dj,Vj)−2tj−12−β(tj−1)(−β(tj)−β(tj−1)Δtlntj−1+2−β(tj−1)tj−1)×IT∗(tj−1,Sj−ΔtSj∗,Ij−ΔtIj∗,IAj−ΔtIAj∗,IDj−ΔtIDj∗,IRj−ΔtIRj∗,ITj−ΔtITj∗,Rj−ΔtRj∗,Dj−ΔtDj∗,Vj−ΔtVj∗)+tj−22−β(tj−2)(−β(tj−1)−β(tj−2)Δtlntj−2+2−β(tj−2)tj−2)×IT∗(tj−2,Sj−ΔtSj∗−ΔtS(j−1)∗,Ij−ΔtIj∗−ΔtI(j−1)∗,IAj−ΔtIAj∗−ΔtIA(j−1)∗,IDj−ΔtIDj∗−ΔtID(j−1)∗,IRj−ΔtIRj∗−ΔtIR(j−1)∗,ITj−ΔtITj∗−ΔtIT(j−1)∗,Rj−ΔtRj∗−ΔtR(j−1)∗,Dj−ΔtDj∗−ΔtD(j−1)∗,Vj−ΔtVj∗−ΔtV(j−1)∗)]ITn+1=×Δ,Rn+1=(Δt)αΓ(α+1)∑j=2ntj−22−β(tj−2)(−β(tj−1)−β(tj−2)Δtlntj−2+2−β(tj−2)tj−2)Rn+1=×R∗(tj−2,Sj−ΔtSj∗−ΔtS(j−1)∗,Ij−ΔtIj∗−ΔtI(j−1)∗,IAj−ΔtIAj∗−ΔtIA(j−1)∗,IDj−ΔtIDj∗−ΔtID(j−1)∗,IRj−ΔtIRj∗−ΔtIR(j−1)∗,ITj−ΔtITj∗−ΔtIT(j−1)∗,Rj−ΔtRj∗−ΔtR(j−1)∗,Dj−ΔtDj∗−ΔtD(j−1)∗,Vj−ΔtVj∗−ΔtV(j−1)∗)×ΠRn+1=+(Δt)αΓ(α+2)∑j=2n[tj−12−β(tj−1)(−β(tj)−β(tj−1)Δtlntj−1+2−β(tj−1)tj−1)×R∗(tj−1,Sj−ΔtSj∗,Ij−ΔtIj∗,IAj−ΔtIAj∗,IDj−ΔtIDj∗,IRj−ΔtIRj∗,ITj−ΔtITj∗,Rj−ΔtRj∗,Dj−ΔtDj∗,Vj−ΔtVj∗)−tj−22−β(tj−2)(−β(tj−1)−β(tj−2)Δtlntj−2+2−β(tj−2)tj−2)×R∗(tj−2,Sj−ΔtSj∗−ΔtS(j−1)∗,Ij−ΔtIj∗−ΔtI(j−1)∗,IAj−ΔtIAj∗−ΔtIA(j−1)∗,IDj−ΔtIDj∗−ΔtID(j−1)∗,IRj−ΔtIRj∗−ΔtIR(j−1)∗,ITj−ΔtITj∗−ΔtIT(j−1)∗,Rj−ΔtRj∗−ΔtR(j−1)∗,Dj−ΔtDj∗−ΔtD(j−1)∗,Vj−ΔtVj∗−ΔtV(j−1)∗)]Rn+1=×ΣRn+1=+(Δt)α2Γ(α+3)∑j=2n[tj2−β(tj)(−β(tj+1)−β(tj)Δtlntj+2−β(tj)tj)×R∗(tj,Sj,Ij,IAj,IDj,IRj,ITj,Rj,Dj,Vj)−2tj−12−β(tj−1)(−β(tj)−β(tj−1)Δtlntj−1+2−β(tj−1)tj−1)×R∗(tj−1,Sj−ΔtSj∗,Ij−ΔtIj∗,IAj−ΔtIAj∗,IDj−ΔtIDj∗,IRj−ΔtIRj∗,ITj−ΔtITj∗,Rj−ΔtRj∗,Dj−ΔtDj∗,Vj−ΔtVj∗)+tj−22−β(tj−2)(−β(tj−1)−β(tj−2)Δtlntj−2+2−β(tj−2)tj−2)×R∗(tj−2,Sj−ΔtSj∗−ΔtS(j−1)∗,Ij−ΔtIj∗−ΔtI(j−1)∗,IAj−ΔtIAj∗−ΔtIA(j−1)∗,IDj−ΔtIDj∗−ΔtID(j−1)∗,IRj−ΔtIRj∗−ΔtIR(j−1)∗,ITj−ΔtITj∗−ΔtIT(j−1)∗,Rj−ΔtRj∗−ΔtR(j−1)∗,Dj−ΔtDj∗−ΔtD(j−1)∗,Vj−ΔtVj∗−ΔtV(j−1)∗)]Rn+1=×Δ,Dn+1=(Δt)αΓ(α+1)∑j=2ntj−22−β(tj−2)(−β(tj−1)−β(tj−2)Δtlntj−2+2−β(tj−2)tj−2)Dn+1=×D∗(tj−2,Sj−ΔtSj∗−ΔtS(j−1)∗,Ij−ΔtIj∗−ΔtI(j−1)∗,IAj−ΔtIAj∗−ΔtIA(j−1)∗,IDj−ΔtIDj∗−ΔtID(j−1)∗,IRj−ΔtIRj∗−ΔtIR(j−1)∗,ITj−ΔtITj∗−ΔtIT(j−1)∗,Rj−ΔtRj∗−ΔtR(j−1)∗,Dj−ΔtDj∗−ΔtD(j−1)∗,Vj−ΔtVj∗−ΔtV(j−1)∗)×ΠDn+1=+(Δt)αΓ(α+2)∑j=2n[tj−12−β(tj−1)(−β(tj)−β(tj−1)Δtlntj−1+2−β(tj−1)tj−1)×D∗(tj−1,Sj−ΔtSj∗,Ij−ΔtIj∗,IAj−ΔtIAj∗,IDj−ΔtIDj∗,IRj−ΔtIRj∗,ITj−ΔtITj∗,Rj−ΔtRj∗,Dj−ΔtDj∗,Vj−ΔtVj∗)−tj−22−β(tj−2)(−β(tj−1)−β(tj−2)Δtlntj−2+2−β(tj−2)tj−2)×D∗(tj−2,Sj−ΔtSj∗−ΔtS(j−1)∗,Ij−ΔtIj∗−ΔtI(j−1)∗,IAj−ΔtIAj∗−ΔtIA(j−1)∗,IDj−ΔtIDj∗−ΔtID(j−1)∗,IRj−ΔtIRj∗−ΔtIR(j−1)∗,ITj−ΔtITj∗−ΔtIT(j−1)∗,Rj−ΔtRj∗−ΔtR(j−1)∗,Dj−ΔtDj∗−ΔtD(j−1)∗,Vj−ΔtVj∗−ΔtV(j−1)∗)]Dn+1=×ΣDn+1=+(Δt)α2Γ(α+3)∑j=2n[tj2−β(tj)(−β(tj+1)−β(tj)Δtlntj+2−β(tj)tj)×D∗(tj,Sj,Ij,IAj,IDj,IRj,ITj,Rj,Dj,Vj)−2tj−12−β(tj−1)(−β(tj)−β(tj−1)Δtlntj−1+2−β(tj−1)tj−1)×D∗(tj−1,Sj−ΔtSj∗,Ij−ΔtIj∗,IAj−ΔtIAj∗,IDj−ΔtIDj∗,IRj−ΔtIRj∗,ITj−ΔtITj∗,Rj−ΔtRj∗,Dj−ΔtDj∗,Vj−ΔtVj∗)+tj−22−β(tj−2)(−β(tj−1)−β(tj−2)Δtlntj−2+2−β(tj−2)tj−2)×D∗(tj−2,Sj−ΔtSj∗−ΔtS(j−1)∗,Ij−ΔtIj∗−ΔtI(j−1)∗,IAj−ΔtIAj∗−ΔtIA(j−1)∗,IDj−ΔtIDj∗−ΔtID(j−1)∗,IRj−ΔtIRj∗−ΔtIR(j−1)∗,ITj−ΔtITj∗−ΔtIT(j−1)∗,Rj−ΔtRj∗−ΔtR(j−1)∗,Dj−ΔtDj∗−ΔtD(j−1)∗,Vj−ΔtVj∗−ΔtV(j−1)∗)]Dn+1=×Δ,Vn+1=(Δt)αΓ(α+1)∑j=2ntj−22−β(tj−2)(−β(tj−1)−β(tj−2)Δtlntj−2+2−β(tj−2)tj−2)Vn+1=×V∗(tj−2,Sj−ΔtSj∗−ΔtS(j−1)∗,Ij−ΔtIj∗−ΔtI(j−1)∗,IAj−ΔtIAj∗−ΔtIA(j−1)∗,IDj−ΔtIDj∗−ΔtID(j−1)∗,IRj−ΔtIRj∗−ΔtIR(j−1)∗,ITj−ΔtITj∗−ΔtIT(j−1)∗,Rj−ΔtRj∗−ΔtR(j−1)∗,Dj−ΔtDj∗−ΔtD(j−1)∗,Vj−ΔtVj∗−ΔtV(j−1)∗)×ΠVn+1=+(Δt)αΓ(α+2)∑j=2n[tj−12−β(tj−1)(−β(tj)−β(tj−1)Δtlntj−1+2−β(tj−1)tj−1)×V∗(tj−1,Sj−ΔtSj∗,Ij−ΔtIj∗,IAj−ΔtIAj∗,IDj−ΔtIDj∗,IRj−ΔtIRj∗,ITj−ΔtITj∗,Rj−ΔtRj∗,Dj−ΔtDj∗,Vj−ΔtVj∗)−tj−22−β(tj−2)(−β(tj−1)−β(tj−2)Δtlntj−2+2−β(tj−2)tj−2)×V∗(tj−2,Sj−ΔtSj∗−ΔtS(j−1)∗,Ij−ΔtIj∗−ΔtI(j−1)∗,IAj−ΔtIAj∗−ΔtIA(j−1)∗,IDj−ΔtIDj∗−ΔtID(j−1)∗,IRj−ΔtIRj∗−ΔtIR(j−1)∗,ITj−ΔtITj∗−ΔtIT(j−1)∗,Rj−ΔtRj∗−ΔtR(j−1)∗,Dj−ΔtDj∗−ΔtD(j−1)∗,Vj−ΔtVj∗−ΔtV(j−1)∗)]Vn+1=×ΣVn+1=+(Δt)α2Γ(α+3)∑j=2n[tj2−β(tj)(−β(tj+1)−β(tj)Δtlntj+2−β(tj)tj)×V∗(tj,Sj,Ij,IAj,IDj,IRj,ITj,Rj,Dj,Vj)−2tj−12−β(tj−1)(−β(tj)−β(tj−1)Δtlntj−1+2−β(tj−1)tj−1)×V∗(tj−1,Sj−ΔtSj∗,Ij−ΔtIj∗,IAj−ΔtIAj∗,IDj−ΔtIDj∗,IRj−ΔtIRj∗,ITj−ΔtITj∗,Rj−ΔtRj∗,Dj−ΔtDj∗,Vj−ΔtVj∗)+tj−22−β(tj−2)(−β(tj−1)−β(tj−2)Δtlntj−2+2−β(tj−2)tj−2)×V∗(tj−2,Sj−ΔtSj∗−ΔtS(j−1)∗,Ij−ΔtIj∗−ΔtI(j−1)∗,IAj−ΔtIAj∗−ΔtIA(j−1)∗,IDj−ΔtIDj∗−ΔtID(j−1)∗,IRj−ΔtIRj∗−ΔtIR(j−1)∗,ITj−ΔtITj∗−ΔtIT(j−1)∗,Rj−ΔtRj∗−ΔtR(j−1)∗,Dj−ΔtDj∗−ΔtD(j−1)∗,Vj−ΔtVj∗−ΔtV(j−1)∗)]Vn+1=×Δ.

## Numerical simulation

In this section, using the numerical solutions obtained in the previous section, we present a numerical method for all cases. The numerical simulations are depicted for different values of fractional order and fractal dimension as presented in Figs. 26–37.

141 $$\begin{aligned}& {}_{0}^{FFM}D_{t}^{\alpha ,\beta }S = \Lambda - \bigl( \delta ( t ) \bigl( \alpha I^{\ast }+w\beta I_{D}^{\ast }+ \gamma wI_{A}^{ \ast }+w\delta _{1}I_{R}^{\ast }+w \delta _{2}I_{T}^{\ast } \bigr) + \gamma _{1}+\mu _{1} \bigr) S, \\& {}_{0}^{FFM}D_{t}^{\alpha ,\beta }I = \bigl( \delta ( t ) \bigl( \alpha I^{\ast }+w\beta I_{D}^{\ast }+ \gamma wI_{A}^{\ast }+w \delta _{1}I_{R}^{\ast }+w \delta _{2}I_{T}^{\ast } \bigr) \bigr) S- ( \varepsilon +\xi +\lambda +\mu _{1} ) I, \\& {}_{0}^{FFM}D_{t}^{\alpha ,\beta }I_{A} = \xi I- ( \theta +\mu + \chi +\mu _{1} ) I_{A}, \\& {}_{0}^{FFM}D_{t}^{\alpha ,\beta }I_{D} = \varepsilon I- ( \eta + \varphi +\mu _{1} ) I_{D}, \\& {}_{0}^{FFM}D_{t}^{\alpha ,\beta }I_{R} = \eta I_{D}+\theta I_{A}- ( v+\xi +\mu _{1} ) I_{R}, \\& {}_{0}^{FFM}D_{t}^{\alpha ,\tau }I_{T} = \mu I_{A}+vI_{R}- ( \sigma +\tau +\mu _{1} ) I_{T}, \\& {}_{0}^{FFM}D_{t}^{\alpha ,\tau }R = \lambda I+ \varphi I_{D}+ \chi I_{A}+\xi I_{R}+\sigma I_{T}- ( \Phi +\mu _{1} ) R, \\& {}_{0}^{FFM}D_{t}^{\alpha ,\tau }D = \tau I_{T}, \\& {}_{0}^{FFM}D_{t}^{\alpha ,\tau }V = \gamma _{1}S+\Phi R-\mu _{1}V, \end{aligned}$$

where

142 $$\delta (t)=\left\{\begin{array}{c} d_{0}(1-a_{n})\cos(-b\frac{t-t_{0}}{T}),0<t<t_{0}\\ d_{0},t_{0}<t<t_{1}\\ d_{1}(1-a_{r})\cos(-b\frac{t-t_{1}}{T}),t_{1}<t<t_{2}\\ d_{1},t_{2}<t<t_{3}\\ d_{2}(1-a_{t})\cos(-b\frac{t-t_{2}}{T}),t>t_{3} \end{array}\right\}.$$

Also, the initial conditions are

143 $$\begin{aligned}& S ( 0 ) =800{,}000,\quad\quad I ( 0 ) =3,\quad\quad I_{A} ( 0 ) =0, \quad\quad I_{D} ( 0 ) =0,\quad\quad I_{R} ( 0 ) =0, \\& I_{T} ( 0 ) =0,\quad\quad R ( 0 ) =0,\quad\quad D ( 0 ) =0,\quad \quad V ( 0 ) =0. \end{aligned}$$

Also, the parameters are chosen as follows:

144 $$\begin{aligned}& \Lambda =810{,}000,\quad\quad \eta =0.12,\quad\quad \chi =0.15,\quad\quad v=0.4, \quad\quad \gamma =0.09, \\& \beta =0.75,\quad\quad \gamma _{1}=0.4,\quad\quad \mu _{1}=0.3,\quad\quad \varepsilon =0.161, \quad\quad \tau =0.0199,\\& \Phi =0.015, \quad\quad \lambda =0.0345,\quad\quad \varphi =0.0345,\quad\quad \delta _{1}=0.5, \quad\quad \xi =0.015, \\& \sigma =0.015,\quad\quad \delta _{0}=0.99, \quad\quad \Delta t =900,\quad\quad t_{0}=30,\quad\quad \delta _{2}=0.4,\quad\quad w=0.4, \\& b=0.2,\quad\quad a_{n}=0.1,\quad\quad a_{r}=0.2, \quad\quad a_{t}=\text{0.3},\quad\quad d_{0}=0.02,\quad\quad d_{1}=0.2,\\& d_{2}=0.15. \end{aligned}$$

**Figure 26:**
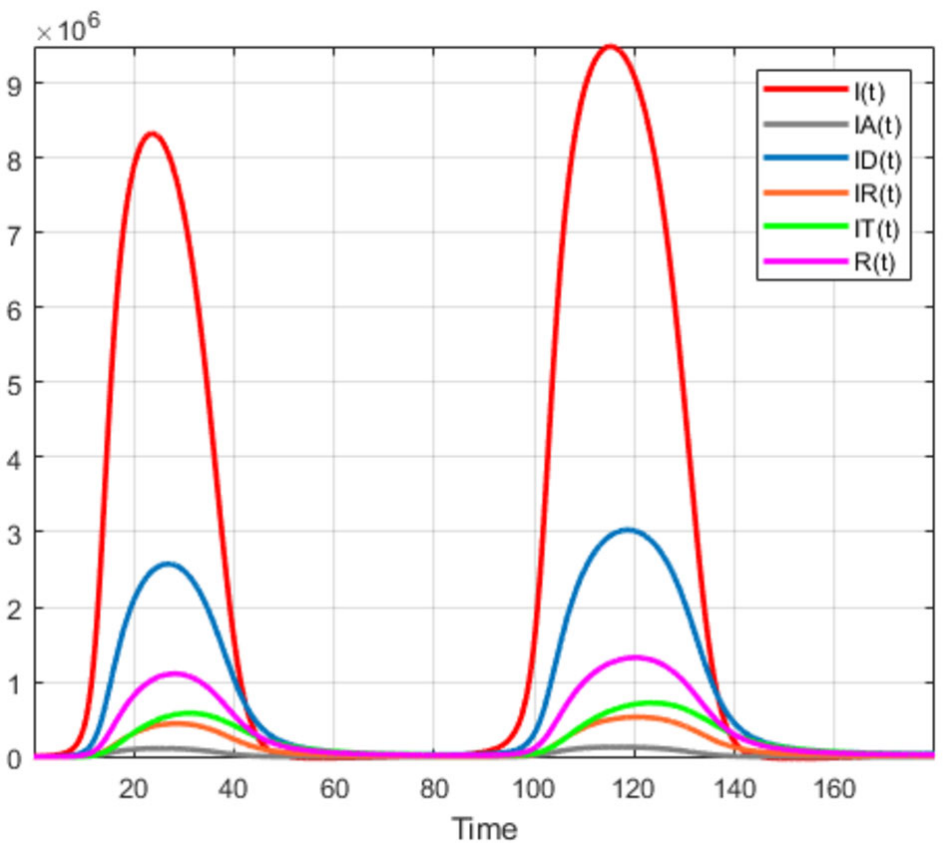
Numerical visualization of COVID-19 model for $\alpha =0.76$

**Figure 27:**
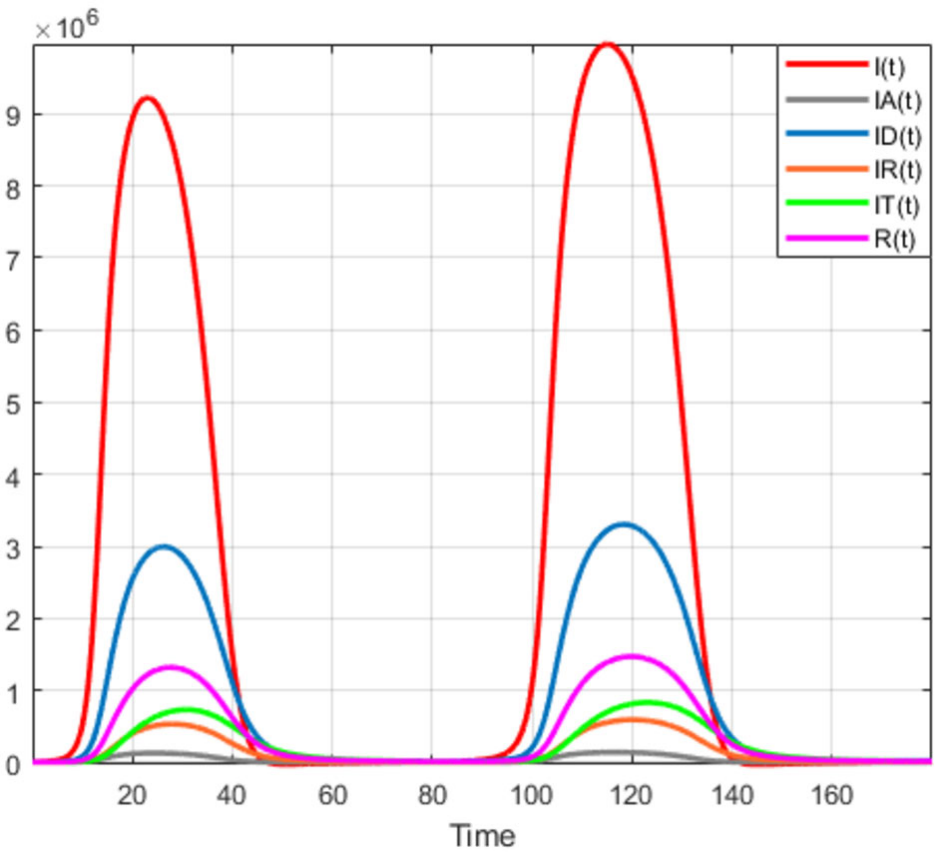
Numerical visualization of COVID-19 model for $\alpha =0.85$

**Figure 28:**
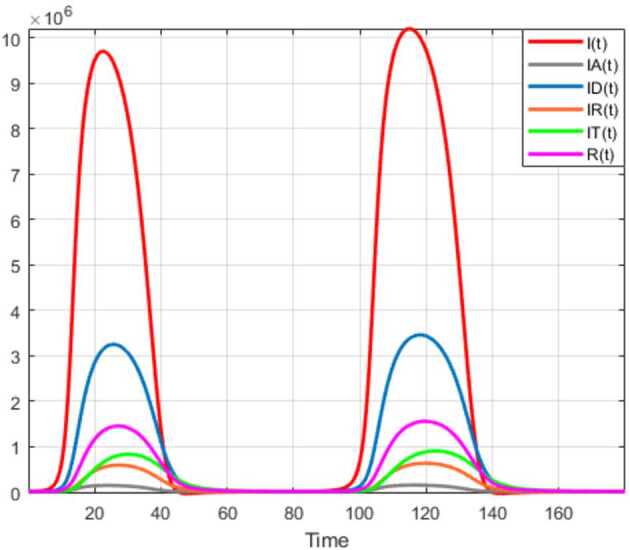
Numerical visualization of COVID-19 model for $\alpha =0.91$

**Figure 29:**
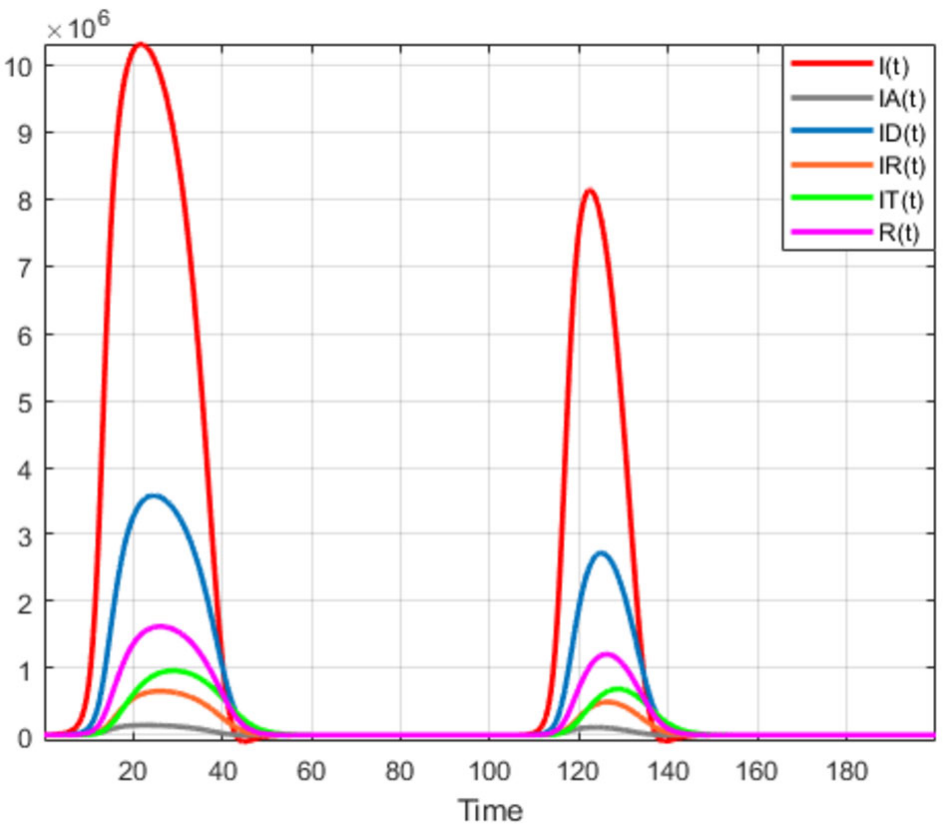
Numerical visualization of COVID-19 model for $\alpha =0.76$

**Figure 30:**
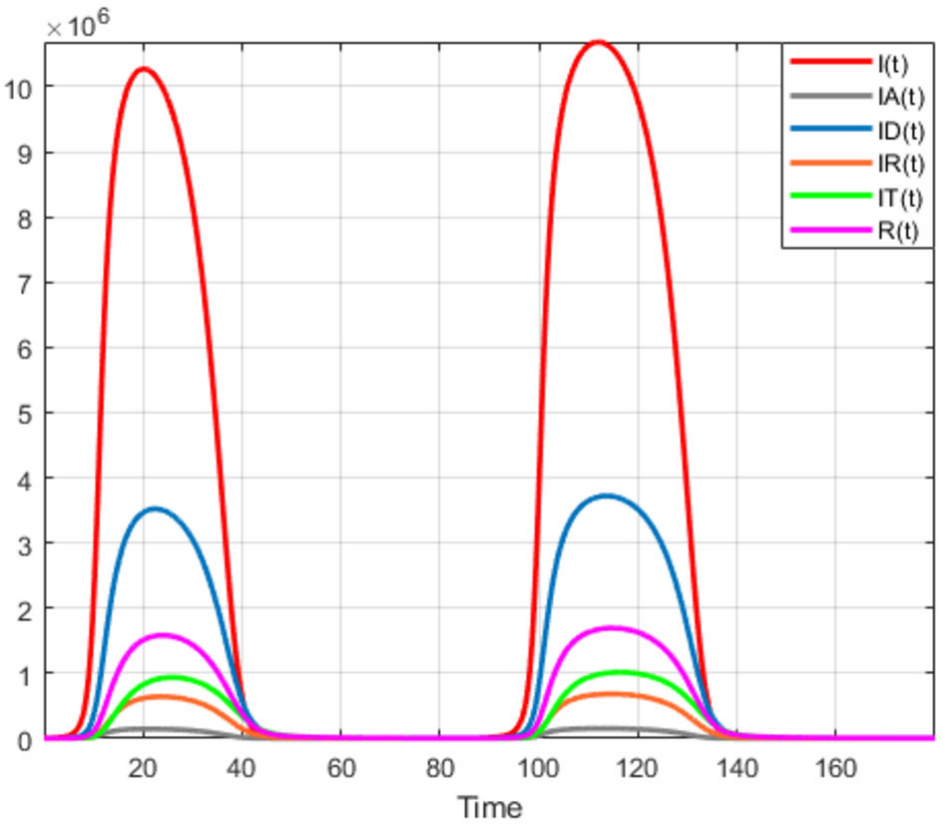
Numerical visualization of COVID-19 model for $\alpha =0.90$, $\beta =0.85$

**Figure 31:**
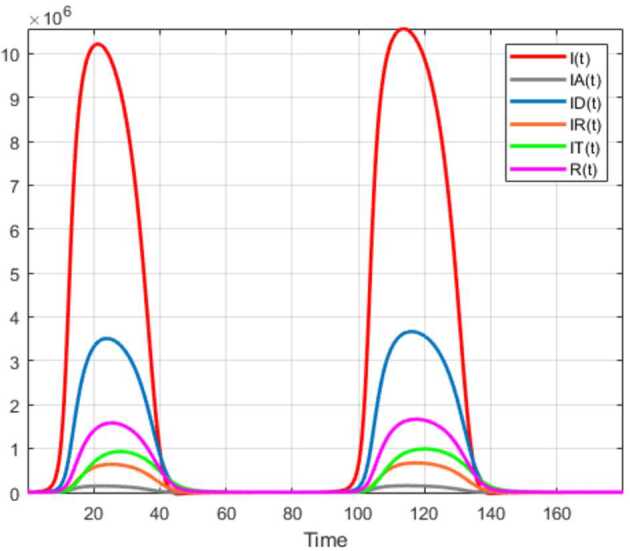
Numerical visualization of COVID-19 model for $\alpha =0.95$, $\beta =0.95$

**Figure 32:**
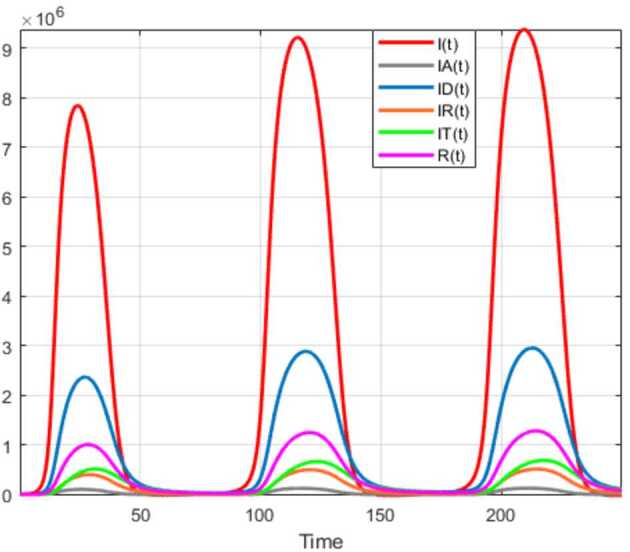
Numerical visualization of COVID-19 model for $\alpha =0.72$

**Figure 33:**
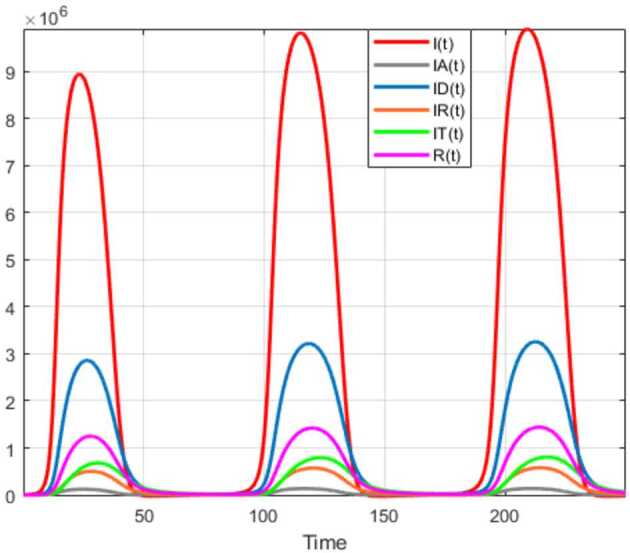
Numerical visualization of COVID-19 model for $\alpha =0.82$

**Figure 34:**
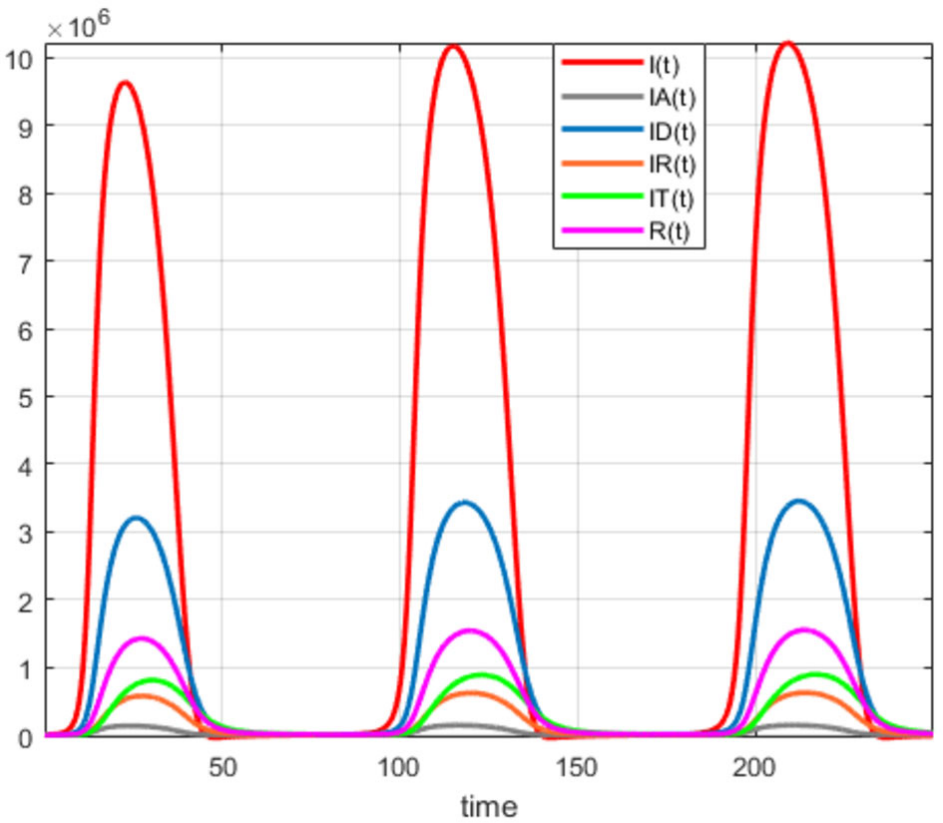
Numerical visualization of COVID-19 model for $\alpha =0.90$

**Figure 35:**
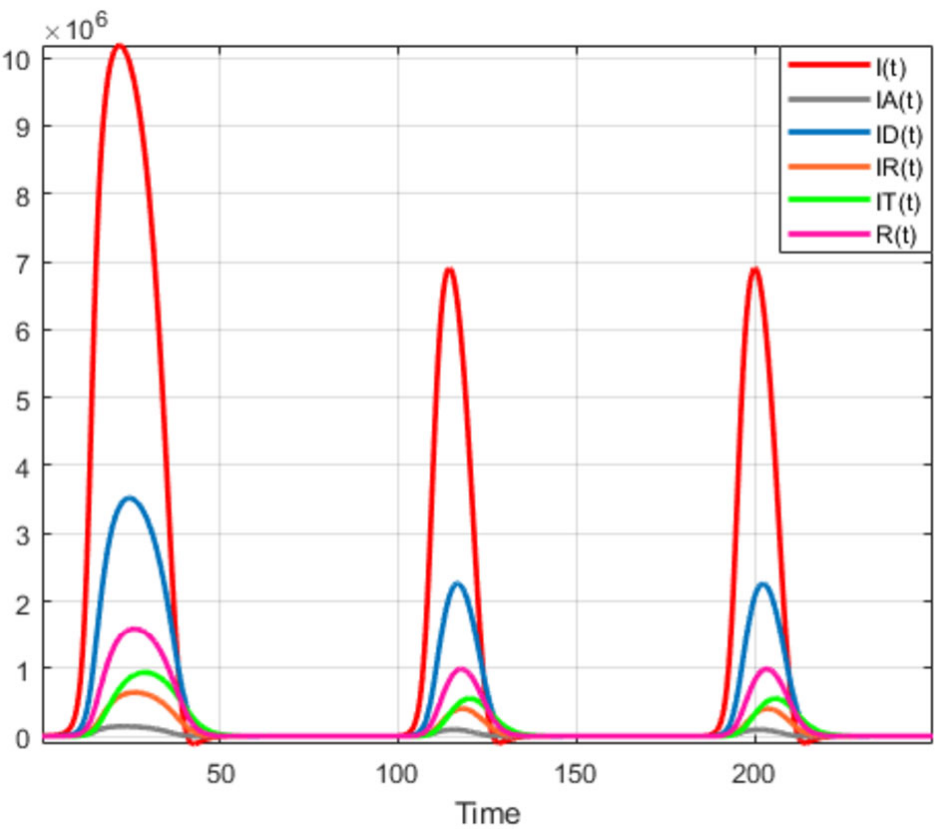
Numerical visualization of COVID-19 model for $\alpha =1$

**Figure 36:**
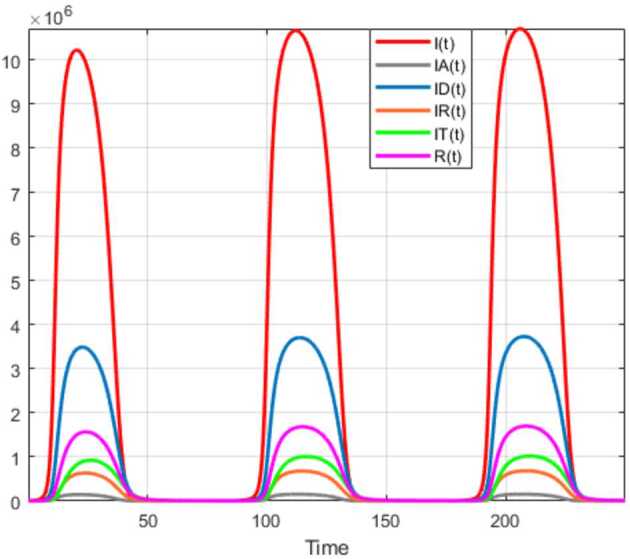
Numerical visualization of COVID-19 model for $\alpha =0.89$, $\beta =0.85$

**Figure 37:**
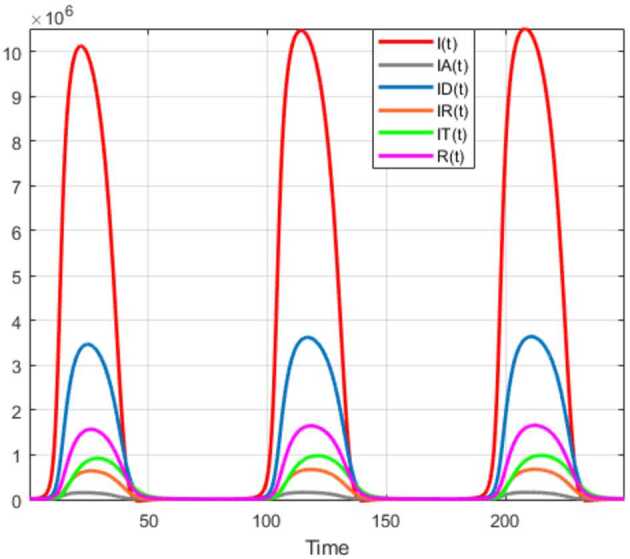
Numerical visualization of COVID-19 model for $\alpha =0.95$, $\beta =0.97$

## Likelihood with hyper-Poisson distribution

Using the suggested numerical model, we obtain the approximate solution $( S^{\ast } ( t ) ,I^{\ast } ( t ) , I_{A}^{ \ast } ( t ) ,I_{D}^{\ast } ( t ) ,I_{R}^{\ast } ( t ) ,I_{T}^{\ast } ( t ) ,R^{\ast } ( t ) ,D^{\ast } ( t ) ,V^{\ast } ( t ) ) $. We are more interested in $I^{\ast } ( t )$, $R^{\ast } ( t )$, and $D^{\ast } ( t ) $ and the approximate solution *I*, *R*, *D* because we have the collected data $z_{I}^{t}$, $z_{R}^{t}$, $z_{D}^{t}$ which represent the number of infections, recovered, and deaths daily. We assume that such follow hyper-Poisson distribution with parameters. The hyper-Poisson distribution is given as follows:

145 $$ P ( X=k ) = \frac{\Gamma ( \beta ) }{\Gamma ( k+\beta ) \Phi ( 1,\beta ,\lambda ) },\quad \lambda >0,k=0,1,2,\ldots,n, $$

where

146 $$ \Phi ( 1,\beta ,\lambda ) =\sum_{k=0}^{\infty } \frac{ ( 1 ) _{k}\lambda ^{k}}{ ( \beta ) _{k}k!}, \quad ( \beta ) _{k}=\beta ( \beta +1 ) \cdots ( \beta +k ) $$

$\Omega _{''}$ with parameters $k_{1}$, $k_{2}$, $k_{3}$

147 $$\begin{aligned}& k_{1} =\Omega _{1}I^{\ast } ( t ), \\& k_{2} =\Omega _{2}R^{\ast } ( t ), \\& k_{3} =\Omega _{3}D^{\ast } ( t ) \end{aligned}$$

and

148 $$\begin{aligned}& z_{I}^{t} \sim HP \bigl( k_{1}=\Omega _{1}I^{\ast } ( t ) \bigr), \\& z_{R}^{t} \sim HP \bigl( k_{2}=\Omega _{2}R^{\ast } ( t ) \bigr), \\& z_{D}^{t} \sim HP \bigl( k_{3}=\Omega _{3}D^{\ast } ( t ) \bigr) . \end{aligned}$$

Here, the parameters $\Omega _{1}$, $\Omega _{2}$, and $\Omega _{3}$ are a combination of collection accuracy and detectability of infected, recovered, and dead. Thus the likelihood function is defined as follows:

149 $$\begin{aligned}& L ( k_{1} ) =\prod_{t=0}^{n}g \bigl( z_{I}^{t}/k_{1} \bigr), \\& L ( k_{2} ) =\prod_{t=0}^{n}g \bigl( z_{R}^{t}/k_{2} \bigr), \\& L ( k_{3} ) =\prod_{t=0}^{n}g \bigl( z_{D}^{t}/k_{3} \bigr) . \end{aligned}$$

Thus

150 $$\begin{aligned}& L ( k_{1} ) =\prod_{t=0}^{n} \frac{\Gamma ( \beta ) \lambda ^{z_{I}^{t}}}{\Gamma ( z_{I}^{t}+\beta ) \Phi ( 1,\beta ,\lambda ) }, \\& L ( k_{2} ) =\prod_{t=0}^{n} \frac{\Gamma ( \beta ) \lambda ^{z_{R}^{t}}}{\Gamma ( z_{R}^{t}+\beta ) \Phi ( 1,\beta ,\lambda ) }, \\& L ( k_{3} ) =\prod_{t=0}^{n} \frac{\Gamma ( \beta ) \lambda ^{z_{D}^{t}}}{\Gamma ( z_{D}^{t}+\beta ) \Phi ( 1,\beta ,\lambda ) }. \end{aligned}$$

Without loss of generality, we consider $L ( k_{1} ) $:

151 $$\begin{aligned}& \begin{aligned}[t] \log L ( k_{1} ) &=\sum _{t=0}^{n}\log \frac{\Gamma ( \beta ) \lambda ^{z_{I}^{t}}}{\Gamma ( z_{I}^{t}+\beta ) \Phi ( 1,\beta ,\lambda ) } \\ &=\sum_{t=0}^{n} \bigl[ \log \Gamma ( \beta ) +z_{I}^{t} \log \bigl( \Omega _{1}I^{\ast } \bigr) -\log \Gamma \bigl( z_{I}^{t}+ \beta \bigr) -\log \Phi \bigl( 1,\beta ,\Omega _{1}I^{\ast } \bigr) \bigr] \end{aligned} \end{aligned}$$

and

152 $$\begin{aligned}& \begin{aligned} \frac{\partial \log L ( k_{1} ) }{\partial z_{I}^{t}} &=\sum_{t=0}^{n} \log ( \Omega _{1} ) +\sum_{t=0}^{n} \log \bigl( I^{ \ast } \bigr) -\sum_{t=0}^{n} \frac{ ( \Gamma ( z_{I}^{t}+\beta ) ) \prime }{\Gamma ( z_{I}^{t}+\beta ) } \\ &=n \bigl[ \log ( \Omega _{1} ) +\log \bigl( I^{\ast } \bigr) \bigr] -\sum_{t=0}^{n} \frac{ ( \Gamma ( z_{I}^{t}+\beta ) ) \prime }{\Gamma ( z_{I}^{t}+\beta ) } \\ &=n\log \bigl( \Omega _{1}I^{\ast } \bigr) -\sum _{t=0}^{n} \frac{ ( \Gamma ( z_{I}^{t}+\beta ) ) \prime }{\Gamma ( z_{I}^{t}+\beta ) }, \end{aligned} \end{aligned}$$

153 $$\begin{aligned}& \begin{aligned}[t] \frac{\partial \log L ( k_{1} ) }{\partial I^{\ast }} &=nz_{I}^{t}\frac{I^{\ast }\prime }{I^{\ast }}-\sum_{t=0}^{n} \frac{\Phi ( 1,\beta ,\Omega _{1}I^{\ast } ) \prime }{\Phi ( 1,\beta ,\Omega _{1}I^{\ast } ) } \\ &=nz_{I}^{t}\frac{I^{\ast }\prime }{I^{\ast }}-n \frac{\Phi ( 1,\beta ,\Omega _{1}I^{\ast } ) \prime }{\Phi ( 1,\beta ,\Omega _{1}I^{\ast } ) }, \end{aligned} \end{aligned}$$

154 $$\begin{aligned}& \begin{aligned}[t] \frac{\partial \log L ( k_{1} ) }{\partial \Omega _{1}} &=nz_{I}^{t} \frac{\Omega _{1}\prime }{\Omega _{1}}-n \frac{\Phi ( 1,\beta ,\Omega _{1}I^{\ast } ) \prime }{\Phi ( 1,\beta ,\Omega _{1}I^{\ast } ) } \\ &=-n \frac{\Phi ( 1,\beta ,\Omega _{1}I^{\ast } ) \prime }{\Phi ( 1,\beta ,\Omega _{1}I^{\ast } ) }, \end{aligned} \end{aligned}$$

155 $$\begin{aligned}& \begin{aligned}[t] L ( k_{2} ) &=\sum _{t=0}^{n}\log \frac{\Gamma ( \beta ) \lambda ^{z_{R}^{t}}}{\Gamma ( z_{R}^{t}+\beta ) \Phi ( 1,\beta ,\lambda ) } \\ &=\sum_{t=0}^{n} \bigl[ \log \Gamma ( \beta ) +z_{R}^{t} \log \lambda -\log \Gamma \bigl( z_{R}^{t}+\beta \bigr) -\log \Phi ( 1,\beta ,\lambda ) \bigr], \end{aligned} \end{aligned}$$

156 $$\begin{aligned}& \begin{aligned} \frac{\partial \log L ( k_{2} ) }{\partial z_{R}^{t}} &=\sum_{t=0}^{n} \log ( \Omega _{2} ) +\sum_{t=0}^{n} \log \bigl( R^{ \ast } \bigr) -\sum_{t=0}^{n} \frac{ ( \Gamma ( z_{R}^{t}+\beta ) ) \prime }{\Gamma ( z_{R}^{t}+\beta ) } \\ &=n \bigl[ \log ( \Omega _{2} ) +\log \bigl( R^{\ast } \bigr) \bigr] -\sum_{t=0}^{n} \frac{ ( \Gamma ( z_{R}^{t}+\beta ) ) \prime }{\Gamma ( z_{R}^{t}+\beta ) } \\ &=n\log \bigl( \Omega _{2}R^{\ast } \bigr) -\sum _{t=0}^{n} \frac{ ( \Gamma ( z_{R}^{t}+\beta ) ) \prime }{\Gamma ( z_{R}^{t}+\beta ) } , \end{aligned} \end{aligned}$$

157 $$\begin{aligned}& \begin{aligned}[t] \frac{\partial \log L ( k_{2} ) }{\partial R^{\ast }} &=nz_{R}^{t}\frac{R^{\ast }\prime }{R^{\ast }}-\sum_{t=0}^{n} \frac{\Phi ( 1,\beta ,\Omega _{2}R^{\ast } ) \prime }{\Phi ( 1,\beta ,\Omega _{2}R^{\ast } ) } \\ &=nz_{R}^{t}\frac{R^{\ast }\prime }{R^{\ast }}-n \frac{\Phi ( 1,\beta ,\Omega _{2}R^{\ast } ) \prime }{\Phi ( 1,\beta ,\Omega _{2}R^{\ast } ) }, \end{aligned} \end{aligned}$$

158 $$\begin{aligned}& \begin{aligned}[t] \frac{\partial \log L ( k_{2} ) }{\partial \Omega _{2}} &=nz_{R}^{t} \frac{\Omega _{2}\prime }{\Omega _{2}}-n \frac{\Phi ( 1,\beta ,\Omega _{2}R^{\ast } ) \prime }{\Phi ( 1,\beta ,\Omega _{2}R^{\ast } ) } \\ &=-n \frac{\Phi ( 1,\beta ,\Omega _{2}R^{\ast } ) \prime }{\Phi ( 1,\beta ,\Omega _{2}R^{\ast } ) } , \end{aligned} \end{aligned}$$

159 $$\begin{aligned}& \begin{aligned}[t] L ( k_{3} ) &=\sum_{t=0}^{n} \log \frac{\Gamma ( \beta ) \lambda ^{z_{D}^{t}}}{\Gamma ( z_{D}^{t}+\beta ) \Phi ( 1,\beta ,\lambda ) } \\ &=\sum_{t=0}^{n} \bigl[ \log \Gamma ( \beta ) +z_{D}^{t} \log \lambda -\log \Gamma \bigl( z_{D}^{t}+\beta \bigr) -\log \Phi ( 1,\beta ,\lambda ) \bigr] , \end{aligned} \end{aligned}$$

160 $$\begin{aligned}& \begin{aligned} \frac{\partial \log L ( k_{3} ) }{\partial z_{D}^{t}} &=\sum_{t=0}^{n} \log ( \Omega _{3} ) +\sum_{t=0}^{n} \log \bigl( D^{ \ast } \bigr) -\sum_{t=0}^{n} \frac{ ( \Gamma ( z_{D}^{t}+\beta ) ) \prime }{\Gamma ( z_{D}^{t}+\beta ) } \\ &=n \bigl[ \log ( \Omega _{3} ) +\log \bigl( D^{\ast } \bigr) \bigr] -\sum_{t=0}^{n} \frac{ ( \Gamma ( z_{D}^{t}+\beta ) ) \prime }{\Gamma ( z_{D}^{t}+\beta ) } \\ &=n\log \bigl( \Omega _{3}D^{\ast } \bigr) -\sum _{t=0}^{n} \frac{ ( \Gamma ( z_{D}^{t}+\beta ) ) \prime }{\Gamma ( z_{D}^{t}+\beta ) } , \end{aligned} \end{aligned}$$

161 $$\begin{aligned}& \begin{aligned}[t] \frac{\partial \log L ( k_{3} ) }{\partial R^{\ast }} &=nz_{D}^{t}\frac{D^{\ast }\prime }{D^{\ast }}-\sum_{t=0}^{n} \frac{\Phi ( 1,\beta ,\Omega _{3}D^{\ast } ) \prime }{\Phi ( 1,\beta ,\Omega _{3}D^{\ast } ) } \\ &=nz_{D}^{t}\frac{D^{\ast }\prime }{D^{\ast }}-n \frac{\Phi ( 1,\beta ,\Omega _{3}D^{\ast } ) \prime }{\Phi ( 1,\beta ,\Omega _{3}D^{\ast } ) } , \end{aligned} \end{aligned}$$

162 $$\begin{aligned}& \begin{aligned}[t] \frac{\partial \log L ( k_{3} ) }{\partial \Omega _{3}} &=nz_{D}^{t} \frac{\Omega _{3}\prime }{\Omega _{3}}-n \frac{\Phi ( 1,\beta ,\Omega _{3}D^{\ast } ) \prime }{\Phi ( 1,\beta ,\Omega _{3}D^{\ast } ) } \\ &=-n \frac{\Phi ( 1,\beta ,\Omega _{3}D^{\ast } ) \prime }{\Phi ( 1,\beta ,\Omega _{3}D^{\ast } ) }. \end{aligned} \end{aligned}$$

## Likelihood with Weibull distribution

We will do the same routine for the Weibull distribution known as

163 $$ P ( X=k ) =\frac{k}{\alpha } \biggl( \frac{\lambda }{\alpha } \biggr) ^{k-1}\exp ( -\lambda /\alpha ) ^{k},\quad \lambda ,\alpha >0,k=0,1,2,\ldots,n, $$

Ω with parameters $k_{1}$, $k_{2}$, $k_{3}$

164 $$\begin{aligned}& k_{1} =\Omega _{1}I^{\ast } ( t ), \\& k_{2} =\Omega _{2}R^{\ast } ( t ), \\& k_{3} =\Omega _{3}D^{\ast } ( t ) \end{aligned}$$

and

165 $$\begin{aligned}& \varepsilon _{I}^{t} \sim W \bigl( k_{1}=\Omega _{1}I^{\ast } ( t ) \bigr), \\& \varepsilon _{R}^{t} \sim W \bigl( k_{2}=\Omega _{2}R^{\ast } ( t ) \bigr), \\& \varepsilon _{D}^{t} \sim W \bigl( k_{3}=\Omega _{3}D^{\ast } ( t ) \bigr) . \end{aligned}$$

Thus the likelihood function is given by

166 $$\begin{aligned}& L ( k_{1} ) =\prod_{t=0}^{n}W \bigl( \varepsilon _{I}^{t}/k_{1} \bigr), \\& L ( k_{2} ) =\prod_{t=0}^{n}W \bigl( \varepsilon _{R}^{t}/k_{2} \bigr), \\& L ( k_{3} ) =\prod_{t=0}^{n}W \bigl( \varepsilon _{D}^{t}/k_{3} \bigr) . \end{aligned}$$

Thus

167 $$\begin{aligned}& L ( k_{1} ) =\prod_{t=0}^{n} \frac{\varepsilon _{I}^{t}}{\alpha } \biggl( \frac{\lambda }{\alpha } \biggr) ^{\varepsilon _{I}^{t}-1} \exp ( -\lambda /\alpha ) ^{\varepsilon _{I}^{t}}, \\& L ( k_{2} ) =\prod_{t=0}^{n} \frac{\varepsilon _{R}^{t}}{\alpha } \biggl( \frac{\lambda }{\alpha } \biggr) ^{\varepsilon _{R}^{t}-1} \exp ( -\lambda /\alpha ) ^{\varepsilon _{R}^{t}}, \\& L ( k_{3} ) =\prod_{t=0}^{n} \frac{\varepsilon _{D}^{t}}{\alpha } \biggl( \frac{\lambda }{\alpha } \biggr) ^{\varepsilon _{D}^{t}-1} \exp ( -\lambda /\alpha ) ^{\varepsilon _{D}^{t}}. \end{aligned}$$

Without loss of generality, we consider $L ( k_{1} ) $:

168 $$\begin{aligned} \log L ( k_{1} ) =&\sum_{t=0}^{n} \log \biggl[ \frac{\varepsilon _{I}^{t}}{\alpha } \biggl( \frac{\Omega _{1}I^{\ast }}{\alpha } \biggr) ^{\varepsilon _{I}^{t}-1} \exp \bigl( -\Omega _{1}I^{\ast }/\alpha \bigr) ^{\varepsilon _{I}^{t}} \biggr] \\ =& \bigl[ \log \varepsilon _{I}^{t}-\log \alpha + \bigl( \varepsilon _{I}^{t}-1 \bigr) \bigl[ \log \bigl( \Omega _{1}I^{ \ast } \bigr) -\log \alpha \bigr] -\varepsilon _{I}^{t} \bigl( - \Omega _{1}I^{\ast }/ \alpha \bigr) \bigr] \end{aligned}$$

and

169 $$\begin{aligned}& \begin{aligned}[b] \frac{\partial \log L ( k_{1} ) }{\partial \varepsilon _{I}^{t}} &=\sum_{t=0}^{n} \frac{\varepsilon _{I}^{t}\prime }{\varepsilon _{I}^{t}}+\sum_{t=0}^{n} \bigl[ \log \bigl( \Omega _{1}I^{\ast } \bigr) - \log \alpha \bigr] -\sum_{t=0}^{n} \bigl( -\Omega _{1}I^{\ast }/\alpha \bigr) \\ &=n\frac{\varepsilon _{I}^{t}\prime }{\varepsilon _{I}^{t}}+n \bigl[ \log \bigl( \Omega _{1}I^{\ast } \bigr) -\log \alpha \bigr] +n \bigl( \Omega _{1}I^{\ast }/ \alpha \bigr), \end{aligned} \end{aligned}$$

170 $$\begin{aligned}& \begin{aligned}[t] \frac{\partial \log L ( k_{1} ) }{\partial I^{\ast }} &=n \bigl( \varepsilon _{I}^{t}-1 \bigr) \frac{I^{\ast }\prime }{I^{\ast }}-\sum _{t=0}^{n} \frac{ ( -\Omega _{1}I^{\ast }/\alpha ) \prime }{ ( -\Omega _{1}I^{\ast }/\alpha ) } \\ &=n \bigl( \varepsilon _{I}^{t}-1 \bigr) \frac{I^{\ast }\prime }{I^{\ast }}-n\frac{ ( -\Omega _{1}I^{\ast }/\alpha ) \prime }{ ( -\Omega _{1}I^{\ast }/\alpha ) }, \end{aligned} \end{aligned}$$

171 $$\begin{aligned}& \begin{aligned}[t] \frac{\partial \log L ( k_{1} ) }{\partial \Omega _{1}} &=n \bigl( \varepsilon _{I}^{t}-1 \bigr) \frac{\Omega _{1}\prime }{\Omega _{1}}-n \frac{ ( -\Omega _{1}I^{\ast }/\alpha ) \prime }{ ( -\Omega _{1}I^{\ast }/\alpha ) } \\ &=-n \frac{ ( -\Omega _{1}I^{\ast }/\alpha ) \prime }{ ( -\Omega _{1}I^{\ast }/\alpha ) } , \end{aligned} \end{aligned}$$

172 $$\begin{aligned}& \begin{aligned}[b] \log L ( k_{2} ) &=\sum _{t=0}^{n}\log \biggl[ \frac{\varepsilon _{R}^{t}}{\alpha } \biggl( \frac{\Omega _{2}I^{\ast }}{\alpha } \biggr) ^{\varepsilon _{R}^{t}-1} \exp \bigl( -\Omega _{2}R^{\ast }/\alpha \bigr) ^{\varepsilon _{I}^{t}} \biggr] \\ &= \bigl[ \log \varepsilon _{R}^{t}-\log \alpha + \bigl( \varepsilon _{R}^{t}-1 \bigr) \bigl[ \log \bigl( \Omega _{2}R^{ \ast } \bigr) -\log \alpha \bigr] -\varepsilon _{R}^{t} \bigl( - \Omega _{2}R^{\ast }/ \alpha \bigr) \bigr] \end{aligned} \end{aligned}$$

and

173 $$\begin{aligned}& \begin{aligned}[b] \frac{\partial \log L ( k_{2} ) }{\partial \varepsilon _{R}^{t}} &=\sum_{t=0}^{n} \frac{\varepsilon _{R}^{t}\prime }{\varepsilon _{R}^{t}}+\sum_{t=0}^{n} \bigl[ \log \bigl( \Omega _{2}R^{\ast } \bigr) - \log \alpha \bigr] -\sum_{t=0}^{n} \bigl( -\Omega _{2}R^{\ast }/\alpha \bigr) \\ &=n\frac{\varepsilon _{R}^{t}\prime }{\varepsilon _{R}^{t}}+n \bigl[ \log \bigl( \Omega _{2}R^{\ast } \bigr) -\log \alpha \bigr] +n \bigl( -\Omega _{2}R^{\ast }/ \alpha \bigr), \end{aligned} \end{aligned}$$

174 $$\begin{aligned}& \begin{aligned}[t] \frac{\partial \log L ( k_{2} ) }{\partial R^{\ast }} &=n \bigl( \varepsilon _{R}^{t}-1 \bigr) \frac{R^{\ast }\prime }{R^{\ast }}-\sum _{t=0}^{n} \frac{ ( -\Omega _{2}R^{\ast }/\alpha ) \prime }{ ( -\Omega _{2}R^{\ast }/\alpha ) } \\ &=n \bigl( \varepsilon _{R}^{t}-1 \bigr) \frac{R^{\ast }\prime }{R^{\ast }}-n\frac{ ( -\Omega _{2}R^{\ast }/\alpha ) \prime }{ ( -\Omega _{2}R^{\ast }/\alpha ) } , \end{aligned} \end{aligned}$$

175 $$\begin{aligned}& \begin{aligned}[t] \frac{\partial \log L ( k_{2} ) }{\partial \Omega _{2}} &=n \bigl( \varepsilon _{R}^{t}-1 \bigr) \frac{\Omega _{2}\prime }{\Omega _{2}}-n \frac{ ( -\Omega _{2}R^{\ast }/\alpha ) \prime }{ ( -\Omega _{2}R^{\ast }/\alpha ) } \\ &=-n \frac{ ( -\Omega _{2}R^{\ast }/\alpha ) \prime }{ ( -\Omega _{2}R^{\ast }/\alpha ) }, \end{aligned} \end{aligned}$$

176 $$\begin{aligned}& \begin{aligned}[t] \log L ( k_{3} ) &=\sum _{t=0}^{n}\log \biggl[ \frac{\varepsilon _{D}^{t}}{\alpha } \biggl( \frac{\Omega _{3}D^{\ast }}{\alpha } \biggr) ^{\varepsilon _{D}^{t}-1} \exp \bigl( -\Omega _{3}D^{\ast }/\alpha \bigr) ^{\varepsilon _{D}^{t}} \biggr] \\ &= \bigl[ \log \varepsilon _{D}^{t}-\log \alpha + \bigl( \varepsilon _{D}^{t}-1 \bigr) \bigl[ \log \bigl( \Omega _{3}D^{ \ast } \bigr) -\log \alpha \bigr] -\varepsilon _{D}^{t} \bigl( - \Omega _{3}D^{\ast }/ \alpha \bigr) \bigr] \end{aligned} \end{aligned}$$

and

177 $$\begin{aligned}& \begin{aligned}[b] \frac{\partial \log L ( k_{3} ) }{\partial \varepsilon _{D}^{t}} &=\sum_{t=0}^{n} \frac{\varepsilon _{D}^{t}\prime }{\varepsilon _{D}^{t}}+\sum_{t=0}^{n} \bigl[ \log \bigl( \Omega _{3}D^{\ast } \bigr) - \log \alpha \bigr] -\sum_{t=0}^{n} \bigl( -\Omega _{3}D^{\ast }/\alpha \bigr) \\ &=n\frac{\varepsilon _{D}^{t}\prime }{\varepsilon _{D}^{t}}+n \bigl[ \log \bigl( \Omega _{3}R^{\ast } \bigr) -\log \alpha \bigr] +n \bigl( -\Omega _{3}D^{\ast }/ \alpha \bigr), \end{aligned} \end{aligned}$$

178 $$\begin{aligned}& \begin{aligned}[t] \frac{\partial \log L ( k_{3} ) }{\partial D^{\ast }} &=n \bigl( \varepsilon _{D}^{t}-1 \bigr) \frac{D^{\ast }\prime }{D^{\ast }}-\sum _{t=0}^{n} \frac{ ( -\Omega _{3}D^{\ast }/\alpha ) \prime }{ ( -\Omega _{3}D^{\ast }/\alpha ) } \\ &=n \bigl( \varepsilon _{I}^{t}-1 \bigr) \frac{D^{\ast }\prime }{D^{\ast }}-n\frac{ ( -\Omega _{3}D^{\ast }/\alpha ) \prime }{ ( -\Omega _{3}D^{\ast }/\alpha ) } , \end{aligned} \end{aligned}$$

179 $$\begin{aligned}& \begin{aligned}[t] \frac{\partial \log L ( k_{1} ) }{\partial \Omega _{1}} &=n \bigl( \varepsilon _{D}^{t}-1 \bigr) \frac{\Omega _{3}\prime }{\Omega _{3}}-n \frac{ ( -\Omega _{3}D^{\ast }/\alpha ) \prime }{ ( -\Omega _{3}D^{\ast }/\alpha ) } \\ &=-n \frac{ ( -\Omega _{3}D^{\ast }/\alpha ) \prime }{ ( -\Omega _{3}D^{\ast }/\alpha ) }. \end{aligned} \end{aligned}$$

## Likelihood with Mittag-Leffler distribution

Finally, we shall use the Mittag-Leffler distribution for similar processes. The Mittag-Leffler distribution is defined by

180 $$ P ( X=k ) = \frac{\lambda ^{k}}{\Gamma ( \alpha k+\beta ) E_{\alpha ,\beta } ( \lambda ) },\quad \lambda >0,k=0,1,2,\ldots,n, $$

where

181 $$ E_{\alpha ,\beta } ( \lambda ) =\sum_{k=0}^{\infty } \frac{\lambda ^{k}}{\Gamma ( \alpha k+\beta ) }. $$

$\Omega_{i}$, $i=1,2,3$ with parameters $k_{1}$, $k_{2}$, $k_{3}$

182 $$\begin{aligned}& k_{1} =\Omega _{1}I^{\ast } ( t ), \\& k_{2} =\Omega _{2}R^{\ast } ( t ), \\& k_{3} =\Omega _{3}D^{\ast } ( t ) \end{aligned}$$

and

183 $$\begin{aligned}& \varepsilon _{I}^{t} \sim ML \bigl( k_{1}= \Omega _{1}I^{\ast } ( t ) \bigr), \\& \varepsilon _{R}^{t} \sim ML \bigl( k_{2}= \Omega _{2}R^{\ast } ( t ) \bigr), \\& \varepsilon _{D}^{t} \sim ML \bigl( k_{3}= \Omega _{3}D^{\ast } ( t ) \bigr) . \end{aligned}$$

Thus the likelihood function is written as

184 $$\begin{aligned}& L ( k_{1} ) =\prod_{t=0}^{n}ML \bigl( \varepsilon _{I}^{t}/k_{1} \bigr), \\& L ( k_{2} ) =\prod_{t=0}^{n}ML \bigl( \varepsilon _{R}^{t}/k_{2} \bigr), \\& L ( k_{3} ) =\prod_{t=0}^{n}ML \bigl( \varepsilon _{D}^{t}/k_{3} \bigr) . \end{aligned}$$

Thus

185 $$\begin{aligned}& L ( k_{1} ) =\prod_{t=0}^{n} \frac{\lambda ^{\varepsilon _{I}^{t}}}{\Gamma ( \alpha \varepsilon _{I}^{t}+\beta ) E_{\alpha ,\beta } ( \lambda ) }, \\& L ( k_{2} ) =\prod_{t=0}^{n} \frac{\lambda ^{\varepsilon _{R}^{t}}}{\Gamma ( \alpha \varepsilon _{R}^{t}+\beta ) E_{\alpha ,\beta } ( \lambda ) }, \\& L ( k_{3} ) =\prod_{t=0}^{n} \frac{\lambda ^{\varepsilon _{D}^{t}}}{\Gamma ( \alpha \varepsilon _{D}^{t}+\beta ) E_{\alpha ,\beta } ( \lambda ) }. \end{aligned}$$

We write $L ( k_{1} ) $:

186 $$\begin{aligned} \log L ( k_{1} ) =&\log \frac{\lambda ^{\varepsilon _{I}^{t}}}{\Gamma ( \alpha \varepsilon _{I}^{t}+\beta ) E_{\alpha ,\beta } ( \lambda ) } \\ =&\sum_{t=0}^{n} \bigl[ \varepsilon _{I}^{t}\log \bigl( \Omega _{1}I^{ \ast } \bigr) -\log \Gamma \bigl( \alpha \varepsilon _{I}^{t}+ \beta \bigr) -\log E_{\alpha ,\beta } \bigl( \Omega _{1}I^{\ast } \bigr) \bigr] \end{aligned}$$

and

187 $$\begin{aligned}& \begin{aligned} \frac{\partial \log L ( k_{1} ) }{\partial \varepsilon _{I}^{t}} &=\sum_{t=0}^{n} \log ( \Omega _{1} ) +\sum_{t=0}^{n} \log \bigl( I^{\ast } \bigr) -\sum_{t=0}^{n} \frac{ ( \Gamma ( \alpha \varepsilon _{I}^{t}+\beta ) ) \prime }{\Gamma ( \alpha \varepsilon _{I}^{t}+\beta ) } \\ &=n \bigl[ \log ( \Omega _{1} ) +\log \bigl( I^{\ast } \bigr) \bigr] -\sum_{t=0}^{n} \frac{ ( \Gamma ( \alpha \varepsilon _{I}^{t}+\beta ) ) \prime }{\Gamma ( \alpha \varepsilon _{I}^{t}+\beta ) } \\ &=n\log \bigl( \Omega _{1}I^{\ast } \bigr) -\sum _{t=0}^{n} \frac{ ( \Gamma ( \alpha \varepsilon _{I}^{t}+\beta ) ) \prime }{\Gamma ( \alpha \varepsilon _{I}^{t}+\beta ) } , \end{aligned} \end{aligned}$$

188 $$\begin{aligned}& \begin{aligned}[t] \frac{\partial \log L ( k_{1} ) }{\partial I^{\ast }} &=n \varepsilon _{I}^{t} \frac{I^{\ast }\prime }{I^{\ast }}-\sum_{t=0}^{n}\frac{E_{\alpha ,\beta } ( \Omega _{1}I^{\ast } ) \prime }{E_{\alpha ,\beta } ( \Omega _{1}I^{\ast } ) } \\ &=n\varepsilon _{I}^{t}\frac{I^{\ast }\prime }{I^{\ast }}-n \frac{E_{\alpha ,\beta } ( \Omega _{1}I^{\ast } ) \prime }{E_{\alpha ,\beta } ( \Omega _{1}I^{\ast } ) } , \end{aligned} \end{aligned}$$

189 $$\begin{aligned}& \begin{aligned}[t] \frac{\partial \log L ( k_{1} ) }{\partial \Omega _{1}} &=n \varepsilon _{I}^{t} \frac{\Omega _{1}\prime }{\Omega _{1}}-n \frac{E_{\alpha ,\beta } ( \Omega _{1}I^{\ast } ) \prime }{E_{\alpha ,\beta } ( \Omega _{1}I^{\ast } ) } \\ &=-n \frac{E_{\alpha ,\beta } ( \Omega _{1}I^{\ast } ) \prime }{E_{\alpha ,\beta } ( \Omega _{1}I^{\ast } ) }. \end{aligned} \end{aligned}$$

With the same routine,

190 $$\begin{aligned} \log L ( k_{2} ) =&\sum_{t=0}^{n} \log \frac{\lambda ^{\varepsilon _{R}^{t}}}{\Gamma ( \alpha \varepsilon _{I}^{t}+\beta ) E_{\alpha ,\beta } ( \lambda ) } \\ =&\sum_{t=0}^{n} \bigl[ \varepsilon _{R}^{t}\log \bigl( \Omega _{1}R^{ \ast } \bigr) -\log \Gamma \bigl( \alpha \varepsilon _{R}^{t}+ \beta \bigr) -\log E_{\alpha ,\beta } \bigl( \Omega _{2}R^{\ast } \bigr) \bigr] \end{aligned}$$

and

191 $$\begin{aligned}& \begin{aligned} \frac{\partial \log L ( k_{2} ) }{\partial \varepsilon _{R}^{t}} &=\sum_{t=0}^{n} \log ( \Omega _{2} ) +\sum_{t=0}^{n} \log \bigl( R^{\ast } \bigr) -\sum_{t=0}^{n} \frac{ ( \Gamma ( \alpha \varepsilon _{R}^{t}+\beta ) ) \prime }{\Gamma ( \alpha \varepsilon _{R}^{t}+\beta ) } \\ &=n \bigl[ \log ( \Omega _{2} ) +\log \bigl( R^{\ast } \bigr) \bigr] -\sum_{t=0}^{n} \frac{ ( \Gamma ( \alpha \varepsilon _{R}^{t}+\beta ) ) \prime }{\Gamma ( \alpha \varepsilon _{R}^{t}+\beta ) } \\ &=n\log \bigl( \Omega _{2}R^{\ast } \bigr) -\sum _{t=0}^{n} \frac{ ( \Gamma ( \alpha \varepsilon _{R}^{t}+\beta ) ) \prime }{\Gamma ( \alpha \varepsilon _{R}^{t}+\beta ) } , \end{aligned} \end{aligned}$$

192 $$\begin{aligned}& \begin{aligned}[t] \frac{\partial \log L ( k_{2} ) }{\partial R^{\ast }} &=n \varepsilon _{R}^{t} \frac{R^{\ast }\prime }{R^{\ast }}-\sum_{t=0}^{n}\frac{E_{\alpha ,\beta } ( \Omega _{2}R^{\ast } ) \prime }{E_{\alpha ,\beta } ( \Omega _{2}R^{\ast } ) } \\ &=n\varepsilon _{I}^{t}\frac{R^{\ast }\prime }{R^{\ast }}-n \frac{E_{\alpha ,\beta } ( \Omega _{2}R^{\ast } ) \prime }{E_{\alpha ,\beta } ( \Omega _{2}R^{\ast } ) } , \end{aligned} \end{aligned}$$

193 $$\begin{aligned}& \begin{aligned}[t] \frac{\partial \log L ( k_{2} ) }{\partial \Omega _{2}} &=n \varepsilon _{R}^{t} \frac{\Omega _{2}\prime }{\Omega _{2}}-n \frac{E_{\alpha ,\beta } ( \Omega _{2}R^{\ast } ) \prime }{E_{\alpha ,\beta } ( \Omega _{2}R^{\ast } ) } \\ &=-n \frac{E_{\alpha ,\beta } ( \Omega _{2}R^{\ast } ) \prime }{E_{\alpha ,\beta } ( \Omega _{2}R^{\ast } ) } \end{aligned} \end{aligned}$$

and

194 $$\begin{aligned} \log L ( k_{3} ) =&\sum_{t=0}^{n} \log \frac{\lambda ^{\varepsilon _{D}^{t}}}{\Gamma ( \alpha \varepsilon _{D}^{t}+\beta ) E_{\alpha ,\beta } ( \lambda ) } \\ =&\sum_{t=0}^{n} \bigl[ \varepsilon _{D}^{t}\log \bigl( \Omega _{3}D^{ \ast } \bigr) -\log \Gamma \bigl( \alpha \varepsilon _{D}^{t}+ \beta \bigr) -\log E_{\alpha ,\beta } \bigl( \Omega _{3}D^{\ast } \bigr) \bigr] \end{aligned}$$

and

195 $$\begin{aligned}& \begin{aligned} \frac{\partial \log L ( k_{1} ) }{\partial \varepsilon _{I}^{t}} &=\sum_{t=0}^{n} \log ( \Omega _{3} ) +\sum_{t=0}^{n} \log \bigl( D^{\ast } \bigr) -\sum_{t=0}^{n} \frac{ ( \Gamma ( \alpha \varepsilon _{D}^{t}+\beta ) ) \prime }{\Gamma ( \alpha \varepsilon _{D}^{t}+\beta ) } \\ &=n \bigl[ \log ( \Omega _{3} ) +\log \bigl( D^{\ast } \bigr) \bigr] -\sum_{t=0}^{n} \frac{ ( \Gamma ( \alpha \varepsilon _{D}^{t}+\beta ) ) \prime }{\Gamma ( \alpha \varepsilon _{D}^{t}+\beta ) } \\ &=n\log \bigl( \Omega _{3}D^{\ast } \bigr) -\sum _{t=0}^{n} \frac{ ( \Gamma ( \alpha \varepsilon _{D}^{t}+\beta ) ) \prime }{\Gamma ( \alpha \varepsilon _{D}^{t}+\beta ) } , \end{aligned} \end{aligned}$$

196 $$\begin{aligned}& \begin{aligned}[t] \frac{\partial \log L ( k_{3} ) }{\partial D^{\ast }} &=n \varepsilon _{D}^{t} \frac{D^{\ast }\prime }{D^{\ast }}-\sum_{t=0}^{n}\frac{E_{\alpha ,\beta } ( \Omega _{3}D^{\ast } ) \prime }{E_{\alpha ,\beta } ( \Omega _{3}D^{\ast } ) } \\ &=n\varepsilon _{D}^{t}\frac{D^{\ast }\prime }{D^{\ast }}-n \frac{E_{\alpha ,\beta } ( \Omega _{3}D^{\ast } ) \prime }{E_{\alpha ,\beta } ( \Omega _{3}D^{\ast } ) } , \end{aligned} \end{aligned}$$

197 $$\begin{aligned}& \begin{aligned}[t] \frac{\partial \log L ( k_{3} ) }{\partial \Omega _{3}} &=n \varepsilon _{D}^{t} \frac{\Omega _{1}\prime }{\Omega _{1}}-n \frac{E_{\alpha ,\beta } ( \Omega _{3}D^{\ast } ) \prime }{E_{\alpha ,\beta } ( \Omega _{3}D^{\ast } ) } \\ &=-n \frac{E_{\alpha ,\beta } ( \Omega _{3}D^{\ast } ) \prime }{E_{\alpha ,\beta } ( \Omega _{3}D^{\ast } ) }. \end{aligned} \end{aligned}$$

## Conclusion

Up to date humans have relied on forecasting with the aim to better control their world, or at least to have an asymptotic idea of their future. They have many ways to achieve this, one way is to use the deterministic approach and another is stochastic one. In this work, we presented a comprehensive analysis ranging from stochastic, fractal to differentiation with the aim to predict the future behavior of COVID-19 with cases studied in Africa and Europe. With stochastic approach, we were able to detect a possibility of the second wave of COVID-19 spread in Europe and in Africa, a continuous exponential growth could be possible. We presented an extension of the blancmange function to capture more fractal behaviors, and some examples were presented resembling the COVID-19 spread in various countries in Africa and Europe. A complex and nonlinear mathematical model with wave function was considered and solved numerically with a modified scheme.

## Data Availability

There are no data for this paper.
